# Competitive Endogenous RNA Network Construction and Comparison of Lung Squamous Cell Carcinoma in Smokers and Nonsmokers

**DOI:** 10.1155/2019/5292787

**Published:** 2019-12-04

**Authors:** Yan Yao, Tingting Zhang, Lingyu Qi, Ruijuan Liu, Gongxi Liu, Xue Wang, Jie Li, Jia Li, Changgang Sun

**Affiliations:** ^1^Clinical Medical Colleges, Weifang Medical University, No. 7166, Baotong Western Street, Weifang, Shandong Province, China; ^2^College of First Clinical Medicine, Shandong University of Traditional Chinese Medicine, No. 16369, Jingshi Road, Jinan, Shandong Province, China; ^3^Department of Oncology, Weifang Traditional Chinese Hospital, No. 1055, Weizhou Road, Weifang, Shandong Province, China; ^4^Medical Colleges, Qingdao University, No. 308, Ningxia Road, Shinan District, Qingdao, Shandong Province, China; ^5^Department of Oncology, Affiliated Hospital of Qingdao University, No. 16 Jiangsu Road, Shinan District, Qingdao, Shandong Province, China; ^6^Innovative Institute of Chinese Medicine and Pharmacy, Shandong University of Traditional Chinese Medicine, Jinan, 250014 Shandong, China

## Abstract

**Background:**

Lung squamous cell carcinoma (LUSC) is a subtype of highly malignant lung cancer with poor prognosis, for which smoking is the main risk factor. However, the underlying genetic and molecular mechanisms of smoking-related LUSC remain largely unknown.

**Methods:**

We mined existing LUSC-related mRNA, miRNA, and lncRNA transcriptome data and corresponding clinical data from The Cancer Genome Atlas (TCGA) database and divided them into smoking and nonsmoking groups, followed by differential expression analysis. Functional enrichment analysis of the unique differentially expressed mRNAs of the two groups was performed using the DAVID database. Subsequently, the lncRNA-miRNA-mRNA competing endogenous RNA (ceRNA) network of LUSC in smoking and nonsmoking groups was constructed. Finally, survival analyses were performed to determine the effects of differentially expressed lncRNAs/mRNAs/miRNAs that were involved in the ceRNA network on overall survival and to discover the hub genes.

**Results:**

A total of 1696 lncRNAs, 125 miRNAs, and 3246 mRNAs and 1784 lncRNAs, 96 miRNAs, and 3229 mRNAs with differentially expressed profiles were identified in the smoking and nonsmoking groups, respectively. The ceRNA network and survival analysis revealed four lncRNAs (LINC00466, DLX6-AS1, LINC00261, and AGBL1), one miRNA (hsa-mir-210), and two mRNAs (CITED2 and ENPP4), with the potential as biomarkers for smoking-related LUSC diagnosis and prognosis.

**Conclusion:**

Taken together, our research has identified the differences in the ceRNA regulatory networks between smoking and nonsmoking LUSC, which could lay the foundation for future clinical research.

## 1. Introduction

Lung cancer accounts for the highest incidence of all malignant tumors and is the leading cause of cancer-related death worldwide [1]. It is a disease that poses great health and life threat to the population. Lung squamous cell carcinoma (LUSC) is the main histological subtype accounting for 30%-51% of lung cancer cases [2]. The cause is still not completely clear. A large amount of data indicates that smoking is the main factor influencing the risk of lung cancer, with 90% of these cases and 80% of lung cancer deaths attributed to cigarette smoking [3–5].

Cigarette smoke contains more than 5000 chemicals and over 60 carcinogens. The major carcinogens include nicotine-derived nitrosamine ketone (NNK) and polycyclic aromatic hydrocarbons (PAH), which can induce lung cancer formation in fully immune laboratory animals [6, 7]. The effect of smoking on lung cancer is not only related to genetic mutations but also leads to insensitive EGFR tyrosine kinase inhibitor (TKIs) and poor progression-free survival [8, 9]. In the past few years, researchers have attempted to provide novel insights into the molecular mechanisms of LUSC. However, the underlying molecular mechanism remains unclear. Therefore, it is imperative to find out how smoking-related carcinogens alter the intracellular signaling pathways and gene mechanisms in LUSC.

Only 2% of the human transcriptome is composed of protein-encoding RNAs, and the remaining 98% are noncoding RNAs. Recently, there has been increasing evidence that many noncoding RNAs are major regulators of target gene expression levels [10, 11]. Among them, although long noncoding RNAs (lncRNA) have little or no protein coding ability, they play an important role in many biological processes such as transcription, splicing, and translation and have the potential to be used as a biomarker for diagnosis and prognosis [12, 13]. In 2011, Prof. Pandolfi proposed the hypothesis of competing endogenous RNAs (ceRNAs) [14]. Subsequently, a large number of studies confirmed that ceRNA could regulate human cells [15, 16]. Messenger RNA (mRNA) and lncRNA share one or more miRNA response elements (MREs) and may act as natural microRNA (miRNA) sponges that lead to the downregulation of the intracellular miRNA function [17]. Studies have found that various types of RNAs, such as mRNA, pseudogenes, lncRNA, circular RNA, and miRNA, can communicate with each other through the ceRNA mechanisms, thereby regulating various tumor cells and their microenvironment and affecting tumor proliferation and migration [18]. Therefore, an imbalance in the ceRNA network may lead to the pathogenesis of the disease.

Recent studies have indicated that HMGA2 and LINC00858 function as ceRNA to promote lung cancer progression and MTAT rs1061451 was a protective factor of non-small-cell lung cancer (NSCLC) [19–21]. miRNA expression is heterogeneous in NSCLC patients in the smoking state. miR-574-5p, miR-874, and miR-361-3p dysregulation was associated with metastasis of non-small-cell lung cancer (NSCLC) [22, 23]. miR-21 and miR-10a could be used as predictive markers for overall survival and disease-free progression of lung cancer patients [24]. However, the differences between the complex RNA mechanisms among smoking and nonsmoking LUSC patients have not been fully explored, highlighting the importance of studying the ceRNA networks for smoking-related LUSC.

In the present study, we obtained the LUSC-related mRNA, miRNA, and lncRNA transcriptome data and the corresponding clinical data from The Cancer Genome Atlas (TCGA) database. It was divided into two groups based on smoking. In the next step, the differentially expressed lncRNA, miRNAs, and mRNA were analyzed. Based on a composite profile of the data from these three RNAs, we constructed a ceRNA coexpression network of LUSC. To the best of our knowledge, this is the first study to investigate the smoking-related LUSC ceRNA network. Finally, we found that some RNAs were closely related to smoking in patients with LUSC. The results of this study help elaborate the underlying mechanisms in LUSC through ceRNA networks, identify potential therapeutic and prognostic target genes, and provide new directions for future research.

## 2. Materials and Methods

### 2.1. Patient Datasets and Data Preprocessing

Sequencing data of the three types of RNAs in lung squamous cell carcinoma and adjacent noncancerous tissues and their corresponding clinical data was obtained from The Cancer Genome Atlas (TCGA) database. Integration of this RNA data and extraction of the lncRNA expression profiles was done using the R bioconductor package TCGABiolinks [25]. Genes were annotated using the Ensembl online database (http://www.ensembl.org). Based on the clinical data, we divided the samples into two groups, smoking and nonsmoking. Finally, we obtained lncRNA, miRNA, and mRNA expression profiles of the two groups. This study was in compliance with the publication guidelines provided by TCGA, and the data obtained from TCGA did not require approval from the ethics committee.

### 2.2. Differential Expression Analysis

We utilized the R Bioconductor package edgeR to identify the differentially expressed lncRNAs (DElncRNA), miRNAs (DEmiRNA), and mRNAs (DEmRNA) [26]. Filtering criteria for the differential expression of these three RNAs in the smoking and nonsmoking groups were as follows. (1) At least 25% of the samples have a gene expression raw count level greater than 2. (2) The original RNA sequencing was normalized using the trimmed mean of M-values (TMM) method. (3) Thresholds of ∣log2 fold change∣ > 2 and false discovery rate (FDR) or adjusted *P* value < 0.01. We visualized the corresponding heat map and clustering results using gplots package in R.

### 2.3. Functional Enrichment Analysis

In order to annotate the different underlying biological processes of dysregulated mRNAs in the smoking group, compared to the nonsmoking group, we removed the DEmRNAs that were common in both groups. The Integrate Discovery Database (DAVID 6.8) (https://david.abcc.ncifcrf.gov/) [27] was used to perform Gene Ontology.

GO and Kyoto Encyclopedia of Genes and Genomes (KEGG) pathway analysis was performed for the remaining dysregulated genes. The adjusted *P* value of less than 0.05 was considered meaningful. Ggplot2 and GOplot R packages were used to visualize the results [28].

### 2.4. ceRNA Network Construction

We constructed a ceRNA network in accordance with the theory that lncRNA can affect miRNAs and further regulate mRNA expression by acting as a miRNA sponge. First, we decoded the miRNA sequences by using the starBase v2.0 database (http://starbase.sysu.edu.cn) [29] and successfully paired DEmiRNAs 3p or 5p transcript information. The miRcode database (http://www.mircode.org) [30] and DIANA-LncBase v2 [31] were used to construct lncRNA-miRNA interaction pairs. miRDB (http://www.mirdb.org/) [32], miRTarBase (http://mirtarbase.mbc.nctu.edu.tw/) [33], and TargetScan (http://www.targetscan.org/) [34] were used to predict target genes of the DEmiRNAs and establish miRNA-mRNA interaction pairs. To increase the reliability of the results, only genes present in all three databases were regarded as target genes of these DEmiRNAs. To compare the target genes with DEmRNAs, we used the Venny online website, and only the overlapping portions of the genes and their interaction pairs were further analyzed. Then, based on the lncRNA-miRNA pairs and miRNA-mRNA pairs, we established the lncRNA-miRNA-mRNA ceRNA network, and the results of the smoking and nonsmoking groups were visualized using the Cytoscape v3.6.1 software [35]. Finally, the top analysis was performed by using the APP CentiScaPe [36], and the hub genes were selected from the ceRNA network with a degree of ≥5 as standard.

### 2.5. Overall Survival Analysis

To determine the relationship between prognosis and differentially expressed RNA signatures, we divided the LUSC samples into two groups: high or low, based on tumor expression data for each type of RNA in the ceRNA network. Survival analysis was performed using a standard Kaplan–Meier univariate curve with the “survival” package in R3.3.2. *P* values < 0.05 were considered statistically significant.

## 3. Results

### 3.1. Patient Characteristics and Differentially Expressed RNAs

**Figure 1:**
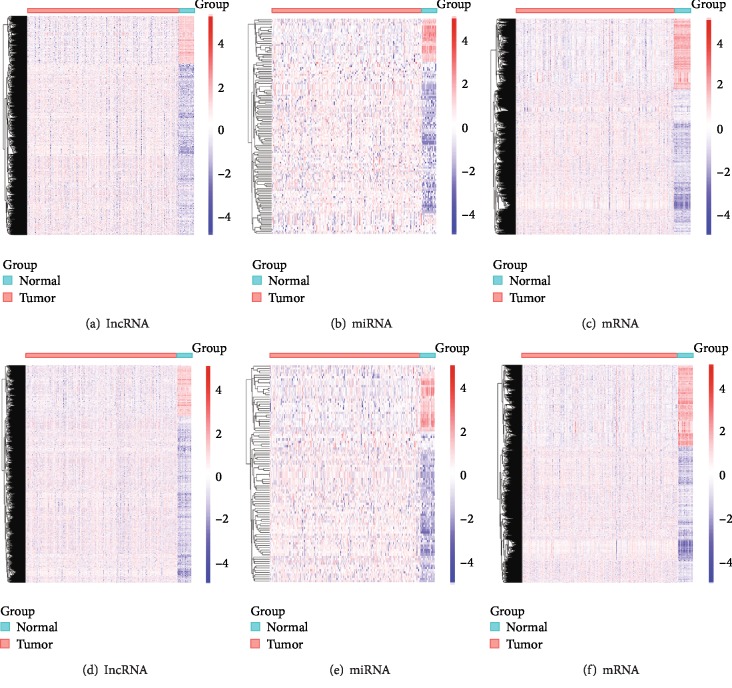
Clustered heat maps of the differentially expressed RNAs in LUSC. The rows represent RNAs and columns represent the samples; ∣log2FC∣ > 2, FDR < 0.01. (a–c) Differentially expressed lncRNAs, miRNAs, and mRNAs in smoking LUSC. (d–f) Differentially expressed lncRNAs, miRNAs, and mRNAs in nonsmoking LUSC. FC: fold change; FDR: false discovery rate; LUSC: lung squamous cell carcinoma; lncRNAs: long noncoding RNAs; miRNAs: microRNAs; mRNAs: messenger RNAs.

**Figure 2:**
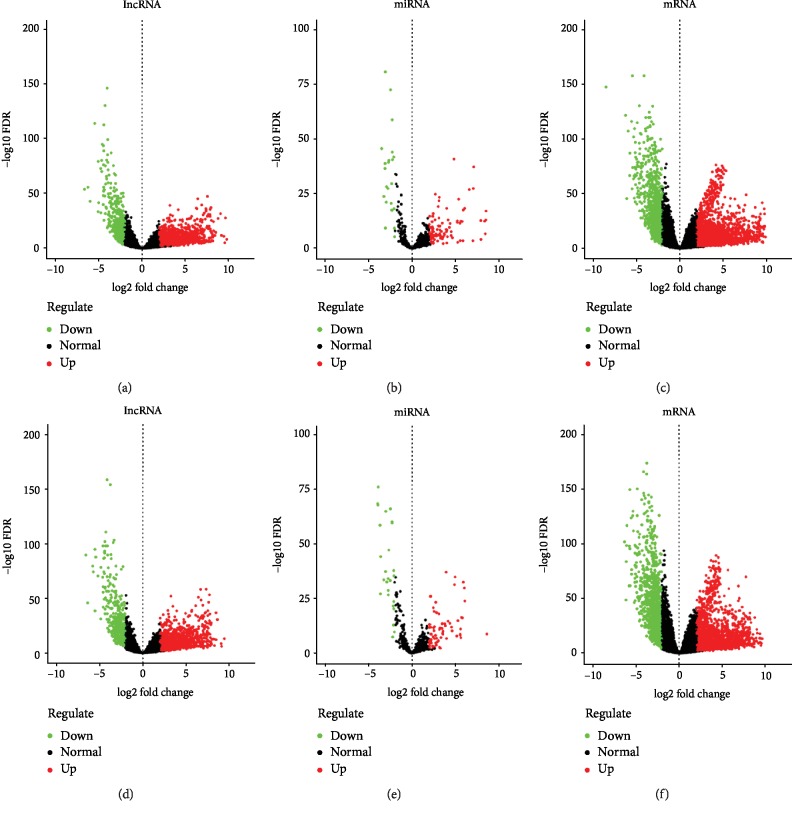
Volcano plot of differentially expressed RNAs in LUSC; ∣log2FC∣ > 2, FDR < 0.01. (a–c) Differentially expressed lncRNAs, miRNAs, and mRNAs in smoking LUSC. (d–f) Differentially expressed lncRNAs, miRNAs, and mRNAs in nonsmoking LUSC. The red dot represents upregulated RNAs and green dot represents downregulated RNAs.

The LUSC transcriptome profiling data and the corresponding clinical information were obtained using R bioconductor package TCGABiolinks. A total of 551 gene expression quantification data, which included 245 smoking samples and 306 nonsmoking samples, and 523 miRNA expression quantification data, which included 229 smoking samples and 294 nonsmoking samples, were used for this study. Using the edgeR package of R, with the cut-off criteria of ∣log2FC∣ > 2 and FDR < 0.01, we identified 1324 upregulated lncRNAs, 372 downregulated lncRNAs, 100 upregulated miRNAs, 25 downregulated miRNAs, 2179 upregulated mRNAs, and 1067 downregulated mRNAs in the smoking group (Supplementary Tables [Supplementary-material supplementary-material-1]–[Supplementary-material supplementary-material-1]). 1345 upregulated lncRNAs, 403 downregulated lncRNAs, 72 upregulated miRNAs, 26 downregulated miRNAs, 2026 upregulated mRNAs, and 1203 downregulated mRNAs were identified in the nonsmoking group (Supplementary Tables [Supplementary-material supplementary-material-1]–[Supplementary-material supplementary-material-1]). Heat maps and volcano plots of the differences between lncRNA, miRNA, and mRNA expression among the smoking and nonsmoking groups are shown in Figures 1 and 2.

### 3.2. GO and KEGG Pathway Analysis

**Figure 3:**
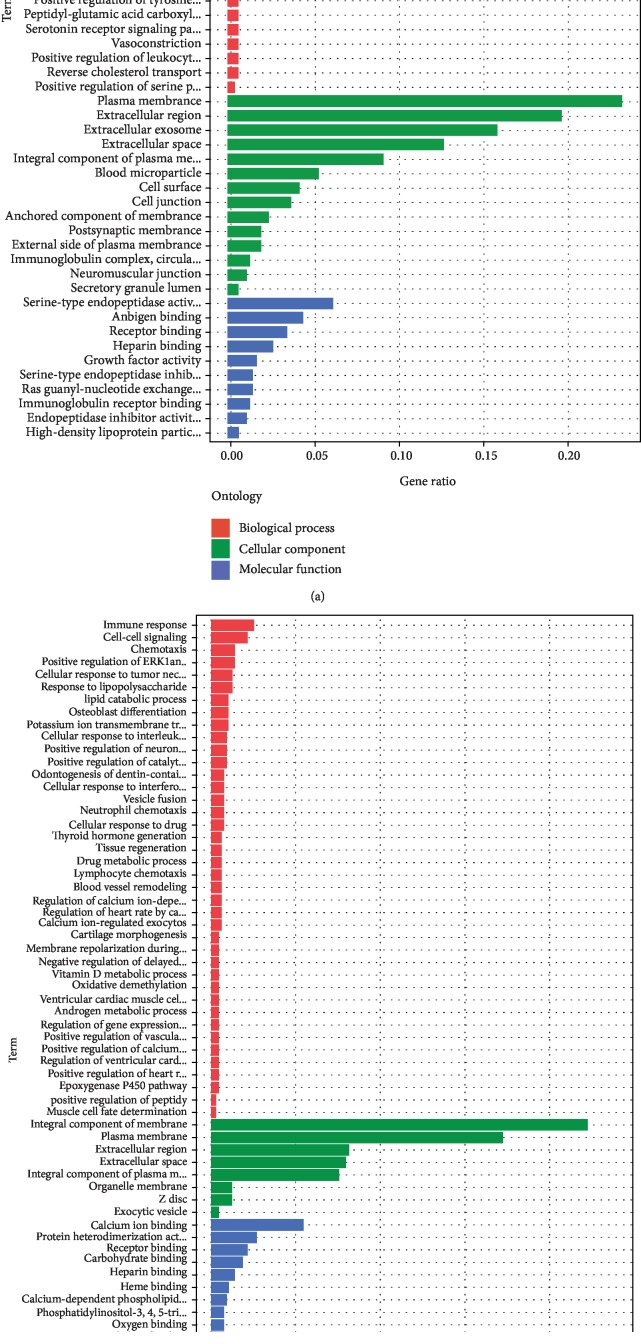
GO analysis of unique differentially expressed mRNAs (DEmRNAs) in smoking and nonsmoking LUSC. (a) Unique DEmRNA results of GO analysis in smoking LUSC. (b) Unique DEmRNA results of GO analysis in nonsmoking LUSC.

**Figure 4:**
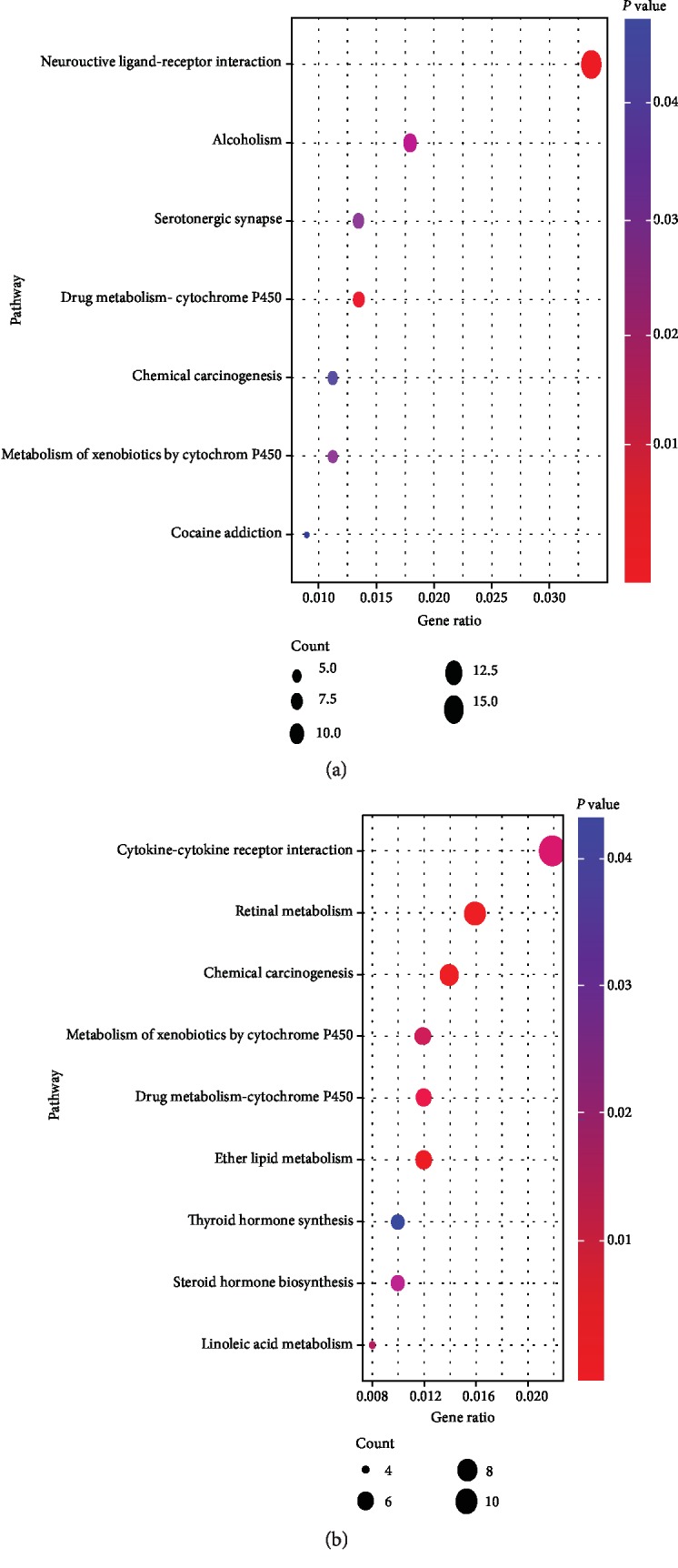
KEGG pathways analysis of unique differentially expressed mRNAs (DEmRNAs) in smoking and nonsmoking LUSC. (a) Unique DEmRNA results of KEGG pathways in smoking LUSC. (b) Unique DEmRNA results of KEGG pathways in nonsmoking LUSC.

In order to enhance our understanding of the functions of DEmRNAs between the smoking and nonsmoking groups, we removed the DEmRNAs common in both groups. As a result, 423 DEmRNAs in the smoking group and 406 DEmRNAs in the nonsmoking group were included in the functional enrichment analysis. The results showed unique GO terms in the smoking LUSC group. Biological process (BP) mainly included cell differentiation, phagocytosis, recognition, immune response, complement activation, and classical pathway; cell components (CC) mainly included extracellular region, immunoglobulin complex, circulating, plasma membrane, and cell junction; and molecular functions (MF) mainly included antigen binding, immunoglobulin receptor binding, endopeptidase inhibitor activity, and growth factor activity. In the nonsmoking LUSC group, BP mainly included cell-cell signaling, cellular response to tumor necrosis factor, cellular response to interleukin-1, and drug metabolic process; CC mainly included integral component of membrane, extracellular space, integral component of plasma membrane, and plasma membrane; and MF mainly included calcium ion binding, carbohydrate, polysaccharide, and CCR chemokine receptor binding, and steroid hydroxylase activity. All GO analysis results are shown in Figure 3. Additionally, in terms of KEGG pathway analysis, the smoking group included unique pathways like neuroactive ligand-receptor interaction, serotonergic synapse, and cocaine addiction. However, the important cancer pathways in the nonsmoking group were enriched in the following pathways: retinol, ether lipid, and linoleic acid metabolism, cytokine-cytokine receptor interaction, steroid hormone biosynthesis, and thyroid hormone synthesis. The specific results of KEGG analysis are shown in Figure 4.

### 3.3. Construction of ceRNA Network in LUSC

**Figure 5:**
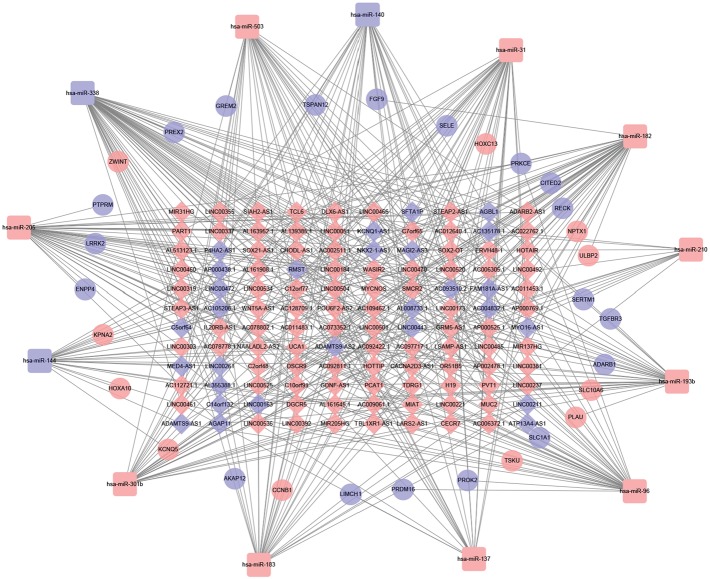
ceRNA networks of smoking LUSC. Red represents upregulation, and blue represents downregulation. lncRNAs, miRNAs, and mRNAs in the networks are represented as diamonds, round rectangles, and circles, respectively.

**Figure 6:**
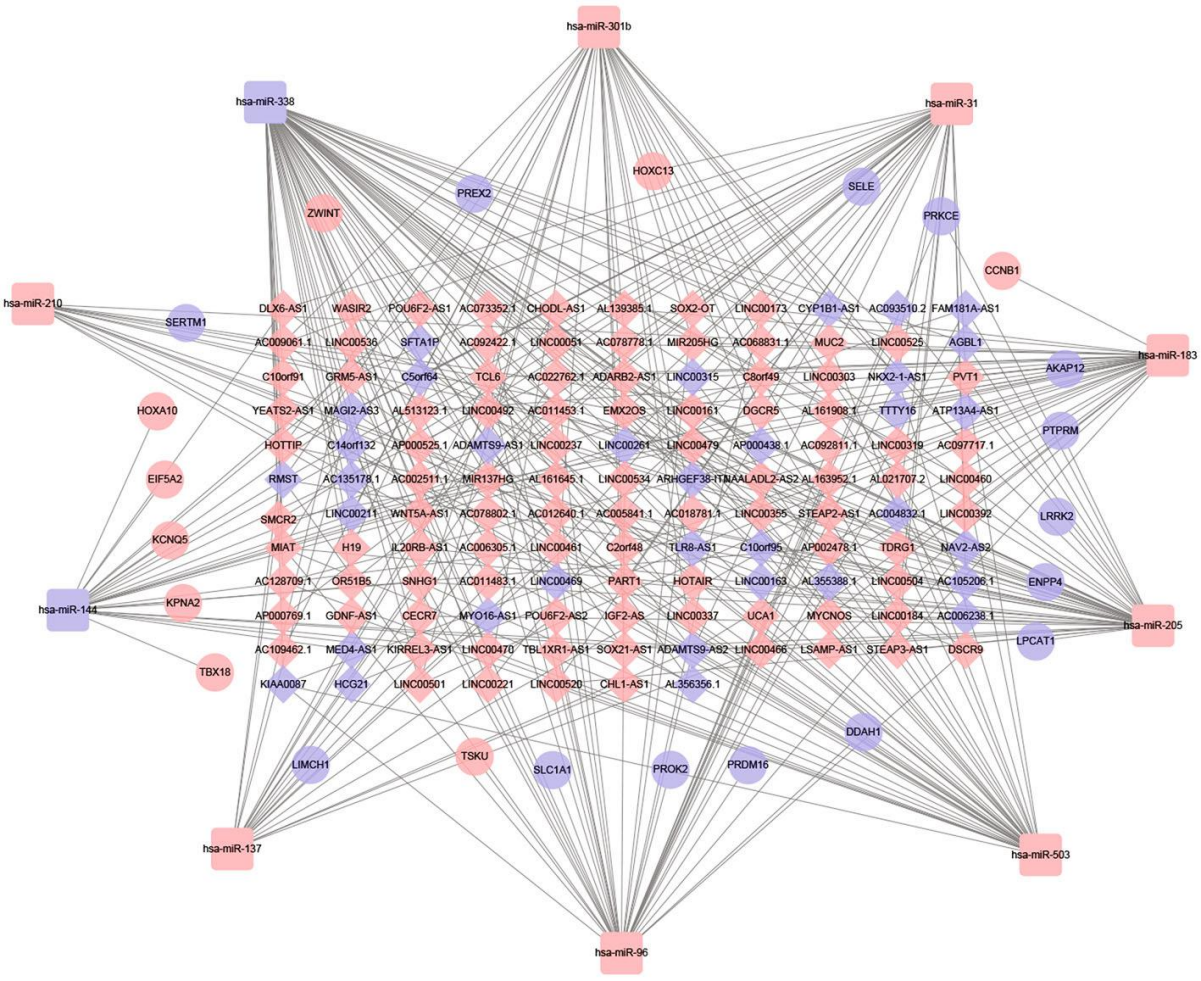
ceRNA networks of nonsmoking LUSC. Red represents upregulation, and blue represents downregulation. lncRNAs, miRNAs, and mRNAs in the networks are represented as diamonds, round rectangles, and circles, respectively.

The ceRNA network graph was constructed based on the lncRNA-miRNA pairs and miRNA-mRNA pairs and visualized using Cytoscape v3.6.1 (Figures 5 and 6). There were 131 common RNAs (102 lncRNAs, 10 miRNAs, and 19 mRNAs) in the ceRNA network. The smoking group consisted of 120 lncRNAs, 13 miRNAs, and 30 mRNAs, a total of 163 nodes and 433 edges, and the nonsmoking group consisted of 128 lncRNAs, 10 miRNAs, and 23 mRNAs, a total of 161 nodes and 348 edges. Node connections in the network can reflect the interaction between RNAs, and the stronger the connectivity, the more the importance of biological functions of this RNA in the network. Therefore, hub RNAs were defined as having a degree ≥ 5 by using topology analysis, and these hub RNAs were used for further analysis. The top three hub RNAs were hsa-miR-338, hsa-miR-182, and hsa-miR-205 in the smoking group and hsa-miR-338, hsa-miR-205, and hsa-miR-96 in the nonsmoking group.

### 3.4. Survival Analysis of Smoking- and Nonsmoking-Related RNAs in LUSC

**Figure 7:**
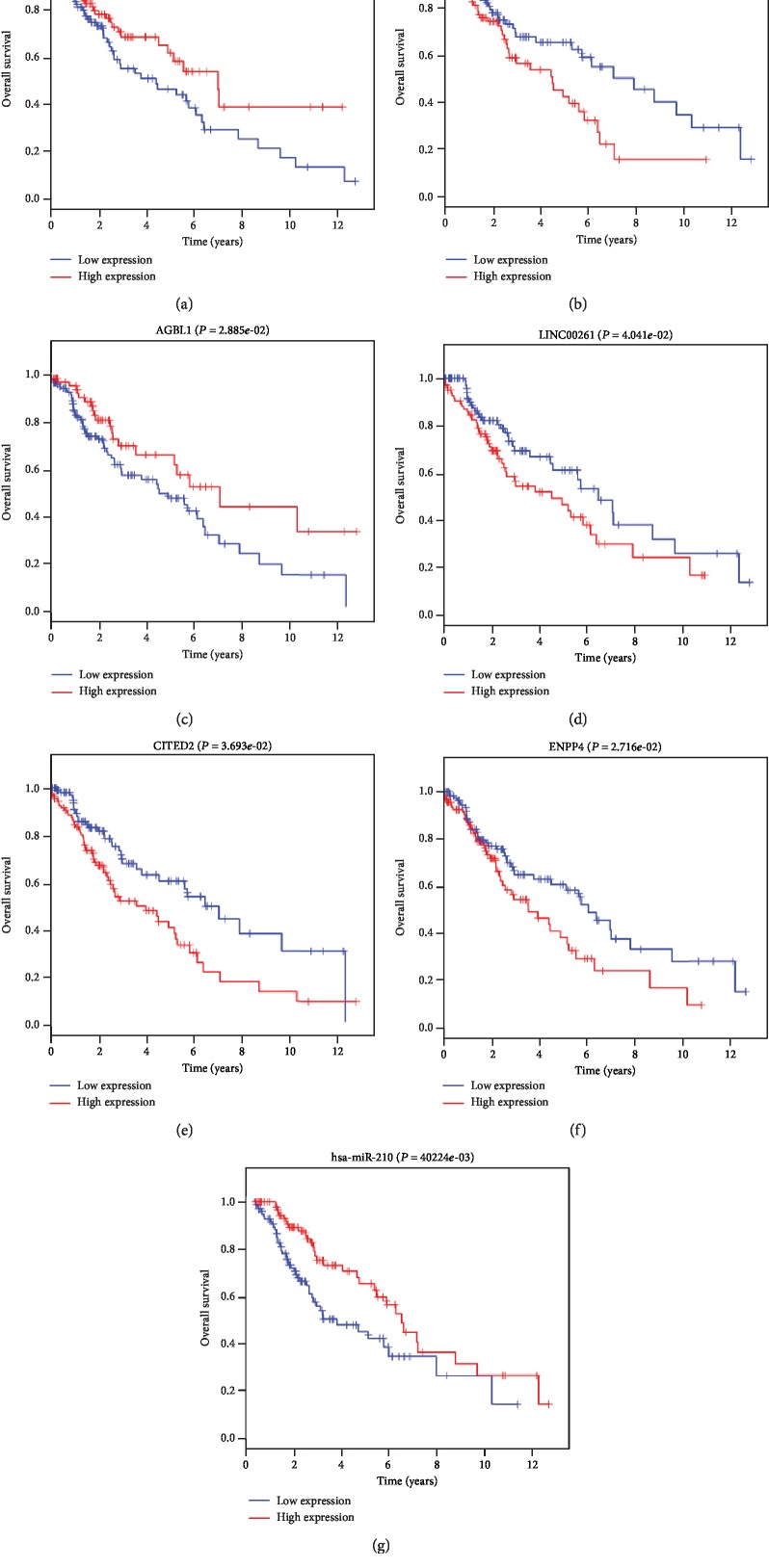
Overall survival analysis of RNAs in the ceRNA network of smoking LUSC; *P* < 0.05. (a–d) DElncRNAs; (e, f) DEmRNAs; (g) DEmiRNA. Horizontal axis is OS time (years) and vertical axis stands for survival function.

After topological analysis of the ceRNA network, we performed a survival analysis on the selected hub RNAs. As shown in Figure 7, two DElncRNAs of high expression levels (LINC00466, DLX6-AS1), two DElncRNAs of low expression levels (LINC00261, AGBL1), two DEmRNAs of low expression levels (CITED2, ENPP4), and one upregulated DEmiRNA (hsa-miR-210) were significantly related to the survival of LUSC in the smoking group (*P* < 0.05). ceRNA theory argues that lncRNA can competitively bind to miRNA and regulate its downstream target genes; based on this hypothesis, we inferred that LINC00261 and AGBL13 may serve as ceRNAs of CITED2 and all of them were predicted to interact with hsa-miR-182. LINC00466 and AGBL1 may serve as ceRNAs of ENPP4, and all of them were predicted to interact with hsa-miR-205. In the nonsmoking group, we only found one DEmRNA (PRDM16) and four DElncRNAs (AC002511.1, MAGI2-AS3, MYO16-AS1, and UCA1) that are closely related to the survival of LUSC with a *P* value < 0.05. Unfortunately, we did not find a ceRNA regulatory axis associated with the nonsmoking groups. In addition, compared to the nonsmoking group, all seven prognostic-related RNAs in the smoking group were unique.

## 4. Discussion

Lung cancer is the leading cause of cancer-related deaths worldwide, and the overall five-year survival rate is still not more than 15% [37]. Lung squamous cell carcinoma (LUSC) is the second most common type of lung cancer with a wide range of genomic features, and its occurrence is closely related to smoking [38–40]. Therefore, understanding the effect of smoking is critical to the intrinsic genetic susceptibility of LUSC. High-throughput sequencing and bioinformatics can help us screen the entire DNA mutation profile in order to understand the origins and characteristics of LUSC. The ceRNA hypothesis provides a deeper understanding of the mechanism of tumorigenesis and provides important new clues and new guiding theories to direct the research of tumor diagnosis and treatment. However, many of the specific genetic alterations, especially associated with the mechanisms of smoking-induced pathogenesis in LUSC, remain unknown. Hence, looking for available biomarkers and finding better therapeutic targets for smoking-related LUSC are essential for improving its prognosis in patients.

In our study, we divided the LUSC data obtained from TCGA into the smoking and nonsmoking groups. Through differential expression analysis, a total of 1696 DElncRNAs, 125 DEmiRNAs, and 3246 DEmRNAs in the smoking group and 1784 DElncRNAs, 96 DEmiRNAs, and 3229 DEmRNAs in the nonsmoking group were identified. The results showed that the smoking group had stronger heterogeneity than the nonsmoking group. The functional enrichment analysis consisted 423 unique DEmRNAs in the smoking group and 406 unique DEmRNAs in the nonsmoking group. The DEmRNA-related GO analysis revealed that smoking could promote cell differentiation, phagocytosis, recognition, immune response, and antigen binding. Compared to the nonsmoking group, the pathway analysis demonstrated that there were four unique pathways in the smoking group, neuroactive ligand-receptor interaction, alcoholism, serotonergic synapse, and cocaine addiction. Therefore, our enrichment results might suggest that smoking plays an important role in LUSC via those pathways. Studies have shown that smokers have increased hypothalamic activity after intravenous injections of nicotine and characteristics of the hypothalamus associated with drug addiction [41, 42]. Importantly, the extent of hypothalamic activation was significantly correlated with cocaine addiction severity [43], suggesting that cocaine addiction is an important pathway in smoking LUSC patients. Furthermore, neuroactive ligand-receptor interaction pathway *in vivo* could induce the dysregulation of miRNAs [44]. These studies indicate that smoking could directly or indirectly affect LUSC.

Studies indicate that lncRNA has important biological functions in regulating gene expression at different levels such as epigenetic levels, transcriptional regulation, and posttranscriptional regulation [12, 45]. Abnormally expressed lncRNAs can play an important role in tumor suppressor or oncogenes, in tumorigenesis and development of lung cancer, breast cancer, and prostate cancer [46–48]. lncRNA can regulate mRNA expression as ceRNA. According to the ceRNA network and overall survival analysis, a total of 4 lncRNAs and 2 mRNAs were identified as biomarkers of the smoking-related LUSC. LINC00466, AGBL1, CITED2, and ENPP4 were negatively related to overall survival, and LINC00261 and DLX6-AS1were positively related to overall survival. Interestingly, our analysis confirmed that LINC00261 and AGBL1 may serve as hsa-miR-182. LINC00466 and AGBL13 may serve as hsa-miR-205 sponges to modulate CITED2 ENPP4. So far, LINC00261, LINC00466, and AGBL13 had not been reported in any of the related studies. However, previous studies have shown that DLX6-AS1 was highly expressed in gastric cancer and lung adenocarcinoma [49, 50], which is consistent with our findings. In our study, we found that DLX6-AS1 had a good prognosis in LUSC and was significantly associated with smoking. Additionally, we also found that the two oncogenes CITED2 and ENPP4 were highly related to the prognosis of smoking-related LUSC. Numerous studies have shown that CITED2 can promote cancer metastasis, cell proliferation, and apoptosis and participate in the regulation of various transcriptional responses [51–53]. ENPP4 is a BCG-activated tumoricidal macrophage protein that can indirectly or directly contact receptors such as ATP receptors or insulin receptors on the surface of tumor cells and also destroy the release of tumor cells [54, 55]. Finally, we believe that our study is the first to find that CITED2 and ENPP4 could be prognostic biomarkers for smoking-related LUSC.

ceRNAs can competitively bind to microRNA response elements (MREs), revealing that miRNAs are at the center of ceRNA networks. It has been reported that dysregulated miRNAs play various roles in initiation, progression, invasiveness, and metastasis of tumors [56]. In the smoking ceRNA network, we found that hsa-miR-338, hsa-miR-182, and hsa-miR-205 were the top three RNAs with wide range of connections. More importantly, comparative analysis of the two groups of ceRNA network showed only three unique DEmiRNAs (hsa-miR-140, hsa-miR-193b, and hsa-miR-182) in the smoking group. Considering its unique role in the smoking ceRNA network, we conclude that the upregulated miRNA hsa-miR-182 plays an important role in smoking-related LUSC. Studies have confirmed that miR182 is highly expressed in melanoma, colon cancer, and lung cancer and can promote cell migration and invasion by inhibiting FOXO3 and MITF [57–59]. Moreover, survival analysis demonstrated that upregulated hsa-miR-210 could prolong patient survival time. Upregulation of miR-210 induced by a hypoxic microenvironment has been found to be a biomarker for many cancers such as breast cancer, clear cell renal cell carcinoma, and non-small-cell lung cancer [60–62]. Our study revealed the difference between smoking and nonsmoking ceRNA and identified specific prognostic biomarkers for smoking-related LUSC.

TCGA is a large-scale sequence database, which can be used to perform comprehensive multidimensional analysis. We constructed a ceRNA network using a large amount of data in TCGA; however, the interactions in this network are complex. Therefore, further research is needed to confirm the current findings.

## 5. Conclusion

In conclusion, we have identified the differences in the ceRNA regulatory networks between smoking and nonsmoking LUSC. Seven specific ceRNAs (LINC00466, DLX6-AS1, LINC00261, AGBL1, CITED2, ENPP4, and hsa-miR-210) associated with smoking-related LUSC prognosis that could be potential biomarkers for smoking-related LUSC diagnosis and prognosis were identified. Our research will contribute in further understanding the pathogenesis of LUSC and lay the foundation for future clinical research.

## Data Availability

The data used to support the findings of this study are included within the article.

## Supplementary Materials

Supplementary Materials Supplementary Tables 1–3: differentially expressed lncRNAs, miRNAs, and mRNAs in smoking LUSC. Supplementary Tables 4–6: differentially expressed lncRNAs, miRNAs, and mRNAs in nonsmoking LUSC. FC: fold change; FDR: false discovery rate; LUSC: lung squamous cell carcinoma.
