# Supplementary material for: Competitive Endogenous RNA Network Construction and Comparison of Lung Squamous Cell Carcinoma in Smokers and Nonsmokers
Source: Dis Markers. 2019 Dec 4;2019:5292787. doi: 10.1155/2019/5292787 (PMC6914966; doi:10.1155/2019/5292787)
Supplement: Supplementary Materials — Supplementary Tables 1–3: differentially expressed lncRNAs, miRNAs, and mRNAs in smoking LUSC. Supplementary Tables 4–6: differentially expressed lncRNAs, miRNAs, and mRNAs in nonsmoking LUSC. FC: fold change; FDR: false discovery rate; LUSC: lung squamous cell carcinoma. [file 5292787.f1.docx]

**Supplementary table 1: Differentially expressed lncRNAs in smoking LUSC**

| **lncRNA** | **logFC** | **p-value** | **FDR** | **regulate** |
| --- | --- | --- | --- | --- |
| AL136982.1 | -2.15165 | 4.54E-21 | 1.14E-19 | Down |
| MIR1-1HG-AS1 | -2.073775 | 2.33E-11 | 1.93E-10 | Down |
| FO393415.1 | -2.498204 | 1.15E-14 | 1.47E-13 | Down |
| LINC00982 | -2.559461 | 5.15E-21 | 1.28E-19 | Down |
| AC011944.1 | -2.620113 | 7.19E-24 | 2.33E-22 | Down |
| AC105206.1 | -4.309542 | 1.33E-50 | 2.34E-48 | Down |
| C5orf64 | -2.954917 | 4.91E-37 | 3.89E-35 | Down |
| AC004832.1 | -2.592031 | 8.86E-11 | 6.62E-10 | Down |
| UMODL1-AS1 | -2.959806 | 7.44E-14 | 8.46E-13 | Down |
| AL354714.1 | -3.708023 | 8.64E-32 | 4.92E-30 | Down |
| ACOXL-AS1 | -2.795202 | 1.58E-50 | 2.68E-48 | Down |
| MAMDC2-AS1 | -2.149941 | 1.15E-36 | 8.87E-35 | Down |
| AC092384.1 | -2.346282 | 1.66E-25 | 5.77E-24 | Down |
| AC134312.1 | -3.154558 | 5.91E-37 | 4.64E-35 | Down |
| AC007608.1 | -2.361798 | 2.43E-19 | 5.22E-18 | Down |
| AC007277.1 | -2.580126 | 2.97E-11 | 2.39E-10 | Down |
| AC116407.1 | -3.953695 | 6.16E-82 | 3.56E-79 | Down |
| AL772337.1 | -2.204048 | 0.000132979 | 0.000349465 | Down |
| LMO7DN-IT1 | -2.793511 | 2.27E-29 | 1.10E-27 | Down |
| LINC02471 | -3.045238 | 1.66E-15 | 2.33E-14 | Down |
| AL133264.2 | -2.029682 | 2.06E-15 | 2.85E-14 | Down |
| LINC00570 | -2.02792 | 3.76E-10 | 2.55E-09 | Down |
| AL606469.1 | -5.453718 | 2.69E-118 | 7.23E-115 | Down |
| AL031599.1 | -2.190359 | 5.18E-14 | 5.99E-13 | Down |
| AL355388.1 | -4.424251 | 7.87E-59 | 2.12E-56 | Down |
| SMIM25 | -3.046014 | 2.98E-62 | 8.61E-60 | Down |
| AC007182.1 | -2.37768 | 3.39E-13 | 3.59E-12 | Down |
| AC083949.1 | -2.012664 | 9.66E-18 | 1.75E-16 | Down |
| AL353150.1 | -2.120556 | 3.74E-27 | 1.51E-25 | Down |
| PGM5-AS1 | -3.801925 | 5.03E-33 | 3.08E-31 | Down |
| LINC01645 | -4.247824 | 8.44E-79 | 4.01E-76 | Down |
| AL591178.1 | -2.959535 | 3.78E-16 | 5.75E-15 | Down |
| LHFPL3-AS2 | -4.434648 | 5.08E-55 | 1.08E-52 | Down |
| AC079630.1 | -3.751611 | 4.09E-49 | 6.35E-47 | Down |
| AC078942.1 | -2.27137 | 3.12E-11 | 2.50E-10 | Down |
| SFTA1P | -4.358032 | 1.89E-56 | 4.37E-54 | Down |
| ATP13A4-AS1 | -2.105143 | 2.46E-05 | 7.41E-05 | Down |
| CADM3-AS1 | -3.251756 | 9.09E-28 | 3.85E-26 | Down |
| PACRG-AS3 | -4.489458 | 1.32E-42 | 1.37E-40 | Down |
| AC004540.2 | -2.282235 | 2.19E-14 | 2.68E-13 | Down |
| LINC00694 | -3.144239 | 1.66E-36 | 1.26E-34 | Down |
| AL731557.1 | -3.052311 | 1.00E-35 | 7.22E-34 | Down |
| AL109741.1 | -2.9201 | 1.16E-45 | 1.51E-43 | Down |
| AL135960.1 | -3.471479 | 7.11E-49 | 1.08E-46 | Down |
| AL008733.1 | -2.243214 | 3.73E-07 | 1.61E-06 | Down |
| LINC01811 | -2.587133 | 7.25E-12 | 6.42E-11 | Down |
| AL365214.2 | -2.007151 | 1.81E-20 | 4.27E-19 | Down |
| LINC01108 | -3.13911 | 8.91E-22 | 2.38E-20 | Down |
| BX248123.1 | -2.557664 | 5.17E-24 | 1.68E-22 | Down |
| LINC00840 | -2.369252 | 1.41E-10 | 1.02E-09 | Down |
| AL390036.1 | -2.836143 | 5.64E-35 | 3.90E-33 | Down |
| AC004112.1 | -2.115811 | 2.37E-26 | 8.63E-25 | Down |
| LHFPL3-AS1 | -2.889481 | 3.98E-14 | 4.72E-13 | Down |
| AC135178.1 | -3.138057 | 7.98E-24 | 2.56E-22 | Down |
| AL451069.1 | -2.010645 | 6.30E-07 | 2.61E-06 | Down |
| C14orf132 | -2.354045 | 3.91E-29 | 1.86E-27 | Down |
| LINC01985 | -3.206116 | 1.03E-21 | 2.75E-20 | Down |
| AC010998.1 | -3.840369 | 1.07E-35 | 7.67E-34 | Down |
| LINC01624 | -2.467965 | 1.64E-22 | 4.75E-21 | Down |
| AC018647.1 | -3.63425 | 4.14E-58 | 1.01E-55 | Down |
| AL713965.1 | -2.020145 | 8.13E-07 | 3.30E-06 | Down |
| TARID | -3.133304 | 8.19E-31 | 4.38E-29 | Down |
| AC003991.1 | -3.544525 | 5.93E-44 | 6.75E-42 | Down |
| AL590226.1 | -4.463999 | 2.00E-97 | 2.31E-94 | Down |
| AL033519.3 | -3.293243 | 1.30E-23 | 4.12E-22 | Down |
| AL445307.1 | -3.988795 | 4.26E-89 | 3.44E-86 | Down |
| SRGAP3-AS2 | -3.106816 | 1.13E-11 | 9.70E-11 | Down |
| LINC01165 | -2.470023 | 6.20E-19 | 1.28E-17 | Down |
| LINC02587 | -2.013044 | 5.75E-11 | 4.44E-10 | Down |
| MED4-AS1 | -2.321009 | 5.57E-34 | 3.66E-32 | Down |
| LINC02038 | -3.143114 | 3.25E-31 | 1.76E-29 | Down |
| AL662860.1 | -2.494494 | 5.05E-15 | 6.70E-14 | Down |
| KCNQ1-AS1 | -2.049274 | 2.49E-15 | 3.41E-14 | Down |
| AC105053.1 | -3.458007 | 1.81E-31 | 1.00E-29 | Down |
| LINC00443 | -2.022531 | 9.76E-11 | 7.22E-10 | Down |
| LINC01747 | -2.057494 | 3.37E-15 | 4.56E-14 | Down |
| AL078645.1 | -2.399594 | 5.46E-21 | 1.35E-19 | Down |
| AL035409.1 | -2.707708 | 2.15E-38 | 1.83E-36 | Down |
| AL353152.1 | -2.998792 | 1.96E-14 | 2.42E-13 | Down |
| AL391807.1 | -2.106398 | 6.41E-11 | 4.92E-10 | Down |
| HHATL-AS1 | -2.975894 | 7.62E-16 | 1.12E-14 | Down |
| AC013275.1 | -2.716544 | 4.95E-19 | 1.03E-17 | Down |
| AC096637.2 | -2.412805 | 2.20E-14 | 2.69E-13 | Down |
| AC002451.1 | -2.385507 | 3.05E-14 | 3.67E-13 | Down |
| AL445426.1 | -2.239526 | 1.72E-21 | 4.45E-20 | Down |
| AC226101.1 | -2.868819 | 1.70E-20 | 4.02E-19 | Down |
| AC013264.1 | -2.519413 | 1.10E-12 | 1.08E-11 | Down |
| AL592114.3 | -2.307698 | 1.41E-09 | 8.81E-09 | Down |
| AP002856.2 | -5.971281 | 1.09E-45 | 1.44E-43 | Down |
| AL353747.3 | -2.672056 | 2.47E-07 | 1.10E-06 | Down |
| AC092652.1 | -2.334475 | 3.89E-07 | 1.67E-06 | Down |
| PGM5P4-AS1 | -3.412184 | 9.33E-39 | 8.19E-37 | Down |
| AC011899.1 | -2.723477 | 2.45E-09 | 1.48E-08 | Down |
| LINC01105 | -3.784725 | 1.48E-22 | 4.29E-21 | Down |
| LINC01031 | -2.485007 | 2.05E-18 | 4.02E-17 | Down |
| AL354714.3 | -4.186897 | 2.21E-40 | 2.07E-38 | Down |
| AC084030.1 | -2.075175 | 3.86E-09 | 2.27E-08 | Down |
| VIPR1-AS1 | -2.815591 | 4.80E-44 | 5.54E-42 | Down |
| ELN-AS1 | -2.364515 | 3.81E-13 | 4.01E-12 | Down |
| LINC01412 | -2.816883 | 1.58E-25 | 5.53E-24 | Down |
| AL355304.1 | -2.120027 | 5.05E-22 | 1.38E-20 | Down |
| AC092809.2 | -2.6938 | 4.54E-18 | 8.42E-17 | Down |
| COL4A2-AS1 | -2.042539 | 1.22E-18 | 2.44E-17 | Down |
| LINC01166 | -2.58778 | 1.74E-08 | 9.29E-08 | Down |
| AL157786.1 | -2.06643 | 3.36E-22 | 9.38E-21 | Down |
| AC011899.2 | -3.052604 | 1.08E-50 | 1.93E-48 | Down |
| LINC00892 | -2.16069 | 8.29E-15 | 1.07E-13 | Down |
| AC093797.1 | -2.028493 | 1.25E-15 | 1.80E-14 | Down |
| LINC00702 | -3.106489 | 2.00E-44 | 2.41E-42 | Down |
| LINC00472 | -2.541988 | 1.93E-35 | 1.36E-33 | Down |
| AC007743.1 | -2.229976 | 1.11E-17 | 1.99E-16 | Down |
| LINC01765 | -3.01538 | 2.35E-11 | 1.94E-10 | Down |
| LINC00656 | -3.929535 | 5.16E-63 | 1.60E-60 | Down |
| AC004947.1 | -3.87054 | 1.93E-28 | 8.61E-27 | Down |
| AL157895.1 | -2.555457 | 8.67E-28 | 3.69E-26 | Down |
| AC243772.3 | -2.413441 | 2.60E-24 | 8.65E-23 | Down |
| LANCL1-AS1 | -4.274553 | 1.19E-134 | 4.79E-131 | Down |
| AC007405.1 | -2.944926 | 8.13E-37 | 6.32E-35 | Down |
| MAGI2-AS3 | -2.279089 | 2.41E-36 | 1.80E-34 | Down |
| LINC01506 | -2.148259 | 1.30E-12 | 1.26E-11 | Down |
| AL445470.1 | -2.943753 | 7.74E-22 | 2.08E-20 | Down |
| LINC00163 | -3.712779 | 6.50E-27 | 2.55E-25 | Down |
| LINC01827 | -3.005548 | 8.56E-15 | 1.10E-13 | Down |
| LINC00940 | -2.836647 | 2.25E-15 | 3.10E-14 | Down |
| SPAAR | -3.30166 | 5.46E-79 | 2.76E-76 | Down |
| AC006159.1 | -3.249874 | 8.53E-27 | 3.31E-25 | Down |
| AC008268.1 | -6.619769 | 7.04E-57 | 1.67E-54 | Down |
| LINC00607 | -3.219429 | 2.43E-58 | 6.13E-56 | Down |
| APOA1-AS | -2.070431 | 3.02E-32 | 1.77E-30 | Down |
| LINC01936 | -3.617296 | 1.51E-62 | 4.52E-60 | Down |
| Z82246.1 | -3.325581 | 4.89E-21 | 1.22E-19 | Down |
| LINC01447 | -2.832203 | 1.56E-15 | 2.21E-14 | Down |
| AC097059.2 | -2.10147 | 1.46E-08 | 7.89E-08 | Down |
| AL161618.1 | -2.805085 | 1.04E-09 | 6.63E-09 | Down |
| MYO16-AS1 | -2.994564 | 9.77E-19 | 1.98E-17 | Down |
| AP001189.1 | -3.930954 | 5.80E-75 | 2.47E-72 | Down |
| AC091806.1 | -2.347227 | 2.48E-22 | 7.03E-21 | Down |
| AC123023.1 | -2.790921 | 2.23E-12 | 2.12E-11 | Down |
| AC007278.2 | -2.272147 | 1.01E-12 | 1.00E-11 | Down |
| STARD13-AS | -2.553966 | 4.45E-26 | 1.58E-24 | Down |
| SMAD9-IT1 | -2.331253 | 3.36E-24 | 1.10E-22 | Down |
| LINC01844 | -2.460859 | 5.18E-17 | 8.65E-16 | Down |
| AL138900.2 | -2.145568 | 6.28E-06 | 2.13E-05 | Down |
| LINC01732 | -2.152305 | 1.35E-09 | 8.48E-09 | Down |
| AL445489.1 | -2.308917 | 2.29E-08 | 1.20E-07 | Down |
| AC096531.2 | -3.929 | 4.90E-33 | 3.02E-31 | Down |
| AL138828.1 | -2.521967 | 1.09E-15 | 1.58E-14 | Down |
| P4HA2-AS1 | -2.022469 | 1.44E-13 | 1.60E-12 | Down |
| LINC00211 | -2.481838 | 3.72E-14 | 4.42E-13 | Down |
| AC002066.1 | -2.900956 | 7.41E-34 | 4.79E-32 | Down |
| AC093110.1 | -4.034857 | 5.72E-151 | 4.63E-147 | Down |
| ZEB2-AS1 | -2.001606 | 8.73E-31 | 4.61E-29 | Down |
| LINC01352 | -2.75354 | 7.96E-34 | 5.10E-32 | Down |
| LINC01625 | -2.494634 | 5.50E-18 | 1.01E-16 | Down |
| AC116366.2 | -2.104515 | 8.84E-22 | 2.37E-20 | Down |
| AC092691.1 | -4.254619 | 5.35E-34 | 3.54E-32 | Down |
| LINC01168 | -2.547624 | 1.88E-09 | 1.16E-08 | Down |
| AC092040.1 | -2.022879 | 4.86E-15 | 6.47E-14 | Down |
| ADAMTS9-AS1 | -4.082462 | 1.76E-66 | 5.68E-64 | Down |
| ADAMTS9-AS2 | -3.570005 | 6.20E-91 | 5.56E-88 | Down |
| AL158847.1 | -2.942878 | 7.50E-23 | 2.25E-21 | Down |
| AC119424.1 | -3.758394 | 2.54E-44 | 3.02E-42 | Down |
| PARAL1 | -3.816618 | 5.15E-25 | 1.76E-23 | Down |
| AL512328.1 | -2.481344 | 5.18E-29 | 2.45E-27 | Down |
| LINC02016 | -6.223463 | 8.78E-59 | 2.29E-56 | Down |
| AC087521.1 | -3.103352 | 4.60E-37 | 3.68E-35 | Down |
| LINC01513 | -2.351973 | 7.80E-10 | 5.07E-09 | Down |
| LINC00968 | -4.407381 | 9.92E-93 | 1.00E-89 | Down |
| AC091948.1 | -2.166234 | 8.41E-41 | 8.09E-39 | Down |
| MEF2C-AS1 | -2.097982 | 8.23E-20 | 1.86E-18 | Down |
| AL645924.1 | -2.510031 | 1.34E-10 | 9.78E-10 | Down |
| LINC01197 | -2.629429 | 3.01E-38 | 2.54E-36 | Down |
| AC093772.1 | -3.345359 | 9.58E-35 | 6.50E-33 | Down |
| AC095050.1 | -4.738731 | 7.60E-35 | 5.20E-33 | Down |
| C8orf34-AS1 | -2.589215 | 4.49E-13 | 4.68E-12 | Down |
| HHIP-AS1 | -2.666373 | 1.64E-26 | 6.11E-25 | Down |
| LINC01093 | -2.408455 | 3.40E-11 | 2.72E-10 | Down |
| AC114291.1 | -3.712796 | 8.84E-17 | 1.44E-15 | Down |
| AC105389.2 | -2.601648 | 2.55E-23 | 7.93E-22 | Down |
| LINC02265 | -2.311326 | 6.22E-14 | 7.15E-13 | Down |
| AC112206.2 | -3.969886 | 3.62E-103 | 5.85E-100 | Down |
| AC010255.2 | -2.277776 | 1.35E-11 | 1.14E-10 | Down |
| AC113349.1 | -3.005056 | 5.67E-10 | 3.74E-09 | Down |
| AC105384.1 | -2.504571 | 7.71E-22 | 2.08E-20 | Down |
| LINC02147 | -2.709776 | 5.86E-25 | 1.98E-23 | Down |
| AC007663.2 | -2.445688 | 2.78E-17 | 4.82E-16 | Down |
| AP000866.2 | -3.044155 | 5.63E-70 | 1.98E-67 | Down |
| LINC01612 | -2.539312 | 2.14E-10 | 1.52E-09 | Down |
| AC112722.1 | -3.19515 | 1.59E-50 | 2.68E-48 | Down |
| AL049544.1 | -4.473493 | 4.53E-51 | 8.31E-49 | Down |
| AC109361.2 | -2.482068 | 2.08E-22 | 5.95E-21 | Down |
| AC079467.1 | -3.886072 | 6.81E-23 | 2.05E-21 | Down |
| AC037459.2 | -2.173722 | 2.47E-53 | 4.98E-51 | Down |
| F11-AS1 | -2.714948 | 3.22E-12 | 3.02E-11 | Down |
| MIR3945HG | -4.58579 | 7.42E-99 | 9.99E-96 | Down |
| LINC02435 | -2.422506 | 2.75E-19 | 5.89E-18 | Down |
| AC096751.2 | -2.108773 | 2.35E-10 | 1.65E-09 | Down |
| AC139713.2 | -2.072316 | 2.01E-13 | 2.19E-12 | Down |
| AC104211.2 | -3.865752 | 9.61E-50 | 1.52E-47 | Down |
| AC046195.1 | -3.656003 | 7.55E-23 | 2.26E-21 | Down |
| NKX2-1-AS1 | -2.316615 | 8.65E-10 | 5.59E-09 | Down |
| AC090796.1 | -3.805763 | 1.83E-25 | 6.35E-24 | Down |
| LINC01863 | -4.023397 | 1.09E-20 | 2.61E-19 | Down |
| AC021546.1 | -2.285877 | 2.62E-14 | 3.16E-13 | Down |
| RBPMS-AS1 | -2.608178 | 1.48E-33 | 9.45E-32 | Down |
| AC011365.2 | -2.54291 | 2.36E-23 | 7.38E-22 | Down |
| AC012349.1 | -2.551351 | 9.06E-13 | 9.11E-12 | Down |
| AP001972.1 | -2.820577 | 5.86E-23 | 1.78E-21 | Down |
| AP001189.3 | -3.074717 | 1.84E-45 | 2.32E-43 | Down |
| AC009806.1 | -2.735193 | 3.06E-36 | 2.27E-34 | Down |
| LINC02489 | -3.580217 | 3.31E-22 | 9.32E-21 | Down |
| AP000842.2 | -2.071282 | 6.45E-07 | 2.66E-06 | Down |
| AC009652.1 | -2.236526 | 2.88E-10 | 1.98E-09 | Down |
| AC090559.1 | -2.318212 | 2.86E-31 | 1.56E-29 | Down |
| KC877392.1 | -2.138044 | 1.07E-12 | 1.06E-11 | Down |
| AP001189.5 | -2.75162 | 2.85E-26 | 1.02E-24 | Down |
| TBX5-AS1 | -2.697145 | 2.11E-43 | 2.27E-41 | Down |
| AP002954.1 | -2.208419 | 6.99E-18 | 1.28E-16 | Down |
| AP003064.2 | -2.62558 | 3.41E-17 | 5.83E-16 | Down |
| AP001528.2 | -2.652945 | 1.76E-48 | 2.59E-46 | Down |
| AP001189.6 | -2.399295 | 2.35E-18 | 4.54E-17 | Down |
| AP000439.1 | -2.105482 | 9.00E-09 | 5.01E-08 | Down |
| RMST | -3.478746 | 1.98E-17 | 3.46E-16 | Down |
| AC010175.1 | -2.078908 | 1.41E-16 | 2.24E-15 | Down |
| AC084880.3 | -3.141739 | 1.47E-22 | 4.29E-21 | Down |
| AC026369.3 | -4.227325 | 1.10E-61 | 3.08E-59 | Down |
| AP000438.1 | -3.381133 | 5.86E-43 | 6.15E-41 | Down |
| AP002761.3 | -2.014102 | 2.25E-26 | 8.22E-25 | Down |
| C11orf97 | -2.762038 | 4.99E-08 | 2.49E-07 | Down |
| AC084398.2 | -3.047415 | 2.11E-25 | 7.27E-24 | Down |
| AC027288.1 | -4.643396 | 7.13E-84 | 4.80E-81 | Down |
| AL132857.1 | -3.444644 | 1.26E-18 | 2.53E-17 | Down |
| AC023509.2 | -3.128991 | 7.49E-44 | 8.41E-42 | Down |
| HLX-AS1 | -2.563247 | 2.08E-21 | 5.28E-20 | Down |
| AC124312.2 | -2.134168 | 1.14E-17 | 2.05E-16 | Down |
| AC027288.3 | -4.402011 | 6.35E-117 | 1.28E-113 | Down |
| AC024257.1 | -3.516842 | 5.05E-30 | 2.62E-28 | Down |
| AC090001.1 | -2.383039 | 3.24E-09 | 1.93E-08 | Down |
| FAM181A-AS1 | -2.034179 | 3.51E-07 | 1.52E-06 | Down |
| LINC00930 | -2.092396 | 1.08E-07 | 5.05E-07 | Down |
| AC091544.2 | -2.28945 | 2.37E-10 | 1.66E-09 | Down |
| AL357093.1 | -2.882315 | 3.26E-18 | 6.12E-17 | Down |
| AL357093.2 | -2.409195 | 1.85E-09 | 1.14E-08 | Down |
| LINC02289 | -2.088523 | 3.74E-16 | 5.71E-15 | Down |
| AL157955.2 | -2.32059 | 9.42E-13 | 9.46E-12 | Down |
| AL162511.1 | -4.115325 | 4.72E-39 | 4.23E-37 | Down |
| AL049871.1 | -2.386469 | 7.07E-26 | 2.50E-24 | Down |
| AC013457.1 | -5.061923 | 8.26E-36 | 6.01E-34 | Down |
| PTCSC3 | -2.162977 | 6.93E-13 | 7.09E-12 | Down |
| LINC00924 | -2.237525 | 6.26E-20 | 1.44E-18 | Down |
| AC015914.1 | -3.17406 | 7.98E-55 | 1.65E-52 | Down |
| AC012409.1 | -2.157453 | 5.14E-12 | 4.67E-11 | Down |
| CTXND1 | -4.448827 | 1.15E-79 | 6.22E-77 | Down |
| AC026992.1 | -3.265969 | 1.40E-38 | 1.20E-36 | Down |
| AC111152.2 | -2.117855 | 2.46E-26 | 8.92E-25 | Down |
| AC106738.2 | -2.629194 | 1.23E-14 | 1.56E-13 | Down |
| AL365361.1 | -2.478703 | 6.40E-27 | 2.52E-25 | Down |
| LINC02126 | -2.275589 | 2.12E-18 | 4.14E-17 | Down |
| AC130456.1 | -2.826077 | 5.92E-21 | 1.46E-19 | Down |
| AC130456.2 | -2.986467 | 2.94E-20 | 6.83E-19 | Down |
| LINC00261 | -3.01657 | 7.65E-13 | 7.78E-12 | Down |
| LINC01571 | -2.559943 | 6.02E-07 | 2.49E-06 | Down |
| AC027277.2 | -2.841486 | 6.24E-27 | 2.47E-25 | Down |
| AC068700.1 | -2.489364 | 4.27E-26 | 1.52E-24 | Down |
| AL133355.1 | -2.392869 | 6.37E-53 | 1.23E-50 | Down |
| LINC01290 | -2.438348 | 1.73E-48 | 2.59E-46 | Down |
| AC008669.1 | -2.260448 | 5.52E-42 | 5.51E-40 | Down |
| AC022164.1 | -2.547303 | 7.54E-27 | 2.94E-25 | Down |
| AC025280.1 | -3.014186 | 4.37E-28 | 1.92E-26 | Down |
| AC093510.2 | -2.506003 | 9.96E-29 | 4.55E-27 | Down |
| LINC02555 | -3.643214 | 2.23E-26 | 8.21E-25 | Down |
| AC093278.2 | -2.425848 | 1.91E-45 | 2.37E-43 | Down |
| AC096921.2 | -2.936451 | 2.27E-56 | 5.09E-54 | Down |
| AC083837.1 | -3.465721 | 6.90E-48 | 9.62E-46 | Down |
| AC026992.2 | -3.91391 | 1.43E-52 | 2.70E-50 | Down |
| LINC00165 | -2.231188 | 5.06E-05 | 0.000143329 | Down |
| AC005592.1 | -2.10201 | 2.17E-10 | 1.54E-09 | Down |
| LINC01996 | -4.383908 | 1.04E-27 | 4.38E-26 | Down |
| LINC02185 | -3.345779 | 2.93E-35 | 2.04E-33 | Down |
| AC027601.2 | -2.030801 | 2.57E-27 | 1.05E-25 | Down |
| AC005736.1 | -2.50491 | 6.38E-22 | 1.74E-20 | Down |
| AC129507.2 | -2.828799 | 4.24E-19 | 8.84E-18 | Down |
| AC108134.3 | -2.186219 | 1.63E-27 | 6.67E-26 | Down |
| AC129507.3 | -2.628819 | 4.60E-15 | 6.13E-14 | Down |
| AC127521.1 | -2.60621 | 3.41E-33 | 2.14E-31 | Down |
| AC027281.1 | -3.2453 | 6.82E-13 | 6.99E-12 | Down |
| AC015908.2 | -2.111406 | 1.18E-12 | 1.16E-11 | Down |
| HID1-AS1 | -3.821728 | 4.76E-70 | 1.75E-67 | Down |
| AC091588.1 | -2.13426 | 1.14E-11 | 9.73E-11 | Down |
| AC104984.1 | -3.720839 | 1.72E-31 | 9.68E-30 | Down |
| LINC01908 | -2.330239 | 4.86E-08 | 2.43E-07 | Down |
| AP001094.2 | -2.477402 | 1.35E-29 | 6.79E-28 | Down |
| AC005358.2 | -2.469277 | 6.87E-16 | 1.02E-14 | Down |
| GATA6-AS1 | -3.266501 | 2.79E-55 | 6.09E-53 | Down |
| AC104984.4 | -4.765425 | 2.73E-76 | 1.22E-73 | Down |
| AP001094.3 | -2.455356 | 4.93E-12 | 4.49E-11 | Down |
| AC091588.3 | -2.773313 | 5.77E-28 | 2.48E-26 | Down |
| SLC14A2-AS1 | -2.554132 | 1.92E-19 | 4.17E-18 | Down |
| PCAT19 | -2.870679 | 4.29E-47 | 5.87E-45 | Down |
| AC005901.1 | -2.664036 | 3.56E-22 | 9.85E-21 | Down |
| AC005884.1 | -4.08722 | 3.84E-84 | 2.82E-81 | Down |
| AC020911.2 | -2.33026 | 2.21E-12 | 2.11E-11 | Down |
| AC011444.3 | -2.44467 | 3.23E-19 | 6.86E-18 | Down |
| AC002398.2 | -3.120086 | 2.64E-24 | 8.76E-23 | Down |
| AC005180.1 | -2.628544 | 1.13E-26 | 4.25E-25 | Down |
| AC003070.2 | -2.053177 | 1.77E-12 | 1.70E-11 | Down |
| AC079210.1 | -2.446397 | 6.81E-21 | 1.67E-19 | Down |
| AC005180.2 | -2.472752 | 9.16E-31 | 4.81E-29 | Down |
| LINC01836 | -2.576843 | 2.84E-28 | 1.26E-26 | Down |
| MIR497HG | -2.156872 | 5.20E-50 | 8.40E-48 | Down |
| AC011511.5 | -3.564315 | 1.74E-43 | 1.91E-41 | Down |
| AC005856.1 | -3.206556 | 1.77E-71 | 6.83E-69 | Down |
| LDLRAD4-AS1 | -3.680128 | 1.05E-45 | 1.42E-43 | Down |
| AC093567.1 | -2.491207 | 1.85E-29 | 9.04E-28 | Down |
| AC010776.2 | -3.927035 | 3.42E-22 | 9.49E-21 | Down |
| AC243960.3 | -2.195628 | 8.28E-24 | 2.64E-22 | Down |
| FENDRR | -4.120583 | 8.33E-69 | 2.81E-66 | Down |
| AC010329.1 | -2.984641 | 2.32E-18 | 4.49E-17 | Down |
| AC135012.3 | -5.046395 | 8.60E-83 | 5.35E-80 | Down |
| AC245128.3 | -2.024836 | 3.64E-13 | 3.84E-12 | Down |
| AC243967.2 | -2.21783 | 3.06E-12 | 2.88E-11 | Down |
| AC007193.2 | -2.240549 | 3.77E-11 | 2.99E-10 | Down |
| LINC01082 | -4.634843 | 1.76E-73 | 7.13E-71 | Down |
| AC092071.1 | -3.223764 | 3.56E-17 | 6.05E-16 | Down |
| AL589765.7 | -2.707666 | 5.09E-14 | 5.92E-13 | Down |
| LINC02104 | -2.822488 | 4.54E-34 | 3.03E-32 | Down |
| AL139041.1 | -2.673314 | 2.58E-45 | 3.16E-43 | Down |
| AC005740.4 | -2.069902 | 6.24E-32 | 3.60E-30 | Down |
| AGAP11 | -2.123799 | 4.05E-16 | 6.09E-15 | Down |
| AC012467.1 | -2.156674 | 1.17E-11 | 1.00E-10 | Down |
| LINC00551 | -2.781013 | 2.25E-22 | 6.40E-21 | Down |
| AC116312.1 | -2.207052 | 1.87E-13 | 2.05E-12 | Down |
| AL357054.4 | -2.48168 | 3.30E-36 | 2.42E-34 | Down |
| AC006273.1 | -2.893767 | 5.31E-48 | 7.67E-46 | Down |
| AC009974.1 | -2.144281 | 3.38E-20 | 7.82E-19 | Down |
| AC004982.1 | -2.21123 | 9.61E-30 | 4.88E-28 | Down |
| AC147067.2 | -2.922288 | 2.36E-43 | 2.51E-41 | Down |
| AC019193.2 | -3.061473 | 1.26E-45 | 1.61E-43 | Down |
| AC010976.2 | -3.579658 | 4.27E-41 | 4.15E-39 | Down |
| AL445423.1 | -2.992931 | 4.65E-42 | 4.69E-40 | Down |
| AC011899.3 | -2.689592 | 5.89E-29 | 2.75E-27 | Down |
| AC016717.2 | -2.994138 | 1.37E-22 | 4.01E-21 | Down |
| AC027449.1 | -2.275354 | 1.15E-19 | 2.57E-18 | Down |
| SFTPD-AS1 | -2.903381 | 3.25E-29 | 1.55E-27 | Down |
| AGBL1 | -3.242211 | 8.19E-19 | 1.67E-17 | Down |
| AC024337.2 | -2.396605 | 1.57E-32 | 9.37E-31 | Down |
| AC018755.4 | -2.456671 | 1.12E-26 | 4.24E-25 | Down |
| AC236972.3 | -4.985165 | 2.81E-44 | 3.29E-42 | Down |
| AL135999.3 | -3.735146 | 2.50E-25 | 8.58E-24 | Down |
| AC024909.2 | -2.135267 | 3.85E-17 | 6.52E-16 | Down |
| AC023449.2 | -2.228004 | 2.04E-19 | 4.42E-18 | Down |
| AL034397.3 | -2.262294 | 1.85E-18 | 3.63E-17 | Down |
| AC007671.1 | -2.530615 | 2.06E-24 | 6.91E-23 | Down |
| AC018529.1 | -2.94431 | 1.47E-40 | 1.39E-38 | Down |
| AC245884.11 | -2.026418 | 1.23E-14 | 1.56E-13 | Down |
| AL445493.3 | -3.594834 | 2.36E-40 | 2.19E-38 | Down |
| AC104237.2 | -2.890338 | 1.03E-18 | 2.08E-17 | Down |
| BX005040.1 | -2.238884 | 2.36E-05 | 7.15E-05 | Down |
| AL133320.1 | -2.348057 | 8.12E-07 | 3.30E-06 | Down |
| AL590491.2 | -2.321303 | 1.94E-05 | 5.96E-05 | Down |
| AC104237.3 | -2.611413 | 6.77E-20 | 1.55E-18 | Down |
| AC025271.4 | -3.666427 | 4.67E-53 | 9.21E-51 | Down |
| AC012409.3 | -2.524217 | 1.12E-23 | 3.55E-22 | Down |
| AC110048.2 | -2.045471 | 3.91E-19 | 8.19E-18 | Down |
| AL353770.4 | -2.613367 | 3.97E-07 | 1.70E-06 | Down |
| AC013553.3 | -2.461774 | 3.65E-37 | 2.95E-35 | Down |
| AC005277.2 | -2.6316 | 1.88E-26 | 6.96E-25 | Down |
| PGM5P3-AS1 | -2.419289 | 1.98E-09 | 1.21E-08 | Down |
| AC037198.2 | -2.440467 | 4.95E-21 | 1.23E-19 | Down |
| LINC02033 | -2.018487 | 5.25E-23 | 1.60E-21 | Down |
| AC091891.2 | -2.820718 | 9.02E-16 | 1.32E-14 | Down |
| LINC00891 | -3.136558 | 1.23E-43 | 1.36E-41 | Down |
| LINC01587 | 3.4706176 | 1.52E-06 | 5.84E-06 | Up |
| H19 | 3.3115108 | 3.64E-05 | 0.000106688 | Up |
| LINC00470 | 4.1512507 | 8.24E-09 | 4.60E-08 | Up |
| LINC00525 | 2.4442214 | 3.42E-07 | 1.48E-06 | Up |
| PART1 | 4.6609878 | 5.97E-11 | 4.60E-10 | Up |
| C2orf48 | 3.0149528 | 4.25E-15 | 5.67E-14 | Up |
| LINC01116 | 2.8479532 | 6.69E-10 | 4.39E-09 | Up |
| IGF2BP2-AS1 | 3.1115294 | 1.39E-08 | 7.50E-08 | Up |
| OR51B5 | 2.6374682 | 0.000846876 | 0.001891543 | Up |
| MIR31HG | 3.7170881 | 6.23E-08 | 3.06E-07 | Up |
| AP000769.1 | 2.7371527 | 3.51E-17 | 5.98E-16 | Up |
| CCDC197 | 2.7097031 | 4.93E-05 | 0.000140038 | Up |
| LINC00303 | 2.8843205 | 0.000395209 | 0.00094348 | Up |
| TYMSOS | 3.4038186 | 5.27E-27 | 2.10E-25 | Up |
| AP000679.1 | 3.5760175 | 7.61E-15 | 9.92E-14 | Up |
| LINC01561 | 3.7952231 | 4.71E-12 | 4.32E-11 | Up |
| AC020907.1 | 6.9519718 | 7.60E-26 | 2.67E-24 | Up |
| MTUS2-AS1 | 2.5114406 | 3.10E-06 | 1.12E-05 | Up |
| C10orf91 | 3.7146562 | 4.94E-16 | 7.37E-15 | Up |
| LINC01559 | 6.9678847 | 1.18E-14 | 1.51E-13 | Up |
| OGFRP1 | 2.7200557 | 1.68E-23 | 5.30E-22 | Up |
| LINC00518 | 4.8353037 | 7.66E-07 | 3.13E-06 | Up |
| B3GALT5-AS1 | 2.6871752 | 4.94E-05 | 0.000140134 | Up |
| LINC01405 | 2.866115 | 0.00050584 | 0.001179368 | Up |
| TCL6 | 2.2697044 | 0.000299998 | 0.00073219 | Up |
| AC010969.1 | 2.2372501 | 0.004634657 | 0.008869629 | Up |
| LINC00319 | 2.7539857 | 3.62E-06 | 1.29E-05 | Up |
| AC069277.1 | 5.3549272 | 7.78E-21 | 1.89E-19 | Up |
| LINC00615 | 5.6477165 | 4.16E-09 | 2.43E-08 | Up |
| C20orf204 | 2.3067725 | 3.26E-11 | 2.61E-10 | Up |
| LINC00173 | 2.265053 | 9.17E-11 | 6.82E-10 | Up |
| NPSR1-AS1 | 6.1661012 | 4.98E-20 | 1.15E-18 | Up |
| AC068631.1 | 3.5086729 | 3.20E-14 | 3.83E-13 | Up |
| AC090673.1 | 3.1171584 | 3.14E-16 | 4.84E-15 | Up |
| GATA3-AS1 | 2.9990494 | 0.000193589 | 0.000491021 | Up |
| CYYR1-AS1 | 2.0326634 | 0.000105854 | 0.000284 | Up |
| MUC2 | 6.2208683 | 4.12E-07 | 1.75E-06 | Up |
| IQANK1 | 3.1599282 | 1.62E-28 | 7.26E-27 | Up |
| LINC00501 | 5.6203078 | 1.29E-07 | 5.99E-07 | Up |
| LINC02487 | 2.4921466 | 0.000230694 | 0.000576986 | Up |
| LINC00862 | 2.4426535 | 3.10E-09 | 1.86E-08 | Up |
| SLC12A5-AS1 | 2.4614196 | 2.95E-11 | 2.38E-10 | Up |
| TDRG1 | 4.2527929 | 9.27E-07 | 3.73E-06 | Up |
| AL590644.1 | 5.549492 | 4.81E-11 | 3.76E-10 | Up |
| PKP4-AS1 | 2.6569012 | 2.56E-11 | 2.09E-10 | Up |
| LINC01854 | 7.4294283 | 8.00E-09 | 4.48E-08 | Up |
| SMPD5 | 2.8061588 | 3.75E-12 | 3.49E-11 | Up |
| ST8SIA6-AS1 | 3.4403933 | 0.000212485 | 0.000534752 | Up |
| FAM83A-AS1 | 6.4253846 | 1.15E-17 | 2.06E-16 | Up |
| LINC01602 | 4.5724893 | 0.000103186 | 0.000277856 | Up |
| AC005863.1 | 6.1366253 | 8.12E-12 | 7.14E-11 | Up |
| LINC01460 | 2.856855 | 4.64E-10 | 3.11E-09 | Up |
| CALML3-AS1 | 6.0665637 | 1.51E-27 | 6.21E-26 | Up |
| LINC01446 | 4.7059499 | 0.000110295 | 0.000294759 | Up |
| LINC00898 | 7.6604043 | 7.54E-20 | 1.71E-18 | Up |
| ADARB2-AS1 | 3.230524 | 2.05E-05 | 6.29E-05 | Up |
| AC006305.1 | 4.4462662 | 3.40E-18 | 6.37E-17 | Up |
| DUXAP8 | 3.2825984 | 1.43E-17 | 2.53E-16 | Up |
| AC117402.1 | 3.6104487 | 1.45E-10 | 1.05E-09 | Up |
| EWSAT1 | 2.9760598 | 1.42E-06 | 5.52E-06 | Up |
| FIRRE | 3.7010789 | 3.75E-19 | 7.89E-18 | Up |
| AC019080.1 | 2.1375538 | 9.96E-09 | 5.50E-08 | Up |
| UCA1 | 5.822921 | 4.82E-12 | 4.41E-11 | Up |
| LINC00887 | 4.0125315 | 6.00E-16 | 8.92E-15 | Up |
| AC087491.1 | 2.2242444 | 4.03E-05 | 0.00011699 | Up |
| CDIPTOSP | 4.1244145 | 4.33E-14 | 5.11E-13 | Up |
| AC010168.1 | 4.6893265 | 8.94E-27 | 3.44E-25 | Up |
| AC129492.1 | 2.1506227 | 1.80E-07 | 8.15E-07 | Up |
| LINC01139 | 2.6814185 | 9.48E-05 | 0.000257376 | Up |
| LINC01356 | 2.6925127 | 1.26E-06 | 4.93E-06 | Up |
| AC131097.2 | 2.8454295 | 6.32E-08 | 3.10E-07 | Up |
| AC007249.1 | 2.3766471 | 1.40E-06 | 5.44E-06 | Up |
| AC073316.1 | 4.9686504 | 1.03E-07 | 4.84E-07 | Up |
| AC011298.1 | 5.8450248 | 9.54E-09 | 5.29E-08 | Up |
| LINC02076 | 2.7405197 | 2.13E-10 | 1.51E-09 | Up |
| AC246793.1 | 4.5338084 | 9.75E-13 | 9.74E-12 | Up |
| C7orf65 | 2.3602435 | 0.000270766 | 0.000667294 | Up |
| AC112721.1 | 2.6510179 | 9.29E-08 | 4.40E-07 | Up |
| AC112721.2 | 2.9371833 | 4.23E-10 | 2.85E-09 | Up |
| AC079305.1 | 2.0718282 | 4.19E-09 | 2.44E-08 | Up |
| LINC02068 | 2.3036299 | 6.87E-07 | 2.82E-06 | Up |
| AC009988.1 | 4.7682805 | 3.51E-09 | 2.07E-08 | Up |
| LINC01615 | 3.4271853 | 4.28E-11 | 3.37E-10 | Up |
| AL391097.1 | 3.6317308 | 6.63E-07 | 2.73E-06 | Up |
| AL121990.1 | 2.0260643 | 9.82E-06 | 3.20E-05 | Up |
| LINC01208 | 5.5557572 | 4.86E-14 | 5.68E-13 | Up |
| AC116609.1 | 2.9149181 | 0.001147828 | 0.002497034 | Up |
| LINP1 | 3.6468549 | 7.19E-08 | 3.49E-07 | Up |
| AC073365.1 | 9.1652088 | 1.60E-13 | 1.77E-12 | Up |
| AC004870.2 | 3.0336659 | 0.000417239 | 0.00099197 | Up |
| AC006372.1 | 2.9515994 | 0.001150964 | 0.002502943 | Up |
| SMCR2 | 2.3981947 | 7.41E-09 | 4.16E-08 | Up |
| LINC01449 | 2.3260054 | 4.21E-05 | 0.000121801 | Up |
| LINC02561 | 3.4875071 | 9.57E-09 | 5.31E-08 | Up |
| AC091729.1 | 2.821141 | 3.10E-08 | 1.59E-07 | Up |
| MIR548XHG | 7.7333198 | 1.11E-07 | 5.20E-07 | Up |
| AL390729.1 | 2.7901784 | 3.11E-07 | 1.36E-06 | Up |
| MIR3681HG | 2.5262992 | 4.01E-05 | 0.000116318 | Up |
| AC008278.1 | 4.1486964 | 5.35E-06 | 1.84E-05 | Up |
| LINC00466 | 4.5735273 | 9.16E-11 | 6.82E-10 | Up |
| AL391427.1 | 4.0421332 | 1.56E-11 | 1.31E-10 | Up |
| LINC01133 | 3.7035318 | 7.49E-09 | 4.20E-08 | Up |
| AL023754.1 | 5.6074753 | 2.31E-12 | 2.20E-11 | Up |
| AP000697.1 | 4.4759372 | 5.46E-11 | 4.23E-10 | Up |
| AL117329.1 | 9.0570767 | 6.97E-34 | 4.54E-32 | Up |
| LINC01527 | 5.6470356 | 2.81E-10 | 1.94E-09 | Up |
| AL365226.1 | 2.4122048 | 1.63E-08 | 8.74E-08 | Up |
| AL139246.1 | 2.5373192 | 1.99E-06 | 7.51E-06 | Up |
| LINC01280 | 5.0732152 | 3.68E-10 | 2.50E-09 | Up |
| AC114489.1 | 5.3282016 | 1.32E-15 | 1.89E-14 | Up |
| AP001476.1 | 2.3290162 | 0.000229094 | 0.000573515 | Up |
| AL606970.1 | 4.1346448 | 1.24E-05 | 3.96E-05 | Up |
| AC092803.1 | 2.729814 | 3.12E-05 | 9.25E-05 | Up |
| LINC01087 | 3.3717489 | 0.00029523 | 0.000722299 | Up |
| LINC01117 | 2.6339178 | 9.70E-09 | 5.38E-08 | Up |
| AL135787.1 | 2.466994 | 3.62E-05 | 0.000106163 | Up |
| LINC00885 | 2.8080471 | 1.07E-11 | 9.22E-11 | Up |
| AL513164.1 | 3.1262227 | 6.35E-05 | 0.000177606 | Up |
| AC098936.1 | 3.0668004 | 1.58E-06 | 6.06E-06 | Up |
| AL391704.1 | 3.7604255 | 1.37E-05 | 4.35E-05 | Up |
| AC114812.2 | 5.9234323 | 1.83E-16 | 2.89E-15 | Up |
| AC010789.1 | 4.7681879 | 2.60E-06 | 9.50E-06 | Up |
| AC093843.1 | 2.6852894 | 2.53E-05 | 7.62E-05 | Up |
| AL161908.1 | 4.3357238 | 9.11E-07 | 3.67E-06 | Up |
| LINC00393 | 8.1612649 | 2.96E-13 | 3.15E-12 | Up |
| CDKN2A-DT | 2.7724444 | 5.62E-06 | 1.92E-05 | Up |
| AC019155.3 | 5.8808804 | 5.86E-13 | 6.03E-12 | Up |
| LINC00184 | 2.8953655 | 4.78E-09 | 2.76E-08 | Up |
| LINC01752 | 4.2381615 | 7.24E-15 | 9.47E-14 | Up |
| AL512363.1 | 6.973463 | 5.18E-28 | 2.25E-26 | Up |
| AL355483.1 | 3.0121783 | 1.66E-07 | 7.58E-07 | Up |
| LINC00337 | 2.5639457 | 1.20E-10 | 8.77E-10 | Up |
| AL583808.1 | 5.8822409 | 1.12E-21 | 2.97E-20 | Up |
| AC092484.1 | 4.4884685 | 9.86E-07 | 3.95E-06 | Up |
| LINC00237 | 2.5799399 | 0.001919432 | 0.003973087 | Up |
| SLC9A3-AS1 | 2.9250254 | 2.35E-13 | 2.54E-12 | Up |
| AC073957.1 | 4.0252562 | 6.23E-07 | 2.58E-06 | Up |
| AC012354.1 | 3.239648 | 3.97E-05 | 0.000115391 | Up |
| AL662890.1 | 2.0170592 | 0.000100838 | 0.000272258 | Up |
| OSTM1-AS1 | 4.2075891 | 2.30E-06 | 8.55E-06 | Up |
| AL590617.2 | 2.1062712 | 7.02E-16 | 1.04E-14 | Up |
| MIR137HG | 6.5518759 | 4.87E-09 | 2.81E-08 | Up |
| AP000527.1 | 6.3083219 | 6.66E-08 | 3.25E-07 | Up |
| LINC00705 | 2.7537181 | 7.83E-06 | 2.61E-05 | Up |
| AL354707.1 | 3.2008833 | 1.74E-15 | 2.44E-14 | Up |
| CYP4A22-AS1 | 3.0747287 | 1.56E-18 | 3.08E-17 | Up |
| LINC01703 | 3.111799 | 1.31E-21 | 3.42E-20 | Up |
| AL731684.1 | 2.9241857 | 1.82E-06 | 6.93E-06 | Up |
| LINC01393 | 2.3052177 | 3.68E-10 | 2.50E-09 | Up |
| LINC02476 | 6.6352304 | 9.41E-07 | 3.78E-06 | Up |
| LINC01980 | 9.6315558 | 5.99E-30 | 3.06E-28 | Up |
| AP000320.1 | 2.2474296 | 1.20E-06 | 4.73E-06 | Up |
| LINC01623 | 2.7068247 | 6.93E-05 | 0.000192311 | Up |
| AL163953.1 | 5.7648998 | 1.06E-08 | 5.81E-08 | Up |
| MIAT | 2.253454 | 9.90E-08 | 4.67E-07 | Up |
| AC073321.1 | 4.376067 | 1.50E-11 | 1.27E-10 | Up |
| AC092669.1 | 4.5911551 | 1.70E-09 | 1.05E-08 | Up |
| LINC00626 | 8.501667 | 2.17E-16 | 3.40E-15 | Up |
| LINC01456 | 5.7442986 | 1.18E-10 | 8.61E-10 | Up |
| SATB2-AS1 | 3.4322608 | 9.34E-12 | 8.13E-11 | Up |
| AL355482.2 | 2.2457878 | 2.01E-07 | 9.06E-07 | Up |
| AC124944.1 | 2.1028094 | 5.72E-09 | 3.26E-08 | Up |
| LEMD1-AS1 | 2.1045473 | 4.53E-06 | 1.58E-05 | Up |
| LINC00381 | 2.9725231 | 4.61E-06 | 1.60E-05 | Up |
| AL122058.1 | 3.837722 | 2.00E-11 | 1.66E-10 | Up |
| HAGLROS | 4.5244882 | 1.61E-23 | 5.10E-22 | Up |
| LINC01876 | 2.3182155 | 2.93E-10 | 2.02E-09 | Up |
| PARD3-AS1 | 2.4331528 | 1.62E-10 | 1.16E-09 | Up |
| C12orf77 | 4.3248957 | 7.18E-05 | 0.000198511 | Up |
| LINC02542 | 2.2056663 | 1.47E-09 | 9.16E-09 | Up |
| LINC01748 | 6.271452 | 2.88E-39 | 2.62E-37 | Up |
| LINC01918 | 3.3682633 | 3.21E-09 | 1.92E-08 | Up |
| UPK1A-AS1 | 3.0630174 | 0.000361256 | 0.000868325 | Up |
| CYP4F26P | 2.9616738 | 3.56E-09 | 2.10E-08 | Up |
| LINC00974 | 2.4436268 | 2.74E-06 | 9.97E-06 | Up |
| LINC01535 | 2.0817217 | 2.49E-06 | 9.15E-06 | Up |
| FGF12-AS3 | 2.7153357 | 0.000319326 | 0.000775149 | Up |
| AC007966.1 | 2.3604758 | 1.96E-08 | 1.04E-07 | Up |
| NAALADL2-AS2 | 5.9239799 | 1.44E-14 | 1.81E-13 | Up |
| AL023802.1 | 2.1501845 | 0.000667264 | 0.001519347 | Up |
| AC092167.1 | 3.6299318 | 3.05E-08 | 1.57E-07 | Up |
| LINC02527 | 2.8905562 | 2.14E-05 | 6.52E-05 | Up |
| LINC00511 | 4.1824613 | 7.96E-38 | 6.56E-36 | Up |
| LINC00629 | 2.3398577 | 2.60E-08 | 1.35E-07 | Up |
| Z98257.1 | 2.5230361 | 0.000360756 | 0.000867382 | Up |
| AC084149.1 | 4.5482193 | 2.07E-06 | 7.78E-06 | Up |
| AL512785.1 | 6.5648646 | 1.88E-08 | 9.96E-08 | Up |
| LINC01630 | 2.8707231 | 0.000705875 | 0.001597808 | Up |
| AC110015.1 | 3.6580192 | 1.87E-05 | 5.79E-05 | Up |
| DLG1-AS1 | 2.0320289 | 3.20E-09 | 1.92E-08 | Up |
| AC012501.1 | 6.3345209 | 1.91E-11 | 1.59E-10 | Up |
| AC099066.2 | 3.3251026 | 2.84E-12 | 2.68E-11 | Up |
| SLC2A1-AS1 | 3.3392099 | 1.07E-22 | 3.19E-21 | Up |
| AL122019.1 | 3.1818499 | 6.12E-06 | 2.08E-05 | Up |
| AL391056.1 | 2.0347421 | 1.90E-05 | 5.87E-05 | Up |
| SOX21-AS1 | 3.872735 | 6.79E-15 | 8.89E-14 | Up |
| STEAP2-AS1 | 2.432359 | 1.29E-07 | 5.98E-07 | Up |
| LINC00355 | 8.0672708 | 1.86E-15 | 2.60E-14 | Up |
| AL391097.2 | 2.3054113 | 7.82E-06 | 2.60E-05 | Up |
| LINC01850 | 3.6263316 | 1.06E-06 | 4.22E-06 | Up |
| MRLN | 2.6338397 | 0.001393552 | 0.002977603 | Up |
| SELENOOLP | 3.1660814 | 0.000244698 | 0.000609556 | Up |
| LINC01429 | 2.836715 | 2.61E-07 | 1.15E-06 | Up |
| RAPGEF4-AS1 | 2.7711925 | 2.03E-07 | 9.16E-07 | Up |
| AL160408.1 | 2.8739988 | 2.56E-07 | 1.14E-06 | Up |
| MELTF-AS1 | 2.9699084 | 2.21E-26 | 8.15E-25 | Up |
| LINC01494 | 2.9358385 | 2.25E-06 | 8.38E-06 | Up |
| AC097713.1 | 6.6567035 | 3.32E-12 | 3.11E-11 | Up |
| RNF144A-AS1 | 2.1180674 | 1.59E-08 | 8.52E-08 | Up |
| AL355607.1 | 2.6341353 | 8.83E-06 | 2.90E-05 | Up |
| LINC02048 | 2.2736787 | 0.000168335 | 0.000432808 | Up |
| PCAT6 | 3.5374669 | 5.04E-32 | 2.93E-30 | Up |
| TBX18-AS1 | 2.1178671 | 0.001121221 | 0.002441549 | Up |
| LINC00392 | 9.7767263 | 1.19E-09 | 7.52E-09 | Up |
| LINC01546 | 2.389775 | 2.34E-06 | 8.66E-06 | Up |
| AC106875.1 | 4.7846572 | 0.000112284 | 0.000299204 | Up |
| AL513283.1 | 4.6142653 | 3.46E-05 | 0.000101606 | Up |
| AC022387.1 | 3.0828897 | 0.000103535 | 0.000278609 | Up |
| AL138962.1 | 3.3124731 | 2.32E-06 | 8.60E-06 | Up |
| AC010731.2 | 4.4536651 | 1.39E-09 | 8.74E-09 | Up |
| AC005042.2 | 3.841045 | 3.56E-07 | 1.54E-06 | Up |
| AC007381.1 | 2.0446407 | 0.000600832 | 0.001381708 | Up |
| HOTAIR | 6.5082522 | 5.74E-11 | 4.44E-10 | Up |
| AL023755.1 | 2.6849102 | 1.53E-10 | 1.10E-09 | Up |
| LINC00659 | 2.6432245 | 4.12E-05 | 0.000119147 | Up |
| AL591806.1 | 2.1166106 | 2.09E-09 | 1.27E-08 | Up |
| LINC01344 | 2.5585658 | 1.58E-06 | 6.08E-06 | Up |
| AC107419.1 | 4.2029544 | 1.06E-08 | 5.83E-08 | Up |
| AL365356.3 | 4.9870889 | 3.37E-08 | 1.73E-07 | Up |
| LINC02041 | 2.8825456 | 8.53E-11 | 6.41E-10 | Up |
| SATB1-AS1 | 2.3833666 | 2.75E-13 | 2.94E-12 | Up |
| AL356479.1 | 3.8766103 | 3.61E-08 | 1.84E-07 | Up |
| LINC01205 | 5.4335106 | 6.04E-06 | 2.05E-05 | Up |
| AL445471.1 | 2.217772 | 1.03E-11 | 8.86E-11 | Up |
| AC233280.1 | 3.4880007 | 3.11E-16 | 4.80E-15 | Up |
| AC022201.1 | 5.3428514 | 3.50E-08 | 1.78E-07 | Up |
| LINC01981 | 5.0763108 | 9.27E-16 | 1.35E-14 | Up |
| AL596223.1 | 3.7135985 | 1.23E-05 | 3.95E-05 | Up |
| AL133353.1 | 2.7568795 | 2.58E-14 | 3.13E-13 | Up |
| AL353693.1 | 3.8285757 | 1.73E-11 | 1.45E-10 | Up |
| AL139246.4 | 2.8775406 | 1.03E-07 | 4.81E-07 | Up |
| AL596330.1 | 4.3936625 | 6.02E-07 | 2.49E-06 | Up |
| MIR5689HG | 2.6364733 | 4.04E-05 | 0.000117102 | Up |
| LINC00858 | 4.0059296 | 5.60E-11 | 4.34E-10 | Up |
| AL451062.1 | 3.3430457 | 2.40E-05 | 7.28E-05 | Up |
| AC053503.2 | 2.1589329 | 1.09E-07 | 5.08E-07 | Up |
| AL390115.1 | 2.3668268 | 0.000178552 | 0.000457042 | Up |
| AC011287.1 | 6.9736653 | 8.73E-20 | 1.97E-18 | Up |
| AL109914.1 | 2.0437298 | 0.001654257 | 0.003467718 | Up |
| MYOSLID | 3.4383465 | 1.62E-13 | 1.79E-12 | Up |
| AL161636.1 | 2.7684307 | 0.000132296 | 0.000347895 | Up |
| AL109924.2 | 3.4219213 | 2.20E-08 | 1.16E-07 | Up |
| STEAP3-AS1 | 2.1285722 | 1.59E-07 | 7.30E-07 | Up |
| CASC20 | 4.6342674 | 1.96E-09 | 1.20E-08 | Up |
| AL157373.2 | 2.1786684 | 9.31E-06 | 3.05E-05 | Up |
| AC128709.1 | 2.1347848 | 0.000148484 | 0.000386315 | Up |
| TFAP2A-AS1 | 3.1961428 | 2.72E-26 | 9.77E-25 | Up |
| AL590666.2 | 3.3187143 | 2.08E-08 | 1.10E-07 | Up |
| AL357143.1 | 2.6422702 | 1.91E-05 | 5.88E-05 | Up |
| AC080129.1 | 4.4997185 | 2.11E-06 | 7.92E-06 | Up |
| MAGEA4-AS1 | 8.1297435 | 1.37E-13 | 1.53E-12 | Up |
| AC007128.1 | 6.359577 | 1.01E-38 | 8.81E-37 | Up |
| AC006357.1 | 4.2211472 | 8.14E-07 | 3.30E-06 | Up |
| TRPM2-AS | 4.6113 | 1.06E-17 | 1.90E-16 | Up |
| AL022316.1 | 2.6490162 | 3.12E-07 | 1.36E-06 | Up |
| FGF12-AS2 | 2.6328658 | 7.77E-05 | 0.000213374 | Up |
| AP000688.1 | 2.0795019 | 6.86E-08 | 3.34E-07 | Up |
| NFE4 | 2.5682629 | 0.004185845 | 0.008093834 | Up |
| ELOVL2-AS1 | 3.2898611 | 1.54E-05 | 4.84E-05 | Up |
| FEZF1-AS1 | 5.8468422 | 3.14E-17 | 5.41E-16 | Up |
| AL121580.1 | 5.0031865 | 1.20E-15 | 1.72E-14 | Up |
| DSCR9 | 2.0082378 | 1.24E-11 | 1.06E-10 | Up |
| AL118508.1 | 3.3278518 | 1.68E-06 | 6.44E-06 | Up |
| AC114803.1 | 5.6423363 | 6.64E-15 | 8.71E-14 | Up |
| AL356234.2 | 5.6291246 | 1.94E-12 | 1.86E-11 | Up |
| TEX48 | 2.1886116 | 3.76E-05 | 0.000109906 | Up |
| KLHL7-DT | 2.1260396 | 5.69E-10 | 3.75E-09 | Up |
| AL645608.3 | 2.8741032 | 3.24E-09 | 1.93E-08 | Up |
| AL035252.2 | 2.6662248 | 9.17E-05 | 0.000249659 | Up |
| FOXD3-AS1 | 7.8315983 | 4.16E-33 | 2.59E-31 | Up |
| AC068580.2 | 3.9241525 | 6.33E-08 | 3.11E-07 | Up |
| LINC01614 | 3.434346 | 6.08E-13 | 6.25E-12 | Up |
| MIR205HG | 6.1222086 | 1.31E-27 | 5.45E-26 | Up |
| LINC02541 | 2.9521977 | 1.44E-11 | 1.21E-10 | Up |
| AL049649.1 | 6.2855178 | 3.23E-09 | 1.93E-08 | Up |
| AL355303.1 | 2.4840892 | 2.25E-07 | 1.01E-06 | Up |
| LINC02522 | 3.0595507 | 3.73E-05 | 0.000109037 | Up |
| LINC01468 | 8.4863142 | 1.33E-16 | 2.14E-15 | Up |
| AC093702.1 | 2.9077825 | 2.07E-06 | 7.77E-06 | Up |
| LINC01098 | 2.9490963 | 0.000167185 | 0.000429987 | Up |
| AC097347.1 | 3.2618402 | 4.43E-14 | 5.21E-13 | Up |
| APCDD1L-DT | 3.6746798 | 3.37E-09 | 2.01E-08 | Up |
| AC106799.1 | 6.1325981 | 4.03E-18 | 7.49E-17 | Up |
| AC004888.1 | 2.8107189 | 5.23E-06 | 1.80E-05 | Up |
| MANCR | 4.5777214 | 3.86E-11 | 3.06E-10 | Up |
| TBL1XR1-AS1 | 2.4565871 | 1.71E-07 | 7.78E-07 | Up |
| AP000696.1 | 5.3880916 | 2.57E-11 | 2.10E-10 | Up |
| LINC01160 | 2.1401615 | 3.19E-05 | 9.42E-05 | Up |
| AC005392.2 | 2.0592452 | 0.001381907 | 0.002958197 | Up |
| AL445673.1 | 2.3287193 | 3.69E-08 | 1.88E-07 | Up |
| WASIR2 | 2.596065 | 2.91E-08 | 1.51E-07 | Up |
| LINC01305 | 7.1147293 | 2.32E-17 | 4.05E-16 | Up |
| AC074389.2 | 4.8666385 | 5.78E-05 | 0.000162254 | Up |
| AC141930.2 | 2.4683163 | 0.000363659 | 0.000873062 | Up |
| AL365356.4 | 4.6702809 | 5.14E-14 | 5.97E-13 | Up |
| LINC01249 | 8.397215 | 2.02E-13 | 2.20E-12 | Up |
| CLEC12A-AS1 | 3.0181983 | 1.83E-07 | 8.30E-07 | Up |
| AC234772.2 | 3.1422593 | 7.71E-16 | 1.13E-14 | Up |
| AC009501.1 | 2.2913587 | 4.19E-09 | 2.44E-08 | Up |
| LUARIS | 4.5826693 | 1.00E-13 | 1.13E-12 | Up |
| FSIP2-AS1 | 2.4905427 | 2.31E-10 | 1.63E-09 | Up |
| LINC01698 | 6.7977671 | 3.63E-16 | 5.55E-15 | Up |
| AL033397.1 | 8.1633288 | 8.50E-12 | 7.46E-11 | Up |
| AL354993.1 | 2.9067837 | 8.09E-13 | 8.20E-12 | Up |
| AF064860.2 | 2.2053455 | 0.001386529 | 0.002964948 | Up |
| AC112907.1 | 2.8904103 | 4.65E-06 | 1.62E-05 | Up |
| ABCA9-AS1 | 5.1389868 | 2.22E-16 | 3.46E-15 | Up |
| CHODL-AS1 | 2.8699264 | 2.45E-05 | 7.39E-05 | Up |
| DLX6-AS1 | 7.1833832 | 1.76E-34 | 1.19E-32 | Up |
| Z93403.1 | 3.8407836 | 1.03E-14 | 1.32E-13 | Up |
| LINC01611 | 5.8923805 | 3.59E-11 | 2.86E-10 | Up |
| PCAT7 | 3.7112103 | 2.09E-20 | 4.91E-19 | Up |
| LINC01819 | 2.3606835 | 0.00374362 | 0.00728793 | Up |
| AL139393.1 | 3.5637935 | 2.72E-08 | 1.41E-07 | Up |
| AL031848.1 | 3.2743692 | 1.63E-09 | 1.01E-08 | Up |
| AL359551.1 | 5.0104139 | 3.88E-11 | 3.08E-10 | Up |
| DARS-AS1 | 2.1002572 | 7.76E-21 | 1.89E-19 | Up |
| AC116666.1 | 4.8569324 | 1.40E-07 | 6.44E-07 | Up |
| AL078590.2 | 4.4392847 | 1.09E-08 | 5.96E-08 | Up |
| AC005537.1 | 4.6905545 | 1.55E-26 | 5.79E-25 | Up |
| LEF1-AS1 | 2.2995035 | 9.47E-15 | 1.21E-13 | Up |
| LINC01807 | 6.8154443 | 2.50E-19 | 5.37E-18 | Up |
| LINC01063 | 2.8423154 | 7.43E-15 | 9.70E-14 | Up |
| LINC01697 | 3.8337453 | 1.87E-07 | 8.48E-07 | Up |
| LINC00867 | 2.2351841 | 0.004210801 | 0.00813082 | Up |
| LINC01873 | 5.2627734 | 1.10E-19 | 2.46E-18 | Up |
| Z99943.1 | 2.3169257 | 4.37E-06 | 1.53E-05 | Up |
| AL596442.1 | 3.0798288 | 0.000219092 | 0.000550351 | Up |
| AL353613.1 | 2.8323421 | 0.00027539 | 0.000677452 | Up |
| LINC02043 | 4.3096403 | 7.59E-16 | 1.12E-14 | Up |
| AL139327.2 | 5.9191866 | 3.93E-11 | 3.11E-10 | Up |
| AL031674.1 | 2.8557411 | 4.58E-05 | 0.000131251 | Up |
| AL138902.1 | 3.5316424 | 2.84E-17 | 4.91E-16 | Up |
| AC093627.1 | 4.3377746 | 8.40E-06 | 2.78E-05 | Up |
| AL354864.1 | 3.2958113 | 2.96E-05 | 8.80E-05 | Up |
| AC006329.1 | 2.8030818 | 6.98E-11 | 5.32E-10 | Up |
| AL354824.2 | 2.8651589 | 0.000105136 | 0.000282354 | Up |
| LARS2-AS1 | 2.2935391 | 3.78E-06 | 1.34E-05 | Up |
| AC092920.1 | 3.2636864 | 4.50E-07 | 1.90E-06 | Up |
| AC073323.1 | 2.2757523 | 0.000106374 | 0.00028511 | Up |
| AC104088.1 | 5.1051348 | 7.28E-20 | 1.66E-18 | Up |
| LINC01549 | 3.6162517 | 3.25E-07 | 1.41E-06 | Up |
| AL139125.1 | 2.1445382 | 3.69E-06 | 1.31E-05 | Up |
| AL122034.1 | 4.3012006 | 3.75E-07 | 1.61E-06 | Up |
| LINC02576 | 2.3249814 | 7.41E-10 | 4.84E-09 | Up |
| LINC00665 | 2.2149988 | 3.68E-09 | 2.17E-08 | Up |
| LINC01705 | 4.5714878 | 1.03E-15 | 1.49E-14 | Up |
| AC002511.1 | 2.0211852 | 0.001146893 | 0.002496105 | Up |
| AC097717.1 | 2.1507182 | 6.38E-09 | 3.62E-08 | Up |
| AC004990.1 | 2.3063455 | 8.32E-05 | 0.000227887 | Up |
| AL355483.3 | 3.026841 | 1.00E-06 | 4.00E-06 | Up |
| AC003986.2 | 2.5981553 | 9.97E-07 | 3.98E-06 | Up |
| AL590708.1 | 2.2169228 | 5.12E-09 | 2.95E-08 | Up |
| AF165147.1 | 3.6695289 | 4.39E-10 | 2.95E-09 | Up |
| AL157400.2 | 3.4774508 | 4.56E-10 | 3.06E-09 | Up |
| ERVH48-1 | 3.9650942 | 1.27E-05 | 4.05E-05 | Up |
| LINC01399 | 3.0260258 | 4.44E-07 | 1.88E-06 | Up |
| AC133785.1 | 7.3835033 | 7.35E-39 | 6.52E-37 | Up |
| AL512604.2 | 2.538739 | 6.78E-06 | 2.28E-05 | Up |
| XXYLT1-AS1 | 3.2238369 | 1.63E-10 | 1.17E-09 | Up |
| AL122008.3 | 2.9552913 | 2.37E-06 | 8.73E-06 | Up |
| AL391361.3 | 5.2703491 | 3.69E-10 | 2.50E-09 | Up |
| AC096537.1 | 3.2222294 | 2.06E-08 | 1.09E-07 | Up |
| AL445524.1 | 2.8332565 | 1.76E-16 | 2.78E-15 | Up |
| AC008163.1 | 4.3431421 | 0.000623146 | 0.00142633 | Up |
| AL021395.1 | 5.5237907 | 2.70E-06 | 9.84E-06 | Up |
| LINC01518 | 7.9561858 | 1.95E-11 | 1.62E-10 | Up |
| AC005162.2 | 2.9649006 | 2.54E-07 | 1.13E-06 | Up |
| LINC00460 | 3.230466 | 3.94E-06 | 1.39E-05 | Up |
| AC011294.1 | 2.3465135 | 0.000332642 | 0.000805056 | Up |
| AL161630.1 | 2.3652741 | 0.004281383 | 0.008253305 | Up |
| AL592043.1 | 5.7351382 | 6.71E-10 | 4.40E-09 | Up |
| AL138789.1 | 5.0508342 | 3.11E-14 | 3.74E-13 | Up |
| LINC00462 | 4.9058244 | 6.45E-09 | 3.66E-08 | Up |
| AC019068.1 | 3.2492399 | 4.08E-06 | 1.44E-05 | Up |
| MYCNOS | 3.6004456 | 2.10E-05 | 6.41E-05 | Up |
| LINC01251 | 3.4884388 | 7.71E-05 | 0.000211935 | Up |
| LINC01136 | 2.0404842 | 1.13E-11 | 9.69E-11 | Up |
| AP000695.2 | 2.1620752 | 3.14E-09 | 1.89E-08 | Up |
| KCNQ5-IT1 | 4.9048901 | 8.94E-13 | 9.03E-12 | Up |
| POU6F2-AS2 | 7.8545581 | 1.79E-31 | 9.98E-30 | Up |
| LINC01503 | 2.0830583 | 5.66E-14 | 6.52E-13 | Up |
| Z83851.1 | 2.5809241 | 4.43E-23 | 1.36E-21 | Up |
| AL591501.1 | 4.0672143 | 1.22E-06 | 4.80E-06 | Up |
| AC009970.1 | 2.278552 | 3.34E-06 | 1.20E-05 | Up |
| AC073529.1 | 2.1862503 | 2.75E-17 | 4.77E-16 | Up |
| LINC02535 | 3.5266811 | 5.53E-08 | 2.74E-07 | Up |
| KCNH1-IT1 | 5.1766968 | 2.69E-10 | 1.87E-09 | Up |
| AL138720.1 | 3.0707894 | 2.93E-12 | 2.76E-11 | Up |
| DEPDC1-AS1 | 3.7249476 | 2.36E-18 | 4.54E-17 | Up |
| LINC01731 | 2.059797 | 0.001688012 | 0.003527495 | Up |
| AC009264.1 | 7.7083105 | 1.18E-21 | 3.11E-20 | Up |
| AC098828.3 | 2.9491035 | 4.50E-05 | 0.000129377 | Up |
| LINC01250 | 3.0831412 | 1.53E-06 | 5.88E-06 | Up |
| AC147651.3 | 2.142303 | 0.003761885 | 0.007318197 | Up |
| AC092811.1 | 2.1834716 | 0.000101773 | 0.000274507 | Up |
| AC002076.1 | 3.301048 | 1.24E-14 | 1.56E-13 | Up |
| AF015262.1 | 3.8952615 | 2.34E-07 | 1.04E-06 | Up |
| AC005064.1 | 3.3315972 | 2.02E-05 | 6.21E-05 | Up |
| LINC01287 | 6.5118889 | 2.22E-07 | 9.93E-07 | Up |
| IQCM | 5.967265 | 3.77E-16 | 5.74E-15 | Up |
| AC003958.2 | 6.3578405 | 1.26E-12 | 1.23E-11 | Up |
| AL035258.1 | 6.7740693 | 1.10E-10 | 8.09E-10 | Up |
| LINC01524 | 4.4547264 | 2.45E-11 | 2.01E-10 | Up |
| AL512622.1 | 2.2724947 | 0.000156337 | 0.000405574 | Up |
| DPP10-AS1 | 2.21447 | 0.004292916 | 0.00826962 | Up |
| AC062015.1 | 6.6734032 | 6.41E-08 | 3.13E-07 | Up |
| BX324167.1 | 2.8149212 | 3.25E-07 | 1.41E-06 | Up |
| FAM237A | 5.3434586 | 4.95E-08 | 2.47E-07 | Up |
| LINC00601 | 3.6904122 | 1.82E-06 | 6.90E-06 | Up |
| AL022318.1 | 2.3442098 | 0.001492047 | 0.003162968 | Up |
| FAM83C-AS1 | 3.0003762 | 2.11E-15 | 2.91E-14 | Up |
| AF127577.3 | 8.2431529 | 2.49E-26 | 8.98E-25 | Up |
| LINC01634 | 3.11096 | 2.38E-06 | 8.79E-06 | Up |
| LINC01281 | 2.1025846 | 9.75E-07 | 3.91E-06 | Up |
| AC012360.1 | 2.5386219 | 4.73E-12 | 4.33E-11 | Up |
| AC016723.1 | 5.3155959 | 1.01E-06 | 4.03E-06 | Up |
| AC093083.1 | 3.1828181 | 3.98E-08 | 2.02E-07 | Up |
| LINC01967 | 6.6953395 | 9.58E-22 | 2.55E-20 | Up |
| AC103923.1 | 2.5263332 | 2.06E-14 | 2.53E-13 | Up |
| BARX1-DT | 7.1671288 | 1.04E-13 | 1.17E-12 | Up |
| LINC01647 | 3.3752175 | 0.000314082 | 0.000764256 | Up |
| AC007389.3 | 4.0867962 | 7.42E-08 | 3.59E-07 | Up |
| AC010148.1 | 3.3029302 | 1.84E-19 | 4.01E-18 | Up |
| AC099796.1 | 3.585892 | 4.30E-06 | 1.50E-05 | Up |
| LINC00941 | 3.8223001 | 1.82E-11 | 1.52E-10 | Up |
| LINC01564 | 4.1395676 | 8.05E-14 | 9.15E-13 | Up |
| AC007405.2 | 3.4990432 | 4.98E-08 | 2.48E-07 | Up |
| AL138760.1 | 3.6388795 | 3.46E-08 | 1.77E-07 | Up |
| AC004448.2 | 2.3946703 | 0.000577378 | 0.001331561 | Up |
| AL354766.2 | 2.7412691 | 3.85E-05 | 0.000112269 | Up |
| AC004920.1 | 3.8996398 | 6.66E-08 | 3.24E-07 | Up |
| AL034399.2 | 5.0856033 | 9.36E-21 | 2.25E-19 | Up |
| ELFN1-AS1 | 4.0355245 | 1.85E-08 | 9.86E-08 | Up |
| LINC01639 | 5.0295493 | 2.52E-06 | 9.25E-06 | Up |
| PTCSC2 | 4.3477131 | 1.62E-09 | 1.00E-08 | Up |
| KDM4A-AS1 | 2.5362359 | 5.67E-17 | 9.42E-16 | Up |
| AC009262.1 | 5.3751367 | 3.35E-23 | 1.03E-21 | Up |
| LINC01361 | 2.8879408 | 6.12E-10 | 4.03E-09 | Up |
| AC019197.1 | 2.9499918 | 5.75E-07 | 2.39E-06 | Up |
| SLC12A9-AS1 | 2.241864 | 1.15E-13 | 1.28E-12 | Up |
| AL354719.2 | 3.9535587 | 8.31E-10 | 5.38E-09 | Up |
| AL513123.1 | 7.530955 | 4.04E-22 | 1.11E-20 | Up |
| AL355472.3 | 2.5933388 | 7.55E-08 | 3.64E-07 | Up |
| AL359313.1 | 2.8210102 | 0.00040835 | 0.000972555 | Up |
| AL589986.2 | 3.5702427 | 2.45E-08 | 1.28E-07 | Up |
| FAM157A | 2.0124177 | 1.87E-09 | 1.15E-08 | Up |
| AC010894.3 | 4.092813 | 9.91E-10 | 6.35E-09 | Up |
| AC003092.1 | 4.0622091 | 1.17E-05 | 3.78E-05 | Up |
| LINC00896 | 2.7510931 | 5.15E-10 | 3.42E-09 | Up |
| AC074286.1 | 2.4197543 | 0.000150778 | 0.000392031 | Up |
| SIX3-AS1 | 2.5786521 | 0.001828505 | 0.003799473 | Up |
| ATP13A5-AS1 | 3.6084442 | 2.54E-06 | 9.31E-06 | Up |
| AC003986.3 | 2.6078668 | 0.000206015 | 0.000519765 | Up |
| AL033527.2 | 2.9433895 | 4.36E-08 | 2.19E-07 | Up |
| DLX2-DT | 5.844805 | 4.11E-13 | 4.33E-12 | Up |
| OVAAL | 2.9376149 | 0.001804871 | 0.003754225 | Up |
| AL033384.1 | 4.789906 | 7.63E-17 | 1.25E-15 | Up |
| AC024560.1 | 2.6125624 | 1.49E-06 | 5.75E-06 | Up |
| AC005865.1 | 2.9336883 | 5.72E-12 | 5.16E-11 | Up |
| AL162411.1 | 2.675713 | 2.39E-05 | 7.25E-05 | Up |
| AL139412.1 | 4.0095502 | 2.59E-14 | 3.15E-13 | Up |
| AL136131.2 | 2.3772963 | 0.001682936 | 0.003518707 | Up |
| MLIP-IT1 | 2.7299506 | 2.70E-07 | 1.19E-06 | Up |
| LINC01812 | 2.8909207 | 8.36E-05 | 0.000228948 | Up |
| LINC01792 | 2.5406645 | 0.000263547 | 0.000650894 | Up |
| AL445072.1 | 5.4009582 | 3.41E-13 | 3.60E-12 | Up |
| LINC01968 | 4.1165781 | 2.71E-15 | 3.70E-14 | Up |
| BX276092.7 | 7.0228532 | 8.09E-08 | 3.88E-07 | Up |
| LINC01343 | 3.1450465 | 0.000132282 | 0.000347895 | Up |
| AL034397.2 | 2.2142073 | 0.000412328 | 0.000981161 | Up |
| HOXD-AS2 | 3.9322154 | 5.19E-12 | 4.70E-11 | Up |
| CECR7 | 2.4217749 | 2.15E-06 | 8.03E-06 | Up |
| AL359771.1 | 4.3575733 | 1.44E-06 | 5.59E-06 | Up |
| UNC5B-AS1 | 2.048746 | 3.75E-07 | 1.61E-06 | Up |
| DGCR5 | 2.8818716 | 3.02E-11 | 2.43E-10 | Up |
| Z97206.2 | 2.3549068 | 2.57E-05 | 7.71E-05 | Up |
| AL590666.3 | 2.1736763 | 1.14E-05 | 3.67E-05 | Up |
| AP000251.1 | 4.6661323 | 3.42E-28 | 1.51E-26 | Up |
| AL161937.2 | 2.49086 | 5.69E-13 | 5.86E-12 | Up |
| AL139039.3 | 2.8614 | 8.69E-10 | 5.61E-09 | Up |
| AL109615.3 | 2.8433416 | 2.05E-10 | 1.46E-09 | Up |
| AC011287.2 | 3.1932545 | 1.67E-06 | 6.41E-06 | Up |
| AF130359.1 | 2.7434377 | 3.55E-05 | 0.000104093 | Up |
| LINC01143 | 3.4947465 | 4.35E-06 | 1.52E-05 | Up |
| AC010099.4 | 3.0291299 | 2.08E-07 | 9.38E-07 | Up |
| DDR1-DT | 2.2154134 | 9.42E-05 | 0.000255852 | Up |
| AC092422.1 | 2.3979579 | 0.000103136 | 0.000277815 | Up |
| AL035425.1 | 3.8193089 | 1.93E-06 | 7.31E-06 | Up |
| KCNMB2-AS1 | 7.5604809 | 3.10E-50 | 5.11E-48 | Up |
| AL606970.4 | 4.045133 | 2.14E-05 | 6.51E-05 | Up |
| SPATA3-AS1 | 2.216723 | 1.67E-06 | 6.40E-06 | Up |
| AC245100.6 | 3.458642 | 1.40E-08 | 7.55E-08 | Up |
| LINC00391 | 3.2984139 | 9.46E-07 | 3.80E-06 | Up |
| LINC00707 | 2.4418767 | 1.46E-05 | 4.61E-05 | Up |
| LINC01448 | 4.9627652 | 1.01E-06 | 4.01E-06 | Up |
| LINC02031 | 4.1553166 | 5.98E-08 | 2.95E-07 | Up |
| AC008040.1 | 2.3713451 | 7.42E-05 | 0.000204565 | Up |
| ATP6V1B1-AS1 | 3.0390789 | 3.30E-09 | 1.96E-08 | Up |
| AC019211.1 | 3.6494959 | 2.58E-12 | 2.44E-11 | Up |
| AC002310.2 | 2.3269683 | 4.58E-11 | 3.59E-10 | Up |
| AC069439.2 | 4.1344733 | 1.22E-05 | 3.91E-05 | Up |
| AC069431.1 | 3.6187546 | 2.48E-10 | 1.73E-09 | Up |
| AC128689.1 | 5.497549 | 1.72E-21 | 4.45E-20 | Up |
| AC055758.1 | 5.460171 | 1.12E-06 | 4.41E-06 | Up |
| SAMMSON | 3.4434198 | 2.97E-08 | 1.53E-07 | Up |
| LINC00973 | 4.1120117 | 4.57E-05 | 0.000131124 | Up |
| TM4SF1-AS1 | 2.6514708 | 1.79E-08 | 9.53E-08 | Up |
| AC063952.1 | 3.9054442 | 6.69E-17 | 1.10E-15 | Up |
| LSAMP-AS1 | 4.8359654 | 2.06E-14 | 2.53E-13 | Up |
| HOXA11-AS | 6.3890888 | 1.83E-19 | 4.01E-18 | Up |
| LINC01994 | 3.7743294 | 7.11E-07 | 2.91E-06 | Up |
| LINC01192 | 4.6858128 | 6.71E-06 | 2.26E-05 | Up |
| AL160408.4 | 5.7000086 | 1.40E-11 | 1.18E-10 | Up |
| PAQR9-AS1 | 2.0387006 | 0.002671182 | 0.00536427 | Up |
| AC123768.1 | 2.590748 | 3.61E-08 | 1.84E-07 | Up |
| AC112493.1 | 2.800084 | 0.000173781 | 0.00044511 | Up |
| AC084864.1 | 4.1003567 | 3.50E-17 | 5.98E-16 | Up |
| AC093904.2 | 3.5472258 | 2.34E-10 | 1.65E-09 | Up |
| AL365356.5 | 7.0459153 | 3.38E-19 | 7.16E-18 | Up |
| HOXB-AS4 | 5.01387 | 1.52E-09 | 9.46E-09 | Up |
| AC121764.1 | 4.1675184 | 0.000705862 | 0.001597808 | Up |
| LINC01206 | 9.4506708 | 1.33E-12 | 1.30E-11 | Up |
| LINC02005 | 3.517168 | 5.79E-06 | 1.98E-05 | Up |
| SOX2-OT | 5.0058243 | 3.64E-13 | 3.84E-12 | Up |
| AC011005.4 | 2.7443301 | 4.23E-13 | 4.43E-12 | Up |
| AC068633.1 | 3.9045191 | 0.000427293 | 0.001012893 | Up |
| LINC01998 | 3.4324143 | 0.000772393 | 0.001734723 | Up |
| MNX1-AS1 | 5.0843658 | 2.63E-11 | 2.14E-10 | Up |
| AC068985.1 | 4.7139574 | 7.44E-08 | 3.59E-07 | Up |
| LINC01214 | 4.7860328 | 5.37E-07 | 2.25E-06 | Up |
| CACNA2D3-AS1 | 3.0317958 | 7.69E-06 | 2.57E-05 | Up |
| HOTTIP | 6.3372336 | 1.15E-15 | 1.66E-14 | Up |
| AC108751.4 | 2.003992 | 4.81E-06 | 1.66E-05 | Up |
| AC117386.2 | 4.8814458 | 2.95E-07 | 1.29E-06 | Up |
| LINC01322 | 3.9839791 | 2.42E-10 | 1.69E-09 | Up |
| AC091153.3 | 2.845878 | 9.00E-14 | 1.02E-12 | Up |
| LINC01995 | 3.7480577 | 9.74E-06 | 3.18E-05 | Up |
| SIAH2-AS1 | 2.0003721 | 3.66E-09 | 2.16E-08 | Up |
| AC117394.2 | 2.3130559 | 1.26E-10 | 9.18E-10 | Up |
| AC093904.3 | 3.3080011 | 1.49E-06 | 5.74E-06 | Up |
| AL138759.1 | 2.2325632 | 3.12E-06 | 1.13E-05 | Up |
| LINC00698 | 2.2159019 | 0.000105947 | 0.000284156 | Up |
| AC055758.2 | 4.6418799 | 1.60E-05 | 5.01E-05 | Up |
| LINC01213 | 3.1994058 | 2.80E-05 | 8.35E-05 | Up |
| LINC01391 | 5.3358533 | 1.08E-08 | 5.89E-08 | Up |
| WNT5A-AS1 | 2.386779 | 1.02E-08 | 5.60E-08 | Up |
| LINC02086 | 3.2862878 | 4.88E-08 | 2.43E-07 | Up |
| AC108676.1 | 4.8216661 | 9.68E-12 | 8.41E-11 | Up |
| AL109761.1 | 2.6564326 | 4.32E-07 | 1.84E-06 | Up |
| AC087667.1 | 5.1879435 | 7.02E-11 | 5.34E-10 | Up |
| ARNTL2-AS1 | 4.1831332 | 3.77E-12 | 3.51E-11 | Up |
| AC026250.1 | 2.7320862 | 8.72E-19 | 1.78E-17 | Up |
| LINC00461 | 5.8406478 | 6.71E-10 | 4.40E-09 | Up |
| DDX11-AS1 | 2.9221209 | 1.28E-29 | 6.46E-28 | Up |
| AC022075.1 | 4.2421861 | 3.26E-23 | 1.01E-21 | Up |
| AC009292.1 | 2.7357201 | 5.71E-05 | 0.000160431 | Up |
| MIR4300HG | 2.5302113 | 0.000722979 | 0.001631497 | Up |
| AF233439.1 | 2.2484088 | 4.19E-06 | 1.47E-05 | Up |
| LINC01096 | 6.4515889 | 1.95E-23 | 6.12E-22 | Up |
| CASC8 | 4.9046075 | 1.44E-20 | 3.42E-19 | Up |
| AC138904.1 | 3.259589 | 1.43E-09 | 8.97E-09 | Up |
| AC079089.1 | 2.2616403 | 2.21E-06 | 8.23E-06 | Up |
| LINC02466 | 6.3771943 | 3.67E-18 | 6.84E-17 | Up |
| AC099487.1 | 6.3872189 | 7.03E-20 | 1.60E-18 | Up |
| SNCA-AS1 | 2.4390494 | 0.002119486 | 0.004349306 | Up |
| AC005329.1 | 2.6098262 | 4.05E-07 | 1.73E-06 | Up |
| AC008443.3 | 3.5368552 | 8.18E-08 | 3.91E-07 | Up |
| AC108174.1 | 4.9796992 | 2.17E-06 | 8.11E-06 | Up |
| LINC01194 | 8.1642637 | 3.35E-08 | 1.72E-07 | Up |
| LINC02505 | 4.4217263 | 0.000109751 | 0.000293482 | Up |
| LINC02438 | 5.7124515 | 3.53E-10 | 2.40E-09 | Up |
| AC114956.1 | 3.1098743 | 2.53E-13 | 2.71E-12 | Up |
| AC004053.1 | 2.4613793 | 8.59E-07 | 3.47E-06 | Up |
| LINC02014 | 2.8002555 | 1.14E-12 | 1.13E-11 | Up |
| AC010307.2 | 2.744857 | 5.56E-08 | 2.75E-07 | Up |
| AC023794.1 | 2.0664009 | 8.55E-08 | 4.07E-07 | Up |
| AC010275.1 | 6.7338707 | 1.01E-26 | 3.85E-25 | Up |
| AC010280.1 | 6.8178964 | 1.28E-15 | 1.83E-14 | Up |
| LINC00504 | 2.604961 | 2.33E-06 | 8.63E-06 | Up |
| AC011352.1 | 3.7579576 | 5.26E-09 | 3.01E-08 | Up |
| AC010395.1 | 2.1945757 | 8.77E-06 | 2.89E-05 | Up |
| AC016642.1 | 3.2827876 | 0.001960351 | 0.004049488 | Up |
| AC024230.1 | 5.5681793 | 3.20E-10 | 2.20E-09 | Up |
| LINC02437 | 4.6791563 | 4.69E-07 | 1.98E-06 | Up |
| AC022784.1 | 3.2367013 | 1.95E-06 | 7.36E-06 | Up |
| OTX2-AS1 | 4.5515684 | 8.19E-08 | 3.91E-07 | Up |
| AC114956.2 | 4.2355526 | 1.30E-18 | 2.59E-17 | Up |
| AC106882.1 | 2.3525323 | 2.49E-08 | 1.30E-07 | Up |
| GDNF-AS1 | 3.6547466 | 6.36E-09 | 3.61E-08 | Up |
| AC126768.1 | 4.9982362 | 6.48E-11 | 4.97E-10 | Up |
| LINC00992 | 2.5723291 | 1.83E-07 | 8.30E-07 | Up |
| LINC02428 | 7.3170911 | 8.75E-29 | 4.02E-27 | Up |
| AC106771.1 | 5.9809977 | 1.69E-06 | 6.46E-06 | Up |
| AC097512.1 | 5.5603092 | 1.83E-07 | 8.30E-07 | Up |
| AC131254.1 | 2.1750879 | 3.87E-05 | 0.000112736 | Up |
| AC012640.1 | 3.0663676 | 8.54E-15 | 1.10E-13 | Up |
| AC106799.2 | 7.4614848 | 7.29E-21 | 1.78E-19 | Up |
| AL133372.2 | 3.5763305 | 8.81E-06 | 2.90E-05 | Up |
| AC126768.2 | 3.2265167 | 1.01E-09 | 6.43E-09 | Up |
| AC093895.1 | 5.2240974 | 8.02E-29 | 3.70E-27 | Up |
| AC093599.1 | 5.6889114 | 3.27E-14 | 3.91E-13 | Up |
| LINC01033 | 2.2737628 | 0.003020401 | 0.005999965 | Up |
| C5orf66-AS1 | 8.2567841 | 5.78E-18 | 1.06E-16 | Up |
| AC027627.1 | 2.4661156 | 2.70E-05 | 8.07E-05 | Up |
| LINC02362 | 2.4319613 | 1.39E-10 | 1.01E-09 | Up |
| AC034223.1 | 5.1550632 | 3.03E-07 | 1.33E-06 | Up |
| AC025183.1 | 8.0143493 | 6.27E-16 | 9.30E-15 | Up |
| TMEM132D-AS1 | 7.9012255 | 2.24E-07 | 1.00E-06 | Up |
| AC026785.3 | 7.2178583 | 2.78E-12 | 2.63E-11 | Up |
| AC091868.2 | 3.8732678 | 4.22E-13 | 4.43E-12 | Up |
| AC025183.2 | 4.707185 | 5.51E-07 | 2.30E-06 | Up |
| AC093274.1 | 2.441095 | 0.000159528 | 0.000412395 | Up |
| CASC11 | 2.6027897 | 5.56E-10 | 3.68E-09 | Up |
| AL033397.2 | 3.9966227 | 4.33E-16 | 6.49E-15 | Up |
| CASC9 | 7.412399 | 9.28E-38 | 7.57E-36 | Up |
| IL20RB-AS1 | 5.0466423 | 7.90E-10 | 5.13E-09 | Up |
| AC116049.2 | 6.7804641 | 1.61E-28 | 7.26E-27 | Up |
| LINC02477 | 2.3656683 | 0.00135298 | 0.002900885 | Up |
| LINC01470 | 2.6429254 | 3.67E-05 | 0.000107355 | Up |
| AC023886.1 | 3.7051681 | 2.47E-05 | 7.44E-05 | Up |
| LINC01258 | 2.513139 | 0.000158216 | 0.000409921 | Up |
| LINC01234 | 7.888767 | 1.23E-19 | 2.72E-18 | Up |
| LINC02465 | 3.9004866 | 6.69E-09 | 3.78E-08 | Up |
| LINC00942 | 7.1215045 | 2.86E-11 | 2.32E-10 | Up |
| HOXC13-AS | 8.7618901 | 1.70E-29 | 8.36E-28 | Up |
| AC106772.1 | 3.3691699 | 2.03E-11 | 1.68E-10 | Up |
| AC106795.2 | 2.1596758 | 2.05E-09 | 1.25E-08 | Up |
| LINC02261 | 2.0762461 | 0.005016605 | 0.009540876 | Up |
| AC126768.3 | 3.667049 | 7.73E-07 | 3.15E-06 | Up |
| AC112178.1 | 5.723451 | 3.05E-07 | 1.33E-06 | Up |
| AC004704.1 | 3.8859613 | 4.23E-05 | 0.000122392 | Up |
| PVT1 | 2.4898859 | 1.21E-21 | 3.18E-20 | Up |
| AC024581.1 | 5.4627191 | 1.96E-21 | 4.99E-20 | Up |
| LINC02495 | 2.1225979 | 2.91E-05 | 8.68E-05 | Up |
| AC006487.1 | 2.0801379 | 0.000160397 | 0.000414235 | Up |
| LINC00536 | 6.6970022 | 1.15E-10 | 8.43E-10 | Up |
| LINC02223 | 2.8458722 | 7.24E-05 | 0.000199986 | Up |
| SLC7A11-AS1 | 2.4012834 | 1.32E-05 | 4.21E-05 | Up |
| AC096719.1 | 2.524865 | 3.31E-06 | 1.19E-05 | Up |
| AC114316.2 | 2.3686326 | 8.48E-06 | 2.80E-05 | Up |
| AC025176.1 | 2.1404195 | 2.25E-07 | 1.01E-06 | Up |
| LINC02377 | 7.704905 | 3.07E-07 | 1.34E-06 | Up |
| AC122694.1 | 6.2898277 | 3.26E-10 | 2.23E-09 | Up |
| HOXC-AS2 | 5.8308579 | 1.34E-22 | 3.95E-21 | Up |
| AC034206.1 | 4.4342504 | 1.18E-12 | 1.16E-11 | Up |
| AC068647.2 | 3.2416535 | 5.49E-07 | 2.29E-06 | Up |
| AC109439.2 | 3.8546746 | 0.000474258 | 0.001110013 | Up |
| AC114296.1 | 4.7976421 | 5.53E-13 | 5.71E-12 | Up |
| AP001790.1 | 6.2304447 | 5.29E-13 | 5.47E-12 | Up |
| LINC02502 | 2.9204826 | 6.10E-06 | 2.07E-05 | Up |
| AC106798.1 | 4.5145395 | 1.84E-15 | 2.57E-14 | Up |
| CLSTN2-AS1 | 2.4926246 | 2.51E-05 | 7.54E-05 | Up |
| HOXC-AS1 | 3.9875147 | 2.05E-13 | 2.24E-12 | Up |
| AP002784.1 | 4.3854655 | 3.02E-11 | 2.43E-10 | Up |
| AC079160.1 | 7.8685636 | 4.40E-38 | 3.67E-36 | Up |
| AC109454.3 | 5.5846195 | 3.74E-09 | 2.20E-08 | Up |
| LINC01511 | 5.4300942 | 8.01E-06 | 2.66E-05 | Up |
| AC083906.3 | 2.1560608 | 0.000172664 | 0.000442389 | Up |
| LINC00491 | 7.552835 | 1.66E-31 | 9.40E-30 | Up |
| AC009123.1 | 4.2540161 | 9.42E-17 | 1.53E-15 | Up |
| AC010343.3 | 5.2465852 | 1.15E-10 | 8.44E-10 | Up |
| LINC02269 | 4.0025324 | 2.35E-07 | 1.05E-06 | Up |
| AC096759.2 | 6.3554076 | 4.53E-14 | 5.33E-13 | Up |
| AC005865.2 | 2.3206515 | 1.29E-06 | 5.06E-06 | Up |
| LINC02111 | 3.2546879 | 1.27E-05 | 4.05E-05 | Up |
| AC108865.1 | 3.0227071 | 0.000851946 | 0.001901818 | Up |
| AC010595.1 | 7.6000431 | 6.80E-32 | 3.90E-30 | Up |
| LINC02208 | 3.0806671 | 2.76E-06 | 1.01E-05 | Up |
| AL035458.2 | 2.254007 | 1.61E-09 | 9.97E-09 | Up |
| AC105460.1 | 8.0595904 | 1.06E-08 | 5.83E-08 | Up |
| LINC00492 | 2.8319709 | 3.80E-07 | 1.63E-06 | Up |
| LINC02382 | 2.9416763 | 0.000729228 | 0.001645141 | Up |
| AC020551.1 | 5.3662457 | 3.73E-07 | 1.61E-06 | Up |
| ZFPM2-AS1 | 5.09288 | 3.26E-17 | 5.60E-16 | Up |
| LINC02163 | 7.6676107 | 2.69E-33 | 1.70E-31 | Up |
| LINC02506 | 3.1694748 | 0.002020337 | 0.004162759 | Up |
| AC090502.1 | 4.8138585 | 1.95E-06 | 7.38E-06 | Up |
| AC113346.1 | 5.2530308 | 1.93E-16 | 3.04E-15 | Up |
| HOXC-AS3 | 6.6948529 | 1.26E-11 | 1.07E-10 | Up |
| AC025244.1 | 5.8199518 | 1.51E-14 | 1.89E-13 | Up |
| AC034223.2 | 4.8454931 | 5.02E-08 | 2.50E-07 | Up |
| AC011352.3 | 5.4484463 | 1.75E-14 | 2.17E-13 | Up |
| LINC02475 | 3.5921599 | 2.33E-06 | 8.64E-06 | Up |
| AC012625.1 | 3.6510004 | 1.13E-09 | 7.15E-09 | Up |
| LINC02315 | 3.6489733 | 1.62E-09 | 1.01E-08 | Up |
| AC122710.2 | 2.3123035 | 2.36E-06 | 8.70E-06 | Up |
| LINC00958 | 5.8659958 | 1.77E-22 | 5.10E-21 | Up |
| AC104071.1 | 5.3375271 | 0.000318877 | 0.000774294 | Up |
| AC096734.1 | 2.7003711 | 3.44E-06 | 1.23E-05 | Up |
| AC099520.1 | 4.9595534 | 2.37E-08 | 1.24E-07 | Up |
| AC105460.2 | 5.4014187 | 5.21E-10 | 3.45E-09 | Up |
| LINC01385 | 5.0186226 | 4.83E-12 | 4.42E-11 | Up |
| STPG2-AS1 | 2.0524823 | 0.003413261 | 0.006699675 | Up |
| LINC02241 | 3.693938 | 0.000424617 | 0.001007438 | Up |
| AC120042.2 | 2.9892027 | 2.59E-07 | 1.14E-06 | Up |
| AC100801.1 | 7.0999685 | 3.12E-06 | 1.12E-05 | Up |
| LINC01605 | 4.2337375 | 4.98E-13 | 5.16E-12 | Up |
| AC109479.1 | 2.6399539 | 1.30E-07 | 6.03E-07 | Up |
| AC009630.2 | 3.2543697 | 2.42E-09 | 1.46E-08 | Up |
| AC138356.3 | 3.8603156 | 2.19E-05 | 6.66E-05 | Up |
| HOXA10-AS | 5.8732454 | 9.10E-17 | 1.48E-15 | Up |
| AC083841.1 | 2.7062974 | 1.14E-05 | 3.69E-05 | Up |
| AP001574.1 | 2.3990167 | 1.10E-05 | 3.55E-05 | Up |
| AC090192.2 | 3.4344762 | 1.51E-06 | 5.82E-06 | Up |
| AC068228.1 | 4.677655 | 1.22E-10 | 8.91E-10 | Up |
| AC078906.1 | 2.4567723 | 3.00E-07 | 1.32E-06 | Up |
| AC111149.2 | 3.9350293 | 1.57E-05 | 4.92E-05 | Up |
| AP003469.1 | 2.159888 | 3.29E-05 | 9.72E-05 | Up |
| AC084024.3 | 2.7420065 | 4.51E-06 | 1.57E-05 | Up |
| AC008708.1 | 2.8931948 | 0.004467481 | 0.008577266 | Up |
| AC069120.1 | 3.95713 | 1.62E-06 | 6.22E-06 | Up |
| AC090809.1 | 6.7682494 | 1.44E-07 | 6.64E-07 | Up |
| AC131902.1 | 3.5516418 | 0.000153702 | 0.00039912 | Up |
| AC021785.1 | 4.397684 | 3.37E-08 | 1.73E-07 | Up |
| LINC00534 | 2.2146128 | 0.000688814 | 0.001562691 | Up |
| AP003469.2 | 3.0091649 | 3.46E-19 | 7.32E-18 | Up |
| AC083973.1 | 4.5456593 | 2.98E-18 | 5.64E-17 | Up |
| AC124067.2 | 2.4364232 | 4.02E-07 | 1.72E-06 | Up |
| LINC02159 | 3.5227816 | 9.81E-11 | 7.26E-10 | Up |
| PCAT1 | 2.0083188 | 1.17E-08 | 6.37E-08 | Up |
| AC022568.1 | 3.6426231 | 0.001543014 | 0.003259894 | Up |
| AC012213.1 | 6.8088177 | 1.62E-19 | 3.58E-18 | Up |
| AF121898.1 | 3.5939856 | 2.45E-06 | 8.98E-06 | Up |
| AC004080.2 | 4.9431804 | 6.07E-12 | 5.43E-11 | Up |
| AC120193.1 | 2.4518561 | 0.000113951 | 0.000303108 | Up |
| AC090568.2 | 2.8396758 | 1.01E-07 | 4.73E-07 | Up |
| AC022639.1 | 6.8542203 | 1.40E-16 | 2.24E-15 | Up |
| AC018953.1 | 4.5191551 | 2.75E-07 | 1.21E-06 | Up |
| AF279873.3 | 5.8786737 | 1.88E-05 | 5.82E-05 | Up |
| LINC01592 | 3.7905576 | 1.07E-08 | 5.88E-08 | Up |
| AC008464.1 | 5.2939829 | 1.87E-06 | 7.08E-06 | Up |
| ZFHX4-AS1 | 4.7190956 | 1.02E-06 | 4.07E-06 | Up |
| AC022733.1 | 2.9929419 | 0.001155734 | 0.002511964 | Up |
| AP000424.1 | 3.0845889 | 1.77E-06 | 6.75E-06 | Up |
| AC011632.1 | 7.7365669 | 3.39E-22 | 9.46E-21 | Up |
| AC022274.1 | 2.717535 | 9.09E-07 | 3.66E-06 | Up |
| AC083841.2 | 3.7021936 | 1.45E-06 | 5.61E-06 | Up |
| AC108002.1 | 2.4581707 | 9.19E-12 | 8.02E-11 | Up |
| AC091182.2 | 4.2655418 | 9.39E-12 | 8.17E-11 | Up |
| AC091946.1 | 4.0278776 | 3.69E-05 | 0.000107964 | Up |
| AC008663.1 | 4.7628027 | 1.32E-08 | 7.14E-08 | Up |
| AC104248.1 | 2.26287 | 1.48E-05 | 4.68E-05 | Up |
| AC105999.2 | 9.5553721 | 2.43E-06 | 8.95E-06 | Up |
| AC018616.1 | 4.3342284 | 7.12E-11 | 5.41E-10 | Up |
| FER1L6-AS2 | 4.7109683 | 1.90E-05 | 5.87E-05 | Up |
| AC023202.1 | 2.8945148 | 0.000125127 | 0.000330441 | Up |
| LINC01419 | 10.423454 | 1.99E-08 | 1.05E-07 | Up |
| AC103409.1 | 4.1678291 | 9.33E-05 | 0.000253537 | Up |
| AC105118.1 | 5.1695411 | 1.45E-14 | 1.82E-13 | Up |
| AC007991.3 | 2.6320482 | 0.001075929 | 0.002348618 | Up |
| AC016573.1 | 2.2950167 | 4.65E-06 | 1.62E-05 | Up |
| LINC00051 | 4.6191615 | 1.51E-05 | 4.75E-05 | Up |
| AC092818.1 | 2.4174159 | 4.39E-05 | 0.000126451 | Up |
| AC034154.1 | 4.9277397 | 2.48E-08 | 1.29E-07 | Up |
| AP001208.2 | 3.1308094 | 5.04E-08 | 2.51E-07 | Up |
| AC025524.2 | 2.4918524 | 0.000297629 | 0.000727508 | Up |
| AC025434.1 | 2.5334276 | 6.80E-05 | 0.00018929 | Up |
| CASC19 | 6.5774062 | 2.74E-15 | 3.73E-14 | Up |
| LINC01485 | 2.5968279 | 2.72E-08 | 1.41E-07 | Up |
| AC100782.1 | 3.4017497 | 2.71E-09 | 1.63E-08 | Up |
| AC037486.1 | 4.8133073 | 1.36E-08 | 7.34E-08 | Up |
| AC083967.1 | 4.212707 | 4.84E-05 | 0.000137678 | Up |
| MAFA-AS1 | 4.7769148 | 5.76E-12 | 5.19E-11 | Up |
| MIR2052HG | 4.6029203 | 1.04E-18 | 2.09E-17 | Up |
| AP000424.2 | 2.0328068 | 0.000282622 | 0.000693132 | Up |
| AC022762.1 | 2.6070612 | 9.01E-06 | 2.96E-05 | Up |
| AC015689.1 | 2.161178 | 6.33E-08 | 3.11E-07 | Up |
| AL136088.1 | 2.8450428 | 0.000124308 | 0.000328709 | Up |
| AC044839.2 | 5.5628033 | 3.12E-07 | 1.36E-06 | Up |
| AC105219.2 | 3.0339573 | 1.20E-09 | 7.56E-09 | Up |
| AC080023.1 | 2.74767 | 2.88E-13 | 3.07E-12 | Up |
| BBOX1-AS1 | 6.9009017 | 3.81E-42 | 3.90E-40 | Up |
| AP001783.1 | 2.8484847 | 9.16E-05 | 0.000249659 | Up |
| AC027031.2 | 2.4020129 | 4.42E-12 | 4.07E-11 | Up |
| AP003119.1 | 3.8448478 | 3.31E-06 | 1.19E-05 | Up |
| AC018716.1 | 4.7933753 | 1.03E-06 | 4.08E-06 | Up |
| AC009646.2 | 2.6086706 | 0.001670247 | 0.003496702 | Up |
| LINC02551 | 2.2236639 | 4.57E-05 | 0.0001311 | Up |
| AP003390.1 | 4.3967686 | 5.36E-28 | 2.32E-26 | Up |
| AP001547.1 | 4.51699 | 1.24E-05 | 3.97E-05 | Up |
| AP003063.1 | 3.7154185 | 1.26E-07 | 5.84E-07 | Up |
| AC105219.4 | 2.5204699 | 5.45E-17 | 9.06E-16 | Up |
| AC124276.1 | 3.4544748 | 3.73E-08 | 1.90E-07 | Up |
| AF131216.3 | 5.0661636 | 4.69E-11 | 3.67E-10 | Up |
| AC093496.1 | 2.8521307 | 0.000670816 | 0.001526574 | Up |
| AC067930.5 | 2.8997076 | 1.59E-10 | 1.14E-09 | Up |
| GRM5-AS1 | 3.2802278 | 2.36E-07 | 1.05E-06 | Up |
| AP003119.2 | 3.1410401 | 2.50E-18 | 4.80E-17 | Up |
| OVOL1-AS1 | 2.4905742 | 2.03E-07 | 9.17E-07 | Up |
| AP000880.1 | 2.1654813 | 1.92E-08 | 1.02E-07 | Up |
| AP001360.2 | 7.7576699 | 3.90E-11 | 3.08E-10 | Up |
| AP003390.2 | 3.1031144 | 7.99E-08 | 3.84E-07 | Up |
| AC018716.2 | 4.9222388 | 7.60E-08 | 3.67E-07 | Up |
| SMIM35 | 2.5023496 | 4.84E-08 | 2.42E-07 | Up |
| RRM1-AS1 | 2.0045256 | 1.02E-05 | 3.31E-05 | Up |
| AC108136.1 | 5.3888221 | 8.68E-14 | 9.84E-13 | Up |
| AP002957.1 | 2.229302 | 1.46E-05 | 4.61E-05 | Up |
| SMILR | 4.2212954 | 8.78E-11 | 6.58E-10 | Up |
| AP002008.3 | 2.242764 | 0.002011771 | 0.004146167 | Up |
| AL137804.1 | 2.9879833 | 3.44E-07 | 1.49E-06 | Up |
| MIR9-3HG | 3.115074 | 5.32E-14 | 6.15E-13 | Up |
| AP000820.1 | 2.3683276 | 0.000537529 | 0.001244634 | Up |
| AC008114.1 | 2.107855 | 2.22E-06 | 8.28E-06 | Up |
| FAM222A-AS1 | 2.2336482 | 1.10E-06 | 4.35E-06 | Up |
| AC022509.1 | 3.981092 | 1.76E-07 | 8.01E-07 | Up |
| AC026310.2 | 4.395973 | 6.34E-10 | 4.17E-09 | Up |
| AC006064.3 | 2.0058882 | 3.35E-09 | 1.99E-08 | Up |
| AP000439.3 | 4.0289956 | 0.000886221 | 0.001968541 | Up |
| AC007848.1 | 4.5173981 | 3.20E-18 | 6.02E-17 | Up |
| AC125616.1 | 3.938663 | 8.11E-08 | 3.88E-07 | Up |
| AC090673.2 | 3.7498189 | 4.28E-08 | 2.16E-07 | Up |
| LINC02443 | 4.740484 | 1.68E-15 | 2.35E-14 | Up |
| AC006206.1 | 3.319126 | 6.79E-07 | 2.80E-06 | Up |
| AC022075.2 | 3.4488071 | 2.69E-11 | 2.18E-10 | Up |
| AP003721.1 | 2.8725499 | 2.75E-13 | 2.94E-12 | Up |
| AC007848.2 | 3.5635492 | 3.28E-08 | 1.69E-07 | Up |
| LINC02387 | 3.5704933 | 9.11E-09 | 5.07E-08 | Up |
| LINC02454 | 3.7299479 | 1.78E-07 | 8.08E-07 | Up |
| AC087258.1 | 4.670848 | 2.32E-06 | 8.63E-06 | Up |
| AC006206.2 | 5.4357413 | 2.88E-29 | 1.39E-27 | Up |
| AC010186.1 | 2.0861243 | 1.22E-05 | 3.91E-05 | Up |
| AP003559.1 | 2.6291576 | 8.85E-09 | 4.93E-08 | Up |
| AC023796.2 | 2.06869 | 6.25E-05 | 0.000175049 | Up |
| LINC02393 | 6.445251 | 1.54E-08 | 8.25E-08 | Up |
| AC087318.1 | 2.8294688 | 2.17E-06 | 8.11E-06 | Up |
| AP000851.1 | 4.6590542 | 5.98E-07 | 2.48E-06 | Up |
| AC084819.1 | 4.3706851 | 2.15E-06 | 8.05E-06 | Up |
| AP001453.2 | 3.5592147 | 1.12E-22 | 3.31E-21 | Up |
| AC131009.2 | 3.8945192 | 5.04E-11 | 3.93E-10 | Up |
| AC135782.1 | 2.2783497 | 0.000220601 | 0.000553627 | Up |
| AC084816.1 | 5.9010665 | 5.33E-12 | 4.82E-11 | Up |
| AC008115.2 | 2.6180045 | 3.11E-06 | 1.12E-05 | Up |
| AC008011.2 | 2.6849622 | 1.57E-06 | 6.05E-06 | Up |
| LINC02417 | 2.9211617 | 6.87E-05 | 0.000190694 | Up |
| TMPO-AS1 | 2.4913559 | 6.60E-28 | 2.82E-26 | Up |
| AC090709.1 | 3.1406406 | 3.48E-11 | 2.77E-10 | Up |
| AC131157.1 | 6.3347684 | 2.47E-11 | 2.03E-10 | Up |
| AC023511.1 | 4.9151616 | 2.37E-07 | 1.05E-06 | Up |
| AC123905.1 | 3.5975514 | 6.36E-08 | 3.11E-07 | Up |
| AC126177.4 | 2.2232483 | 0.002746758 | 0.005507829 | Up |
| AC068305.2 | 2.2976457 | 7.85E-08 | 3.77E-07 | Up |
| AC025575.1 | 3.2835542 | 6.79E-07 | 2.80E-06 | Up |
| AC055736.1 | 5.7494941 | 2.82E-09 | 1.70E-08 | Up |
| AC011601.1 | 4.4269168 | 1.35E-07 | 6.24E-07 | Up |
| LINC01475 | 4.1099381 | 3.72E-06 | 1.32E-05 | Up |
| AC078778.1 | 2.2763197 | 1.82E-18 | 3.59E-17 | Up |
| G2E3-AS1 | 7.9924252 | 1.35E-27 | 5.58E-26 | Up |
| AC089983.1 | 3.3550454 | 2.58E-07 | 1.14E-06 | Up |
| AC078860.1 | 3.3897057 | 5.49E-08 | 2.72E-07 | Up |
| LINC02588 | 5.6150452 | 3.93E-08 | 2.00E-07 | Up |
| AL139023.1 | 7.0158317 | 1.58E-15 | 2.23E-14 | Up |
| LINC02404 | 6.5132627 | 1.32E-06 | 5.16E-06 | Up |
| LINC02156 | 3.6952116 | 4.86E-17 | 8.14E-16 | Up |
| LINC02416 | 3.8024507 | 7.62E-18 | 1.38E-16 | Up |
| AC008083.1 | 3.9353189 | 6.21E-08 | 3.05E-07 | Up |
| LINC02457 | 5.7539098 | 2.04E-11 | 1.69E-10 | Up |
| AC025575.2 | 6.3494649 | 3.65E-16 | 5.58E-15 | Up |
| AC078923.1 | 2.9378776 | 2.83E-06 | 1.03E-05 | Up |
| AC078820.1 | 2.2374985 | 0.00024502 | 0.000610158 | Up |
| AC089987.2 | 3.8268606 | 1.52E-09 | 9.43E-09 | Up |
| LINC02444 | 3.5900155 | 1.65E-06 | 6.34E-06 | Up |
| LINC00485 | 2.1072088 | 0.000779116 | 0.001748002 | Up |
| AC002351.1 | 6.1207875 | 3.72E-09 | 2.19E-08 | Up |
| LINC00592 | 2.8485193 | 1.40E-12 | 1.36E-11 | Up |
| AC090503.2 | 3.9236104 | 2.25E-06 | 8.38E-06 | Up |
| AL589182.1 | 5.792154 | 4.65E-09 | 2.69E-08 | Up |
| LINC02313 | 3.7673268 | 2.14E-07 | 9.60E-07 | Up |
| LINC00640 | 3.413531 | 3.91E-16 | 5.93E-15 | Up |
| AL049830.3 | 3.0189612 | 8.25E-10 | 5.34E-09 | Up |
| AL132712.1 | 2.0765363 | 2.84E-13 | 3.03E-12 | Up |
| AL079307.1 | 3.4230701 | 6.93E-07 | 2.85E-06 | Up |
| LINC00645 | 3.097122 | 2.92E-07 | 1.28E-06 | Up |
| AL136298.1 | 3.3622388 | 7.71E-06 | 2.57E-05 | Up |
| AL391152.1 | 3.3863459 | 1.88E-15 | 2.61E-14 | Up |
| LINC01629 | 5.0648795 | 7.71E-09 | 4.32E-08 | Up |
| AL049870.2 | 2.3727179 | 0.00164234 | 0.003446399 | Up |
| AC026495.1 | 2.3231152 | 0.000210506 | 0.000529937 | Up |
| AL136018.1 | 5.6372513 | 6.88E-11 | 5.26E-10 | Up |
| AL079303.1 | 3.4801722 | 2.55E-11 | 2.09E-10 | Up |
| AL049874.3 | 3.9056795 | 2.32E-06 | 8.60E-06 | Up |
| LINC01269 | 3.3845145 | 1.03E-08 | 5.64E-08 | Up |
| LINC00871 | 5.4819189 | 1.73E-11 | 1.45E-10 | Up |
| AL358334.2 | 4.9517365 | 3.46E-16 | 5.32E-15 | Up |
| LINC02301 | 4.2150646 | 9.92E-05 | 0.000268117 | Up |
| AC122685.1 | 6.2813083 | 4.82E-10 | 3.23E-09 | Up |
| LINC00520 | 4.2001153 | 4.51E-11 | 3.53E-10 | Up |
| AL133153.2 | 2.3475286 | 3.51E-10 | 2.39E-09 | Up |
| LINC02310 | 4.17191 | 1.53E-08 | 8.22E-08 | Up |
| AC131532.1 | 6.0708532 | 6.77E-21 | 1.66E-19 | Up |
| AL133370.1 | 6.0998473 | 1.33E-07 | 6.16E-07 | Up |
| LINC02321 | 3.0944088 | 2.08E-13 | 2.26E-12 | Up |
| EGLN3-AS1 | 3.3683211 | 4.56E-05 | 0.000130898 | Up |
| AL049775.2 | 3.0854193 | 7.18E-05 | 0.000198453 | Up |
| LINC01956 | 5.0549196 | 7.90E-07 | 3.21E-06 | Up |
| AL133467.2 | 5.0203821 | 5.09E-08 | 2.53E-07 | Up |
| AL358332.1 | 2.5964894 | 1.65E-10 | 1.18E-09 | Up |
| SALRNA1 | 2.2440977 | 6.98E-08 | 3.39E-07 | Up |
| LINC00519 | 6.1918606 | 4.30E-24 | 1.41E-22 | Up |
| AC244502.3 | 2.2737722 | 6.77E-05 | 0.000188604 | Up |
| LINC02332 | 2.2342344 | 8.01E-07 | 3.26E-06 | Up |
| LINC00648 | 3.2485412 | 3.43E-05 | 0.000100754 | Up |
| AL161757.5 | 4.0089175 | 2.33E-06 | 8.63E-06 | Up |
| AC004816.1 | 2.59466 | 7.33E-17 | 1.21E-15 | Up |
| AC023906.2 | 6.4404464 | 2.13E-18 | 4.16E-17 | Up |
| LINC00928 | 4.5941275 | 1.43E-12 | 1.39E-11 | Up |
| LINC02323 | 3.1162056 | 7.99E-11 | 6.01E-10 | Up |
| MIR4713HG | 5.7493657 | 2.01E-31 | 1.10E-29 | Up |
| AC022405.1 | 2.3024645 | 3.28E-06 | 1.18E-05 | Up |
| AC109462.1 | 3.237581 | 4.80E-07 | 2.02E-06 | Up |
| AC020891.2 | 6.3852601 | 6.75E-29 | 3.13E-27 | Up |
| AC015660.1 | 3.593404 | 1.05E-08 | 5.78E-08 | Up |
| AC025580.1 | 2.4533278 | 1.06E-05 | 3.45E-05 | Up |
| AC013652.1 | 3.7642028 | 1.27E-27 | 5.31E-26 | Up |
| AC087473.1 | 2.2928211 | 0.000980157 | 0.002155865 | Up |
| LINC01833 | 6.9212007 | 3.96E-11 | 3.13E-10 | Up |
| AC025219.1 | 2.2330658 | 4.15E-09 | 2.43E-08 | Up |
| AC013652.2 | 2.7374642 | 1.29E-06 | 5.06E-06 | Up |
| LINC02253 | 6.98553 | 2.09E-19 | 4.50E-18 | Up |
| AC027243.1 | 3.2826081 | 1.10E-09 | 7.02E-09 | Up |
| LINC01583 | 3.4437925 | 2.17E-09 | 1.32E-08 | Up |
| AC067863.1 | 4.5166728 | 2.11E-12 | 2.02E-11 | Up |
| LINC01491 | 3.7021433 | 2.99E-05 | 8.89E-05 | Up |
| AC023034.1 | 3.2023875 | 7.43E-14 | 8.46E-13 | Up |
| AC023034.2 | 3.5692053 | 1.89E-14 | 2.34E-13 | Up |
| AC010478.1 | 2.0421657 | 0.001304494 | 0.002805934 | Up |
| LINC02254 | 2.7319051 | 2.70E-06 | 9.84E-06 | Up |
| AC087612.1 | 6.8802625 | 1.02E-07 | 4.78E-07 | Up |
| IDH2-DT | 2.8430055 | 7.71E-06 | 2.57E-05 | Up |
| AC104041.1 | 4.1018053 | 1.66E-19 | 3.65E-18 | Up |
| AC020891.3 | 4.5735706 | 5.01E-16 | 7.45E-15 | Up |
| AL354993.2 | 3.5334131 | 1.04E-20 | 2.48E-19 | Up |
| AC103740.2 | 3.9828199 | 4.06E-08 | 2.05E-07 | Up |
| AC100839.2 | 5.1075555 | 8.25E-07 | 3.34E-06 | Up |
| AC007907.1 | 2.307717 | 0.000703057 | 0.001593216 | Up |
| LINC02109 | 5.1973608 | 7.44E-10 | 4.85E-09 | Up |
| AC012640.2 | 2.4290613 | 3.48E-19 | 7.33E-18 | Up |
| AL163952.1 | 4.2250179 | 7.38E-09 | 4.15E-08 | Up |
| LINC01992 | 7.1492015 | 1.24E-09 | 7.80E-09 | Up |
| AC112176.1 | 4.1210936 | 1.63E-07 | 7.45E-07 | Up |
| AC012174.1 | 3.533552 | 6.14E-09 | 3.50E-08 | Up |
| LINC00556 | 2.5143495 | 0.000756726 | 0.001703847 | Up |
| AL032819.1 | 2.6310465 | 1.80E-08 | 9.60E-08 | Up |
| AC093249.2 | 2.1075313 | 9.77E-12 | 8.48E-11 | Up |
| AC126696.1 | 2.1131747 | 4.36E-05 | 0.000125885 | Up |
| LINC02137 | 3.0855602 | 2.70E-08 | 1.40E-07 | Up |
| AC000032.1 | 3.7618643 | 0.000338721 | 0.000818297 | Up |
| AC124944.3 | 2.4796854 | 3.68E-15 | 4.94E-14 | Up |
| LINC02562 | 3.3043828 | 7.45E-18 | 1.36E-16 | Up |
| AC093515.1 | 5.1654413 | 2.48E-07 | 1.10E-06 | Up |
| AC083801.2 | 5.9181284 | 4.98E-13 | 5.16E-12 | Up |
| Z92544.1 | 2.0367487 | 1.72E-06 | 6.54E-06 | Up |
| AL353746.1 | 2.3445557 | 0.000258426 | 0.000638831 | Up |
| LINC02182 | 2.2129894 | 4.33E-05 | 0.000124945 | Up |
| ATP2A1-AS1 | 2.3426793 | 5.90E-15 | 7.78E-14 | Up |
| AL355607.2 | 6.4515704 | 4.56E-09 | 2.64E-08 | Up |
| C15orf59-AS1 | 3.289281 | 1.41E-05 | 4.46E-05 | Up |
| AC104794.3 | 5.9750839 | 1.89E-11 | 1.57E-10 | Up |
| AC126696.3 | 2.8758595 | 6.29E-08 | 3.09E-07 | Up |
| AC011374.1 | 5.4986906 | 2.85E-19 | 6.08E-18 | Up |
| AL359851.1 | 2.8328925 | 5.81E-06 | 1.98E-05 | Up |
| AC009097.2 | 3.0570116 | 1.04E-13 | 1.17E-12 | Up |
| AC012531.1 | 3.1595705 | 6.48E-09 | 3.66E-08 | Up |
| AC106785.2 | 4.785306 | 1.50E-06 | 5.79E-06 | Up |
| AC020658.3 | 2.5698609 | 3.66E-08 | 1.86E-07 | Up |
| AC138305.1 | 5.2686072 | 6.01E-15 | 7.91E-14 | Up |
| AC004158.1 | 5.0235099 | 9.25E-06 | 3.03E-05 | Up |
| AL445531.1 | 4.0734154 | 4.84E-12 | 4.42E-11 | Up |
| AC120498.4 | 5.6798578 | 1.40E-11 | 1.19E-10 | Up |
| AC093520.1 | 2.7275518 | 3.61E-10 | 2.46E-09 | Up |
| AC106799.3 | 6.8079216 | 4.63E-17 | 7.79E-16 | Up |
| LINC01964 | 4.1305221 | 1.64E-06 | 6.29E-06 | Up |
| AC099506.1 | 2.1202409 | 0.000465317 | 0.001091502 | Up |
| AP005233.2 | 4.9415075 | 3.45E-09 | 2.05E-08 | Up |
| AC015818.2 | 3.0289607 | 1.12E-07 | 5.22E-07 | Up |
| LINC01254 | 3.6437985 | 3.17E-05 | 9.37E-05 | Up |
| AC009139.1 | 3.3547719 | 5.66E-05 | 0.000159239 | Up |
| LINC01416 | 3.7503783 | 1.36E-08 | 7.34E-08 | Up |
| FOXC2-AS1 | 2.3292893 | 0.001574484 | 0.003315113 | Up |
| AP006545.1 | 2.0601019 | 2.21E-09 | 1.34E-08 | Up |
| AC023824.3 | 5.4049133 | 5.98E-06 | 2.04E-05 | Up |
| LINC00557 | 3.4979449 | 1.17E-05 | 3.78E-05 | Up |
| LINC01633 | 7.0624473 | 9.30E-33 | 5.61E-31 | Up |
| AC126407.1 | 4.0749403 | 5.49E-10 | 3.63E-09 | Up |
| LINC01572 | 3.2256972 | 9.16E-42 | 9.03E-40 | Up |
| LINC02544 | 2.2883079 | 5.95E-08 | 2.94E-07 | Up |
| WFDC21P | 2.2179927 | 1.21E-05 | 3.88E-05 | Up |
| LINC01228 | 3.6012012 | 0.000840008 | 0.001876722 | Up |
| LINC02178 | 5.2632906 | 6.44E-10 | 4.23E-09 | Up |
| AC093904.4 | 3.4789096 | 3.24E-09 | 1.93E-08 | Up |
| AL049555.1 | 6.4527971 | 5.41E-48 | 7.67E-46 | Up |
| LINC02473 | 4.4576053 | 1.29E-12 | 1.26E-11 | Up |
| AC009065.5 | 2.0505644 | 1.61E-06 | 6.20E-06 | Up |
| AL137802.2 | 2.0453259 | 8.58E-08 | 4.08E-07 | Up |
| LINC01989 | 2.4309846 | 0.005153877 | 0.009776639 | Up |
| AC112484.3 | 2.361734 | 1.09E-10 | 8.00E-10 | Up |
| LINC02188 | 2.1490229 | 0.000829955 | 0.00185683 | Up |
| AL031058.1 | 3.309152 | 8.73E-27 | 3.38E-25 | Up |
| AC092142.1 | 2.7044152 | 3.08E-11 | 2.48E-10 | Up |
| LINC02128 | 3.9050561 | 1.08E-06 | 4.29E-06 | Up |
| AC112236.1 | 5.3030519 | 2.17E-14 | 2.66E-13 | Up |
| AC122134.1 | 4.2623815 | 2.14E-06 | 8.02E-06 | Up |
| AC106779.1 | 2.2500109 | 1.04E-09 | 6.63E-09 | Up |
| AC009081.1 | 4.7534049 | 4.90E-07 | 2.06E-06 | Up |
| LINC02152 | 4.6928743 | 6.90E-06 | 2.32E-05 | Up |
| AC009061.1 | 2.1979694 | 1.29E-05 | 4.11E-05 | Up |
| AC134312.5 | 2.8083302 | 6.07E-12 | 5.43E-11 | Up |
| AC010491.1 | 2.4370204 | 1.02E-19 | 2.28E-18 | Up |
| VPS9D1-AS1 | 3.826965 | 4.65E-28 | 2.03E-26 | Up |
| AC010735.1 | 2.0113336 | 0.000316148 | 0.00076838 | Up |
| AL591222.1 | 3.7381014 | 1.14E-07 | 5.30E-07 | Up |
| AL035425.3 | 6.0220184 | 8.78E-08 | 4.17E-07 | Up |
| AC021087.4 | 5.3226113 | 8.16E-09 | 4.56E-08 | Up |
| AC013391.3 | 6.1029015 | 3.37E-15 | 4.56E-14 | Up |
| AC040174.1 | 5.4749807 | 2.11E-15 | 2.91E-14 | Up |
| AP003119.3 | 2.4352639 | 9.72E-13 | 9.72E-12 | Up |
| AC091230.1 | 2.3214218 | 0.000862939 | 0.001921578 | Up |
| AC009075.1 | 2.1487807 | 2.55E-05 | 7.67E-05 | Up |
| SSTR5-AS1 | 4.8619788 | 3.34E-07 | 1.45E-06 | Up |
| AL133383.1 | 2.185612 | 0.000884299 | 0.001964813 | Up |
| AC027228.2 | 3.4249893 | 5.95E-23 | 1.80E-21 | Up |
| LINC02582 | 7.1737784 | 2.84E-06 | 1.03E-05 | Up |
| AL033527.3 | 2.2440495 | 6.05E-08 | 2.98E-07 | Up |
| AC007342.4 | 2.0607487 | 3.86E-07 | 1.66E-06 | Up |
| LINC02141 | 5.1966608 | 5.37E-08 | 2.66E-07 | Up |
| AC090282.1 | 5.0726811 | 2.49E-11 | 2.04E-10 | Up |
| SMIM36 | 3.5187947 | 3.24E-08 | 1.67E-07 | Up |
| BCAR4 | 4.4597296 | 6.61E-05 | 0.000184324 | Up |
| AC092115.3 | 3.3287272 | 4.68E-14 | 5.48E-13 | Up |
| AC116025.2 | 2.4405385 | 6.36E-07 | 2.63E-06 | Up |
| AL157931.1 | 2.6372919 | 1.24E-06 | 4.88E-06 | Up |
| AC004034.1 | 2.3379347 | 1.91E-08 | 1.02E-07 | Up |
| AJ003147.1 | 3.9124963 | 2.94E-06 | 1.06E-05 | Up |
| LINC01979 | 2.4481358 | 1.47E-06 | 5.67E-06 | Up |
| AJ003147.2 | 3.8460464 | 2.74E-06 | 9.96E-06 | Up |
| AC005722.2 | 3.403886 | 4.62E-08 | 2.32E-07 | Up |
| AC009121.1 | 3.1262485 | 4.14E-16 | 6.23E-15 | Up |
| AC100791.2 | 4.5690537 | 1.66E-12 | 1.61E-11 | Up |
| LINC01977 | 3.5439071 | 1.97E-14 | 2.43E-13 | Up |
| AC118754.2 | 2.2321593 | 2.12E-05 | 6.47E-05 | Up |
| AC009121.2 | 2.2290896 | 1.87E-08 | 9.92E-08 | Up |
| LINC01896 | 7.6679 | 6.65E-08 | 3.24E-07 | Up |
| AC004477.1 | 2.0190696 | 9.53E-17 | 1.55E-15 | Up |
| TMEM238L | 3.7906798 | 0.000299257 | 0.000730823 | Up |
| AC100872.1 | 2.580805 | 0.000474307 | 0.001110013 | Up |
| AP005328.1 | 3.7039048 | 2.39E-10 | 1.68E-09 | Up |
| AC090125.1 | 5.7707011 | 1.54E-15 | 2.18E-14 | Up |
| AC007639.1 | 3.2853985 | 4.01E-16 | 6.06E-15 | Up |
| AC079062.1 | 7.3073524 | 2.48E-08 | 1.29E-07 | Up |
| AP005230.1 | 5.5359137 | 1.62E-29 | 8.01E-28 | Up |
| AC090371.2 | 2.7574834 | 7.18E-06 | 2.41E-05 | Up |
| LINC01543 | 2.735802 | 1.17E-06 | 4.60E-06 | Up |
| AC080037.1 | 5.385144 | 3.32E-22 | 9.32E-21 | Up |
| AC104564.2 | 2.363644 | 4.98E-07 | 2.09E-06 | Up |
| AC124254.1 | 2.1532719 | 0.000931111 | 0.002058634 | Up |
| LINC02003 | 3.4464322 | 7.66E-10 | 4.99E-09 | Up |
| AC005291.1 | 2.8979589 | 1.95E-05 | 5.99E-05 | Up |
| AC090403.1 | 2.4632184 | 0.001181412 | 0.00256295 | Up |
| AC084346.2 | 2.3002299 | 1.19E-08 | 6.48E-08 | Up |
| AC025211.1 | 3.1551162 | 5.83E-12 | 5.24E-11 | Up |
| AC091170.1 | 3.785946 | 3.99E-05 | 0.000115897 | Up |
| AC104564.4 | 2.2805792 | 0.000158748 | 0.00041051 | Up |
| AC107926.1 | 2.2116881 | 0.002241322 | 0.004578394 | Up |
| AC145207.8 | 2.6233554 | 3.71E-07 | 1.60E-06 | Up |
| AC005722.3 | 4.2934724 | 2.50E-07 | 1.11E-06 | Up |
| AC068025.1 | 2.7343829 | 9.08E-12 | 7.93E-11 | Up |
| AC145343.1 | 2.1617973 | 2.62E-10 | 1.82E-09 | Up |
| AC129926.1 | 2.3340906 | 1.71E-05 | 5.34E-05 | Up |
| AC004147.4 | 2.0353302 | 0.000333761 | 0.00080728 | Up |
| PCAT18 | 4.4762355 | 6.79E-05 | 0.000188895 | Up |
| AC099850.3 | 3.2736052 | 7.33E-33 | 4.45E-31 | Up |
| AC023301.1 | 4.7810429 | 2.17E-16 | 3.40E-15 | Up |
| AP001099.1 | 2.0570752 | 7.12E-06 | 2.39E-05 | Up |
| LINC00668 | 6.858391 | 3.26E-18 | 6.12E-17 | Up |
| AC090912.1 | 2.1732537 | 8.40E-12 | 7.38E-11 | Up |
| GACAT2 | 4.0462734 | 2.63E-11 | 2.15E-10 | Up |
| ESRG | 7.3951057 | 3.74E-07 | 1.61E-06 | Up |
| AC016888.1 | 2.5281521 | 8.17E-15 | 1.06E-13 | Up |
| AP005203.1 | 2.8001819 | 4.49E-05 | 0.000129192 | Up |
| LINC01910 | 3.1364905 | 1.70E-06 | 6.49E-06 | Up |
| LIVAR | 3.1933501 | 1.43E-07 | 6.57E-07 | Up |
| AP002478.1 | 4.1517556 | 4.61E-10 | 3.09E-09 | Up |
| MAGEA10-MAGEA5 | 5.4779572 | 1.93E-13 | 2.12E-12 | Up |
| AP001025.1 | 2.3208485 | 3.03E-05 | 9.00E-05 | Up |
| AC008109.1 | 5.8465991 | 9.17E-08 | 4.35E-07 | Up |
| LINC01887 | 3.5696776 | 2.38E-08 | 1.25E-07 | Up |
| AC024267.6 | 2.0532099 | 4.10E-07 | 1.75E-06 | Up |
| DSG1-AS1 | 6.3836528 | 3.97E-19 | 8.30E-18 | Up |
| AC061975.6 | 5.9881025 | 3.18E-07 | 1.38E-06 | Up |
| AC005993.1 | 5.5650175 | 3.69E-18 | 6.88E-17 | Up |
| LINC01929 | 2.4189699 | 6.57E-06 | 2.22E-05 | Up |
| TCF4-AS1 | 3.1864983 | 5.82E-06 | 1.98E-05 | Up |
| AC010980.2 | 2.4337916 | 6.16E-09 | 3.50E-08 | Up |
| LINC01905 | 3.2254681 | 5.09E-10 | 3.38E-09 | Up |
| AC005256.1 | 6.2656068 | 9.96E-12 | 8.63E-11 | Up |
| LINC02081 | 2.4132785 | 4.19E-09 | 2.44E-08 | Up |
| LINC01842 | 4.0637558 | 2.78E-11 | 2.25E-10 | Up |
| MIR2117HG | 5.3500585 | 4.99E-15 | 6.63E-14 | Up |
| AC022031.1 | 5.7078642 | 1.75E-16 | 2.78E-15 | Up |
| LINC01775 | 2.6944624 | 4.42E-11 | 3.47E-10 | Up |
| AC104971.1 | 2.0109838 | 6.27E-10 | 4.13E-09 | Up |
| AP005264.3 | 2.54909 | 9.59E-05 | 0.000260111 | Up |
| AC011498.3 | 2.3710286 | 7.48E-08 | 3.61E-07 | Up |
| AC020928.2 | 2.8505851 | 3.11E-07 | 1.36E-06 | Up |
| AC022031.2 | 7.9526898 | 9.20E-40 | 8.45E-38 | Up |
| AC068473.3 | 3.3281487 | 2.49E-08 | 1.30E-07 | Up |
| AC008649.1 | 5.6068375 | 1.03E-13 | 1.16E-12 | Up |
| AC021504.1 | 4.6649986 | 1.82E-17 | 3.20E-16 | Up |
| AC009271.1 | 4.7906778 | 1.73E-17 | 3.05E-16 | Up |
| AC005330.1 | 2.4245993 | 4.48E-07 | 1.89E-06 | Up |
| AC016205.1 | 4.5841208 | 1.94E-21 | 4.97E-20 | Up |
| LINC01901 | 4.2431635 | 3.24E-09 | 1.93E-08 | Up |
| AC016168.2 | 2.8741693 | 0.000764334 | 0.00171954 | Up |
| AC010327.3 | 2.5568655 | 0.000243333 | 0.000606717 | Up |
| LINC01926 | 2.253581 | 2.09E-05 | 6.38E-05 | Up |
| AC021683.3 | 4.8626588 | 5.80E-08 | 2.87E-07 | Up |
| AC024592.2 | 2.0407709 | 6.86E-05 | 0.000190479 | Up |
| LINC01539 | 2.7974822 | 3.80E-06 | 1.35E-05 | Up |
| RUNDC3A-AS1 | 3.7810272 | 9.89E-19 | 2.00E-17 | Up |
| AC004221.1 | 4.0808816 | 4.11E-17 | 6.94E-16 | Up |
| AC011483.1 | 2.864343 | 0.000158504 | 0.000410338 | Up |
| AC008750.3 | 2.5982258 | 1.66E-05 | 5.20E-05 | Up |
| AC009955.2 | 2.6924439 | 8.22E-06 | 2.72E-05 | Up |
| AC007785.1 | 5.228406 | 8.65E-12 | 7.59E-11 | Up |
| AC010328.1 | 3.1686779 | 0.000184113 | 0.000469787 | Up |
| AC010605.1 | 3.5322091 | 6.58E-11 | 5.04E-10 | Up |
| AC008687.2 | 2.5387375 | 2.15E-08 | 1.13E-07 | Up |
| LINC02560 | 2.7510802 | 3.25E-10 | 2.22E-09 | Up |
| AL132655.1 | 2.6678835 | 8.76E-07 | 3.54E-06 | Up |
| AC006262.1 | 3.1986706 | 7.02E-11 | 5.34E-10 | Up |
| AC245884.9 | 3.1921739 | 4.01E-06 | 1.42E-05 | Up |
| IGFL2-AS1 | 7.9275891 | 1.29E-17 | 2.29E-16 | Up |
| AL121761.2 | 2.4160717 | 3.97E-07 | 1.70E-06 | Up |
| AP003680.1 | 3.3220052 | 2.77E-18 | 5.29E-17 | Up |
| LINC01711 | 3.1148604 | 1.07E-12 | 1.06E-11 | Up |
| AL356740.2 | 2.5477019 | 4.93E-05 | 0.000140001 | Up |
| AL356740.3 | 2.9447751 | 2.92E-08 | 1.51E-07 | Up |
| LINC01224 | 3.3834644 | 4.49E-06 | 1.57E-05 | Up |
| AC092070.4 | 2.3071177 | 6.96E-06 | 2.34E-05 | Up |
| AC008403.3 | 2.5234672 | 9.44E-10 | 6.07E-09 | Up |
| AC011453.1 | 2.8086866 | 0.002690997 | 0.005400037 | Up |
| AL136172.1 | 2.3988209 | 1.50E-08 | 8.09E-08 | Up |
| AC078802.1 | 2.674416 | 5.17E-11 | 4.02E-10 | Up |
| AC018695.2 | 2.381277 | 4.67E-06 | 1.62E-05 | Up |
| AC091057.4 | 2.2470851 | 2.78E-15 | 3.78E-14 | Up |
| AC040174.2 | 4.3739732 | 1.08E-11 | 9.27E-11 | Up |
| AC036176.3 | 6.5201324 | 3.09E-18 | 5.84E-17 | Up |
| KC877982.1 | 7.7616493 | 3.21E-10 | 2.20E-09 | Up |
| AL513318.2 | 3.8685473 | 2.81E-11 | 2.27E-10 | Up |
| AC027243.2 | 3.7326516 | 3.97E-10 | 2.68E-09 | Up |
| AL133467.4 | 5.6318963 | 4.10E-08 | 2.07E-07 | Up |
| AC125494.3 | 2.6987357 | 5.93E-07 | 2.46E-06 | Up |
| AL162413.1 | 4.7428032 | 1.41E-09 | 8.81E-09 | Up |
| AC106900.2 | 5.6147971 | 1.55E-29 | 7.72E-28 | Up |
| AC013731.1 | 2.4792981 | 1.14E-19 | 2.55E-18 | Up |
| LINC00221 | 4.8608216 | 0.000971683 | 0.002137809 | Up |
| AC010719.1 | 2.1525655 | 1.99E-13 | 2.17E-12 | Up |
| AC015849.5 | 3.5464423 | 1.88E-12 | 1.81E-11 | Up |
| AP000526.1 | 4.6278262 | 4.34E-13 | 4.54E-12 | Up |
| AC090578.1 | 2.9105007 | 1.08E-06 | 4.28E-06 | Up |
| LINC01050 | 2.635758 | 8.74E-08 | 4.16E-07 | Up |
| AL683887.1 | 6.2260762 | 8.25E-17 | 1.35E-15 | Up |
| AC011337.1 | 2.2881462 | 4.58E-12 | 4.21E-11 | Up |
| SMIM32 | 3.3933991 | 2.83E-06 | 1.03E-05 | Up |
| AC012213.4 | 7.1957965 | 5.69E-19 | 1.18E-17 | Up |
| AC073195.1 | 2.3400732 | 2.27E-18 | 4.41E-17 | Up |
| AL096865.1 | 2.0777525 | 1.86E-11 | 1.55E-10 | Up |
| AL589740.1 | 4.0293581 | 4.68E-07 | 1.98E-06 | Up |
| AL136162.1 | 2.6938125 | 3.23E-21 | 8.14E-20 | Up |
| U47924.1 | 2.7824223 | 9.00E-13 | 9.06E-12 | Up |
| AL139424.1 | 2.3979488 | 4.07E-07 | 1.73E-06 | Up |
| AC013400.1 | 2.0056402 | 3.90E-08 | 1.98E-07 | Up |
| AC019080.4 | 4.9804177 | 6.90E-12 | 6.12E-11 | Up |
| AL365181.2 | 4.2365295 | 5.04E-08 | 2.51E-07 | Up |
| AC122710.3 | 2.9842747 | 1.25E-09 | 7.86E-09 | Up |
| LINC01607 | 2.1046293 | 2.12E-10 | 1.50E-09 | Up |
| AL390719.2 | 2.0970298 | 2.34E-10 | 1.65E-09 | Up |
| AF106564.1 | 3.3291743 | 0.000101748 | 0.000274507 | Up |
| AC097358.2 | 3.5532884 | 3.86E-19 | 8.10E-18 | Up |
| AL451050.2 | 2.1494663 | 6.06E-11 | 4.66E-10 | Up |
| AL356277.3 | 3.0177119 | 9.21E-05 | 0.000250612 | Up |
| AC007128.2 | 7.403229 | 2.37E-36 | 1.79E-34 | Up |
| AC116609.3 | 2.913356 | 0.002649407 | 0.005323187 | Up |
| AL160408.5 | 4.6504039 | 2.60E-11 | 2.12E-10 | Up |
| AL365181.3 | 3.795086 | 9.96E-09 | 5.50E-08 | Up |
| BX284668.6 | 2.1030768 | 3.33E-07 | 1.44E-06 | Up |
| AL645608.7 | 2.2084662 | 2.15E-06 | 8.03E-06 | Up |
| AL391244.3 | 2.014715 | 2.17E-20 | 5.07E-19 | Up |
| AC113194.1 | 3.5399845 | 8.84E-06 | 2.90E-05 | Up |
| AP005328.2 | 2.3440866 | 8.68E-05 | 0.000237299 | Up |
| AL021807.1 | 2.1369369 | 9.75E-08 | 4.60E-07 | Up |
| AC017048.3 | 2.4635808 | 0.000142092 | 0.000370522 | Up |
| AFAP1-AS1 | 4.7732654 | 2.34E-08 | 1.22E-07 | Up |
| AC073352.1 | 2.2736799 | 2.73E-11 | 2.21E-10 | Up |
| U62317.2 | 2.5203607 | 6.81E-13 | 6.99E-12 | Up |
| AC010913.1 | 2.4858918 | 1.64E-13 | 1.81E-12 | Up |
| AP005137.2 | 2.4560464 | 5.09E-05 | 0.000144005 | Up |
| AL121658.1 | 2.1068516 | 7.49E-13 | 7.65E-12 | Up |
| AC007881.3 | 2.1132159 | 2.20E-09 | 1.34E-08 | Up |
| AC103702.2 | 5.464887 | 9.78E-14 | 1.10E-12 | Up |
| AP000864.1 | 2.6103836 | 1.97E-08 | 1.04E-07 | Up |
| AL008721.1 | 3.5288857 | 9.64E-08 | 4.55E-07 | Up |
| AC015712.6 | 2.2468346 | 9.14E-07 | 3.68E-06 | Up |
| AC074044.1 | 2.1016649 | 3.38E-09 | 2.01E-08 | Up |
| AP000525.1 | 2.1512731 | 3.40E-08 | 1.74E-07 | Up |
| AC113189.4 | 3.1221684 | 1.27E-11 | 1.08E-10 | Up |
| AL353708.3 | 2.0154248 | 1.38E-15 | 1.96E-14 | Up |
| AL022324.3 | 3.284601 | 9.78E-09 | 5.42E-08 | Up |
| AP000553.2 | 2.1230461 | 2.57E-08 | 1.34E-07 | Up |
| LINC02012 | 3.47426 | 2.50E-13 | 2.68E-12 | Up |
| DGCR9 | 2.3749251 | 6.94E-09 | 3.92E-08 | Up |
| AC007250.1 | 2.5965405 | 6.80E-06 | 2.29E-05 | Up |
| AC017083.1 | 2.194362 | 1.30E-14 | 1.63E-13 | Up |
| AC093865.1 | 2.9994989 | 4.60E-08 | 2.31E-07 | Up |
| AC007663.3 | 2.0151679 | 4.17E-08 | 2.11E-07 | Up |
| AL133215.2 | 3.1460037 | 5.43E-30 | 2.79E-28 | Up |
| AC006946.2 | 2.2824639 | 6.21E-06 | 2.11E-05 | Up |
| AC090912.3 | 2.6165274 | 2.19E-06 | 8.18E-06 | Up |
| AC116565.1 | 4.1508648 | 2.09E-05 | 6.38E-05 | Up |
| AC010997.5 | 2.2004155 | 4.28E-08 | 2.16E-07 | Up |
| AL008718.2 | 2.1035026 | 2.58E-09 | 1.56E-08 | Up |
| AC009275.1 | 2.2134475 | 4.30E-08 | 2.17E-07 | Up |
| RBAKDN | 3.141866 | 7.90E-06 | 2.62E-05 | Up |
| Z83851.2 | 2.361375 | 1.74E-17 | 3.06E-16 | Up |
| AL355472.4 | 3.0210001 | 3.00E-10 | 2.06E-09 | Up |
| AC069222.1 | 2.054205 | 9.25E-11 | 6.87E-10 | Up |
| AC005291.2 | 2.7378788 | 1.12E-05 | 3.62E-05 | Up |
| AC083880.1 | 2.3577954 | 2.96E-15 | 4.02E-14 | Up |
| AP000904.1 | 2.8133674 | 0.00077576 | 0.001741366 | Up |
| AC006946.3 | 2.2236227 | 7.06E-05 | 0.000195437 | Up |
| AL645608.9 | 2.0418922 | 4.00E-06 | 1.41E-05 | Up |
| AL391069.4 | 2.6430832 | 2.88E-06 | 1.05E-05 | Up |
| LINC02348 | 2.5993435 | 2.04E-06 | 7.69E-06 | Up |
| AC020658.4 | 4.0706991 | 3.36E-14 | 4.02E-13 | Up |
| AL109954.2 | 3.0313919 | 3.83E-06 | 1.36E-05 | Up |
| AC023158.2 | 3.0971614 | 6.90E-05 | 0.000191498 | Up |
| FRMD6-AS1 | 2.4251978 | 2.62E-16 | 4.06E-15 | Up |
| AC022079.1 | 2.9992731 | 4.50E-08 | 2.26E-07 | Up |
| AL049794.1 | 2.5796351 | 2.90E-12 | 2.74E-11 | Up |
| AC022929.2 | 2.0067367 | 0.000350124 | 0.000844331 | Up |
| AC090970.3 | 2.1260653 | 1.32E-11 | 1.12E-10 | Up |
| AL355338.1 | 2.3235687 | 6.63E-22 | 1.80E-20 | Up |
| AC025166.1 | 3.888597 | 7.97E-20 | 1.80E-18 | Up |
| AL023803.2 | 2.5201005 | 2.09E-16 | 3.28E-15 | Up |
| LINC01297 | 5.9018047 | 4.02E-07 | 1.72E-06 | Up |
| AC132807.2 | 4.4580785 | 4.38E-06 | 1.53E-05 | Up |
| AC011700.1 | 3.4669843 | 7.87E-07 | 3.20E-06 | Up |
| AC087588.2 | 2.7355539 | 1.31E-08 | 7.12E-08 | Up |
| AL031710.2 | 2.0167059 | 4.69E-10 | 3.14E-09 | Up |
| AL161431.1 | 5.9060378 | 3.72E-11 | 2.96E-10 | Up |
| AC018695.6 | 2.9969395 | 1.33E-12 | 1.29E-11 | Up |
| AL121832.3 | 2.1240535 | 1.23E-14 | 1.56E-13 | Up |
| AL049539.1 | 3.043017 | 2.53E-14 | 3.08E-13 | Up |
| AL121827.2 | 2.8340987 | 2.46E-07 | 1.09E-06 | Up |
| AL139385.1 | 2.9232802 | 2.27E-16 | 3.54E-15 | Up |
| AC110285.6 | 2.2353019 | 3.21E-07 | 1.39E-06 | Up |
| AC026336.3 | 5.2161024 | 1.11E-08 | 6.09E-08 | Up |
| AC009118.2 | 3.19339 | 2.38E-15 | 3.27E-14 | Up |
| AC209154.2 | 6.5642284 | 4.17E-06 | 1.46E-05 | Up |
| AL136221.1 | 2.9968805 | 1.41E-07 | 6.51E-07 | Up |
| AC137834.2 | 2.5621189 | 2.20E-10 | 1.55E-09 | Up |
| AC105137.2 | 2.2348571 | 1.66E-15 | 2.33E-14 | Up |
| AC008556.1 | 2.2789534 | 4.69E-09 | 2.71E-08 | Up |
| AL122125.1 | 2.4043132 | 4.25E-13 | 4.44E-12 | Up |
| AL589743.5 | 2.6813837 | 7.37E-06 | 2.46E-05 | Up |
| AC006449.5 | 2.001898 | 4.94E-13 | 5.13E-12 | Up |
| AC083809.1 | 3.3873052 | 2.43E-06 | 8.95E-06 | Up |
| LHX1-DT | 4.9435265 | 5.09E-06 | 1.75E-05 | Up |
| AL160412.1 | 4.00307 | 4.27E-06 | 1.49E-05 | Up |
| AP001065.2 | 2.9253368 | 0.001383118 | 0.002960005 | Up |
| AC010331.1 | 2.0654618 | 1.58E-11 | 1.33E-10 | Up |
| AC010271.2 | 2.5441199 | 3.10E-07 | 1.35E-06 | Up |
| AC007996.1 | 2.173428 | 4.00E-12 | 3.70E-11 | Up |
| AL023803.3 | 3.1412115 | 1.10E-10 | 8.10E-10 | Up |
| AC008406.3 | 5.6473659 | 2.58E-17 | 4.49E-16 | Up |
| AP003900.1 | 6.1659846 | 1.42E-06 | 5.50E-06 | Up |
| AC107308.1 | 4.622227 | 1.09E-09 | 6.94E-09 | Up |
| AL133325.3 | 6.0893336 | 2.03E-10 | 1.44E-09 | Up |
| AC036108.4 | 2.5523508 | 5.68E-09 | 3.24E-08 | Up |
| AL118505.1 | 2.8476327 | 2.03E-15 | 2.82E-14 | Up |
| LINC01971 | 3.3409178 | 3.42E-11 | 2.73E-10 | Up |
| AL359513.1 | 2.7782549 | 2.29E-20 | 5.36E-19 | Up |
| AC068831.6 | 5.085555 | 1.14E-24 | 3.82E-23 | Up |
| AL161645.1 | 2.0814487 | 0.000255391 | 0.00063191 | Up |
| AC023310.4 | 5.0594533 | 1.46E-08 | 7.89E-08 | Up |
| AL162574.2 | 3.1435692 | 2.98E-05 | 8.86E-05 | Up |
| AC133540.1 | 2.1123238 | 0.000606043 | 0.001392108 | Up |
| AL590226.2 | 4.3439474 | 4.92E-17 | 8.23E-16 | Up |
| AC007383.4 | 4.055879 | 0.001215203 | 0.002629902 | Up |
| CR392039.3 | 3.8225433 | 3.73E-05 | 0.000109037 | Up |
| FAM230C | 6.644244 | 4.58E-06 | 1.59E-05 | Up |
| LINC01666 | 2.9774329 | 4.75E-05 | 0.000135601 | Up |
| AL358613.2 | 2.539534 | 0.000203694 | 0.000514712 | Up |
| FP325330.3 | 6.7809398 | 1.80E-22 | 5.19E-21 | Up |
| AC022784.8 | 2.6174609 | 1.10E-07 | 5.16E-07 | Up |
| CU634019.6 | 2.2138951 | 1.68E-07 | 7.66E-07 | Up |
| LINC01667 | 6.8684199 | 7.44E-06 | 2.49E-05 | Up |
| CU639417.4 | 3.1895789 | 4.49E-11 | 3.52E-10 | Up |
| AL021877.2 | 6.1613073 | 8.80E-11 | 6.59E-10 | Up |
| SH3PXD2A-AS1 | 4.1053415 | 6.75E-12 | 6.01E-11 | Up |
| PCAT5 | 3.2100226 | 4.58E-05 | 0.000131169 | Up |
| LINC01202 | 5.7630267 | 1.20E-09 | 7.56E-09 | Up |
| ELDR | 5.2813456 | 1.95E-08 | 1.03E-07 | Up |
| FOXCUT | 2.7056679 | 2.80E-07 | 1.23E-06 | Up |
| AP003500.1 | 3.3100294 | 2.70E-06 | 9.84E-06 | Up |
| LINC01127 | 2.7104102 | 5.37E-06 | 1.84E-05 | Up |
| AL590637.1 | 2.1714047 | 0.000165128 | 0.000425105 | Up |
| GHET1 | 2.1789088 | 3.15E-17 | 5.42E-16 | Up |
| AP003500.2 | 2.7357241 | 5.60E-05 | 0.000157573 | Up |
| SNHG4 | 2.4503089 | 1.21E-21 | 3.18E-20 | Up |
| BLACAT1 | 2.8250958 | 9.68E-11 | 7.17E-10 | Up |
| SAMD12-AS1 | 2.2929962 | 9.97E-08 | 4.69E-07 | Up |
| AP000851.2 | 4.6442391 | 3.46E-06 | 1.24E-05 | Up |
| AL009179.1 | 2.0170982 | 2.60E-14 | 3.15E-13 | Up |

**Supplementary table 2: Differentially expressed miRNAs in smoking LUSC**

| **miRNA** | **logFC** | **p-value** | **FDR** | **regulate** |
| --- | --- | --- | --- | --- |
| hsa-mir-1-1 | -2.303395 | 4.26E-19 | 5.24E-18 | Down |
| hsa-mir-1-2 | -2.345376 | 2.78E-19 | 3.56E-18 | Down |
| hsa-mir-101-1 | -2.279825 | 1.19E-61 | 1.76E-59 | Down |
| hsa-mir-101-2 | -2.283966 | 8.42E-62 | 1.66E-59 | Down |
| hsa-mir-1258 | -2.029778 | 1.28E-10 | 7.41E-10 | Down |
| hsa-mir-133a-1 | -2.933358 | 3.01E-30 | 8.46E-29 | Down |
| hsa-mir-133a-2 | -2.790306 | 2.15E-29 | 5.71E-28 | Down |
| hsa-mir-133b | -3.224325 | 1.15E-25 | 2.42E-24 | Down |
| hsa-mir-135a-2 | -2.007705 | 1.69E-06 | 5.36E-06 | Down |
| hsa-mir-139 | -2.662355 | 1.26E-42 | 6.75E-41 | Down |
| hsa-mir-140 | -2.107721 | 2.72E-44 | 2.00E-42 | Down |
| hsa-mir-144 | -3.091517 | 3.96E-41 | 1.79E-39 | Down |
| hsa-mir-218-1 | -2.259177 | 6.10E-43 | 3.60E-41 | Down |
| hsa-mir-218-2 | -2.278581 | 1.35E-46 | 1.13E-44 | Down |
| hsa-mir-3065 | -2.07585 | 6.67E-20 | 9.37E-19 | Down |
| hsa-mir-30a | -3.06473 | 4.54E-84 | 2.68E-81 | Down |
| hsa-mir-30d | -2.479313 | 1.29E-75 | 3.81E-73 | Down |
| hsa-mir-326 | -2.405521 | 1.94E-22 | 3.36E-21 | Down |
| hsa-mir-338 | -2.709591 | 1.06E-41 | 5.23E-40 | Down |
| hsa-mir-451a | -3.101656 | 8.09E-39 | 3.18E-37 | Down |
| hsa-mir-4732 | -2.963331 | 5.67E-23 | 1.01E-21 | Down |
| hsa-mir-4777 | -2.339729 | 1.06E-31 | 3.48E-30 | Down |
| hsa-mir-486-1 | -3.47644 | 3.43E-48 | 3.37E-46 | Down |
| hsa-mir-486-2 | -3.484329 | 2.12E-48 | 2.50E-46 | Down |
| hsa-mir-490 | -3.051542 | 1.13E-10 | 6.62E-10 | Down |
| hsa-mir-105-1 | 8.5066242 | 1.55E-14 | 1.48E-13 | Up |
| hsa-mir-105-2 | 8.3603444 | 6.10E-14 | 5.37E-13 | Up |
| hsa-mir-1224 | 2.1510216 | 0.0017265 | 0.0034298 | Up |
| hsa-mir-1248 | 3.2168227 | 9.86E-10 | 5.15E-09 | Up |
| hsa-mir-1254-1 | 2.5170095 | 4.51E-07 | 1.59E-06 | Up |
| hsa-mir-1254-2 | 2.2098846 | 2.38E-05 | 6.31E-05 | Up |
| hsa-mir-1269a | 8.5243182 | 9.53E-19 | 1.15E-17 | Up |
| hsa-mir-1269b | 8.4455158 | 5.99E-08 | 2.44E-07 | Up |
| hsa-mir-1277 | 2.0179129 | 2.26E-12 | 1.67E-11 | Up |
| hsa-mir-129-1 | 3.48616 | 2.69E-06 | 8.22E-06 | Up |
| hsa-mir-129-2 | 3.2779124 | 8.78E-06 | 2.41E-05 | Up |
| hsa-mir-1293 | 5.6433032 | 5.84E-14 | 5.22E-13 | Up |
| hsa-mir-1295b | 2.0869118 | 0.0052238 | 0.009368 | Up |
| hsa-mir-130b | 2.6824817 | 9.79E-27 | 2.31E-25 | Up |
| hsa-mir-135b | 2.3605438 | 2.61E-09 | 1.32E-08 | Up |
| hsa-mir-137 | 4.5212366 | 4.28E-07 | 1.52E-06 | Up |
| hsa-mir-147b | 3.4930118 | 5.21E-13 | 4.16E-12 | Up |
| hsa-mir-149 | 3.1014358 | 1.73E-15 | 1.79E-14 | Up |
| hsa-mir-182 | 2.4024732 | 2.93E-16 | 3.09E-15 | Up |
| hsa-mir-183 | 3.1425322 | 2.93E-25 | 5.95E-24 | Up |
| hsa-mir-1910 | 4.1632281 | 1.49E-14 | 1.44E-13 | Up |
| hsa-mir-1911 | 4.673439 | 5.24E-06 | 1.49E-05 | Up |
| hsa-mir-193b | 2.0613454 | 5.05E-11 | 3.13E-10 | Up |
| hsa-mir-196a-1 | 5.7841146 | 9.03E-14 | 7.72E-13 | Up |
| hsa-mir-196a-2 | 5.5365157 | 5.52E-13 | 4.34E-12 | Up |
| hsa-mir-196b | 5.3750981 | 2.18E-24 | 4.29E-23 | Up |
| hsa-mir-203a | 2.1343137 | 2.74E-07 | 1.01E-06 | Up |
| hsa-mir-205 | 7.1180934 | 1.65E-39 | 6.97E-38 | Up |
| hsa-mir-210 | 4.8467001 | 2.74E-43 | 1.80E-41 | Up |
| hsa-mir-224 | 3.0268138 | 1.09E-11 | 7.37E-11 | Up |
| hsa-mir-301b | 4.0072732 | 4.55E-20 | 6.55E-19 | Up |
| hsa-mir-31 | 5.2357774 | 4.32E-14 | 3.92E-13 | Up |
| hsa-mir-3176 | 2.1525808 | 1.38E-06 | 4.51E-06 | Up |
| hsa-mir-3200 | 2.4682914 | 5.17E-09 | 2.50E-08 | Up |
| hsa-mir-323a | 3.2902758 | 4.21E-07 | 1.51E-06 | Up |
| hsa-mir-323b | 4.0365981 | 9.59E-10 | 5.05E-09 | Up |
| hsa-mir-33a | 2.0732342 | 7.97E-11 | 4.80E-10 | Up |
| hsa-mir-33b | 2.3359579 | 3.68E-11 | 2.34E-10 | Up |
| hsa-mir-345 | 2.4440158 | 1.55E-17 | 1.75E-16 | Up |
| hsa-mir-3609 | 2.233583 | 0.0004652 | 0.0010127 | Up |
| hsa-mir-3619 | 2.3075258 | 5.28E-06 | 1.49E-05 | Up |
| hsa-mir-3651 | 2.0858408 | 5.53E-08 | 2.28E-07 | Up |
| hsa-mir-3662 | 4.3961454 | 8.16E-13 | 6.10E-12 | Up |
| hsa-mir-412 | 2.5725913 | 4.14E-05 | 0.0001057 | Up |
| hsa-mir-4449 | 3.407869 | 4.08E-09 | 1.99E-08 | Up |
| hsa-mir-4491 | 2.4783298 | 1.07E-06 | 3.56E-06 | Up |
| hsa-mir-4640 | 2.0575153 | 1.88E-06 | 5.88E-06 | Up |
| hsa-mir-4652 | 7.0660684 | 2.23E-29 | 5.71E-28 | Up |
| hsa-mir-4664 | 2.8861055 | 4.98E-10 | 2.70E-09 | Up |
| hsa-mir-4665 | 2.8673737 | 3.24E-07 | 1.19E-06 | Up |
| hsa-mir-4668 | 2.0563992 | 2.71E-16 | 2.90E-15 | Up |
| hsa-mir-4687 | 2.752031 | 6.57E-08 | 2.65E-07 | Up |
| hsa-mir-4713 | 3.7244419 | 4.12E-08 | 1.77E-07 | Up |
| hsa-mir-4766 | 3.2185348 | 1.30E-11 | 8.73E-11 | Up |
| hsa-mir-4778 | 4.5256249 | 5.41E-09 | 2.57E-08 | Up |
| hsa-mir-503 | 2.3163828 | 1.17E-13 | 9.70E-13 | Up |
| hsa-mir-509-1 | 2.4317753 | 0.0008447 | 0.0017735 | Up |
| hsa-mir-509-2 | 2.4030672 | 0.0007119 | 0.0015163 | Up |
| hsa-mir-509-3 | 2.4096983 | 0.0006275 | 0.0013463 | Up |
| hsa-mir-5092 | 3.7508995 | 1.63E-07 | 6.36E-07 | Up |
| hsa-mir-516a-1 | 4.0231839 | 0.0017707 | 0.0035057 | Up |
| hsa-mir-516a-2 | 3.6100469 | 0.0037267 | 0.0068926 | Up |
| hsa-mir-519a-1 | 5.563296 | 0.0001251 | 0.0002964 | Up |
| hsa-mir-519a-2 | 5.8841997 | 0.0001486 | 0.0003479 | Up |
| hsa-mir-520a | 7.0107044 | 0.0001453 | 0.0003428 | Up |
| hsa-mir-522 | 5.272552 | 0.0008956 | 0.0018544 | Up |
| hsa-mir-526b | 7.9556124 | 3.48E-05 | 9.00E-05 | Up |
| hsa-mir-541 | 2.4426493 | 0.0026591 | 0.0051271 | Up |
| hsa-mir-545 | 2.3403101 | 1.59E-10 | 9.05E-10 | Up |
| hsa-mir-548f-1 | 4.6495404 | 5.23E-06 | 1.49E-05 | Up |
| hsa-mir-556 | 2.4672991 | 4.72E-07 | 1.66E-06 | Up |
| hsa-mir-5571 | 2.5154451 | 3.36E-07 | 1.23E-06 | Up |
| hsa-mir-5579 | 3.1837524 | 4.64E-08 | 1.94E-07 | Up |
| hsa-mir-570 | 2.2548454 | 5.35E-08 | 2.22E-07 | Up |
| hsa-mir-573 | 3.6207958 | 1.79E-06 | 5.65E-06 | Up |
| hsa-mir-577 | 5.2202588 | 2.17E-13 | 1.78E-12 | Up |
| hsa-mir-579 | 2.0221245 | 7.19E-09 | 3.34E-08 | Up |
| hsa-mir-615 | 3.9441274 | 4.47E-10 | 2.44E-09 | Up |
| hsa-mir-616 | 2.6442981 | 1.34E-14 | 1.31E-13 | Up |
| hsa-mir-628 | 2.0965775 | 1.08E-07 | 4.30E-07 | Up |
| hsa-mir-6499 | 6.0661263 | 4.37E-20 | 6.44E-19 | Up |
| hsa-mir-6510 | 4.3173512 | 5.57E-11 | 3.42E-10 | Up |
| hsa-mir-6512 | 3.2483346 | 6.07E-07 | 2.11E-06 | Up |
| hsa-mir-6728 | 2.3909117 | 8.38E-06 | 2.31E-05 | Up |
| hsa-mir-675 | 3.5006043 | 1.85E-05 | 4.96E-05 | Up |
| hsa-mir-7-3 | 2.9812886 | 2.64E-07 | 9.81E-07 | Up |
| hsa-mir-708 | 3.1061311 | 1.16E-23 | 2.21E-22 | Up |
| hsa-mir-7112 | 2.782522 | 8.57E-07 | 2.89E-06 | Up |
| hsa-mir-760 | 2.4198144 | 1.79E-10 | 1.01E-09 | Up |
| hsa-mir-7641-1 | 3.4546689 | 5.30E-06 | 1.49E-05 | Up |
| hsa-mir-767 | 7.9371582 | 2.83E-14 | 2.61E-13 | Up |
| hsa-mir-7974 | 3.9819446 | 5.51E-09 | 2.60E-08 | Up |
| hsa-mir-873 | 2.9355945 | 0.0002094 | 0.0004825 | Up |
| hsa-mir-877 | 2.269423 | 5.95E-11 | 3.62E-10 | Up |
| hsa-mir-891a | 4.0909971 | 1.60E-06 | 5.11E-06 | Up |
| hsa-mir-9-1 | 5.9002196 | 2.53E-19 | 3.40E-18 | Up |
| hsa-mir-9-2 | 5.9283496 | 1.92E-19 | 2.64E-18 | Up |
| hsa-mir-9-3 | 5.9134472 | 2.74E-19 | 3.56E-18 | Up |
| hsa-mir-944 | 6.5962973 | 6.81E-29 | 1.67E-27 | Up |
| hsa-mir-96 | 3.013737 | 5.84E-21 | 9.00E-20 | Up |

**Supplementary table 3: Differentially expressed mRNAs in smoking LUSC**

| **mRNA** | **logFC** | **p-value** | **FDR** | **regulate** |
| --- | --- | --- | --- | --- |
| FGR | -2.254833 | 5.14E-37 | 1.53E-35 | Down |
| ENPP4 | -2.13113 | 1.21E-25 | 1.96E-24 | Down |
| CFTR | -2.374983 | 7.80E-16 | 5.84E-15 | Down |
| HSPB6 | -3.48714 | 2.77E-61 | 2.46E-59 | Down |
| PDK4 | -3.597759 | 8.98E-46 | 3.90E-44 | Down |
| ZMYND10 | -2.754765 | 1.94E-18 | 1.86E-17 | Down |
| SLC4A1 | -2.526266 | 4.77E-13 | 2.70E-12 | Down |
| ALDH3B1 | -3.176621 | 1.08E-68 | 1.29E-66 | Down |
| ARHGAP44 | -2.797407 | 1.21E-48 | 5.87E-47 | Down |
| CEACAM21 | -2.284025 | 2.16E-29 | 4.39E-28 | Down |
| DNAH9 | -3.194995 | 1.86E-23 | 2.64E-22 | Down |
| CACNA2D2 | -4.38662 | 7.39E-73 | 1.11E-70 | Down |
| SELE | -2.332644 | 2.03E-11 | 9.70E-11 | Down |
| PGLYRP1 | -2.732172 | 5.98E-30 | 1.25E-28 | Down |
| RHOBTB2 | -2.513582 | 2.20E-39 | 7.35E-38 | Down |
| SEMA3G | -3.380211 | 3.56E-103 | 2.15E-100 | Down |
| ANOS1 | -2.982539 | 1.07E-46 | 4.85E-45 | Down |
| SEMA3B | -2.788073 | 6.15E-35 | 1.64E-33 | Down |
| ALOX5 | -2.715223 | 2.01E-47 | 9.32E-46 | Down |
| GPRC5A | -2.870536 | 1.20E-33 | 3.01E-32 | Down |
| SLC11A1 | -2.389417 | 5.91E-43 | 2.28E-41 | Down |
| ATP1A2 | -3.027626 | 1.14E-20 | 1.30E-19 | Down |
| VSIG2 | -2.849064 | 1.72E-19 | 1.81E-18 | Down |
| MARCO | -4.101128 | 1.82E-58 | 1.40E-56 | Down |
| HGF | -2.087835 | 1.25E-16 | 1.02E-15 | Down |
| C8B | -5.143358 | 1.12E-44 | 4.54E-43 | Down |
| FHL1 | -3.919276 | 2.78E-94 | 1.23E-91 | Down |
| HSD17B6 | -3.959348 | 5.13E-113 | 4.88E-110 | Down |
| MUSK | -2.640806 | 8.26E-28 | 1.52E-26 | Down |
| ARHGAP31 | -2.648127 | 4.68E-63 | 4.47E-61 | Down |
| MYOC | -3.240274 | 6.33E-10 | 2.54E-09 | Down |
| DAPK2 | -2.63612 | 2.31E-54 | 1.49E-52 | Down |
| OTC | -4.879513 | 9.59E-58 | 7.16E-56 | Down |
| FLT4 | -2.253908 | 2.26E-47 | 1.04E-45 | Down |
| MSR1 | -3.003447 | 1.54E-48 | 7.43E-47 | Down |
| C6 | -2.949887 | 1.39E-14 | 9.17E-14 | Down |
| ADRB1 | -3.310394 | 1.43E-38 | 4.60E-37 | Down |
| PREX2 | -3.183625 | 1.16E-61 | 1.04E-59 | Down |
| ARHGAP6 | -3.067164 | 4.77E-56 | 3.30E-54 | Down |
| ROS1 | -2.366973 | 3.85E-13 | 2.20E-12 | Down |
| LMO3 | -2.47242 | 1.05E-13 | 6.39E-13 | Down |
| CELF2 | -2.437678 | 1.44E-38 | 4.64E-37 | Down |
| LAMC3 | -2.079889 | 3.35E-25 | 5.29E-24 | Down |
| TNIP3 | -2.055012 | 5.28E-13 | 2.97E-12 | Down |
| CBLN4 | -2.682898 | 2.25E-15 | 1.61E-14 | Down |
| CCDC85A | -3.301961 | 1.64E-51 | 9.21E-50 | Down |
| RASGRF1 | -3.597675 | 5.06E-40 | 1.75E-38 | Down |
| LIMCH1 | -3.34655 | 3.44E-66 | 3.61E-64 | Down |
| WISP2 | -3.115055 | 2.95E-41 | 1.07E-39 | Down |
| CHI3L2 | -2.980876 | 1.99E-24 | 2.98E-23 | Down |
| CALCRL | -2.817307 | 1.08E-68 | 1.29E-66 | Down |
| SLC9A3R2 | -2.175557 | 1.83E-56 | 1.28E-54 | Down |
| GLP2R | -3.50403 | 2.11E-29 | 4.29E-28 | Down |
| TIE1 | -2.635437 | 7.15E-69 | 8.73E-67 | Down |
| SPI1 | -2.024418 | 2.19E-31 | 4.96E-30 | Down |
| CLDN18 | -5.770279 | 4.14E-69 | 5.20E-67 | Down |
| ATP11A | -2.1618 | 1.10E-38 | 3.58E-37 | Down |
| PYGM | -2.241746 | 1.45E-38 | 4.64E-37 | Down |
| ADGRF5 | -3.622294 | 2.69E-92 | 1.01E-89 | Down |
| MAOB | -2.167463 | 5.49E-25 | 8.49E-24 | Down |
| TGFBR3 | -2.064443 | 1.02E-21 | 1.27E-20 | Down |
| PTPN21 | -2.772791 | 2.13E-96 | 1.01E-93 | Down |
| RPS6KA2 | -2.403964 | 8.71E-49 | 4.28E-47 | Down |
| CYBRD1 | -2.321717 | 8.48E-55 | 5.57E-53 | Down |
| LIMS2 | -2.903929 | 5.82E-80 | 1.22E-77 | Down |
| TRHDE | -3.26162 | 3.51E-15 | 2.47E-14 | Down |
| CD5L | -3.845067 | 5.54E-25 | 8.54E-24 | Down |
| FRY | -2.475595 | 2.19E-46 | 9.68E-45 | Down |
| MGLL | -2.130372 | 5.15E-30 | 1.08E-28 | Down |
| NTN4 | -2.269133 | 4.52E-30 | 9.52E-29 | Down |
| SCARF1 | -2.105199 | 4.16E-48 | 1.98E-46 | Down |
| NMRK2 | -2.265449 | 1.36E-10 | 5.88E-10 | Down |
| SPAG6 | -2.526972 | 4.98E-11 | 2.28E-10 | Down |
| ACTN2 | -2.73976 | 9.19E-17 | 7.56E-16 | Down |
| ITGA8 | -3.410131 | 2.89E-71 | 3.95E-69 | Down |
| LAMP3 | -2.788559 | 3.67E-38 | 1.15E-36 | Down |
| ITM2A | -2.331348 | 4.04E-30 | 8.56E-29 | Down |
| TNS1 | -2.993198 | 5.23E-93 | 2.06E-90 | Down |
| SCTR | -2.473617 | 2.05E-16 | 1.62E-15 | Down |
| SLC4A4 | -2.879354 | 3.58E-28 | 6.74E-27 | Down |
| PIH1D3 | -2.728639 | 7.27E-09 | 2.58E-08 | Down |
| CPB2 | -4.491357 | 1.62E-29 | 3.33E-28 | Down |
| CXCL2 | -3.024351 | 2.63E-29 | 5.30E-28 | Down |
| COL4A4 | -2.770804 | 2.44E-29 | 4.92E-28 | Down |
| CACNA1S | -2.889512 | 1.41E-13 | 8.48E-13 | Down |
| PGR | -2.80809 | 4.36E-42 | 1.63E-40 | Down |
| EFR3B | -2.189334 | 4.38E-18 | 4.06E-17 | Down |
| FCN1 | -3.089226 | 5.76E-42 | 2.14E-40 | Down |
| ABCB1 | -2.197148 | 7.43E-23 | 1.01E-21 | Down |
| CASS4 | -2.838062 | 9.50E-52 | 5.40E-50 | Down |
| F11 | -4.886323 | 2.97E-52 | 1.73E-50 | Down |
| CFAP61 | -2.245359 | 2.16E-14 | 1.40E-13 | Down |
| TBX5 | -2.041817 | 3.35E-29 | 6.69E-28 | Down |
| ICAM1 | -2.449518 | 1.31E-28 | 2.53E-27 | Down |
| LYZ | -2.175989 | 1.41E-17 | 1.25E-16 | Down |
| NLRC4 | -2.821873 | 1.08E-80 | 2.33E-78 | Down |
| IL5RA | -2.686213 | 1.43E-22 | 1.90E-21 | Down |
| ABCC6 | -2.250955 | 3.23E-31 | 7.26E-30 | Down |
| APOH | -3.085152 | 8.28E-11 | 3.69E-10 | Down |
| CPA1 | -2.050819 | 1.25E-08 | 4.33E-08 | Down |
| CMA1 | -2.552668 | 5.81E-17 | 4.88E-16 | Down |
| JPH4 | -2.402823 | 2.60E-20 | 2.87E-19 | Down |
| TEKT2 | -2.345028 | 2.42E-14 | 1.56E-13 | Down |
| DPYSL2 | -2.776984 | 1.20E-84 | 3.15E-82 | Down |
| FMO2 | -3.082731 | 3.05E-33 | 7.46E-32 | Down |
| SH2D3C | -2.797411 | 1.28E-86 | 3.56E-84 | Down |
| SORBS1 | -2.651316 | 2.21E-55 | 1.48E-53 | Down |
| CRTAC1 | -2.897258 | 3.39E-20 | 3.72E-19 | Down |
| TPSD1 | -2.031308 | 1.41E-08 | 4.84E-08 | Down |
| PGC | -4.01937 | 5.92E-24 | 8.66E-23 | Down |
| IGFALS | -2.700359 | 9.29E-26 | 1.51E-24 | Down |
| GADD45B | -2.23971 | 1.12E-37 | 3.44E-36 | Down |
| SERPIND1 | -2.076619 | 4.15E-07 | 1.19E-06 | Down |
| SUSD2 | -4.437458 | 8.15E-81 | 1.77E-78 | Down |
| SEC14L3 | -4.861722 | 3.57E-49 | 1.79E-47 | Down |
| MFNG | -2.033382 | 3.18E-30 | 6.77E-29 | Down |
| GGTLC2 | -4.070815 | 8.15E-52 | 4.66E-50 | Down |
| SLC5A4 | -2.473582 | 4.39E-22 | 5.61E-21 | Down |
| CTSG | -2.545715 | 1.32E-18 | 1.28E-17 | Down |
| GALNT16 | -2.024033 | 4.25E-11 | 1.96E-10 | Down |
| SLC8A3 | -2.199165 | 3.96E-12 | 2.04E-11 | Down |
| TUBB1 | -3.714268 | 6.50E-102 | 3.79E-99 | Down |
| SPEF1 | -2.026363 | 6.82E-12 | 3.42E-11 | Down |
| SEL1L2 | -2.060971 | 1.56E-07 | 4.72E-07 | Down |
| ANGPT4 | -4.618096 | 1.72E-89 | 5.36E-87 | Down |
| RSPO4 | -2.098821 | 2.36E-10 | 9.91E-10 | Down |
| SIRPB1 | -3.307266 | 9.22E-54 | 5.77E-52 | Down |
| CCM2L | -2.621997 | 1.05E-48 | 5.13E-47 | Down |
| EPPIN | -2.657167 | 8.48E-09 | 3.00E-08 | Down |
| TLR8 | -2.206528 | 1.07E-19 | 1.14E-18 | Down |
| CHRDL1 | -3.663235 | 9.30E-41 | 3.30E-39 | Down |
| RS1 | -4.895716 | 1.25E-81 | 2.83E-79 | Down |
| GATA1 | -2.416226 | 1.18E-39 | 4.01E-38 | Down |
| CD40LG | -2.042842 | 3.90E-14 | 2.46E-13 | Down |
| FGF9 | -2.123089 | 2.12E-10 | 8.97E-10 | Down |
| SGCG | -2.348139 | 7.93E-11 | 3.54E-10 | Down |
| RGCC | -3.410693 | 8.01E-124 | 1.81E-120 | Down |
| MSLN | -2.215313 | 2.88E-09 | 1.07E-08 | Down |
| PLLP | -2.070802 | 9.57E-34 | 2.40E-32 | Down |
| WFDC1 | -2.535738 | 4.24E-45 | 1.78E-43 | Down |
| FOXF1 | -2.781042 | 7.53E-56 | 5.19E-54 | Down |
| AQP9 | -2.21437 | 2.67E-19 | 2.76E-18 | Down |
| CORO2B | -2.212683 | 5.40E-22 | 6.86E-21 | Down |
| RASL12 | -2.300157 | 5.29E-44 | 2.12E-42 | Down |
| CTSH | -2.113472 | 1.77E-28 | 3.39E-27 | Down |
| FAM189A1 | -2.237406 | 1.90E-11 | 9.09E-11 | Down |
| RP1 | -3.008226 | 4.79E-17 | 4.07E-16 | Down |
| RETN | -5.066037 | 2.36E-86 | 6.47E-84 | Down |
| CLEC4M | -4.128358 | 5.47E-36 | 1.55E-34 | Down |
| NOVA2 | -2.805378 | 1.47E-65 | 1.50E-63 | Down |
| LILRA1 | -2.184679 | 1.82E-27 | 3.26E-26 | Down |
| PRX | -3.923086 | 5.98E-111 | 5.41E-108 | Down |
| DENND3 | -2.228794 | 2.57E-38 | 8.12E-37 | Down |
| CD33 | -2.151609 | 6.81E-31 | 1.51E-29 | Down |
| SIGLEC6 | -2.133602 | 3.39E-14 | 2.15E-13 | Down |
| HAS1 | -3.587729 | 9.84E-30 | 2.04E-28 | Down |
| CAV1 | -3.245495 | 7.27E-58 | 5.45E-56 | Down |
| TSPAN12 | -2.643062 | 1.58E-40 | 5.56E-39 | Down |
| CPED1 | -2.810741 | 4.10E-51 | 2.24E-49 | Down |
| COBL | -2.673496 | 8.33E-25 | 1.27E-23 | Down |
| MEOX2 | -2.1538 | 2.28E-20 | 2.53E-19 | Down |
| SLC1A1 | -3.190142 | 1.23E-53 | 7.67E-52 | Down |
| C5 | -2.026655 | 2.77E-22 | 3.60E-21 | Down |
| OGN | -4.477892 | 6.05E-67 | 6.54E-65 | Down |
| ENG | -2.361904 | 1.92E-66 | 2.04E-64 | Down |
| AK1 | -2.301488 | 2.58E-71 | 3.56E-69 | Down |
| PIP5K1B | -2.527688 | 2.11E-29 | 4.29E-28 | Down |
| SH3GL2 | -2.147301 | 7.29E-07 | 2.03E-06 | Down |
| PTGDS | -2.537922 | 1.00E-27 | 1.83E-26 | Down |
| SPOCK2 | -2.971532 | 1.16E-29 | 2.40E-28 | Down |
| CSF3 | -3.981321 | 3.78E-28 | 7.10E-27 | Down |
| ASPA | -3.889613 | 1.50E-81 | 3.36E-79 | Down |
| SLC6A4 | -5.770266 | 5.31E-69 | 6.53E-67 | Down |
| ICAM2 | -2.307296 | 2.73E-39 | 9.04E-38 | Down |
| CCL2 | -2.286264 | 7.76E-23 | 1.05E-21 | Down |
| SGCA | -3.007739 | 1.59E-37 | 4.85E-36 | Down |
| MYH1 | -2.987582 | 4.76E-17 | 4.04E-16 | Down |
| WFS1 | -2.080235 | 3.38E-75 | 5.76E-73 | Down |
| SNX25 | -2.254001 | 2.76E-92 | 1.02E-89 | Down |
| LRP2BP | -2.767895 | 4.60E-65 | 4.62E-63 | Down |
| ZBTB16 | -3.997193 | 2.37E-40 | 8.26E-39 | Down |
| FOLR1 | -3.790979 | 2.10E-36 | 6.08E-35 | Down |
| FOLR3 | -2.732379 | 1.83E-12 | 9.76E-12 | Down |
| C11orf21 | -2.01052 | 3.88E-16 | 2.98E-15 | Down |
| PTPN5 | -3.042373 | 4.48E-23 | 6.21E-22 | Down |
| VWF | -3.033217 | 6.77E-79 | 1.36E-76 | Down |
| SELPLG | -2.182801 | 9.26E-36 | 2.59E-34 | Down |
| DAO | -2.442678 | 1.31E-13 | 7.86E-13 | Down |
| ACSS3 | -2.177727 | 4.96E-22 | 6.32E-21 | Down |
| TNS2 | -2.412971 | 4.97E-82 | 1.15E-79 | Down |
| ALDH2 | -2.098237 | 8.77E-45 | 3.58E-43 | Down |
| ART4 | -2.317525 | 6.81E-09 | 2.43E-08 | Down |
| MGP | -2.355748 | 1.39E-25 | 2.24E-24 | Down |
| ADGRD1 | -3.780559 | 2.21E-68 | 2.56E-66 | Down |
| RSPH4A | -2.679593 | 6.22E-21 | 7.25E-20 | Down |
| NEDD9 | -2.008112 | 3.97E-31 | 8.88E-30 | Down |
| FAM184A | -2.484062 | 3.04E-27 | 5.38E-26 | Down |
| SASH1 | -2.052283 | 1.01E-50 | 5.44E-49 | Down |
| PHACTR1 | -2.800012 | 1.05E-66 | 1.12E-64 | Down |
| BMP5 | -2.339718 | 4.80E-12 | 2.45E-11 | Down |
| FHL5 | -4.051742 | 2.07E-87 | 6.05E-85 | Down |
| KHDRBS2 | -4.033263 | 8.47E-48 | 3.99E-46 | Down |
| C6orf118 | -2.91582 | 1.91E-11 | 9.15E-11 | Down |
| CLIC5 | -5.21556 | 3.98E-113 | 4.00E-110 | Down |
| LY86 | -2.097326 | 1.78E-24 | 2.68E-23 | Down |
| C7 | -3.187485 | 4.68E-26 | 7.78E-25 | Down |
| NME5 | -2.314569 | 2.19E-15 | 1.57E-14 | Down |
| BTNL8 | -3.001357 | 2.78E-41 | 1.01E-39 | Down |
| PCDH12 | -2.563417 | 1.41E-72 | 2.04E-70 | Down |
| LIFR | -2.223142 | 4.11E-20 | 4.50E-19 | Down |
| RBP2 | -3.607449 | 1.30E-43 | 5.11E-42 | Down |
| HYAL1 | -2.772488 | 7.59E-47 | 3.44E-45 | Down |
| HHLA2 | -2.076048 | 7.73E-11 | 3.46E-10 | Down |
| EFCC1 | -3.641936 | 8.16E-87 | 2.30E-84 | Down |
| VIPR1 | -3.543765 | 2.26E-82 | 5.30E-80 | Down |
| TNNC1 | -5.120054 | 2.83E-100 | 1.55E-97 | Down |
| SPTBN1 | -2.190598 | 3.15E-103 | 1.96E-100 | Down |
| ACADL | -3.377795 | 6.09E-44 | 2.43E-42 | Down |
| EFEMP1 | -2.02228 | 2.81E-20 | 3.09E-19 | Down |
| DNAH6 | -2.336088 | 1.41E-16 | 1.14E-15 | Down |
| IL1RL1 | -4.449003 | 2.64E-76 | 4.73E-74 | Down |
| MLPH | -2.692276 | 2.56E-26 | 4.30E-25 | Down |
| EPAS1 | -3.206613 | 5.61E-85 | 1.49E-82 | Down |
| TNR | -3.590473 | 6.79E-23 | 9.25E-22 | Down |
| ANGPTL1 | -3.629855 | 1.73E-67 | 1.92E-65 | Down |
| LEPR | -2.660095 | 7.07E-47 | 3.22E-45 | Down |
| PRG4 | -6.232497 | 1.14E-125 | 2.96E-122 | Down |
| NR5A2 | -2.138864 | 6.61E-32 | 1.52E-30 | Down |
| KMO | -2.097272 | 1.78E-22 | 2.34E-21 | Down |
| ST6GALNAC5 | -2.360759 | 3.96E-36 | 1.13E-34 | Down |
| KIF17 | -2.14292 | 5.55E-32 | 1.28E-30 | Down |
| MROH9 | -3.043881 | 4.60E-21 | 5.43E-20 | Down |
| 2-Mar | -2.005375 | 1.31E-52 | 7.73E-51 | Down |
| SLC5A9 | -3.261293 | 3.13E-33 | 7.65E-32 | Down |
| CASC1 | -2.227286 | 1.16E-16 | 9.47E-16 | Down |
| FILIP1 | -2.791066 | 2.24E-41 | 8.18E-40 | Down |
| ADGB | -3.27643 | 4.70E-15 | 3.25E-14 | Down |
| TCF21 | -4.437579 | 8.75E-108 | 6.88E-105 | Down |
| CASQ2 | -2.743686 | 6.34E-18 | 5.80E-17 | Down |
| PCDH17 | -2.103171 | 2.49E-42 | 9.44E-41 | Down |
| DNAH7 | -2.839571 | 1.30E-29 | 2.68E-28 | Down |
| KLF9 | -2.016886 | 8.68E-35 | 2.29E-33 | Down |
| C2orf40 | -3.046725 | 1.59E-19 | 1.67E-18 | Down |
| SLC46A2 | -4.235522 | 1.38E-59 | 1.14E-57 | Down |
| NR4A3 | -2.753004 | 2.69E-25 | 4.27E-24 | Down |
| CSF3R | -2.166148 | 2.34E-20 | 2.59E-19 | Down |
| FLVCR2 | -2.294174 | 9.21E-44 | 3.65E-42 | Down |
| CFAP58 | -2.313309 | 3.65E-21 | 4.35E-20 | Down |
| DUSP1 | -2.266559 | 2.15E-34 | 5.57E-33 | Down |
| TEK | -3.924295 | 1.40E-123 | 2.54E-120 | Down |
| CCDC170 | -2.705959 | 7.96E-25 | 1.22E-23 | Down |
| MYCT1 | -3.079265 | 3.69E-94 | 1.59E-91 | Down |
| IQSEC3 | -3.071323 | 5.67E-45 | 2.36E-43 | Down |
| SMAD9 | -2.002239 | 4.68E-22 | 5.97E-21 | Down |
| ADRA1A | -4.331649 | 4.03E-36 | 1.15E-34 | Down |
| PDLIM2 | -2.080964 | 2.62E-56 | 1.82E-54 | Down |
| TBX4 | -3.083837 | 4.80E-53 | 2.96E-51 | Down |
| CAT | -2.093283 | 2.52E-59 | 2.06E-57 | Down |
| FABP3 | -2.317876 | 1.37E-38 | 4.41E-37 | Down |
| CCRL2 | -2.30964 | 9.40E-36 | 2.63E-34 | Down |
| LDB3 | -2.320772 | 3.35E-37 | 1.00E-35 | Down |
| RAMP3 | -3.389942 | 1.02E-103 | 6.80E-101 | Down |
| GLIPR2 | -2.448735 | 6.85E-63 | 6.45E-61 | Down |
| RECK | -2.009057 | 7.05E-43 | 2.71E-41 | Down |
| DNAI1 | -2.736974 | 5.03E-13 | 2.84E-12 | Down |
| SFTPA1 | -4.31412 | 1.65E-29 | 3.38E-28 | Down |
| NECAB1 | -3.207326 | 4.23E-49 | 2.11E-47 | Down |
| ADGRE5 | -2.377339 | 2.65E-53 | 1.64E-51 | Down |
| ITIH5 | -2.346542 | 2.08E-13 | 1.22E-12 | Down |
| MMP19 | -2.26628 | 1.57E-23 | 2.24E-22 | Down |
| NR4A1 | -2.178706 | 4.33E-22 | 5.53E-21 | Down |
| PDE1B | -2.252592 | 2.16E-38 | 6.87E-37 | Down |
| NFE2 | -2.07246 | 3.54E-13 | 2.03E-12 | Down |
| C4BPA | -3.134215 | 1.61E-17 | 1.42E-16 | Down |
| DAW1 | -2.426413 | 2.69E-10 | 1.12E-09 | Down |
| MOGAT1 | -3.296428 | 4.88E-21 | 5.74E-20 | Down |
| FAM124B | -2.153318 | 8.13E-29 | 1.58E-27 | Down |
| PTGIS | -2.410873 | 1.36E-25 | 2.19E-24 | Down |
| C20orf85 | -2.677498 | 6.28E-08 | 1.99E-07 | Down |
| ATP8A1 | -2.438532 | 1.56E-30 | 3.38E-29 | Down |
| CEACAM8 | -5.32543 | 4.98E-66 | 5.15E-64 | Down |
| SLC17A3 | -2.877833 | 8.18E-14 | 5.02E-13 | Down |
| TREM1 | -2.805978 | 1.35E-32 | 3.20E-31 | Down |
| KCNK17 | -2.088679 | 3.00E-17 | 2.60E-16 | Down |
| MYRF | -3.607441 | 1.16E-83 | 2.91E-81 | Down |
| SLC10A2 | -3.219808 | 8.70E-14 | 5.32E-13 | Down |
| TEKT3 | -2.237696 | 1.26E-23 | 1.81E-22 | Down |
| MYH2 | -4.627628 | 3.07E-51 | 1.70E-49 | Down |
| KIR2DL1 | -2.486114 | 1.85E-11 | 8.85E-11 | Down |
| C3 | -2.008169 | 7.16E-20 | 7.72E-19 | Down |
| FOSB | -2.773072 | 1.56E-20 | 1.75E-19 | Down |
| FOXA2 | -3.095603 | 1.74E-21 | 2.13E-20 | Down |
| CD93 | -2.956689 | 2.71E-73 | 4.18E-71 | Down |
| BMP2 | -2.048573 | 4.32E-19 | 4.36E-18 | Down |
| FLRT3 | -2.390391 | 2.78E-20 | 3.06E-19 | Down |
| LRRN4 | -4.069948 | 2.42E-49 | 1.23E-47 | Down |
| SIRPD | -2.823583 | 1.27E-38 | 4.11E-37 | Down |
| S1PR4 | -2.103852 | 4.93E-23 | 6.80E-22 | Down |
| MMP24 | -2.797461 | 2.14E-45 | 9.14E-44 | Down |
| F10 | -2.15443 | 1.83E-18 | 1.76E-17 | Down |
| CFP | -2.620399 | 9.33E-39 | 3.03E-37 | Down |
| RHOJ | -2.547013 | 1.31E-57 | 9.72E-56 | Down |
| PZP | -2.13638 | 1.76E-10 | 7.55E-10 | Down |
| OMG | -2.326033 | 2.20E-14 | 1.42E-13 | Down |
| AVPR2 | -2.553799 | 2.68E-29 | 5.39E-28 | Down |
| MASP1 | -2.069686 | 2.55E-10 | 1.07E-09 | Down |
| ATP13A4 | -2.572435 | 1.74E-20 | 1.95E-19 | Down |
| PTPRB | -3.588184 | 1.11E-123 | 2.23E-120 | Down |
| KLF2 | -2.660086 | 7.22E-50 | 3.77E-48 | Down |
| F2RL3 | -2.369788 | 7.67E-29 | 1.50E-27 | Down |
| GNG11 | -2.952588 | 2.46E-56 | 1.72E-54 | Down |
| STEAP4 | -3.321964 | 2.38E-37 | 7.23E-36 | Down |
| KDR | -2.3934 | 5.05E-50 | 2.67E-48 | Down |
| DOCK4 | -2.344278 | 1.15E-71 | 1.62E-69 | Down |
| CDHR3 | -2.164377 | 2.02E-14 | 1.31E-13 | Down |
| CGNL1 | -3.086692 | 2.50E-66 | 2.64E-64 | Down |
| ALDH1A2 | -3.042464 | 1.94E-32 | 4.58E-31 | Down |
| ACKR4 | -3.04976 | 8.04E-61 | 7.02E-59 | Down |
| ADCY4 | -2.455057 | 8.03E-55 | 5.31E-53 | Down |
| RNASE1 | -2.695709 | 2.44E-47 | 1.12E-45 | Down |
| CDO1 | -2.904158 | 8.63E-26 | 1.41E-24 | Down |
| ART1 | -3.476491 | 1.02E-30 | 2.24E-29 | Down |
| KCNA5 | -2.301826 | 1.05E-14 | 6.98E-14 | Down |
| STARD8 | -2.798814 | 1.04E-72 | 1.52E-70 | Down |
| DPP6 | -2.906867 | 1.27E-14 | 8.43E-14 | Down |
| USHBP1 | -2.964897 | 1.29E-93 | 5.17E-91 | Down |
| CACNG6 | -2.105788 | 2.67E-06 | 6.88E-06 | Down |
| IQCN | -2.053058 | 1.55E-16 | 1.25E-15 | Down |
| GATA5 | -2.235109 | 4.18E-11 | 1.93E-10 | Down |
| RGN | -2.506619 | 1.62E-22 | 2.14E-21 | Down |
| AKAP12 | -2.478393 | 2.39E-31 | 5.41E-30 | Down |
| SYNE1 | -3.009215 | 1.74E-67 | 1.92E-65 | Down |
| TTLL9 | -2.090733 | 6.99E-15 | 4.75E-14 | Down |
| COX4I2 | -2.842223 | 1.51E-59 | 1.24E-57 | Down |
| HIGD1B | -3.688746 | 2.99E-114 | 3.18E-111 | Down |
| ADGRE3 | -3.086838 | 2.79E-32 | 6.54E-31 | Down |
| NAPSA | -3.537804 | 7.81E-25 | 1.20E-23 | Down |
| AOC3 | -3.52763 | 1.46E-83 | 3.55E-81 | Down |
| RAMP2 | -3.123848 | 3.01E-134 | 1.09E-130 | Down |
| TMEM204 | -2.152339 | 8.71E-45 | 3.58E-43 | Down |
| FMO5 | -2.772137 | 2.59E-35 | 7.06E-34 | Down |
| RAI2 | -2.33109 | 1.85E-39 | 6.18E-38 | Down |
| CNGA4 | -2.621822 | 8.21E-18 | 7.44E-17 | Down |
| RGS22 | -2.513301 | 1.51E-18 | 1.46E-17 | Down |
| HSPA12B | -2.905123 | 2.76E-100 | 1.55E-97 | Down |
| NES | -2.016779 | 1.97E-22 | 2.58E-21 | Down |
| ALOX5AP | -2.814815 | 1.66E-45 | 7.19E-44 | Down |
| MYH10 | -2.003534 | 3.55E-44 | 1.43E-42 | Down |
| STOML3 | -2.154878 | 4.93E-08 | 1.58E-07 | Down |
| KL | -3.399764 | 1.43E-49 | 7.32E-48 | Down |
| STARD13 | -2.346858 | 8.74E-45 | 3.58E-43 | Down |
| PRAM1 | -2.77851 | 1.80E-58 | 1.39E-56 | Down |
| LGALS12 | -2.082197 | 8.42E-18 | 7.63E-17 | Down |
| PDZD2 | -2.277739 | 6.30E-20 | 6.81E-19 | Down |
| GIMAP6 | -2.562219 | 8.96E-46 | 3.90E-44 | Down |
| LRRIQ1 | -2.344221 | 1.63E-16 | 1.30E-15 | Down |
| SFTPD | -3.678927 | 8.55E-29 | 1.66E-27 | Down |
| DYDC2 | -2.414767 | 1.09E-13 | 6.57E-13 | Down |
| CA1 | -2.393883 | 9.14E-15 | 6.14E-14 | Down |
| LYVE1 | -3.831261 | 2.26E-87 | 6.47E-85 | Down |
| PEBP4 | -4.296896 | 1.42E-37 | 4.35E-36 | Down |
| CNTN6 | -5.176563 | 6.91E-70 | 8.99E-68 | Down |
| GSTM5 | -2.678767 | 7.56E-43 | 2.89E-41 | Down |
| CHIA | -3.656239 | 5.66E-20 | 6.14E-19 | Down |
| HMGCS2 | -2.26566 | 6.95E-06 | 1.70E-05 | Down |
| CD101 | -2.279813 | 6.37E-50 | 3.35E-48 | Down |
| CABLES1 | -2.072964 | 1.21E-27 | 2.20E-26 | Down |
| ADAMTS8 | -4.899125 | 3.88E-79 | 7.96E-77 | Down |
| KLB | -2.824168 | 6.26E-19 | 6.24E-18 | Down |
| FAM189A2 | -4.152215 | 1.28E-84 | 3.30E-82 | Down |
| CD36 | -2.703798 | 6.71E-27 | 1.17E-25 | Down |
| ADGRB3 | -2.157329 | 3.30E-12 | 1.71E-11 | Down |
| STX11 | -2.603683 | 3.39E-50 | 1.81E-48 | Down |
| CPM | -2.481995 | 4.60E-29 | 9.11E-28 | Down |
| CAPN9 | -3.371346 | 4.99E-41 | 1.79E-39 | Down |
| SLC19A3 | -3.342297 | 5.20E-37 | 1.54E-35 | Down |
| CYP27A1 | -2.306926 | 2.89E-32 | 6.79E-31 | Down |
| STAB2 | -2.264607 | 1.55E-21 | 1.91E-20 | Down |
| USP44 | -2.220702 | 1.81E-24 | 2.73E-23 | Down |
| DRAM1 | -2.499306 | 3.05E-54 | 1.95E-52 | Down |
| LMO7 | -2.867245 | 5.84E-94 | 2.40E-91 | Down |
| SCEL | -2.908177 | 1.16E-24 | 1.75E-23 | Down |
| EDNRB | -4.136314 | 3.86E-100 | 2.05E-97 | Down |
| IL6 | -2.776053 | 1.75E-22 | 2.30E-21 | Down |
| NKX2-1 | -2.748896 | 4.90E-15 | 3.38E-14 | Down |
| TM6SF1 | -2.141341 | 4.82E-32 | 1.12E-30 | Down |
| SCN7A | -4.400416 | 2.77E-78 | 5.38E-76 | Down |
| GYPC | -2.085822 | 2.00E-28 | 3.83E-27 | Down |
| TLR4 | -2.008418 | 7.28E-23 | 9.90E-22 | Down |
| WDR38 | -2.625284 | 5.93E-12 | 3.00E-11 | Down |
| HEMGN | -2.243851 | 1.45E-10 | 6.27E-10 | Down |
| IL33 | -2.018487 | 6.90E-14 | 4.27E-13 | Down |
| MGARP | -2.013156 | 3.28E-13 | 1.89E-12 | Down |
| TTC29 | -2.701025 | 2.41E-10 | 1.01E-09 | Down |
| ARRB1 | -2.802199 | 2.95E-55 | 1.97E-53 | Down |
| SLCO2B1 | -2.150138 | 4.79E-26 | 7.95E-25 | Down |
| LRRC32 | -2.881993 | 1.29E-71 | 1.81E-69 | Down |
| SMAD6 | -2.767853 | 7.44E-65 | 7.38E-63 | Down |
| GCOM1 | -3.055264 | 1.01E-35 | 2.81E-34 | Down |
| GIPC2 | -2.023434 | 1.96E-12 | 1.04E-11 | Down |
| ARHGAP29 | -2.354883 | 1.65E-34 | 4.31E-33 | Down |
| DNASE2B | -3.223068 | 1.39E-33 | 3.47E-32 | Down |
| RBP4 | -2.297805 | 2.16E-09 | 8.15E-09 | Down |
| AOX1 | -3.060232 | 5.14E-40 | 1.77E-38 | Down |
| HECW2 | -2.603873 | 5.83E-75 | 9.85E-73 | Down |
| SECISBP2L | -2.018293 | 4.04E-94 | 1.70E-91 | Down |
| MMRN1 | -3.37409 | 3.26E-52 | 1.88E-50 | Down |
| PDE5A | -2.11323 | 1.14E-36 | 3.32E-35 | Down |
| TRPC3 | -2.151194 | 1.47E-21 | 1.82E-20 | Down |
| CDKL2 | -2.548149 | 1.09E-28 | 2.11E-27 | Down |
| ANXA3 | -2.343515 | 2.06E-24 | 3.08E-23 | Down |
| SLC39A8 | -3.681217 | 6.78E-88 | 2.04E-85 | Down |
| PTPRQ | -2.170797 | 3.22E-10 | 1.34E-09 | Down |
| ACVRL1 | -3.057959 | 2.21E-117 | 2.86E-114 | Down |
| METTL21C | -2.268134 | 1.75E-11 | 8.41E-11 | Down |
| SSTR1 | -3.3771 | 3.00E-26 | 5.02E-25 | Down |
| SLC24A4 | -3.069471 | 3.54E-58 | 2.69E-56 | Down |
| FBLN5 | -2.82211 | 1.10E-59 | 9.19E-58 | Down |
| CCDC33 | -2.612728 | 7.00E-11 | 3.15E-10 | Down |
| CYP1A2 | -2.058961 | 0.0005439 | 0.0010126 | Down |
| NTRK3 | -2.1324 | 2.31E-10 | 9.74E-10 | Down |
| MYLK3 | -2.014222 | 6.01E-15 | 4.12E-14 | Down |
| NKD1 | -2.025255 | 1.15E-15 | 8.50E-15 | Down |
| MYOCD | -3.508598 | 1.49E-39 | 5.02E-38 | Down |
| LRRC46 | -2.430267 | 3.91E-20 | 4.28E-19 | Down |
| ABCA8 | -4.311641 | 5.13E-81 | 1.13E-78 | Down |
| GATA6 | -2.44677 | 3.48E-35 | 9.41E-34 | Down |
| NLRP12 | -2.629791 | 3.23E-24 | 4.79E-23 | Down |
| CFAP74 | -2.017219 | 4.29E-09 | 1.56E-08 | Down |
| PRDM16 | -2.40851 | 2.77E-20 | 3.05E-19 | Down |
| FCN3 | -4.531201 | 7.54E-69 | 9.15E-67 | Down |
| CYP4B1 | -4.478391 | 1.22E-51 | 6.88E-50 | Down |
| C1orf162 | -2.399953 | 2.61E-43 | 1.02E-41 | Down |
| ITGA10 | -2.442688 | 3.41E-40 | 1.19E-38 | Down |
| GPA33 | -5.139035 | 1.14E-90 | 3.87E-88 | Down |
| RXRG | -2.587683 | 1.08E-13 | 6.54E-13 | Down |
| RGS5 | -2.091415 | 3.93E-29 | 7.84E-28 | Down |
| RORC | -2.257465 | 8.68E-14 | 5.31E-13 | Down |
| ADAMTSL4 | -2.069555 | 4.99E-16 | 3.80E-15 | Down |
| SELENBP1 | -3.38042 | 6.64E-53 | 4.04E-51 | Down |
| LEFTY2 | -3.431881 | 7.31E-39 | 2.39E-37 | Down |
| REN | -2.32373 | 5.02E-19 | 5.03E-18 | Down |
| AFF3 | -3.19093 | 1.08E-35 | 3.00E-34 | Down |
| GPR17 | -3.312056 | 3.32E-37 | 9.97E-36 | Down |
| SCN1A | -3.161343 | 1.43E-23 | 2.05E-22 | Down |
| ZNF385B | -3.196662 | 4.66E-31 | 1.04E-29 | Down |
| NYAP2 | -2.194358 | 2.92E-11 | 1.37E-10 | Down |
| RBMS3 | -2.275608 | 1.79E-46 | 7.96E-45 | Down |
| GADL1 | -2.388916 | 8.86E-15 | 5.96E-14 | Down |
| CSRNP1 | -2.645444 | 5.25E-69 | 6.50E-67 | Down |
| ITGA9 | -2.141768 | 3.72E-32 | 8.63E-31 | Down |
| STAC | -3.053352 | 4.28E-33 | 1.03E-31 | Down |
| PLA1A | -2.635934 | 4.45E-33 | 1.07E-31 | Down |
| AGTR1 | -2.643435 | 2.38E-17 | 2.07E-16 | Down |
| SLIT2 | -2.749075 | 3.95E-47 | 1.81E-45 | Down |
| ROPN1L | -2.251767 | 1.89E-11 | 9.02E-11 | Down |
| OTULINL | -2.650237 | 3.36E-54 | 2.14E-52 | Down |
| C1QTNF2 | -2.464316 | 2.00E-42 | 7.62E-41 | Down |
| DCDC2 | -2.62092 | 1.34E-19 | 1.42E-18 | Down |
| DAAM2 | -2.345446 | 3.30E-38 | 1.04E-36 | Down |
| HMGCLL1 | -2.601808 | 7.18E-20 | 7.74E-19 | Down |
| TCTE1 | -2.82426 | 2.61E-18 | 2.47E-17 | Down |
| PRSS35 | -2.281911 | 2.32E-18 | 2.21E-17 | Down |
| VIP | -2.218916 | 8.04E-14 | 4.95E-13 | Down |
| SLC22A3 | -2.000577 | 5.50E-11 | 2.50E-10 | Down |
| DENND2A | -2.516687 | 3.17E-45 | 1.33E-43 | Down |
| CXorf36 | -2.54004 | 9.10E-63 | 8.53E-61 | Down |
| AWAT2 | -3.318121 | 9.32E-27 | 1.60E-25 | Down |
| NXF3 | -2.275878 | 9.43E-10 | 3.71E-09 | Down |
| DOCK11 | -2.12053 | 2.05E-45 | 8.76E-44 | Down |
| DOK2 | -2.384112 | 2.81E-40 | 9.78E-39 | Down |
| DPYS | -2.294027 | 4.60E-15 | 3.19E-14 | Down |
| RSPO2 | -3.055013 | 2.85E-16 | 2.22E-15 | Down |
| SHC3 | -2.872459 | 1.04E-49 | 5.37E-48 | Down |
| SNX30 | -2.10855 | 5.25E-76 | 9.30E-74 | Down |
| HMCN2 | -2.497113 | 1.17E-24 | 1.78E-23 | Down |
| TMEM236 | -2.571569 | 5.75E-38 | 1.79E-36 | Down |
| ST8SIA6 | -2.938684 | 1.09E-29 | 2.24E-28 | Down |
| FAM13C | -2.453884 | 2.08E-43 | 8.15E-42 | Down |
| ANKRD1 | -5.742665 | 3.04E-90 | 9.98E-88 | Down |
| CYP17A1 | -2.389339 | 8.58E-27 | 1.48E-25 | Down |
| SCGB1A1 | -3.977038 | 3.01E-22 | 3.91E-21 | Down |
| GRIK4 | -2.28373 | 2.43E-15 | 1.74E-14 | Down |
| GGTLC1 | -5.500744 | 9.29E-57 | 6.59E-55 | Down |
| MS4A2 | -2.909952 | 8.05E-41 | 2.86E-39 | Down |
| ESAM | -2.897196 | 7.98E-98 | 4.01E-95 | Down |
| SCN2B | -2.638601 | 2.27E-23 | 3.21E-22 | Down |
| KLRF1 | -2.090825 | 9.44E-15 | 6.32E-14 | Down |
| PCDH15 | -3.413326 | 2.98E-27 | 5.28E-26 | Down |
| GPM6A | -5.428849 | 9.93E-77 | 1.81E-74 | Down |
| SPATA4 | -2.582966 | 2.56E-14 | 1.65E-13 | Down |
| ENKUR | -2.274284 | 5.66E-16 | 4.28E-15 | Down |
| NR3C2 | -2.463735 | 6.42E-28 | 1.19E-26 | Down |
| FLI1 | -2.117177 | 1.30E-34 | 3.41E-33 | Down |
| WWC2 | -3.194799 | 4.83E-104 | 3.35E-101 | Down |
| GFRA1 | -2.340823 | 6.00E-17 | 5.02E-16 | Down |
| TMEM163 | -2.061846 | 1.53E-18 | 1.48E-17 | Down |
| GUCY1A2 | -2.850831 | 1.26E-67 | 1.41E-65 | Down |
| OLAH | -2.214673 | 1.68E-11 | 8.09E-11 | Down |
| PLEKHH2 | -2.315598 | 4.21E-38 | 1.32E-36 | Down |
| IGSF10 | -2.449029 | 3.56E-22 | 4.58E-21 | Down |
| CAPSL | -2.811693 | 3.10E-13 | 1.79E-12 | Down |
| TCTEX1D1 | -2.389887 | 1.95E-22 | 2.56E-21 | Down |
| WDR78 | -2.184338 | 2.64E-22 | 3.44E-21 | Down |
| SLC16A12 | -2.941099 | 9.89E-31 | 2.17E-29 | Down |
| UTRN | -2.047526 | 2.22E-51 | 1.23E-49 | Down |
| ACOXL | -3.445485 | 1.38E-68 | 1.62E-66 | Down |
| FAM81B | -2.622222 | 7.83E-13 | 4.32E-12 | Down |
| C16orf89 | -3.583647 | 2.25E-29 | 4.57E-28 | Down |
| FAM92B | -2.321642 | 5.19E-12 | 2.64E-11 | Down |
| PID1 | -2.463134 | 6.11E-37 | 1.80E-35 | Down |
| ANKRD29 | -2.499016 | 2.90E-30 | 6.20E-29 | Down |
| DNAAF1 | -2.424184 | 2.99E-15 | 2.12E-14 | Down |
| ROBO4 | -3.525063 | 1.24E-128 | 3.74E-125 | Down |
| NRGN | -2.437797 | 1.27E-21 | 1.57E-20 | Down |
| ABI3BP | -3.489935 | 1.30E-64 | 1.28E-62 | Down |
| ANGPT1 | -2.810045 | 1.94E-39 | 6.49E-38 | Down |
| ABCA9 | -2.374791 | 1.62E-35 | 4.45E-34 | Down |
| ABCA6 | -2.05199 | 1.57E-23 | 2.24E-22 | Down |
| DISP1 | -2.122561 | 7.86E-35 | 2.09E-33 | Down |
| FAM167A | -2.476888 | 5.95E-21 | 6.95E-20 | Down |
| PGM5 | -3.35039 | 2.95E-45 | 1.25E-43 | Down |
| CCDC173 | -2.712832 | 2.11E-29 | 4.29E-28 | Down |
| SORBS2 | -2.434231 | 1.06E-26 | 1.81E-25 | Down |
| PDE1C | -3.136003 | 7.24E-59 | 5.79E-57 | Down |
| JAM2 | -3.048436 | 1.01E-89 | 3.19E-87 | Down |
| FGD5 | -2.624349 | 2.12E-54 | 1.37E-52 | Down |
| PIEZO2 | -2.062811 | 7.90E-19 | 7.82E-18 | Down |
| RSPH10B | -2.027499 | 5.38E-18 | 4.96E-17 | Down |
| GRIA1 | -4.770174 | 8.62E-55 | 5.64E-53 | Down |
| VSIG4 | -3.019565 | 2.37E-45 | 1.01E-43 | Down |
| ALS2CR12 | -2.167936 | 5.77E-22 | 7.32E-21 | Down |
| GNA14 | -2.299537 | 3.95E-21 | 4.70E-20 | Down |
| WIF1 | -2.904909 | 6.90E-11 | 3.11E-10 | Down |
| CFAP161 | -2.350948 | 6.59E-20 | 7.12E-19 | Down |
| ADAMTSL3 | -2.633033 | 2.44E-25 | 3.90E-24 | Down |
| FGF18 | -2.129915 | 1.17E-14 | 7.78E-14 | Down |
| C1orf158 | -3.08106 | 1.44E-11 | 7.00E-11 | Down |
| CACNA1D | -2.445969 | 1.26E-20 | 1.43E-19 | Down |
| HYDIN | -2.854139 | 3.01E-19 | 3.10E-18 | Down |
| ERG | -2.480854 | 5.95E-61 | 5.22E-59 | Down |
| SLC34A2 | -3.435103 | 3.73E-27 | 6.57E-26 | Down |
| DRC1 | -2.249019 | 1.35E-09 | 5.19E-09 | Down |
| CIB4 | -2.218732 | 4.33E-10 | 1.77E-09 | Down |
| ESYT3 | -2.792346 | 5.46E-36 | 1.55E-34 | Down |
| COLEC12 | -2.745323 | 5.12E-42 | 1.91E-40 | Down |
| SHROOM4 | -2.801578 | 3.20E-57 | 2.32E-55 | Down |
| CATIP | -2.585189 | 1.59E-22 | 2.10E-21 | Down |
| KCNB1 | -2.289593 | 2.07E-10 | 8.76E-10 | Down |
| AHCYL2 | -2.325192 | 1.02E-49 | 5.29E-48 | Down |
| ALAS2 | -3.605811 | 7.63E-44 | 3.04E-42 | Down |
| ITLN2 | -5.170691 | 5.49E-77 | 1.02E-74 | Down |
| VWA5B1 | -2.117947 | 6.52E-11 | 2.95E-10 | Down |
| SCUBE1 | -4.380349 | 8.27E-68 | 9.34E-66 | Down |
| PADI4 | -2.519812 | 4.75E-14 | 2.98E-13 | Down |
| STARD9 | -2.541934 | 1.49E-51 | 8.39E-50 | Down |
| DRC7 | -2.680004 | 3.21E-14 | 2.05E-13 | Down |
| ACE | -2.457259 | 2.40E-68 | 2.74E-66 | Down |
| LRRC36 | -4.555633 | 8.68E-109 | 7.13E-106 | Down |
| TPPP3 | -2.683243 | 1.45E-30 | 3.15E-29 | Down |
| AGRP | -4.594488 | 2.62E-94 | 1.18E-91 | Down |
| PTGIR | -2.096188 | 2.52E-35 | 6.89E-34 | Down |
| CPAMD8 | -2.925695 | 8.52E-35 | 2.26E-33 | Down |
| RSPH1 | -2.721847 | 1.64E-17 | 1.44E-16 | Down |
| TMEM190 | -2.160895 | 2.94E-09 | 1.09E-08 | Down |
| JAML | -2.456078 | 5.28E-37 | 1.56E-35 | Down |
| PTH1R | -2.420896 | 2.68E-25 | 4.27E-24 | Down |
| MYL3 | -2.121215 | 1.48E-18 | 1.43E-17 | Down |
| LRRC71 | -2.872536 | 2.25E-18 | 2.14E-17 | Down |
| FGFR4 | -2.868353 | 6.08E-35 | 1.63E-33 | Down |
| CYP3A7 | -2.859581 | 1.29E-40 | 4.54E-39 | Down |
| HK3 | -2.205886 | 7.36E-27 | 1.28E-25 | Down |
| SCGB3A1 | -3.225422 | 1.21E-17 | 1.08E-16 | Down |
| COX7A1 | -2.280621 | 1.14E-38 | 3.68E-37 | Down |
| SIGLEC11 | -3.295232 | 4.11E-40 | 1.42E-38 | Down |
| CD300LG | -4.943742 | 1.11E-42 | 4.24E-41 | Down |
| GRASP | -2.193367 | 1.11E-30 | 2.42E-29 | Down |
| SCIMP | -2.197593 | 2.47E-24 | 3.69E-23 | Down |
| BCL6B | -2.456201 | 8.11E-67 | 8.72E-65 | Down |
| TNFSF13 | -2.099056 | 1.29E-50 | 6.92E-49 | Down |
| ITIH3 | -2.168823 | 1.14E-23 | 1.64E-22 | Down |
| TAL1 | -3.062435 | 5.78E-49 | 2.86E-47 | Down |
| SYNC | -2.030929 | 8.52E-30 | 1.77E-28 | Down |
| UBXN10 | -2.17523 | 9.03E-13 | 4.95E-12 | Down |
| ALPL | -2.25202 | 1.60E-18 | 1.54E-17 | Down |
| C1orf87 | -3.173272 | 2.50E-13 | 1.46E-12 | Down |
| NEXN | -2.26242 | 2.82E-37 | 8.53E-36 | Down |
| B3GALT2 | -2.36919 | 1.41E-17 | 1.26E-16 | Down |
| NTNG1 | -2.565034 | 1.17E-11 | 5.74E-11 | Down |
| WDR63 | -2.667622 | 9.76E-23 | 1.31E-21 | Down |
| KCNT2 | -3.428538 | 1.91E-58 | 1.47E-56 | Down |
| LRRC52 | -2.222943 | 1.52E-08 | 5.20E-08 | Down |
| PIGR | -3.101001 | 3.01E-22 | 3.91E-21 | Down |
| TEKT4 | -2.393457 | 1.59E-14 | 1.04E-13 | Down |
| SPATA18 | -2.084981 | 5.56E-19 | 5.56E-18 | Down |
| NOSTRIN | -3.40661 | 5.99E-115 | 6.76E-112 | Down |
| CFAP221 | -2.627113 | 6.43E-19 | 6.40E-18 | Down |
| HPGDS | -2.712641 | 6.40E-45 | 2.66E-43 | Down |
| C1QTNF7 | -3.960149 | 8.06E-80 | 1.67E-77 | Down |
| TDRD10 | -2.905083 | 9.80E-28 | 1.79E-26 | Down |
| C1orf189 | -2.202743 | 2.02E-09 | 7.63E-09 | Down |
| ALPP | -2.050481 | 8.94E-06 | 2.15E-05 | Down |
| LMOD3 | -2.305662 | 1.15E-22 | 1.54E-21 | Down |
| PROK2 | -2.443007 | 3.68E-14 | 2.33E-13 | Down |
| LMOD1 | -2.465633 | 3.03E-37 | 9.13E-36 | Down |
| CXCR1 | -2.711873 | 3.26E-15 | 2.31E-14 | Down |
| NEK10 | -2.270367 | 6.55E-16 | 4.93E-15 | Down |
| CCDC141 | -3.449902 | 5.56E-40 | 1.91E-38 | Down |
| IHH | -2.529252 | 6.54E-09 | 2.33E-08 | Down |
| TGFBR2 | -2.741946 | 3.71E-71 | 5.04E-69 | Down |
| MNDA | -2.199848 | 1.40E-27 | 2.53E-26 | Down |
| EFHB | -2.606962 | 3.70E-23 | 5.18E-22 | Down |
| PTX3 | -2.877176 | 1.10E-18 | 1.08E-17 | Down |
| DNASE1L3 | -2.824383 | 3.40E-31 | 7.64E-30 | Down |
| PCOLCE2 | -2.017463 | 2.89E-15 | 2.05E-14 | Down |
| CXCL3 | -2.101152 | 5.98E-14 | 3.72E-13 | Down |
| CXCL5 | -2.540961 | 3.22E-13 | 1.86E-12 | Down |
| PPBP | -3.984959 | 1.19E-25 | 1.92E-24 | Down |
| PF4 | -4.070378 | 5.22E-45 | 2.18E-43 | Down |
| CPA3 | -2.232636 | 2.23E-21 | 2.69E-20 | Down |
| TM4SF18 | -2.261002 | 3.30E-37 | 9.94E-36 | Down |
| CLEC3B | -4.657624 | 1.01E-134 | 4.56E-131 | Down |
| SLC6A20 | -3.269668 | 6.23E-33 | 1.50E-31 | Down |
| LRRC2 | -2.783975 | 1.33E-34 | 3.49E-33 | Down |
| CFAP100 | -2.10702 | 1.15E-09 | 4.47E-09 | Down |
| SGMS2 | -2.848877 | 1.33E-57 | 9.83E-56 | Down |
| EMCN | -3.710577 | 9.13E-106 | 6.88E-103 | Down |
| CAMP | -2.784458 | 1.96E-19 | 2.04E-18 | Down |
| HPGD | -2.803629 | 1.33E-25 | 2.14E-24 | Down |
| HHIP | -3.75888 | 1.60E-32 | 3.79E-31 | Down |
| RANBP3L | -2.590838 | 8.90E-26 | 1.45E-24 | Down |
| SCGB3A2 | -2.336609 | 1.60E-09 | 6.14E-09 | Down |
| CITED2 | -2.008572 | 2.72E-37 | 8.25E-36 | Down |
| PI16 | -3.606537 | 1.24E-27 | 2.25E-26 | Down |
| SOX17 | -3.284394 | 8.19E-71 | 1.11E-68 | Down |
| DLC1 | -3.653297 | 2.22E-103 | 1.43E-100 | Down |
| C7orf57 | -2.232914 | 4.47E-13 | 2.53E-12 | Down |
| GPR146 | -2.362501 | 7.33E-62 | 6.69E-60 | Down |
| GPER1 | -2.593975 | 8.63E-38 | 2.67E-36 | Down |
| CA3 | -2.980152 | 7.50E-25 | 1.15E-23 | Down |
| DCSTAMP | -2.547944 | 4.69E-25 | 7.32E-24 | Down |
| C9orf24 | -2.446257 | 1.64E-12 | 8.78E-12 | Down |
| MAMDC2 | -3.552942 | 2.53E-33 | 6.22E-32 | Down |
| C8orf34 | -2.349803 | 9.95E-20 | 1.06E-18 | Down |
| SVEP1 | -2.756763 | 3.21E-51 | 1.76E-49 | Down |
| FBP1 | -3.08667 | 2.15E-45 | 9.16E-44 | Down |
| CYBB | -2.063979 | 1.40E-22 | 1.85E-21 | Down |
| VEGFD | -4.725675 | 1.14E-77 | 2.17E-75 | Down |
| AQP7 | -2.676764 | 1.82E-21 | 2.22E-20 | Down |
| ARMC3 | -2.651711 | 5.31E-13 | 2.99E-12 | Down |
| LRRC18 | -2.987648 | 9.62E-21 | 1.10E-19 | Down |
| PKNOX2 | -2.913653 | 2.15E-50 | 1.15E-48 | Down |
| DEPP1 | -2.396489 | 1.45E-36 | 4.20E-35 | Down |
| SLC18A2 | -2.045914 | 1.04E-22 | 1.39E-21 | Down |
| CLEC1B | -2.729963 | 2.29E-28 | 4.37E-27 | Down |
| JCAD | -2.908346 | 4.94E-73 | 7.51E-71 | Down |
| BTNL9 | -3.157532 | 4.58E-30 | 9.65E-29 | Down |
| CMTM5 | -2.068355 | 7.12E-09 | 2.53E-08 | Down |
| CYYR1 | -2.315692 | 3.70E-40 | 1.28E-38 | Down |
| TMEM100 | -4.997989 | 4.22E-69 | 5.26E-67 | Down |
| CYB5A | -2.08919 | 6.23E-38 | 1.93E-36 | Down |
| TMEM130 | -2.433472 | 1.67E-27 | 3.01E-26 | Down |
| PRTG | -2.00542 | 2.65E-11 | 1.24E-10 | Down |
| MFAP4 | -3.409106 | 2.69E-57 | 1.96E-55 | Down |
| CCDC68 | -2.829062 | 2.41E-39 | 8.03E-38 | Down |
| RRAD | -2.380602 | 2.85E-16 | 2.22E-15 | Down |
| CFAP52 | -2.810555 | 1.29E-20 | 1.46E-19 | Down |
| GPR182 | -2.224509 | 3.01E-28 | 5.70E-27 | Down |
| MS4A7 | -2.793607 | 9.44E-52 | 5.38E-50 | Down |
| MS4A14 | -2.212183 | 7.46E-26 | 1.23E-24 | Down |
| MS4A8 | -2.007981 | 0.0001283 | 0.0002613 | Down |
| MS4A15 | -5.258201 | 9.46E-69 | 1.14E-66 | Down |
| TTC16 | -2.314332 | 8.32E-19 | 8.22E-18 | Down |
| LOXHD1 | -3.448292 | 1.01E-58 | 7.93E-57 | Down |
| DPEP2 | -2.731394 | 8.58E-48 | 4.03E-46 | Down |
| CA4 | -5.197279 | 6.68E-68 | 7.59E-66 | Down |
| GPD1 | -5.463548 | 1.88E-162 | 2.47E-158 | Down |
| KIR3DL1 | -3.082769 | 6.48E-22 | 8.19E-21 | Down |
| PPP1R14A | -2.17033 | 3.45E-22 | 4.45E-21 | Down |
| CD300C | -2.694859 | 6.56E-49 | 3.24E-47 | Down |
| TEKT1 | -3.055556 | 1.66E-13 | 9.92E-13 | Down |
| TMEM88 | -2.488779 | 2.81E-41 | 1.02E-39 | Down |
| ABCA3 | -3.545001 | 6.35E-40 | 2.17E-38 | Down |
| SCARA5 | -4.507342 | 3.36E-57 | 2.43E-55 | Down |
| ACOX2 | -2.039132 | 8.25E-21 | 9.50E-20 | Down |
| FAM107A | -4.971219 | 1.60E-84 | 4.07E-82 | Down |
| CX3CR1 | -3.312697 | 6.81E-50 | 3.57E-48 | Down |
| MFSD2A | -2.023072 | 7.10E-27 | 1.23E-25 | Down |
| TNXB | -4.665138 | 1.76E-99 | 9.09E-97 | Down |
| LGI3 | -3.239785 | 3.16E-24 | 4.70E-23 | Down |
| SFTPC | -6.106963 | 8.82E-48 | 4.13E-46 | Down |
| CAVIN2 | -3.614009 | 1.35E-63 | 1.31E-61 | Down |
| CHRM1 | -3.884646 | 1.56E-30 | 3.38E-29 | Down |
| GFRA2 | -2.058365 | 1.83E-15 | 1.32E-14 | Down |
| DYNLRB2 | -2.525272 | 2.47E-18 | 2.34E-17 | Down |
| ADAM29 | -2.568249 | 2.41E-11 | 1.14E-10 | Down |
| MUCL3 | -3.663861 | 3.42E-26 | 5.72E-25 | Down |
| VWA3B | -3.156096 | 2.20E-22 | 2.88E-21 | Down |
| SLC16A4 | -2.064306 | 1.67E-20 | 1.88E-19 | Down |
| IL7R | -2.011253 | 7.14E-19 | 7.09E-18 | Down |
| ATOH8 | -3.655092 | 4.01E-51 | 2.20E-49 | Down |
| SFTPB | -3.065263 | 3.85E-17 | 3.30E-16 | Down |
| BTNL3 | -2.663 | 2.34E-11 | 1.11E-10 | Down |
| PLA2G4F | -2.593535 | 3.73E-27 | 6.57E-26 | Down |
| STXBP6 | -2.635322 | 6.67E-27 | 1.16E-25 | Down |
| COL4A3 | -3.709215 | 4.88E-50 | 2.60E-48 | Down |
| ZBBX | -2.716698 | 5.41E-12 | 2.75E-11 | Down |
| ARMC4 | -2.453825 | 3.78E-13 | 2.16E-12 | Down |
| IL13 | -2.092082 | 9.39E-09 | 3.30E-08 | Down |
| RSPO1 | -4.279555 | 4.58E-78 | 8.80E-76 | Down |
| GCSAML | -2.481323 | 7.93E-26 | 1.30E-24 | Down |
| ADRB2 | -2.844818 | 6.93E-43 | 2.66E-41 | Down |
| KCNAB1 | -2.103859 | 1.63E-35 | 4.49E-34 | Down |
| SHE | -3.222521 | 3.50E-60 | 2.95E-58 | Down |
| C22orf15 | -2.180695 | 2.16E-14 | 1.40E-13 | Down |
| RSPH10B2 | -2.719604 | 6.40E-20 | 6.92E-19 | Down |
| NPR1 | -3.59957 | 1.41E-83 | 3.48E-81 | Down |
| CD52 | -2.939387 | 2.86E-41 | 1.04E-39 | Down |
| ZEB2 | -2.049407 | 1.60E-40 | 5.63E-39 | Down |
| CLIC3 | -2.553109 | 1.15E-24 | 1.74E-23 | Down |
| GKN1 | -2.698912 | 4.71E-10 | 1.92E-09 | Down |
| GP9 | -4.713216 | 3.76E-66 | 3.93E-64 | Down |
| LDB2 | -3.271425 | 1.02E-119 | 1.54E-116 | Down |
| MYO7B | -2.3039 | 6.15E-15 | 4.21E-14 | Down |
| MYRIP | -3.173074 | 1.34E-39 | 4.53E-38 | Down |
| FAXDC2 | -2.83628 | 3.65E-45 | 1.54E-43 | Down |
| SLN | -2.320981 | 3.89E-12 | 2.01E-11 | Down |
| FABP4 | -4.089117 | 1.11E-31 | 2.53E-30 | Down |
| FRMPD2 | -2.367264 | 2.85E-11 | 1.33E-10 | Down |
| CST5 | -3.545095 | 3.66E-24 | 5.42E-23 | Down |
| TTLL6 | -2.798488 | 8.21E-16 | 6.13E-15 | Down |
| DYDC1 | -2.503287 | 9.04E-10 | 3.57E-09 | Down |
| PLA2G1B | -4.669834 | 8.31E-49 | 4.09E-47 | Down |
| OSCAR | -2.695827 | 8.49E-53 | 5.08E-51 | Down |
| S1PR1 | -3.33661 | 2.91E-90 | 9.75E-88 | Down |
| FPR2 | -3.288521 | 6.30E-37 | 1.85E-35 | Down |
| FPR1 | -2.15469 | 2.25E-20 | 2.49E-19 | Down |
| GIMAP8 | -3.224885 | 1.83E-85 | 4.95E-83 | Down |
| PRKCE | -2.149009 | 5.14E-70 | 6.73E-68 | Down |
| KCNK3 | -3.179434 | 1.90E-27 | 3.40E-26 | Down |
| APLN | -2.862939 | 4.67E-46 | 2.04E-44 | Down |
| GLOD5 | -2.032992 | 1.00E-13 | 6.08E-13 | Down |
| HOPX | -2.457636 | 3.72E-22 | 4.78E-21 | Down |
| OR1L8 | -2.13873 | 7.57E-18 | 6.89E-17 | Down |
| RXFP1 | -2.27757 | 1.25E-11 | 6.08E-11 | Down |
| DNAI2 | -2.784243 | 1.35E-14 | 8.93E-14 | Down |
| NMUR1 | -3.50072 | 1.75E-70 | 2.31E-68 | Down |
| PTCRA | -2.822078 | 3.20E-34 | 8.27E-33 | Down |
| ANO5 | -2.185384 | 8.67E-15 | 5.84E-14 | Down |
| RASGRP4 | -2.664669 | 8.12E-56 | 5.58E-54 | Down |
| KNDC1 | -2.374673 | 1.51E-16 | 1.22E-15 | Down |
| CFAP46 | -2.600401 | 6.29E-16 | 4.74E-15 | Down |
| ANGPTL7 | -4.370829 | 2.88E-43 | 1.12E-41 | Down |
| PRND | -3.151812 | 4.73E-24 | 6.95E-23 | Down |
| KLF17 | -2.682354 | 3.13E-28 | 5.92E-27 | Down |
| AQP4 | -4.087865 | 4.72E-34 | 1.21E-32 | Down |
| FRMD3 | -3.003986 | 5.83E-58 | 4.41E-56 | Down |
| AZU1 | -2.051527 | 9.78E-13 | 5.34E-12 | Down |
| TPSAB1 | -2.01827 | 3.05E-19 | 3.13E-18 | Down |
| NEGR1 | -2.68119 | 2.12E-34 | 5.52E-33 | Down |
| MACROD2 | -2.112168 | 4.36E-15 | 3.03E-14 | Down |
| CLEC12A | -2.449048 | 4.72E-27 | 8.29E-26 | Down |
| PDZD3 | -2.309066 | 3.58E-16 | 2.75E-15 | Down |
| RAB40A | -2.05052 | 1.14E-25 | 1.85E-24 | Down |
| RND1 | -2.712921 | 3.34E-21 | 3.99E-20 | Down |
| COL6A5 | -2.24125 | 8.25E-15 | 5.57E-14 | Down |
| EFCAB12 | -2.478827 | 2.14E-21 | 2.59E-20 | Down |
| HPSE2 | -2.702339 | 9.74E-18 | 8.77E-17 | Down |
| LRRN3 | -3.893974 | 1.84E-60 | 1.57E-58 | Down |
| ABLIM3 | -2.26386 | 1.09E-26 | 1.87E-25 | Down |
| MMRN2 | -2.40502 | 5.09E-41 | 1.82E-39 | Down |
| NDNF | -4.010377 | 7.67E-54 | 4.83E-52 | Down |
| OLR1 | -3.175788 | 8.66E-35 | 2.29E-33 | Down |
| AGR3 | -2.670345 | 2.50E-16 | 1.96E-15 | Down |
| PTPRM | -2.478277 | 1.30E-52 | 7.71E-51 | Down |
| PEAK1 | -2.013759 | 7.72E-59 | 6.09E-57 | Down |
| APOBEC4 | -2.836574 | 5.80E-10 | 2.34E-09 | Down |
| HSPB7 | -2.707617 | 8.25E-37 | 2.41E-35 | Down |
| WFIKKN2 | -2.174205 | 3.70E-10 | 1.52E-09 | Down |
| PIFO | -2.562193 | 2.03E-17 | 1.77E-16 | Down |
| CD34 | -2.573661 | 6.65E-83 | 1.60E-80 | Down |
| MSRB3 | -2.006426 | 4.71E-25 | 7.35E-24 | Down |
| SELP | -2.746249 | 2.56E-34 | 6.62E-33 | Down |
| SLC26A9 | -3.173097 | 8.88E-31 | 1.95E-29 | Down |
| SLCO2A1 | -2.556832 | 1.28E-23 | 1.84E-22 | Down |
| FZD4 | -2.561037 | 2.21E-75 | 3.84E-73 | Down |
| ADGRE1 | -3.010809 | 1.00E-32 | 2.39E-31 | Down |
| DNAH12 | -3.178877 | 1.08E-21 | 1.34E-20 | Down |
| CATSPERD | -2.40755 | 3.08E-09 | 1.14E-08 | Down |
| P2RY14 | -2.240443 | 3.07E-32 | 7.16E-31 | Down |
| DES | -4.245884 | 3.89E-59 | 3.17E-57 | Down |
| VWA3A | -2.811962 | 3.53E-14 | 2.24E-13 | Down |
| GRAMD2A | -2.667422 | 7.87E-36 | 2.21E-34 | Down |
| LPL | -3.786095 | 7.38E-60 | 6.20E-58 | Down |
| LONRF3 | -2.882136 | 1.64E-40 | 5.75E-39 | Down |
| NR2F1 | -2.040672 | 1.20E-33 | 3.00E-32 | Down |
| A2M | -3.265341 | 5.73E-72 | 8.16E-70 | Down |
| CLEC14A | -2.907634 | 4.87E-90 | 1.57E-87 | Down |
| MAP3K19 | -2.787167 | 2.20E-16 | 1.73E-15 | Down |
| NCKAP5 | -3.103578 | 3.31E-41 | 1.19E-39 | Down |
| SCN4B | -2.431898 | 4.92E-27 | 8.62E-26 | Down |
| KCNA3 | -2.728535 | 4.37E-30 | 9.21E-29 | Down |
| MYOZ1 | -3.001211 | 3.18E-31 | 7.18E-30 | Down |
| C2orf73 | -2.519712 | 4.81E-15 | 3.32E-14 | Down |
| HTR3C | -2.401056 | 4.42E-06 | 1.11E-05 | Down |
| PPP1R42 | -2.376118 | 2.63E-11 | 1.24E-10 | Down |
| ZNF366 | -2.521998 | 1.39E-39 | 4.69E-38 | Down |
| SH2D4B | -2.335429 | 5.63E-24 | 8.23E-23 | Down |
| SHISA3 | -2.019989 | 1.73E-07 | 5.19E-07 | Down |
| ERBB4 | -3.046096 | 7.99E-28 | 1.47E-26 | Down |
| LMO7DN | -2.830799 | 2.77E-32 | 6.50E-31 | Down |
| RIIAD1 | -2.417688 | 3.43E-11 | 1.59E-10 | Down |
| TMEM139 | -2.729095 | 2.16E-20 | 2.40E-19 | Down |
| APOLD1 | -2.007758 | 3.35E-30 | 7.13E-29 | Down |
| ERICH3 | -3.02218 | 2.37E-15 | 1.70E-14 | Down |
| C10orf67 | -3.432385 | 3.67E-33 | 8.92E-32 | Down |
| GIMAP7 | -2.480813 | 3.48E-38 | 1.10E-36 | Down |
| TMEM125 | -2.918416 | 5.85E-37 | 1.73E-35 | Down |
| SMCO3 | -2.670458 | 9.22E-42 | 3.40E-40 | Down |
| ALOX15B | -2.476062 | 2.65E-19 | 2.73E-18 | Down |
| FCER1A | -2.136411 | 1.09E-12 | 5.92E-12 | Down |
| CDH5 | -3.423224 | 6.36E-116 | 7.66E-113 | Down |
| FAM216B | -3.348914 | 4.08E-17 | 3.49E-16 | Down |
| MYADM | -2.337964 | 2.54E-32 | 5.98E-31 | Down |
| C1orf194 | -3.014472 | 7.57E-16 | 5.68E-15 | Down |
| ITLN1 | -8.489091 | 7.16E-152 | 4.31E-148 | Down |
| GPBAR1 | -2.801753 | 1.07E-65 | 1.10E-63 | Down |
| TMEM150B | -2.362244 | 4.04E-31 | 9.03E-30 | Down |
| SERTM1 | -4.628485 | 3.38E-36 | 9.70E-35 | Down |
| AGTR2 | -3.037192 | 9.46E-12 | 4.67E-11 | Down |
| MAP3K15 | -2.172205 | 1.82E-15 | 1.32E-14 | Down |
| CXCR2 | -2.076059 | 7.86E-12 | 3.91E-11 | Down |
| GREM2 | -2.502542 | 9.14E-22 | 1.15E-20 | Down |
| CHRM2 | -4.590797 | 9.44E-45 | 3.85E-43 | Down |
| TMEM132C | -2.982561 | 1.80E-11 | 8.64E-11 | Down |
| CFAP65 | -3.052282 | 2.06E-14 | 1.34E-13 | Down |
| AATK | -2.764611 | 3.93E-54 | 2.49E-52 | Down |
| MAB21L2 | -2.091928 | 4.93E-12 | 2.51E-11 | Down |
| TMEM252 | -3.387815 | 7.52E-29 | 1.47E-27 | Down |
| RTKN2 | -4.110881 | 2.74E-162 | 2.47E-158 | Down |
| KCNIP1 | -2.587165 | 3.85E-23 | 5.37E-22 | Down |
| KCNA4 | -2.704609 | 3.42E-08 | 1.12E-07 | Down |
| KIAA2012 | -2.610704 | 7.47E-12 | 3.72E-11 | Down |
| CAV3 | -5.047988 | 2.75E-75 | 4.73E-73 | Down |
| NXPH3 | -2.877151 | 6.43E-45 | 2.67E-43 | Down |
| NTM | -2.433917 | 5.53E-34 | 1.41E-32 | Down |
| C1orf116 | -2.749154 | 2.45E-45 | 1.04E-43 | Down |
| CADM1 | -2.660855 | 1.70E-36 | 4.93E-35 | Down |
| SPNS2 | -2.309296 | 2.02E-33 | 5.00E-32 | Down |
| MCEMP1 | -5.554824 | 6.59E-120 | 1.08E-116 | Down |
| FREM3 | -5.313021 | 2.73E-105 | 1.98E-102 | Down |
| CCBE1 | -3.190803 | 1.88E-31 | 4.26E-30 | Down |
| CABCOCO1 | -2.945227 | 3.80E-21 | 4.53E-20 | Down |
| OVCH2 | -2.327066 | 1.69E-13 | 1.01E-12 | Down |
| SYNDIG1L | -3.414445 | 9.28E-34 | 2.34E-32 | Down |
| ACSM5 | -2.082369 | 1.76E-17 | 1.55E-16 | Down |
| GKN2 | -5.238912 | 3.96E-48 | 1.89E-46 | Down |
| C11orf88 | -2.515125 | 5.42E-15 | 3.72E-14 | Down |
| LHFPL6 | -2.088558 | 1.42E-49 | 7.27E-48 | Down |
| KCTD16 | -2.090675 | 1.41E-13 | 8.49E-13 | Down |
| FAM162B | -3.161533 | 6.39E-65 | 6.38E-63 | Down |
| MAATS1 | -2.315708 | 3.42E-16 | 2.64E-15 | Down |
| ST6GALNAC3 | -2.108619 | 1.73E-18 | 1.67E-17 | Down |
| TMPRSS2 | -2.622528 | 5.01E-25 | 7.77E-24 | Down |
| CLDN5 | -2.914726 | 7.43E-48 | 3.51E-46 | Down |
| PCDH9 | -2.301064 | 5.05E-12 | 2.57E-11 | Down |
| SLIT3 | -3.146651 | 9.93E-49 | 4.86E-47 | Down |
| NCMAP | -2.166235 | 2.88E-18 | 2.70E-17 | Down |
| APOBR | -2.143409 | 1.47E-33 | 3.65E-32 | Down |
| SLC22A10 | -2.477383 | 4.54E-15 | 3.15E-14 | Down |
| F8 | -2.659502 | 2.54E-78 | 4.98E-76 | Down |
| PRSS57 | -2.381563 | 1.75E-14 | 1.14E-13 | Down |
| PPIL6 | -2.144369 | 4.09E-21 | 4.85E-20 | Down |
| KLHL33 | -2.493667 | 2.91E-24 | 4.34E-23 | Down |
| IL3RA | -2.256148 | 1.36E-58 | 1.06E-56 | Down |
| SFTPA2 | -3.993396 | 4.60E-25 | 7.20E-24 | Down |
| ROR1 | -2.930757 | 8.49E-39 | 2.77E-37 | Down |
| MUC1 | -2.01696 | 4.08E-17 | 3.49E-16 | Down |
| MORN5 | -2.781857 | 1.84E-11 | 8.83E-11 | Down |
| NRG3 | -2.820613 | 6.54E-19 | 6.51E-18 | Down |
| CCIN | -2.070781 | 1.95E-21 | 2.38E-20 | Down |
| CD300LF | -2.373069 | 9.29E-32 | 2.13E-30 | Down |
| FFAR4 | -3.552197 | 1.05E-48 | 5.13E-47 | Down |
| SLC51B | -3.260401 | 1.02E-41 | 3.74E-40 | Down |
| TMEM212 | -3.130069 | 1.57E-14 | 1.03E-13 | Down |
| CD300E | -2.097274 | 4.00E-19 | 4.07E-18 | Down |
| GLDN | -3.341562 | 6.11E-75 | 1.02E-72 | Down |
| AKAP14 | -2.288363 | 1.57E-08 | 5.35E-08 | Down |
| RGS7BP | -2.17911 | 7.23E-15 | 4.91E-14 | Down |
| PDE2A | -2.450863 | 4.97E-37 | 1.48E-35 | Down |
| CFAP73 | -2.52839 | 2.29E-15 | 1.64E-14 | Down |
| TMEM232 | -2.390513 | 7.60E-19 | 7.52E-18 | Down |
| KANK3 | -3.360765 | 2.02E-88 | 6.18E-86 | Down |
| LILRA5 | -2.394074 | 2.06E-27 | 3.68E-26 | Down |
| ANGPTL5 | -3.812697 | 3.17E-39 | 1.05E-37 | Down |
| LHFPL3 | -3.307001 | 1.92E-20 | 2.13E-19 | Down |
| C11orf96 | -2.341002 | 1.00E-24 | 1.52E-23 | Down |
| CDHR4 | -2.530238 | 3.44E-10 | 1.42E-09 | Down |
| SPRY4 | -2.160044 | 3.70E-46 | 1.63E-44 | Down |
| TRPV2 | -2.302858 | 4.15E-49 | 2.08E-47 | Down |
| ADH1A | -2.712014 | 4.13E-25 | 6.49E-24 | Down |
| PEAR1 | -2.283847 | 3.35E-35 | 9.08E-34 | Down |
| HELT | -2.580785 | 1.41E-07 | 4.28E-07 | Down |
| FYB2 | -2.984383 | 4.85E-25 | 7.54E-24 | Down |
| LRRC74B | -2.455474 | 4.16E-10 | 1.70E-09 | Down |
| DMBT1 | -2.965436 | 9.84E-15 | 6.57E-14 | Down |
| LCN10 | -2.103207 | 1.94E-06 | 5.09E-06 | Down |
| OVCH1 | -4.875297 | 1.40E-77 | 2.64E-75 | Down |
| NWD1 | -2.017776 | 1.58E-08 | 5.39E-08 | Down |
| RNF133 | -2.037238 | 1.12E-13 | 6.76E-13 | Down |
| SMTNL2 | -2.044673 | 5.43E-11 | 2.47E-10 | Down |
| TCTEX1D4 | -2.329216 | 5.50E-14 | 3.44E-13 | Down |
| INSC | -2.428479 | 1.21E-16 | 9.86E-16 | Down |
| CFAP77 | -2.866565 | 4.86E-16 | 3.70E-15 | Down |
| HBA2 | -3.962553 | 1.29E-49 | 6.65E-48 | Down |
| SAXO2 | -2.282695 | 1.29E-16 | 1.04E-15 | Down |
| PRELP | -2.230775 | 4.57E-23 | 6.34E-22 | Down |
| TMCO2 | -2.888558 | 1.89E-28 | 3.62E-27 | Down |
| SNTN | -2.848353 | 7.60E-14 | 4.68E-13 | Down |
| LRRK2 | -3.381859 | 2.94E-41 | 1.06E-39 | Down |
| C9orf152 | -2.457057 | 3.47E-17 | 2.99E-16 | Down |
| PLAC9 | -3.454273 | 5.92E-95 | 2.74E-92 | Down |
| ALKAL2 | -2.429548 | 5.48E-17 | 4.61E-16 | Down |
| FAM180A | -2.072762 | 1.87E-16 | 1.49E-15 | Down |
| TOGARAM2 | -2.463787 | 2.11E-24 | 3.16E-23 | Down |
| KIAA0408 | -4.53979 | 1.61E-70 | 2.15E-68 | Down |
| S100A4 | -2.398803 | 3.15E-48 | 1.51E-46 | Down |
| FAT4 | -2.112195 | 2.71E-19 | 2.79E-18 | Down |
| KIF19 | -2.189897 | 4.10E-14 | 2.58E-13 | Down |
| HRCT1 | -2.661111 | 3.39E-24 | 5.03E-23 | Down |
| SFTA2 | -3.689734 | 7.31E-24 | 1.06E-22 | Down |
| GIMAP5 | -2.168613 | 6.25E-23 | 8.54E-22 | Down |
| CD55 | -2.382375 | 7.55E-53 | 4.55E-51 | Down |
| ADH1B | -5.086695 | 1.06E-60 | 9.19E-59 | Down |
| FAM180B | -2.007474 | 1.74E-11 | 8.38E-11 | Down |
| DAPK1 | -2.000513 | 1.97E-23 | 2.79E-22 | Down |
| SPINK14 | -2.016977 | 2.21E-08 | 7.39E-08 | Down |
| DTHD1 | -2.607348 | 2.80E-20 | 3.09E-19 | Down |
| SULT1A2 | -2.547343 | 1.85E-22 | 2.42E-21 | Down |
| NEK5 | -2.532265 | 2.57E-30 | 5.51E-29 | Down |
| ADGRA1 | -2.138651 | 1.11E-07 | 3.40E-07 | Down |
| FAM110D | -2.802701 | 4.06E-54 | 2.56E-52 | Down |
| SERPINA1 | -2.408468 | 5.65E-19 | 5.64E-18 | Down |
| KANK2 | -2.471743 | 1.62E-92 | 6.24E-90 | Down |
| GUCA2A | -2.858647 | 2.47E-07 | 7.25E-07 | Down |
| ADARB1 | -2.090977 | 3.60E-35 | 9.73E-34 | Down |
| C5AR1 | -2.242885 | 1.26E-38 | 4.08E-37 | Down |
| VEPH1 | -4.17613 | 4.16E-65 | 4.20E-63 | Down |
| GYPE | -3.498592 | 5.51E-55 | 3.66E-53 | Down |
| SPN | -3.175642 | 7.57E-57 | 5.39E-55 | Down |
| ELANE | -2.689717 | 3.18E-25 | 5.03E-24 | Down |
| DNAH10 | -2.800593 | 6.83E-30 | 1.43E-28 | Down |
| CFAP43 | -2.86372 | 3.96E-23 | 5.52E-22 | Down |
| CFD | -2.695616 | 8.32E-48 | 3.93E-46 | Down |
| MAP1LC3C | -3.486139 | 2.65E-34 | 6.86E-33 | Down |
| CFAP299 | -2.533088 | 2.49E-10 | 1.05E-09 | Down |
| SULT1C4 | -2.011894 | 2.00E-13 | 1.18E-12 | Down |
| HLA-DRB5 | -2.301647 | 8.11E-23 | 1.10E-21 | Down |
| RD3 | -2.047891 | 5.11E-10 | 2.07E-09 | Down |
| SH2D1B | -2.148691 | 2.12E-18 | 2.02E-17 | Down |
| ARC | -2.600705 | 5.48E-22 | 6.96E-21 | Down |
| CCDC69 | -2.064054 | 2.31E-29 | 4.67E-28 | Down |
| PAPSS2 | -2.171583 | 3.79E-24 | 5.60E-23 | Down |
| DUSP27 | -3.344119 | 1.78E-16 | 1.42E-15 | Down |
| ARHGEF15 | -3.203904 | 2.66E-97 | 1.30E-94 | Down |
| GRK5 | -2.624241 | 6.01E-91 | 2.09E-88 | Down |
| CAPN8 | -2.258605 | 1.88E-11 | 8.99E-11 | Down |
| ECT2L | -2.484747 | 3.93E-19 | 4.00E-18 | Down |
| DDO | -2.01497 | 5.71E-18 | 5.25E-17 | Down |
| C1orf141 | -2.66882 | 1.34E-09 | 5.17E-09 | Down |
| LDLRAD1 | -2.850756 | 4.21E-13 | 2.39E-12 | Down |
| LIPN | -2.193665 | 1.61E-12 | 8.62E-12 | Down |
| TMEM273 | -2.234245 | 4.15E-33 | 1.01E-31 | Down |
| GPRIN2 | -2.28501 | 3.84E-22 | 4.92E-21 | Down |
| SYT15 | -2.7045 | 4.16E-33 | 1.01E-31 | Down |
| NOTCH4 | -2.365246 | 3.13E-52 | 1.81E-50 | Down |
| AGER | -5.947575 | 8.52E-111 | 7.34E-108 | Down |
| ERICH2 | -2.098327 | 1.57E-17 | 1.39E-16 | Down |
| CASP12 | -3.734859 | 9.09E-73 | 1.36E-70 | Down |
| MPIG6B | -2.049186 | 1.44E-17 | 1.28E-16 | Down |
| LY6G6F | -2.296976 | 1.77E-08 | 6.02E-08 | Down |
| MAS1L | -3.398514 | 1.55E-20 | 1.75E-19 | Down |
| C9orf135 | -2.501421 | 2.43E-08 | 8.09E-08 | Down |
| TTC25 | -2.066886 | 1.03E-19 | 1.10E-18 | Down |
| ERICH4 | -3.080717 | 2.27E-38 | 7.21E-37 | Down |
| PKHD1L1 | -4.1162 | 1.10E-51 | 6.23E-50 | Down |
| C2orf91 | -3.296051 | 2.61E-27 | 4.63E-26 | Down |
| MT1A | -3.705255 | 5.54E-28 | 1.03E-26 | Down |
| MT1M | -3.550856 | 8.29E-42 | 3.07E-40 | Down |
| CYS1 | -2.274989 | 3.53E-24 | 5.22E-23 | Down |
| CLEC6A | -2.159578 | 3.15E-11 | 1.47E-10 | Down |
| C21orf62 | -3.30568 | 3.42E-34 | 8.79E-33 | Down |
| C18orf63 | -3.285022 | 4.77E-19 | 4.80E-18 | Down |
| RNASE13 | -2.211921 | 1.58E-13 | 9.43E-13 | Down |
| HBA1 | -4.492723 | 6.92E-53 | 4.20E-51 | Down |
| ATP10A | -2.617688 | 1.56E-40 | 5.49E-39 | Down |
| COL6A6 | -3.615731 | 1.11E-46 | 5.01E-45 | Down |
| TRIM71 | -2.541417 | 1.24E-12 | 6.70E-12 | Down |
| GPX3 | -3.190296 | 1.71E-63 | 1.65E-61 | Down |
| TRGJP2 | -2.108641 | 2.89E-06 | 7.40E-06 | Down |
| ACKR1 | -3.224089 | 9.84E-33 | 2.35E-31 | Down |
| GIMAP1 | -2.555272 | 2.28E-44 | 9.22E-43 | Down |
| EMP2 | -2.7089 | 1.84E-91 | 6.51E-89 | Down |
| TMEM213 | -3.03897 | 1.36E-13 | 8.17E-13 | Down |
| SEC14L6 | -3.569888 | 5.27E-50 | 2.78E-48 | Down |
| C5orf49 | -2.518647 | 6.49E-14 | 4.03E-13 | Down |
| C20orf202 | -2.725426 | 4.15E-61 | 3.66E-59 | Down |
| CELA2B | -2.272051 | 2.42E-27 | 4.30E-26 | Down |
| KIR2DS4 | -2.13259 | 3.74E-08 | 1.22E-07 | Down |
| MYBPHL | -2.483325 | 4.65E-11 | 2.13E-10 | Down |
| DCDC2B | -2.473742 | 1.85E-18 | 1.78E-17 | Down |
| PRR29 | -2.182353 | 9.60E-20 | 1.03E-18 | Down |
| CROCC2 | -2.066727 | 3.90E-09 | 1.43E-08 | Down |
| MROH5 | -2.790971 | 6.41E-16 | 4.83E-15 | Down |
| SFTA3 | -3.553002 | 1.34E-22 | 1.78E-21 | Down |
| ANKRD66 | -3.152965 | 9.64E-13 | 5.27E-12 | Down |
| STMND1 | -2.698982 | 3.84E-12 | 1.98E-11 | Down |
| KLHL41 | -2.070098 | 2.21E-16 | 1.74E-15 | Down |
| DEFA3 | -3.129981 | 1.08E-13 | 6.55E-13 | Down |
| LILRA2 | -2.229394 | 3.16E-22 | 4.09E-21 | Down |
| AQP1 | -3.110062 | 1.92E-38 | 6.13E-37 | Down |
| PNMA2 | -2.466387 | 6.80E-28 | 1.25E-26 | Down |
| CD302 | -2.713427 | 1.07E-64 | 1.06E-62 | Down |
| INMT | -4.902153 | 9.38E-119 | 1.30E-115 | Down |
| AKAP2 | -2.974068 | 6.03E-37 | 1.77E-35 | Down |
| PCDHAC2 | -2.368717 | 7.28E-21 | 8.42E-20 | Down |
| WFDC6 | -2.881048 | 1.07E-08 | 3.74E-08 | Down |
| UPK3B | -3.395967 | 1.36E-27 | 2.45E-26 | Down |
| KIR2DL3 | -2.293326 | 6.17E-12 | 3.11E-11 | Down |
| GSTA2 | -2.330904 | 1.89E-07 | 5.64E-07 | Down |
| AL645922.1 | -2.547853 | 1.81E-21 | 2.21E-20 | Down |
| ERVFRD-1 | -2.107866 | 1.17E-09 | 4.55E-09 | Down |
| CCDC13 | -2.253606 | 1.79E-28 | 3.44E-27 | Down |
| HBB | -4.232459 | 3.03E-52 | 1.76E-50 | Down |
| SPATS1 | -2.825959 | 8.61E-09 | 3.04E-08 | Down |
| ECSCR | -3.007042 | 1.12E-82 | 2.66E-80 | Down |
| AC004691.2 | -2.128824 | 1.73E-21 | 2.12E-20 | Down |
| PCDHA12 | -3.074131 | 3.81E-22 | 4.89E-21 | Down |
| INMT-MINDY4 | -4.655495 | 3.01E-62 | 2.78E-60 | Down |
| AL096711.2 | -3.701762 | 1.01E-61 | 9.10E-60 | Down |
| AL928654.3 | -2.980201 | 1.02E-25 | 1.65E-24 | Down |
| RNASE4 | -2.143406 | 3.01E-35 | 8.18E-34 | Down |
| SMIM6 | -2.341334 | 7.75E-14 | 4.77E-13 | Down |
| AL163636.2 | -2.404865 | 1.43E-33 | 3.57E-32 | Down |
| SLC22A31 | -2.355634 | 1.24E-12 | 6.73E-12 | Down |
| MRC1 | -3.775824 | 2.95E-69 | 3.79E-67 | Down |
| AL049634.2 | -2.878251 | 3.00E-32 | 7.00E-31 | Down |
| PECAM1 | -2.916594 | 2.83E-92 | 1.02E-89 | Down |
| MYZAP | -4.187986 | 1.60E-87 | 4.73E-85 | Down |
| GJA5 | -2.09633 | 1.33E-20 | 1.50E-19 | Down |
| GDF10 | -4.892976 | 1.38E-71 | 1.92E-69 | Down |
| FXYD1 | -3.334094 | 3.09E-69 | 3.94E-67 | Down |
| LCN6 | -3.130517 | 1.95E-16 | 1.55E-15 | Down |
| AC008878.3 | -2.133856 | 9.80E-10 | 3.85E-09 | Down |
| ZNF728 | -2.140512 | 3.39E-07 | 9.83E-07 | Down |
| GAS2L2 | -2.717545 | 1.36E-13 | 8.16E-13 | Down |
| FAM47E-STBD1 | -2.519272 | 1.56E-20 | 1.75E-19 | Down |
| GGTLC3 | -3.533634 | 4.29E-22 | 5.49E-21 | Down |
| CCL23 | -3.726531 | 1.17E-52 | 6.98E-51 | Down |
| HNF1B | -3.415086 | 1.04E-25 | 1.69E-24 | Down |
| CCL15-CCL14 | -2.531541 | 6.49E-08 | 2.06E-07 | Down |
| KCNE1B | -2.091438 | 1.86E-09 | 7.06E-09 | Down |
| CCL14 | -3.483815 | 2.88E-37 | 8.70E-36 | Down |
| GPIHBP1 | -4.765946 | 7.05E-82 | 1.61E-79 | Down |
| FO681492.1 | -3.401104 | 7.21E-44 | 2.88E-42 | Down |
| AC006994.2 | -2.310069 | 6.61E-13 | 3.68E-12 | Down |
| PCDH20 | -2.220643 | 1.79E-12 | 9.56E-12 | Down |
| C1orf112 | 2.2972047 | 3.29E-32 | 7.64E-31 | Up |
| GCLC | 3.347386 | 1.71E-17 | 1.50E-16 | Up |
| AOC1 | 2.4157607 | 2.71E-05 | 6.09E-05 | Up |
| WNT16 | 4.1403434 | 5.74E-09 | 2.06E-08 | Up |
| HECW1 | 2.232992 | 1.67E-10 | 7.17E-10 | Up |
| KLHL13 | 2.4997667 | 1.49E-11 | 7.23E-11 | Up |
| FKBP4 | 2.1582789 | 2.00E-25 | 3.21E-24 | Up |
| PRSS22 | 2.193844 | 5.30E-09 | 1.91E-08 | Up |
| HOXA11 | 6.0089381 | 3.36E-19 | 3.43E-18 | Up |
| YBX2 | 4.7111726 | 2.36E-18 | 2.24E-17 | Up |
| KRT33A | 9.5100144 | 1.24E-14 | 8.23E-14 | Up |
| TMEM132A | 3.244994 | 8.60E-40 | 2.93E-38 | Up |
| DLX6 | 8.7051917 | 1.59E-39 | 5.34E-38 | Up |
| CCL26 | 4.0673021 | 3.72E-14 | 2.35E-13 | Up |
| USH1C | 2.1516932 | 0.0004639 | 0.0008718 | Up |
| DBF4 | 2.1338093 | 2.51E-30 | 5.38E-29 | Up |
| NOS2 | 3.3459341 | 1.09E-05 | 2.58E-05 | Up |
| CEACAM7 | 4.5167694 | 7.71E-09 | 2.73E-08 | Up |
| TKTL1 | 3.2373572 | 0.001304 | 0.0022914 | Up |
| E2F2 | 3.1511115 | 2.19E-42 | 8.32E-41 | Up |
| TFAP2B | 5.8867984 | 8.82E-07 | 2.42E-06 | Up |
| TFAP2D | 6.959757 | 1.01E-06 | 2.74E-06 | Up |
| CELSR3 | 2.472492 | 2.64E-14 | 1.70E-13 | Up |
| PLEKHG6 | 2.9742466 | 1.83E-24 | 2.75E-23 | Up |
| MAPK8IP2 | 2.335446 | 1.54E-08 | 5.24E-08 | Up |
| PAX7 | 7.0405891 | 2.22E-08 | 7.43E-08 | Up |
| NCAPD2 | 2.2238989 | 1.30E-27 | 2.35E-26 | Up |
| PRSS3 | 5.8045205 | 7.90E-13 | 4.35E-12 | Up |
| DPF1 | 3.1906357 | 9.98E-14 | 6.07E-13 | Up |
| SYT7 | 2.3311712 | 9.07E-10 | 3.58E-09 | Up |
| ANLN | 4.3179129 | 1.07E-60 | 9.20E-59 | Up |
| GABRA3 | 7.5389383 | 1.41E-41 | 5.20E-40 | Up |
| LTF | 2.4676102 | 2.77E-05 | 6.21E-05 | Up |
| SLC7A14 | 2.4423221 | 0.0037428 | 0.0061706 | Up |
| TACC3 | 2.1941002 | 1.94E-24 | 2.91E-23 | Up |
| STMN4 | 4.7382614 | 1.61E-06 | 4.26E-06 | Up |
| ISL1 | 3.2469132 | 1.45E-05 | 3.38E-05 | Up |
| CLCA4 | 7.4409689 | 1.60E-13 | 9.52E-13 | Up |
| CYP24A1 | 5.5911708 | 4.01E-12 | 2.07E-11 | Up |
| SYT13 | 4.5415459 | 1.05E-07 | 3.25E-07 | Up |
| CPS1 | 3.3199143 | 1.59E-05 | 3.70E-05 | Up |
| ABCC2 | 3.7436199 | 6.64E-08 | 2.10E-07 | Up |
| GCLM | 2.3971511 | 3.10E-10 | 1.29E-09 | Up |
| DEPDC1 | 4.7791757 | 1.56E-68 | 1.82E-66 | Up |
| KCNG1 | 5.6514501 | 7.27E-21 | 8.42E-20 | Up |
| INSRR | 2.0938435 | 1.26E-05 | 2.98E-05 | Up |
| ARNTL2 | 3.3420378 | 1.08E-25 | 1.76E-24 | Up |
| IBSP | 2.6941062 | 5.20E-10 | 2.10E-09 | Up |
| HMGB3 | 2.8795234 | 4.08E-21 | 4.84E-20 | Up |
| DEPDC1B | 4.3948991 | 5.79E-53 | 3.56E-51 | Up |
| SLC18A1 | 2.2094153 | 0.0056988 | 0.0091467 | Up |
| HOXC8 | 3.989517 | 3.37E-15 | 2.38E-14 | Up |
| BEST2 | 3.3991376 | 1.45E-06 | 3.86E-06 | Up |
| TG | 2.6512897 | 1.53E-09 | 5.87E-09 | Up |
| BARX2 | 4.310129 | 1.58E-12 | 8.48E-12 | Up |
| ZIC2 | 7.1340256 | 2.25E-29 | 4.57E-28 | Up |
| DSG2 | 2.1763198 | 7.99E-23 | 1.08E-21 | Up |
| MAGEC2 | 8.9110072 | 1.94E-07 | 5.77E-07 | Up |
| CP | 2.2951208 | 3.02E-06 | 7.71E-06 | Up |
| GUCA1A | 4.0285786 | 3.63E-08 | 1.18E-07 | Up |
| EPN3 | 3.7453634 | 2.93E-32 | 6.85E-31 | Up |
| FOXP3 | 2.1439768 | 1.21E-13 | 7.28E-13 | Up |
| RAD51 | 3.542931 | 5.05E-59 | 4.08E-57 | Up |
| POLQ | 4.6304132 | 3.73E-57 | 2.68E-55 | Up |
| THOC3 | 2.2905467 | 1.25E-22 | 1.67E-21 | Up |
| ALX4 | 3.2833627 | 1.05E-05 | 2.51E-05 | Up |
| MCF2L2 | 2.0752512 | 9.81E-08 | 3.04E-07 | Up |
| ENTPD2 | 2.3626154 | 1.02E-08 | 3.57E-08 | Up |
| PTPRN | 5.2268064 | 1.60E-14 | 1.05E-13 | Up |
| NPFFR2 | 3.6717793 | 0.0004467 | 0.0008411 | Up |
| PHF21B | 2.5489897 | 1.38E-05 | 3.24E-05 | Up |
| SERPINB3 | 4.0147273 | 1.29E-08 | 4.47E-08 | Up |
| F7 | 2.0430755 | 0.0009568 | 0.0017152 | Up |
| LAMC2 | 2.1208487 | 1.45E-06 | 3.85E-06 | Up |
| CAMK2B | 3.867287 | 4.05E-09 | 1.48E-08 | Up |
| STYK1 | 2.4667885 | 2.32E-21 | 2.80E-20 | Up |
| COL11A1 | 6.8451147 | 1.95E-28 | 3.74E-27 | Up |
| SPAG4 | 2.0368245 | 3.17E-13 | 1.84E-12 | Up |
| CDH3 | 3.7996895 | 6.53E-21 | 7.60E-20 | Up |
| GSC2 | 3.9117974 | 6.26E-07 | 1.75E-06 | Up |
| GPC1 | 3.1593158 | 4.99E-20 | 5.43E-19 | Up |
| DLX3 | 2.2740513 | 6.66E-07 | 1.86E-06 | Up |
| DMRT3 | 3.6723689 | 2.12E-08 | 7.09E-08 | Up |
| NGFR | 2.7026958 | 2.13E-07 | 6.31E-07 | Up |
| EYA2 | 3.1098598 | 6.25E-14 | 3.88E-13 | Up |
| MCM10 | 4.3327254 | 1.22E-58 | 9.53E-57 | Up |
| DGKA | 2.0507587 | 4.73E-19 | 4.75E-18 | Up |
| ROPN1 | 4.2947269 | 4.08E-06 | 1.02E-05 | Up |
| SNAP91 | 3.5560295 | 2.61E-06 | 6.73E-06 | Up |
| COL17A1 | 6.1496442 | 4.58E-19 | 4.62E-18 | Up |
| MTHFD2 | 2.0029396 | 4.27E-21 | 5.05E-20 | Up |
| SLC9A3 | 4.0741258 | 4.75E-12 | 2.43E-11 | Up |
| NGEF | 2.849936 | 1.93E-13 | 1.14E-12 | Up |
| ASPM | 4.3233635 | 8.80E-54 | 5.52E-52 | Up |
| MPPED2 | 2.6262577 | 2.06E-08 | 6.91E-08 | Up |
| SYT1 | 2.368869 | 1.16E-08 | 4.05E-08 | Up |
| FGFR3 | 2.4247071 | 6.30E-08 | 2.00E-07 | Up |
| PRR11 | 3.1832703 | 3.50E-49 | 1.76E-47 | Up |
| PITX1 | 6.9149744 | 1.78E-34 | 4.65E-33 | Up |
| GAL | 6.4760723 | 4.31E-18 | 4.00E-17 | Up |
| HES2 | 3.3978147 | 2.33E-14 | 1.51E-13 | Up |
| PFN2 | 3.1672256 | 3.92E-21 | 4.66E-20 | Up |
| ASNS | 2.3484004 | 1.22E-20 | 1.38E-19 | Up |
| CNGB1 | 3.1451625 | 1.89E-07 | 5.65E-07 | Up |
| CHAT | 4.6838583 | 1.04E-06 | 2.83E-06 | Up |
| EPHA8 | 6.0047944 | 9.92E-11 | 4.38E-10 | Up |
| SLC12A3 | 2.5491219 | 0.0001802 | 0.0003602 | Up |
| TRPM5 | 3.9163582 | 4.78E-06 | 1.19E-05 | Up |
| TRIP13 | 3.7569863 | 8.01E-41 | 2.85E-39 | Up |
| MYO3B | 3.7568114 | 3.91E-10 | 1.60E-09 | Up |
| SLC6A15 | 7.2179258 | 7.52E-21 | 8.68E-20 | Up |
| SPP2 | 3.351564 | 9.35E-06 | 2.24E-05 | Up |
| HMMR | 3.591568 | 8.27E-45 | 3.40E-43 | Up |
| CYP2W1 | 4.0842132 | 2.95E-08 | 9.72E-08 | Up |
| MCM2 | 3.4711173 | 1.09E-47 | 5.07E-46 | Up |
| MOV10L1 | 2.0071535 | 2.78E-06 | 7.14E-06 | Up |
| PANX2 | 3.3274206 | 1.68E-14 | 1.10E-13 | Up |
| TP63 | 5.7698796 | 5.52E-28 | 1.02E-26 | Up |
| ADAM11 | 2.4321898 | 1.86E-10 | 7.92E-10 | Up |
| IGF2BP2 | 2.4352056 | 8.92E-12 | 4.41E-11 | Up |
| PPP2R2C | 6.6406185 | 2.03E-26 | 3.43E-25 | Up |
| SNCB | 3.278601 | 9.43E-09 | 3.31E-08 | Up |
| CA12 | 2.5862299 | 1.41E-08 | 4.84E-08 | Up |
| WSCD2 | 2.2918585 | 0.0002453 | 0.000481 | Up |
| KCNQ2 | 4.321905 | 3.48E-06 | 8.82E-06 | Up |
| GTSE1 | 4.1269208 | 6.77E-63 | 6.41E-61 | Up |
| CACNG5 | 2.9119854 | 2.69E-05 | 6.04E-05 | Up |
| FSCN1 | 2.9451888 | 4.56E-22 | 5.81E-21 | Up |
| WDR62 | 3.2437728 | 2.93E-33 | 7.19E-32 | Up |
| PAX2 | 4.0380947 | 3.16E-05 | 7.04E-05 | Up |
| SPAG5 | 3.6628777 | 1.24E-46 | 5.58E-45 | Up |
| ACTL6B | 4.894051 | 1.26E-05 | 2.98E-05 | Up |
| UBE2T | 3.975495 | 1.14E-52 | 6.77E-51 | Up |
| DCX | 5.2657726 | 3.40E-05 | 7.53E-05 | Up |
| SMC1B | 3.3595482 | 2.79E-10 | 1.16E-09 | Up |
| ADCY2 | 2.0642543 | 0.0003308 | 0.0006357 | Up |
| FGF20 | 2.2698492 | 0.0042864 | 0.0070097 | Up |
| TP73 | 2.2382203 | 9.18E-11 | 4.07E-10 | Up |
| DUSP13 | 4.1977137 | 1.86E-10 | 7.94E-10 | Up |
| PAFAH1B3 | 2.6325418 | 1.22E-30 | 2.65E-29 | Up |
| SCGN | 4.7962533 | 0.003381 | 0.0056108 | Up |
| PTPRH | 3.7098581 | 6.94E-15 | 4.72E-14 | Up |
| EPHA6 | 3.3075084 | 7.61E-07 | 2.11E-06 | Up |
| RDH8 | 2.9731888 | 6.44E-05 | 0.0001368 | Up |
| NDC80 | 3.9650841 | 8.27E-55 | 5.45E-53 | Up |
| AFP | 3.5485296 | 1.00E-05 | 2.39E-05 | Up |
| PKP1 | 6.4560911 | 1.69E-30 | 3.64E-29 | Up |
| IL12RB2 | 2.6530152 | 2.47E-10 | 1.04E-09 | Up |
| MPP4 | 2.9396156 | 1.80E-07 | 5.38E-07 | Up |
| COL19A1 | 3.7917894 | 7.08E-07 | 1.97E-06 | Up |
| KCNK2 | 3.1892871 | 1.05E-06 | 2.85E-06 | Up |
| OPRK1 | 2.4978425 | 0.0002799 | 0.0005439 | Up |
| EPYC | 4.9784267 | 1.46E-10 | 6.31E-10 | Up |
| NKAIN1 | 5.1836573 | 1.36E-21 | 1.68E-20 | Up |
| IGSF9 | 4.4866665 | 1.82E-31 | 4.12E-30 | Up |
| ORC1 | 3.6450425 | 2.96E-54 | 1.89E-52 | Up |
| RAD54L | 4.5086441 | 4.84E-66 | 5.03E-64 | Up |
| FOLH1 | 2.2365967 | 5.29E-08 | 1.69E-07 | Up |
| FAT2 | 5.9026814 | 1.11E-24 | 1.68E-23 | Up |
| PPEF1 | 2.4140611 | 1.37E-10 | 5.93E-10 | Up |
| MYBPC2 | 2.1292971 | 3.51E-05 | 7.75E-05 | Up |
| TMPRSS11E | 7.9751105 | 1.07E-21 | 1.33E-20 | Up |
| PTHLH | 6.9444823 | 1.08E-22 | 1.45E-21 | Up |
| TFAP2C | 2.4371084 | 1.96E-21 | 2.38E-20 | Up |
| AURKA | 3.3726746 | 9.30E-55 | 6.07E-53 | Up |
| DNMT3B | 2.917334 | 2.11E-16 | 1.67E-15 | Up |
| TPX2 | 4.9121379 | 5.44E-67 | 5.92E-65 | Up |
| SLC15A1 | 4.6010031 | 1.63E-23 | 2.32E-22 | Up |
| TMEM40 | 4.1327289 | 7.40E-21 | 8.55E-20 | Up |
| SMOX | 2.0182083 | 6.24E-17 | 5.21E-16 | Up |
| SLC4A11 | 3.1842871 | 7.89E-16 | 5.91E-15 | Up |
| LHX5 | 8.764918 | 3.03E-25 | 4.79E-24 | Up |
| CHGB | 4.7758727 | 1.68E-09 | 6.41E-09 | Up |
| FXYD3 | 2.0368997 | 3.22E-08 | 1.06E-07 | Up |
| BIRC5 | 5.0817684 | 9.96E-71 | 1.33E-68 | Up |
| FETUB | 9.4918316 | 1.09E-13 | 6.61E-13 | Up |
| KIF4A | 4.4536764 | 9.56E-76 | 1.68E-73 | Up |
| DLL3 | 7.3211301 | 6.08E-16 | 4.59E-15 | Up |
| NRCAM | 2.7163796 | 1.90E-09 | 7.19E-09 | Up |
| TF | 4.2476191 | 2.13E-07 | 6.31E-07 | Up |
| ORC6 | 4.0873683 | 7.86E-62 | 7.13E-60 | Up |
| RGS17 | 2.3081077 | 1.29E-08 | 4.47E-08 | Up |
| MYH7 | 3.0241564 | 9.75E-07 | 2.66E-06 | Up |
| DAZL | 2.0769227 | 5.45E-05 | 0.0001171 | Up |
| PHGDH | 2.5368079 | 1.00E-16 | 8.23E-16 | Up |
| COL9A3 | 3.491501 | 5.88E-11 | 2.67E-10 | Up |
| CLSPN | 3.1244008 | 1.38E-30 | 3.01E-29 | Up |
| CDC45 | 4.7334556 | 3.39E-60 | 2.87E-58 | Up |
| GABRP | 3.6556228 | 2.66E-07 | 7.79E-07 | Up |
| KRT31 | 10.657708 | 5.62E-17 | 4.72E-16 | Up |
| CDC6 | 4.1905552 | 1.36E-56 | 9.56E-55 | Up |
| TLL2 | 3.6010541 | 3.96E-16 | 3.04E-15 | Up |
| CYP26A1 | 5.6951868 | 2.82E-11 | 1.32E-10 | Up |
| IL11 | 2.1553861 | 2.37E-07 | 6.98E-07 | Up |
| MYO3A | 2.873431 | 1.49E-05 | 3.48E-05 | Up |
| SMIM24 | 2.8397102 | 9.03E-08 | 2.81E-07 | Up |
| DSP | 3.8523009 | 9.69E-36 | 2.70E-34 | Up |
| MAGEB2 | 9.2097892 | 4.38E-09 | 1.60E-08 | Up |
| EFNA2 | 5.8066044 | 1.76E-13 | 1.05E-12 | Up |
| HCN2 | 2.1201698 | 3.64E-10 | 1.50E-09 | Up |
| MMP11 | 6.1151967 | 1.59E-32 | 3.77E-31 | Up |
| CRYBB3 | 2.3864015 | 1.58E-09 | 6.06E-09 | Up |
| SLC16A8 | 2.6267201 | 2.51E-12 | 1.32E-11 | Up |
| CENPM | 3.2697171 | 2.18E-40 | 7.63E-39 | Up |
| 3-Sep | 2.0471001 | 7.54E-09 | 2.68E-08 | Up |
| MIOX | 3.2127968 | 1.88E-08 | 6.33E-08 | Up |
| NEFH | 2.9589784 | 3.05E-05 | 6.80E-05 | Up |
| BIK | 3.0927831 | 2.10E-20 | 2.34E-19 | Up |
| TTLL12 | 2.2905823 | 1.28E-26 | 2.18E-25 | Up |
| CABP7 | 2.6762812 | 9.84E-08 | 3.05E-07 | Up |
| PNPLA5 | 5.2360715 | 3.55E-08 | 1.16E-07 | Up |
| CACNA1I | 2.309829 | 5.51E-07 | 1.55E-06 | Up |
| KCNK10 | 3.4645968 | 2.50E-06 | 6.46E-06 | Up |
| COCH | 4.0347308 | 2.03E-13 | 1.20E-12 | Up |
| POLE2 | 2.7874288 | 6.01E-31 | 1.34E-29 | Up |
| TRIM9 | 2.9875673 | 4.70E-09 | 1.71E-08 | Up |
| CDKN3 | 3.8290009 | 1.19E-46 | 5.34E-45 | Up |
| PLEK2 | 3.154908 | 3.77E-14 | 2.39E-13 | Up |
| ISM2 | 5.7366252 | 3.19E-12 | 1.66E-11 | Up |
| CHGA | 6.4025834 | 7.09E-08 | 2.24E-07 | Up |
| SIX4 | 2.3534394 | 1.14E-20 | 1.30E-19 | Up |
| SERPINA4 | 7.3374069 | 2.03E-08 | 6.84E-08 | Up |
| EFS | 2.0167469 | 1.89E-16 | 1.50E-15 | Up |
| DHRS2 | 4.2794087 | 2.63E-08 | 8.72E-08 | Up |
| MMP9 | 2.5419798 | 9.71E-11 | 4.30E-10 | Up |
| VSX1 | 2.0406743 | 0.0013363 | 0.002346 | Up |
| GINS1 | 4.0895868 | 1.97E-58 | 1.50E-56 | Up |
| MYBL2 | 5.3542792 | 9.72E-74 | 1.52E-71 | Up |
| SALL4 | 4.0455616 | 9.93E-22 | 1.24E-20 | Up |
| BMP7 | 4.1366003 | 3.10E-20 | 3.41E-19 | Up |
| HRH3 | 3.0287765 | 0.0004585 | 0.0008621 | Up |
| NTSR1 | 3.0016816 | 0.0006354 | 0.0011705 | Up |
| NKAIN4 | 3.0416894 | 4.54E-07 | 1.30E-06 | Up |
| COL20A1 | 2.2569058 | 0.0006179 | 0.001141 | Up |
| EEF1A2 | 5.1068542 | 3.48E-11 | 1.61E-10 | Up |
| FERMT1 | 4.385025 | 7.99E-35 | 2.12E-33 | Up |
| PAK5 | 3.076723 | 6.92E-07 | 1.93E-06 | Up |
| JAG1 | 2.248189 | 1.39E-15 | 1.02E-14 | Up |
| E2F1 | 2.0533261 | 6.08E-19 | 6.06E-18 | Up |
| CST4 | 7.8441618 | 8.32E-16 | 6.21E-15 | Up |
| FAM83D | 3.7280174 | 4.01E-36 | 1.14E-34 | Up |
| LAMA1 | 3.4829017 | 1.22E-10 | 5.33E-10 | Up |
| NOL4 | 3.648859 | 0.000242 | 0.000475 | Up |
| H2BFM | 4.5796964 | 8.97E-09 | 3.16E-08 | Up |
| PAGE4 | 4.0897484 | 0.0020124 | 0.0034502 | Up |
| MCF2 | 2.4721254 | 1.16E-05 | 2.76E-05 | Up |
| SCML2 | 2.1981676 | 2.48E-08 | 8.26E-08 | Up |
| GPR50 | 8.1691298 | 2.66E-12 | 1.40E-11 | Up |
| GABRE | 2.5046318 | 2.97E-08 | 9.79E-08 | Up |
| ITIH6 | 3.3545229 | 1.04E-13 | 6.32E-13 | Up |
| CENPI | 3.3942756 | 3.10E-51 | 1.72E-49 | Up |
| DRP2 | 2.4731586 | 4.64E-11 | 2.13E-10 | Up |
| TAF7L | 3.3385678 | 4.38E-10 | 1.79E-09 | Up |
| OLFM4 | 6.3855163 | 1.90E-10 | 8.08E-10 | Up |
| NECAB2 | 3.7332704 | 1.80E-08 | 6.10E-08 | Up |
| SLC7A5 | 3.5960141 | 2.26E-25 | 3.61E-24 | Up |
| RHBDL1 | 2.9937658 | 2.62E-17 | 2.27E-16 | Up |
| SALL1 | 5.8240199 | 7.89E-10 | 3.13E-09 | Up |
| SLC6A2 | 4.6473785 | 3.21E-11 | 1.50E-10 | Up |
| AP3B2 | 3.1992593 | 2.47E-13 | 1.44E-12 | Up |
| TGM5 | 6.0270417 | 1.11E-17 | 9.95E-17 | Up |
| SCG3 | 2.6802506 | 0.0005842 | 0.001082 | Up |
| RHOV | 4.5155441 | 6.28E-30 | 1.32E-28 | Up |
| OIP5 | 3.6985287 | 2.11E-44 | 8.55E-43 | Up |
| TRPA1 | 3.5625762 | 8.11E-09 | 2.87E-08 | Up |
| CALB1 | 7.1240522 | 6.26E-11 | 2.83E-10 | Up |
| DKK4 | 5.173395 | 1.67E-08 | 5.66E-08 | Up |
| NDRG1 | 2.192964 | 9.41E-12 | 4.65E-11 | Up |
| STMN2 | 3.051734 | 4.26E-05 | 9.29E-05 | Up |
| ANXA13 | 3.1690077 | 0.0002836 | 0.0005503 | Up |
| NEFM | 3.5935299 | 5.59E-05 | 0.0001199 | Up |
| MCM4 | 3.0608993 | 6.39E-51 | 3.47E-49 | Up |
| FGL1 | 4.1521486 | 8.61E-05 | 0.0001797 | Up |
| CGB2 | 4.9025812 | 1.27E-06 | 3.40E-06 | Up |
| TUBB4A | 3.5362374 | 2.00E-10 | 8.50E-10 | Up |
| KCNA7 | 3.6866264 | 1.70E-12 | 9.10E-12 | Up |
| CKM | 2.9645445 | 4.45E-06 | 1.11E-05 | Up |
| RNASEH2A | 2.3922994 | 3.19E-30 | 6.78E-29 | Up |
| KLC3 | 3.5627429 | 1.54E-23 | 2.21E-22 | Up |
| AMH | 2.6610559 | 1.96E-06 | 5.14E-06 | Up |
| RSPH6A | 2.4471718 | 0.0002641 | 0.0005151 | Up |
| ASF1B | 3.4587336 | 3.06E-46 | 1.35E-44 | Up |
| TNNT1 | 3.5500807 | 3.01E-11 | 1.41E-10 | Up |
| EPHX3 | 2.0164621 | 7.09E-06 | 1.73E-05 | Up |
| CASP14 | 10.510584 | 5.21E-12 | 2.65E-11 | Up |
| SLC1A6 | 7.6496942 | 1.06E-12 | 5.75E-12 | Up |
| CCNE1 | 4.1476905 | 1.23E-34 | 3.23E-33 | Up |
| CNTD2 | 3.7506643 | 1.15E-11 | 5.66E-11 | Up |
| GPI | 2.0262553 | 7.36E-21 | 8.52E-20 | Up |
| FSD1 | 4.0703331 | 2.12E-15 | 1.52E-14 | Up |
| OVOL3 | 2.280084 | 1.48E-10 | 6.40E-10 | Up |
| CEACAM5 | 3.4434168 | 1.79E-06 | 4.71E-06 | Up |
| CNFN | 5.6110577 | 1.03E-18 | 1.01E-17 | Up |
| GRIN2D | 3.1873242 | 1.05E-12 | 5.74E-12 | Up |
| SYNGR4 | 4.0249843 | 1.48E-09 | 5.70E-09 | Up |
| THEG | 3.9395556 | 2.91E-08 | 9.60E-08 | Up |
| FGF21 | 5.0613782 | 3.20E-08 | 1.05E-07 | Up |
| MAST1 | 3.827394 | 3.53E-21 | 4.21E-20 | Up |
| KCNN1 | 2.6753512 | 4.13E-08 | 1.34E-07 | Up |
| UPK1A | 4.8961921 | 1.33E-07 | 4.04E-07 | Up |
| ATP4A | 7.0885907 | 3.44E-13 | 1.98E-12 | Up |
| TMEM59L | 2.2583964 | 0.0001779 | 0.0003557 | Up |
| ITGB8 | 2.0016402 | 8.26E-11 | 3.69E-10 | Up |
| DLX5 | 6.3601019 | 3.96E-30 | 8.40E-29 | Up |
| STEAP1B | 3.6161415 | 2.69E-13 | 1.57E-12 | Up |
| HOXA1 | 3.0777984 | 2.30E-22 | 3.00E-21 | Up |
| ANKRD7 | 2.5045629 | 9.55E-08 | 2.96E-07 | Up |
| HOXA13 | 7.9724886 | 1.09E-26 | 1.87E-25 | Up |
| EVX1 | 4.9065458 | 1.43E-08 | 4.89E-08 | Up |
| HSPB1 | 2.1325694 | 5.84E-16 | 4.42E-15 | Up |
| NPTX2 | 3.7686915 | 1.39E-08 | 4.77E-08 | Up |
| NUDT1 | 2.0104738 | 6.09E-27 | 1.06E-25 | Up |
| PTPRZ1 | 4.5674985 | 4.17E-17 | 3.56E-16 | Up |
| TFR2 | 3.7051044 | 7.16E-21 | 8.31E-20 | Up |
| EZH2 | 3.3056413 | 5.03E-41 | 1.80E-39 | Up |
| POU6F2 | 5.06349 | 8.73E-21 | 1.00E-19 | Up |
| MYL7 | 3.3760754 | 1.35E-05 | 3.16E-05 | Up |
| LHX2 | 6.7657732 | 1.66E-19 | 1.75E-18 | Up |
| PTGR1 | 2.2522395 | 7.97E-08 | 2.50E-07 | Up |
| AMBP | 3.2393018 | 0.0009839 | 0.0017608 | Up |
| ELAVL2 | 3.0867254 | 3.90E-05 | 8.55E-05 | Up |
| CA9 | 5.6496952 | 4.08E-15 | 2.85E-14 | Up |
| LHX3 | 3.3742552 | 1.30E-05 | 3.07E-05 | Up |
| DNTT | 2.7686165 | 0.0001867 | 0.0003722 | Up |
| ATRNL1 | 2.4579346 | 1.09E-05 | 2.60E-05 | Up |
| TLX1 | 8.0136601 | 7.18E-12 | 3.59E-11 | Up |
| FGF8 | 2.7643678 | 2.61E-05 | 5.87E-05 | Up |
| NPM3 | 2.3729912 | 7.48E-25 | 1.15E-23 | Up |
| PITX3 | 2.4195732 | 1.58E-06 | 4.18E-06 | Up |
| NEURL1 | 2.7662155 | 2.57E-07 | 7.55E-07 | Up |
| UBE2S | 3.17679 | 2.34E-44 | 9.46E-43 | Up |
| LGI1 | 3.5826595 | 0.0044778 | 0.007307 | Up |
| KRT23 | 4.142275 | 7.36E-09 | 2.61E-08 | Up |
| RUNDC3A | 3.3916626 | 4.44E-10 | 1.81E-09 | Up |
| RAPGEFL1 | 3.8569001 | 3.30E-21 | 3.95E-20 | Up |
| KRT37 | 5.7662319 | 1.69E-09 | 6.45E-09 | Up |
| TRIM16L | 3.4528713 | 7.50E-13 | 4.15E-12 | Up |
| ALDH3A1 | 3.9642904 | 5.10E-08 | 1.64E-07 | Up |
| KRT32 | 4.5872027 | 3.73E-13 | 2.14E-12 | Up |
| COL1A1 | 2.0909744 | 3.44E-08 | 1.13E-07 | Up |
| PTGES3L-AARSD1 | 2.789316 | 1.39E-10 | 6.03E-10 | Up |
| RND2 | 2.0915414 | 4.12E-07 | 1.18E-06 | Up |
| ALOX12 | 2.0970527 | 1.11E-06 | 3.01E-06 | Up |
| SLC9A3R1 | 2.603403 | 7.38E-19 | 7.31E-18 | Up |
| FOXN1 | 4.950942 | 1.57E-15 | 1.15E-14 | Up |
| GNRHR | 2.0779851 | 0.0001821 | 0.0003636 | Up |
| SULT1E1 | 2.2048238 | 0.0011812 | 0.0020891 | Up |
| NMU | 4.9951768 | 1.15E-17 | 1.03E-16 | Up |
| ANXA10 | 5.9755501 | 9.02E-09 | 3.17E-08 | Up |
| NEIL3 | 4.338773 | 5.04E-45 | 2.11E-43 | Up |
| NKX3-2 | 4.5756158 | 3.90E-17 | 3.34E-16 | Up |
| HGFAC | 2.2841155 | 1.39E-05 | 3.25E-05 | Up |
| NCAPG | 3.8551411 | 7.06E-64 | 6.90E-62 | Up |
| CCDC34 | 2.5201942 | 3.25E-32 | 7.56E-31 | Up |
| CCKBR | 3.8923498 | 5.19E-05 | 0.0001118 | Up |
| UPK2 | 4.1274786 | 8.20E-08 | 2.57E-07 | Up |
| NECTIN1 | 4.503972 | 1.51E-38 | 4.83E-37 | Up |
| KIAA1549L | 4.7979751 | 7.39E-15 | 5.01E-14 | Up |
| MDK | 2.7270285 | 3.78E-18 | 3.52E-17 | Up |
| CALCA | 5.9545648 | 5.21E-05 | 0.0001122 | Up |
| ASIC1 | 2.1098847 | 7.16E-14 | 4.42E-13 | Up |
| IL23A | 2.4019508 | 2.97E-10 | 1.24E-09 | Up |
| CYP27B1 | 2.1456225 | 4.85E-14 | 3.04E-13 | Up |
| MYF6 | 2.4660995 | 1.25E-05 | 2.95E-05 | Up |
| TRPV4 | 2.1273978 | 2.90E-13 | 1.68E-12 | Up |
| FOXM1 | 4.7708122 | 5.00E-59 | 4.06E-57 | Up |
| RAD51AP1 | 3.3478171 | 6.04E-40 | 2.07E-38 | Up |
| RASAL1 | 4.5525114 | 8.55E-34 | 2.16E-32 | Up |
| FZD10 | 3.1060041 | 7.72E-10 | 3.07E-09 | Up |
| TIMELESS | 2.4011034 | 6.57E-42 | 2.44E-40 | Up |
| GAPDH | 2.6242784 | 3.20E-35 | 8.69E-34 | Up |
| CDCA3 | 4.4649015 | 1.11E-69 | 1.43E-67 | Up |
| SLCO1B3 | 6.7106052 | 1.21E-10 | 5.30E-10 | Up |
| RFX4 | 3.2313035 | 0.0014985 | 0.0026165 | Up |
| TPD52L1 | 2.4006015 | 1.97E-14 | 1.28E-13 | Up |
| FANCE | 2.2186889 | 2.38E-26 | 4.01E-25 | Up |
| TULP1 | 3.0661444 | 1.37E-15 | 1.00E-14 | Up |
| IL17A | 2.7971184 | 0.0006466 | 0.0011899 | Up |
| PRDM13 | 7.4120975 | 4.39E-19 | 4.44E-18 | Up |
| COL9A1 | 2.8746509 | 2.72E-05 | 6.11E-05 | Up |
| WASF1 | 2.7050378 | 2.38E-18 | 2.26E-17 | Up |
| GMNN | 2.2379868 | 5.37E-27 | 9.40E-26 | Up |
| NR2E1 | 6.4615151 | 3.04E-11 | 1.42E-10 | Up |
| PERP | 3.5011494 | 4.35E-36 | 1.24E-34 | Up |
| UNC93A | 6.532071 | 4.38E-12 | 2.25E-11 | Up |
| MDFI | 2.2625145 | 4.36E-16 | 3.34E-15 | Up |
| TTK | 4.7170951 | 5.96E-63 | 5.67E-61 | Up |
| WISP3 | 2.7216572 | 7.28E-07 | 2.02E-06 | Up |
| KIF20A | 3.9652579 | 8.54E-60 | 7.15E-58 | Up |
| GABRG2 | 7.6065413 | 1.24E-10 | 5.43E-10 | Up |
| LMNB1 | 2.2180267 | 1.16E-27 | 2.11E-26 | Up |
| IRX4 | 9.216841 | 1.16E-18 | 1.13E-17 | Up |
| C9 | 2.2082589 | 2.27E-05 | 5.13E-05 | Up |
| CDX1 | 3.231981 | 1.02E-10 | 4.49E-10 | Up |
| HRG | 4.7866515 | 1.13E-09 | 4.38E-09 | Up |
| HGD | 2.6929906 | 0.0012827 | 0.0022561 | Up |
| CLDN16 | 2.1698586 | 7.48E-06 | 1.82E-05 | Up |
| RBP1 | 2.4256892 | 1.55E-09 | 5.94E-09 | Up |
| GRK7 | 3.3388344 | 2.11E-13 | 1.24E-12 | Up |
| WNT5A | 2.1866746 | 8.35E-09 | 2.95E-08 | Up |
| COL7A1 | 6.0670958 | 1.81E-31 | 4.12E-30 | Up |
| FGF12 | 2.1975589 | 2.39E-07 | 7.04E-07 | Up |
| ECT2 | 3.1199073 | 1.52E-38 | 4.86E-37 | Up |
| GNAT1 | 2.3674718 | 0.0001526 | 0.000308 | Up |
| MORC1 | 4.6825643 | 0.0002214 | 0.0004368 | Up |
| PODXL2 | 3.175824 | 1.61E-20 | 1.80E-19 | Up |
| UPK1B | 4.3126426 | 7.98E-07 | 2.20E-06 | Up |
| ABCC5 | 3.5304851 | 1.18E-19 | 1.26E-18 | Up |
| CLCN2 | 2.5179523 | 2.01E-21 | 2.44E-20 | Up |
| SLC4A3 | 2.4227491 | 7.94E-16 | 5.93E-15 | Up |
| ADAM23 | 4.4968912 | 1.50E-12 | 8.08E-12 | Up |
| POMC | 2.8242779 | 6.17E-08 | 1.96E-07 | Up |
| CENPA | 4.9056336 | 1.93E-72 | 2.77E-70 | Up |
| SLC30A3 | 2.4374873 | 1.74E-06 | 4.61E-06 | Up |
| TLX2 | 4.5906423 | 9.30E-13 | 5.09E-12 | Up |
| REG1A | 3.7827529 | 1.87E-06 | 4.91E-06 | Up |
| IGFBP2 | 3.3063094 | 8.91E-15 | 5.99E-14 | Up |
| OTX1 | 4.5788275 | 4.61E-25 | 7.21E-24 | Up |
| PRKAG3 | 2.0604026 | 0.0031844 | 0.0053042 | Up |
| WNT6 | 4.5664325 | 1.01E-10 | 4.45E-10 | Up |
| SLC9A2 | 4.2229679 | 1.88E-14 | 1.22E-13 | Up |
| ABCB6 | 2.5703623 | 3.01E-18 | 2.83E-17 | Up |
| PROC | 3.0599955 | 2.76E-10 | 1.15E-09 | Up |
| DLX2 | 7.4629156 | 4.94E-15 | 3.40E-14 | Up |
| LCT | 2.1908149 | 8.54E-05 | 0.0001784 | Up |
| SLC1A4 | 2.3085656 | 3.60E-17 | 3.10E-16 | Up |
| KISS1R | 3.8937108 | 8.74E-15 | 5.89E-14 | Up |
| MARK1 | 2.6196984 | 1.66E-23 | 2.36E-22 | Up |
| RPE65 | 3.2028193 | 6.59E-06 | 1.61E-05 | Up |
| AGMAT | 2.9533349 | 3.09E-20 | 3.40E-19 | Up |
| MYCL | 2.8245591 | 9.74E-10 | 3.83E-09 | Up |
| MFAP2 | 2.1740298 | 9.50E-15 | 6.36E-14 | Up |
| ACTL8 | 9.1665676 | 4.83E-12 | 2.46E-11 | Up |
| SLC2A1 | 5.8863252 | 6.49E-48 | 3.08E-46 | Up |
| CDC20 | 4.8974457 | 2.10E-78 | 4.17E-76 | Up |
| ARTN | 5.5749825 | 1.08E-29 | 2.23E-28 | Up |
| PTCH2 | 2.8662487 | 3.96E-05 | 8.67E-05 | Up |
| FMO6P | 3.1103327 | 2.55E-06 | 6.57E-06 | Up |
| IRF6 | 2.8505422 | 1.12E-25 | 1.81E-24 | Up |
| SERPINC1 | 2.2288331 | 0.0019172 | 0.0032976 | Up |
| NEK2 | 4.7756528 | 2.03E-67 | 2.22E-65 | Up |
| CENPF | 4.2255178 | 7.86E-50 | 4.09E-48 | Up |
| CHRNB4 | 5.6054084 | 6.30E-31 | 1.40E-29 | Up |
| APOA1 | 5.1338517 | 6.66E-05 | 0.0001413 | Up |
| SLC8A2 | 3.137829 | 3.69E-07 | 1.07E-06 | Up |
| KIF14 | 4.4894956 | 1.34E-57 | 9.87E-56 | Up |
| ATP10B | 4.2442863 | 2.06E-18 | 1.97E-17 | Up |
| ELOVL4 | 3.3123652 | 7.77E-16 | 5.82E-15 | Up |
| ALDH8A1 | 2.1804274 | 0.0003431 | 0.000658 | Up |
| OLFM3 | 5.228936 | 5.36E-05 | 0.0001153 | Up |
| SPP1 | 4.8767469 | 5.25E-19 | 5.26E-18 | Up |
| GDA | 4.6327475 | 2.39E-12 | 1.26E-11 | Up |
| TRIM67 | 2.4690566 | 4.23E-08 | 1.37E-07 | Up |
| ONECUT2 | 4.9404834 | 2.94E-17 | 2.55E-16 | Up |
| PGF | 2.6603755 | 3.97E-15 | 2.77E-14 | Up |
| ESRRB | 2.4875999 | 0.0001995 | 0.0003962 | Up |
| BCL11A | 3.428257 | 7.74E-22 | 9.74E-21 | Up |
| NKX2-3 | 3.3800595 | 1.22E-08 | 4.23E-08 | Up |
| CNNM1 | 2.9892123 | 2.52E-10 | 1.06E-09 | Up |
| HELLS | 3.2070906 | 1.97E-45 | 8.47E-44 | Up |
| HOXB8 | 4.366107 | 4.90E-08 | 1.58E-07 | Up |
| CRHR1 | 3.2465643 | 4.41E-06 | 1.10E-05 | Up |
| HOXB1 | 2.8343097 | 1.06E-05 | 2.52E-05 | Up |
| MSX2 | 2.091883 | 5.70E-07 | 1.61E-06 | Up |
| GRIA2 | 4.5520616 | 1.41E-06 | 3.76E-06 | Up |
| CENPL | 2.1193341 | 2.41E-27 | 4.28E-26 | Up |
| TNFSF18 | 2.1842765 | 1.75E-05 | 4.03E-05 | Up |
| TP53AIP1 | 4.2638741 | 1.87E-10 | 7.97E-10 | Up |
| TEX11 | 3.3769663 | 1.57E-10 | 6.75E-10 | Up |
| TNFSF11 | 3.3139897 | 7.84E-16 | 5.87E-15 | Up |
| SOHLH2 | 3.7981401 | 4.53E-09 | 1.65E-08 | Up |
| NCAPH | 4.2941036 | 4.39E-73 | 6.72E-71 | Up |
| MND1 | 3.6686373 | 7.80E-52 | 4.48E-50 | Up |
| PRB2 | 2.3791396 | 0.0005234 | 0.0009772 | Up |
| IAPP | 4.5188485 | 2.57E-08 | 8.53E-08 | Up |
| RGSL1 | 3.4285211 | 9.36E-06 | 2.24E-05 | Up |
| CSTA | 3.4455037 | 3.69E-13 | 2.12E-12 | Up |
| B4GALT4 | 2.190072 | 3.53E-15 | 2.48E-14 | Up |
| KIF18A | 2.2195992 | 5.60E-26 | 9.25E-25 | Up |
| DEPDC7 | 2.6549559 | 2.07E-16 | 1.63E-15 | Up |
| GJB6 | 9.2934497 | 6.41E-29 | 1.26E-27 | Up |
| GJA3 | 5.14258 | 3.91E-18 | 3.64E-17 | Up |
| HPCA | 2.8178931 | 9.70E-13 | 5.30E-12 | Up |
| PAEP | 5.5537957 | 8.77E-08 | 2.73E-07 | Up |
| NXPH1 | 4.9797198 | 2.01E-09 | 7.57E-09 | Up |
| NPY | 6.1550306 | 0.0001012 | 0.0002089 | Up |
| TWIST1 | 3.3528572 | 2.44E-13 | 1.43E-12 | Up |
| PHF24 | 2.7597914 | 2.24E-06 | 5.82E-06 | Up |
| NUDT10 | 2.3147258 | 5.17E-06 | 1.28E-05 | Up |
| NEUROG3 | 5.1175783 | 0.0005445 | 0.0010137 | Up |
| PLAU | 2.738565 | 3.21E-14 | 2.05E-13 | Up |
| ZWINT | 3.3457387 | 8.42E-56 | 5.77E-54 | Up |
| CENPK | 2.6310965 | 2.20E-27 | 3.93E-26 | Up |
| HOXC13 | 9.5198418 | 8.27E-44 | 3.29E-42 | Up |
| HOXC11 | 8.6032377 | 2.81E-21 | 3.37E-20 | Up |
| HOXC12 | 7.5702353 | 3.06E-08 | 1.01E-07 | Up |
| STIL | 3.1959643 | 4.20E-55 | 2.80E-53 | Up |
| HJURP | 4.7886281 | 3.55E-74 | 5.73E-72 | Up |
| COL10A1 | 4.1426841 | 7.26E-20 | 7.83E-19 | Up |
| PLP1 | 2.8402716 | 0.0019678 | 0.0033809 | Up |
| H2BFWT | 5.3305499 | 7.77E-14 | 4.78E-13 | Up |
| NRK | 2.0516012 | 7.49E-05 | 0.000158 | Up |
| MAGEA9 | 7.8212176 | 6.08E-07 | 1.71E-06 | Up |
| ACVR1C | 3.0636619 | 1.16E-15 | 8.53E-15 | Up |
| RAB38 | 2.1492452 | 9.23E-10 | 3.64E-09 | Up |
| GPR83 | 2.5846037 | 4.22E-06 | 1.06E-05 | Up |
| INHA | 3.284152 | 2.13E-09 | 8.02E-09 | Up |
| CTCFL | 6.4897841 | 8.98E-07 | 2.46E-06 | Up |
| PI3 | 6.429795 | 7.51E-14 | 4.63E-13 | Up |
| ARHGAP40 | 2.3182926 | 6.18E-06 | 1.52E-05 | Up |
| MATN4 | 2.1600664 | 0.0003089 | 0.0005961 | Up |
| GTSF1L | 2.6178661 | 4.27E-07 | 1.22E-06 | Up |
| RBPJL | 2.182199 | 0.000126 | 0.0002569 | Up |
| MAGEA10 | 9.921671 | 1.00E-11 | 4.93E-11 | Up |
| XG | 2.7355136 | 1.78E-09 | 6.75E-09 | Up |
| POF1B | 2.7176268 | 1.84E-10 | 7.85E-10 | Up |
| LYPD3 | 6.3985658 | 5.85E-31 | 1.30E-29 | Up |
| NDP | 2.0282936 | 6.53E-05 | 0.0001387 | Up |
| GRM4 | 4.6867553 | 7.99E-13 | 4.40E-12 | Up |
| PACSIN1 | 2.367487 | 5.33E-08 | 1.71E-07 | Up |
| BTN1A1 | 2.6356554 | 0.0002472 | 0.0004846 | Up |
| HIST1H1D | 3.3627165 | 6.67E-15 | 4.55E-14 | Up |
| HIST1H2BJ | 2.7569424 | 2.54E-12 | 1.34E-11 | Up |
| OR2B6 | 4.4466039 | 1.38E-13 | 8.32E-13 | Up |
| CXCL6 | 2.6321186 | 3.54E-05 | 7.81E-05 | Up |
| PIWIL1 | 4.5020228 | 3.12E-06 | 7.95E-06 | Up |
| SOX21 | 4.8010433 | 1.49E-15 | 1.09E-14 | Up |
| C17orf53 | 3.5714134 | 1.79E-45 | 7.71E-44 | Up |
| SOX9 | 2.0642167 | 1.49E-08 | 5.10E-08 | Up |
| C1orf61 | 4.9641514 | 1.80E-09 | 6.84E-09 | Up |
| CD70 | 2.6518525 | 7.22E-06 | 1.76E-05 | Up |
| TGM3 | 4.2541491 | 5.85E-07 | 1.65E-06 | Up |
| DEFB126 | 6.7765439 | 5.02E-17 | 4.25E-16 | Up |
| PAX1 | 3.1380241 | 0.0001795 | 0.0003588 | Up |
| CST8 | 5.5234916 | 5.19E-07 | 1.47E-06 | Up |
| NKX2-4 | 6.2571571 | 6.64E-07 | 1.85E-06 | Up |
| NKX2-2 | 6.7174605 | 2.97E-06 | 7.59E-06 | Up |
| CSTL1 | 5.2719132 | 2.35E-14 | 1.52E-13 | Up |
| CST11 | 4.2367077 | 1.23E-08 | 4.25E-08 | Up |
| MCM8 | 2.2367365 | 3.02E-32 | 7.04E-31 | Up |
| FAM83C | 9.5261615 | 4.88E-21 | 5.74E-20 | Up |
| PROZ | 2.0518937 | 1.01E-05 | 2.41E-05 | Up |
| SLURP1 | 6.546849 | 1.59E-11 | 7.69E-11 | Up |
| KIRREL2 | 3.4050122 | 1.70E-07 | 5.10E-07 | Up |
| KRT36 | 3.8206525 | 4.94E-09 | 1.79E-08 | Up |
| FLRT1 | 2.3975713 | 2.50E-10 | 1.05E-09 | Up |
| STATH | 9.4869626 | 5.71E-07 | 1.61E-06 | Up |
| PRKCG | 2.2551907 | 3.19E-06 | 8.11E-06 | Up |
| SSX1 | 5.1959119 | 2.81E-06 | 7.21E-06 | Up |
| SIX1 | 2.450112 | 5.86E-13 | 3.28E-12 | Up |
| DLGAP5 | 4.6326227 | 5.54E-74 | 8.70E-72 | Up |
| CTAG2 | 6.3106682 | 9.63E-05 | 0.0001996 | Up |
| EDN2 | 2.105169 | 0.000144 | 0.0002914 | Up |
| HRASLS | 4.0954447 | 8.39E-16 | 6.26E-15 | Up |
| BEST3 | 5.1764217 | 1.18E-06 | 3.18E-06 | Up |
| AUNIP | 3.7726962 | 2.00E-53 | 1.24E-51 | Up |
| OR7C1 | 4.373338 | 4.73E-12 | 2.42E-11 | Up |
| SYNGR3 | 2.330232 | 6.11E-09 | 2.19E-08 | Up |
| PKMYT1 | 4.3452368 | 4.56E-74 | 7.22E-72 | Up |
| CHTF18 | 2.6467789 | 3.37E-29 | 6.73E-28 | Up |
| GNG13 | 2.7992117 | 1.20E-05 | 2.83E-05 | Up |
| VIL1 | 6.161562 | 4.53E-08 | 1.46E-07 | Up |
| GNGT1 | 7.6770991 | 3.16E-51 | 1.74E-49 | Up |
| LRFN1 | 2.0093115 | 6.40E-12 | 3.22E-11 | Up |
| SPINK2 | 2.2368153 | 4.24E-05 | 9.25E-05 | Up |
| PAICS | 2.1603121 | 3.68E-28 | 6.93E-27 | Up |
| PPAT | 2.1766101 | 1.08E-29 | 2.22E-28 | Up |
| ADM2 | 3.185281 | 1.89E-21 | 2.30E-20 | Up |
| RFPL1 | 3.52399 | 7.62E-06 | 1.85E-05 | Up |
| MCHR1 | 2.0957283 | 1.37E-06 | 3.66E-06 | Up |
| BAIAP2L2 | 3.4426572 | 7.85E-22 | 9.87E-21 | Up |
| GALR3 | 2.740542 | 2.86E-06 | 7.33E-06 | Up |
| APOL5 | 2.1532438 | 0.0049171 | 0.0079698 | Up |
| IGLL1 | 3.0644217 | 1.06E-06 | 2.87E-06 | Up |
| KRT17 | 7.175216 | 3.27E-29 | 6.55E-28 | Up |
| VGF | 4.7467555 | 2.23E-08 | 7.48E-08 | Up |
| SMO | 2.3408895 | 1.40E-17 | 1.24E-16 | Up |
| FEZF1 | 6.0973894 | 3.40E-15 | 2.40E-14 | Up |
| GAD1 | 4.3465124 | 6.37E-17 | 5.32E-16 | Up |
| HOXD9 | 2.5060802 | 2.75E-09 | 1.03E-08 | Up |
| HOXD10 | 5.7963289 | 2.91E-19 | 3.00E-18 | Up |
| HOXD11 | 9.7929434 | 2.89E-33 | 7.10E-32 | Up |
| HOXD13 | 9.8207217 | 9.49E-22 | 1.19E-20 | Up |
| BBOX1 | 2.5262274 | 2.58E-08 | 8.57E-08 | Up |
| MYOD1 | 8.3489897 | 2.10E-08 | 7.05E-08 | Up |
| KCNC1 | 2.795981 | 1.22E-06 | 3.28E-06 | Up |
| TPH1 | 4.2473725 | 4.57E-06 | 1.14E-05 | Up |
| E2F8 | 3.3174196 | 6.02E-38 | 1.87E-36 | Up |
| SOX15 | 4.7387925 | 3.30E-22 | 4.28E-21 | Up |
| PIMREG | 4.3428754 | 8.12E-45 | 3.35E-43 | Up |
| KLK14 | 2.7810452 | 2.46E-05 | 5.54E-05 | Up |
| KLK8 | 5.4448047 | 4.97E-10 | 2.02E-09 | Up |
| EGLN3 | 3.6341439 | 1.15E-17 | 1.03E-16 | Up |
| SGO1 | 3.8924457 | 1.61E-54 | 1.04E-52 | Up |
| LBP | 2.7499757 | 9.56E-05 | 0.0001981 | Up |
| SYT5 | 4.2021116 | 3.04E-09 | 1.13E-08 | Up |
| TNNI3 | 2.6854362 | 1.36E-06 | 3.63E-06 | Up |
| FAM155B | 4.1260214 | 1.90E-15 | 1.37E-14 | Up |
| GDPD2 | 4.848196 | 1.92E-11 | 9.16E-11 | Up |
| NCAN | 3.7418173 | 8.15E-08 | 2.55E-07 | Up |
| KIF1A | 6.1250411 | 4.00E-11 | 1.85E-10 | Up |
| FUT5 | 2.3551895 | 9.88E-05 | 0.0002043 | Up |
| EPO | 2.6085262 | 1.27E-07 | 3.89E-07 | Up |
| KLHDC7B | 3.0808327 | 1.17E-08 | 4.07E-08 | Up |
| SULT4A1 | 5.2454711 | 1.84E-16 | 1.47E-15 | Up |
| MNX1 | 2.6567352 | 7.41E-07 | 2.06E-06 | Up |
| RBBP8NL | 3.3989749 | 4.91E-16 | 3.74E-15 | Up |
| PRDM12 | 2.4171035 | 3.11E-09 | 1.15E-08 | Up |
| SLC6A8 | 4.7194299 | 4.56E-35 | 1.23E-33 | Up |
| PNCK | 6.6462394 | 6.15E-28 | 1.14E-26 | Up |
| DUSP9 | 7.3856103 | 1.07E-35 | 2.98E-34 | Up |
| SLC7A10 | 2.5890236 | 0.0001056 | 0.0002174 | Up |
| HSD17B3 | 3.0144184 | 3.38E-08 | 1.11E-07 | Up |
| ULBP2 | 2.749812 | 6.73E-12 | 3.38E-11 | Up |
| C1QL1 | 3.6983412 | 9.91E-14 | 6.03E-13 | Up |
| GFAP | 3.1175481 | 2.16E-06 | 5.62E-06 | Up |
| TEX101 | 4.3228361 | 2.33E-08 | 7.79E-08 | Up |
| CCL25 | 3.6251724 | 5.44E-08 | 1.74E-07 | Up |
| GINS2 | 3.8053497 | 6.40E-40 | 2.19E-38 | Up |
| SLC34A1 | 3.0490458 | 4.36E-11 | 2.01E-10 | Up |
| F12 | 3.8593315 | 7.94E-29 | 1.55E-27 | Up |
| PRR7 | 2.1199969 | 3.46E-17 | 2.98E-16 | Up |
| KREMEN2 | 6.1252913 | 6.65E-24 | 9.69E-23 | Up |
| BARX1 | 9.1829813 | 9.65E-22 | 1.21E-20 | Up |
| CA6 | 3.9847684 | 1.04E-06 | 2.83E-06 | Up |
| KRT34 | 6.215474 | 6.24E-15 | 4.27E-14 | Up |
| KRT33B | 5.1974693 | 2.09E-09 | 7.90E-09 | Up |
| TNS4 | 5.4369302 | 3.19E-29 | 6.39E-28 | Up |
| TOP2A | 4.5519055 | 8.03E-53 | 4.82E-51 | Up |
| LIN28A | 3.3840356 | 0.0012985 | 0.0022827 | Up |
| LRRC9 | 2.4341099 | 1.69E-08 | 5.73E-08 | Up |
| PODNL1 | 2.1339481 | 1.11E-10 | 4.89E-10 | Up |
| RTBDN | 5.4576523 | 8.09E-13 | 4.45E-12 | Up |
| SLC6A11 | 4.8911685 | 3.42E-10 | 1.41E-09 | Up |
| POPDC3 | 2.7669398 | 6.91E-07 | 1.93E-06 | Up |
| ITGB4 | 2.945818 | 8.21E-20 | 8.82E-19 | Up |
| SLC52A1 | 2.5509448 | 1.49E-10 | 6.42E-10 | Up |
| REEP2 | 2.2181571 | 4.69E-09 | 1.71E-08 | Up |
| RHBG | 4.4474158 | 4.25E-12 | 2.19E-11 | Up |
| BCAN | 3.6887369 | 9.15E-08 | 2.84E-07 | Up |
| ALDH3B2 | 5.2340552 | 7.57E-30 | 1.58E-28 | Up |
| TESMIN | 3.6703079 | 9.49E-26 | 1.54E-24 | Up |
| ANGPTL3 | 3.6176117 | 2.40E-06 | 6.20E-06 | Up |
| TPTE2 | 2.1597157 | 8.73E-07 | 2.40E-06 | Up |
| RNF17 | 2.9151435 | 1.59E-08 | 5.41E-08 | Up |
| CCNA1 | 2.3424663 | 5.82E-05 | 0.0001245 | Up |
| IRS4 | 5.6720954 | 2.15E-06 | 5.61E-06 | Up |
| BEX1 | 2.0623524 | 0.0057648 | 0.009246 | Up |
| EPHB2 | 2.1035783 | 6.81E-10 | 2.73E-09 | Up |
| MYO18B | 3.810461 | 1.03E-08 | 3.59E-08 | Up |
| C1QTNF6 | 2.3398867 | 8.56E-19 | 8.43E-18 | Up |
| FAM83F | 4.7843966 | 2.91E-32 | 6.81E-31 | Up |
| NTS | 6.5293065 | 1.02E-09 | 3.99E-09 | Up |
| SPINK5 | 2.0023166 | 2.86E-05 | 6.40E-05 | Up |
| TEX15 | 4.1643219 | 2.70E-07 | 7.90E-07 | Up |
| GSC | 2.889175 | 1.77E-07 | 5.31E-07 | Up |
| VRTN | 3.6217537 | 8.91E-05 | 0.0001856 | Up |
| ADAMDEC1 | 3.2423121 | 3.38E-14 | 2.15E-13 | Up |
| CCNB1 | 3.8300539 | 4.31E-63 | 4.15E-61 | Up |
| GSTM1 | 3.2145068 | 0.0004543 | 0.0008544 | Up |
| REG4 | 4.495823 | 5.00E-05 | 0.0001081 | Up |
| PSRC1 | 2.1620626 | 6.00E-27 | 1.05E-25 | Up |
| PTGFRN | 2.197746 | 2.59E-20 | 2.87E-19 | Up |
| VTCN1 | 4.4893766 | 5.98E-12 | 3.02E-11 | Up |
| GRHL1 | 2.9669315 | 5.42E-23 | 7.44E-22 | Up |
| MYCN | 3.2653186 | 1.94E-07 | 5.77E-07 | Up |
| FST | 3.5618846 | 7.20E-14 | 4.45E-13 | Up |
| CFHR4 | 6.5465993 | 9.28E-15 | 6.22E-14 | Up |
| RAX | 5.9010509 | 2.88E-08 | 9.52E-08 | Up |
| KCTD1 | 2.7808885 | 4.03E-21 | 4.78E-20 | Up |
| SLCO1B1 | 5.6292041 | 3.52E-11 | 1.63E-10 | Up |
| PRH2 | 3.8346374 | 1.72E-05 | 3.97E-05 | Up |
| CDCA8 | 4.0990774 | 9.01E-51 | 4.86E-49 | Up |
| DSC2 | 2.375067 | 2.94E-14 | 1.88E-13 | Up |
| DSG3 | 9.3164209 | 3.23E-33 | 7.89E-32 | Up |
| DSG1 | 5.8878108 | 3.23E-11 | 1.50E-10 | Up |
| DSC3 | 5.5206033 | 4.48E-23 | 6.21E-22 | Up |
| DSC1 | 5.4042375 | 1.51E-13 | 9.05E-13 | Up |
| FHOD3 | 2.3199109 | 2.25E-08 | 7.51E-08 | Up |
| TCN1 | 5.0015099 | 9.36E-10 | 3.69E-09 | Up |
| CLDN10 | 3.2469949 | 1.83E-09 | 6.93E-09 | Up |
| ACRV1 | 2.7072895 | 1.69E-19 | 1.77E-18 | Up |
| PSAT1 | 4.8803321 | 1.50E-39 | 5.04E-38 | Up |
| MSI1 | 3.9494178 | 2.73E-16 | 2.13E-15 | Up |
| HNF1A | 2.3110548 | 0.0033256 | 0.0055246 | Up |
| RNFT2 | 2.1918366 | 2.18E-10 | 9.23E-10 | Up |
| UGT2B28 | 4.1314967 | 7.05E-06 | 1.72E-05 | Up |
| HILPDA | 2.0384345 | 6.45E-11 | 2.92E-10 | Up |
| FAM71F1 | 5.1840008 | 2.50E-10 | 1.05E-09 | Up |
| KCP | 2.4706201 | 3.26E-10 | 1.35E-09 | Up |
| MRAP2 | 3.3013374 | 8.82E-10 | 3.49E-09 | Up |
| EPHA7 | 3.3639795 | 3.22E-07 | 9.36E-07 | Up |
| CGA | 7.3562284 | 4.52E-06 | 1.13E-05 | Up |
| KRT85 | 3.7298471 | 8.06E-05 | 0.0001691 | Up |
| TROAP | 4.9969077 | 9.58E-77 | 1.77E-74 | Up |
| B4GALNT1 | 4.2800023 | 6.80E-18 | 6.21E-17 | Up |
| ESPL1 | 3.7286869 | 1.18E-55 | 8.06E-54 | Up |
| EMX1 | 5.6743724 | 1.80E-15 | 1.31E-14 | Up |
| ZNF670-ZNF695 | 2.3606859 | 1.37E-11 | 6.67E-11 | Up |
| CHRND | 2.4412804 | 0.0001607 | 0.0003232 | Up |
| PAX3 | 5.9154011 | 1.20E-10 | 5.25E-10 | Up |
| SERPINE2 | 2.8426879 | 5.14E-16 | 3.90E-15 | Up |
| WNT10A | 2.4862117 | 3.99E-07 | 1.15E-06 | Up |
| PCDH8 | 5.1904433 | 7.11E-06 | 1.73E-05 | Up |
| CNMD | 4.9298225 | 9.31E-05 | 0.0001933 | Up |
| IGF2BP3 | 4.9867555 | 6.15E-26 | 1.01E-24 | Up |
| DGKB | 2.0491683 | 9.56E-05 | 0.0001982 | Up |
| CIB2 | 2.1692526 | 3.36E-14 | 2.14E-13 | Up |
| BRIP1 | 3.4131246 | 2.89E-42 | 1.09E-40 | Up |
| ACTL6A | 2.3197249 | 6.90E-28 | 1.27E-26 | Up |
| TBR1 | 3.442793 | 2.36E-05 | 5.34E-05 | Up |
| IL36G | 9.4365264 | 1.16E-20 | 1.32E-19 | Up |
| IL36A | 7.2195355 | 3.28E-09 | 1.21E-08 | Up |
| IL36RN | 9.0378386 | 6.52E-22 | 8.23E-21 | Up |
| IL36B | 3.8288712 | 1.43E-05 | 3.33E-05 | Up |
| IL1F10 | 3.2459457 | 1.68E-05 | 3.89E-05 | Up |
| NR5A1 | 6.1145307 | 1.38E-08 | 4.76E-08 | Up |
| CTSV | 4.4267016 | 7.01E-26 | 1.15E-24 | Up |
| LMX1B | 4.1285578 | 5.50E-09 | 1.98E-08 | Up |
| DSCC1 | 2.630924 | 8.77E-33 | 2.10E-31 | Up |
| DMRT1 | 7.2425072 | 8.95E-19 | 8.80E-18 | Up |
| ARHGEF39 | 3.0582317 | 1.16E-26 | 1.97E-25 | Up |
| TFAP2A | 5.2186882 | 5.00E-49 | 2.49E-47 | Up |
| SLC22A7 | 2.6702494 | 0.0017113 | 0.0029605 | Up |
| TUBB2B | 3.9525459 | 9.92E-10 | 3.89E-09 | Up |
| HMGA1 | 2.9936071 | 5.90E-51 | 3.21E-49 | Up |
| FGFBP1 | 3.1776048 | 7.40E-10 | 2.95E-09 | Up |
| PI15 | 2.1798985 | 1.46E-10 | 6.32E-10 | Up |
| GGH | 2.5658127 | 4.21E-21 | 4.98E-20 | Up |
| TMPRSS4 | 5.7844249 | 4.17E-30 | 8.81E-29 | Up |
| MMP7 | 2.1458319 | 6.31E-06 | 1.55E-05 | Up |
| MMP20 | 6.4558351 | 6.88E-09 | 2.45E-08 | Up |
| MMP27 | 2.8551558 | 0.0001521 | 0.000307 | Up |
| TRIM29 | 4.8759707 | 2.47E-28 | 4.72E-27 | Up |
| MMP13 | 7.7213297 | 4.15E-14 | 2.61E-13 | Up |
| UNC13C | 2.8890988 | 0.0002258 | 0.0004452 | Up |
| NUSAP1 | 3.6539906 | 7.33E-59 | 5.84E-57 | Up |
| KIF23 | 3.7823392 | 1.03E-72 | 1.52E-70 | Up |
| ITGA11 | 2.0882993 | 1.20E-08 | 4.16E-08 | Up |
| KNL1 | 3.4324156 | 1.59E-58 | 1.24E-56 | Up |
| ITPKA | 3.6562193 | 1.61E-15 | 1.18E-14 | Up |
| PAK6 | 2.1977835 | 6.16E-17 | 5.15E-16 | Up |
| STRA6 | 3.9057193 | 1.63E-14 | 1.07E-13 | Up |
| BCL2L10 | 4.8494294 | 8.36E-23 | 1.13E-21 | Up |
| BRDT | 7.3876219 | 1.85E-08 | 6.25E-08 | Up |
| SLC44A5 | 3.3449146 | 6.14E-17 | 5.13E-16 | Up |
| CLCA2 | 6.0037727 | 1.52E-17 | 1.35E-16 | Up |
| CGREF1 | 3.5280502 | 6.73E-17 | 5.60E-16 | Up |
| SIX3 | 3.4543635 | 1.25E-05 | 2.95E-05 | Up |
| TRIM54 | 2.1154473 | 0.0004037 | 0.0007654 | Up |
| BTBD16 | 3.6407874 | 8.22E-07 | 2.27E-06 | Up |
| KIF11 | 3.6260548 | 3.43E-65 | 3.48E-63 | Up |
| CEP55 | 4.3147794 | 5.14E-59 | 4.13E-57 | Up |
| GPR87 | 6.5841406 | 2.06E-34 | 5.38E-33 | Up |
| DNA2 | 2.69963 | 9.93E-35 | 2.62E-33 | Up |
| FGF5 | 5.8060788 | 6.12E-09 | 2.19E-08 | Up |
| IL21 | 2.4235461 | 3.63E-05 | 8.01E-05 | Up |
| CENPE | 3.2297392 | 2.87E-43 | 1.12E-41 | Up |
| C4orf17 | 5.2139757 | 4.43E-08 | 1.43E-07 | Up |
| FBN2 | 5.1453112 | 7.41E-12 | 3.69E-11 | Up |
| TTLL8 | 2.8855554 | 3.58E-06 | 9.06E-06 | Up |
| PIK3C2G | 2.0084577 | 0.0004516 | 0.0008498 | Up |
| PLCZ1 | 3.4857627 | 2.60E-05 | 5.86E-05 | Up |
| TMEM117 | 2.602483 | 1.93E-25 | 3.10E-24 | Up |
| SLC38A4 | 2.6066704 | 1.33E-06 | 3.57E-06 | Up |
| COL2A1 | 4.9330045 | 5.73E-09 | 2.06E-08 | Up |
| INHBE | 2.9085374 | 1.33E-10 | 5.77E-10 | Up |
| LGR5 | 2.409414 | 0.0001004 | 0.0002075 | Up |
| ASCL1 | 6.8550734 | 8.08E-07 | 2.23E-06 | Up |
| PDX1 | 5.9088474 | 2.82E-09 | 1.05E-08 | Up |
| SLC39A5 | 4.0855348 | 1.33E-06 | 3.57E-06 | Up |
| RDH16 | 2.7155487 | 1.42E-12 | 7.68E-12 | Up |
| ZIC5 | 8.3682992 | 3.17E-31 | 7.15E-30 | Up |
| GRTP1 | 2.710237 | 1.01E-11 | 4.98E-11 | Up |
| CDH24 | 2.0655334 | 8.00E-20 | 8.60E-19 | Up |
| MDGA2 | 3.1690577 | 0.0010057 | 0.001796 | Up |
| FRMD6 | 2.2086088 | 1.96E-16 | 1.55E-15 | Up |
| SYT16 | 2.2964617 | 1.94E-06 | 5.09E-06 | Up |
| RDH12 | 2.5799249 | 1.93E-07 | 5.75E-07 | Up |
| SORD | 2.2473764 | 6.93E-23 | 9.44E-22 | Up |
| DUOXA2 | 2.3146193 | 8.85E-06 | 2.13E-05 | Up |
| GCNT3 | 2.7364186 | 1.72E-07 | 5.16E-07 | Up |
| PIF1 | 3.266201 | 8.34E-34 | 2.11E-32 | Up |
| RHCG | 8.9157878 | 2.01E-20 | 2.23E-19 | Up |
| FANCI | 3.0383047 | 2.38E-48 | 1.15E-46 | Up |
| TICRR | 4.4582862 | 1.04E-49 | 5.37E-48 | Up |
| ST8SIA2 | 4.2507854 | 1.01E-10 | 4.46E-10 | Up |
| 12-Sep | 3.3213473 | 2.41E-07 | 7.10E-07 | Up |
| CLEC18B | 2.2793225 | 1.58E-08 | 5.39E-08 | Up |
| ADAMTS18 | 2.2794729 | 8.55E-05 | 0.0001787 | Up |
| ADAD2 | 3.156878 | 2.14E-10 | 9.05E-10 | Up |
| OSGIN1 | 2.0466715 | 1.91E-05 | 4.37E-05 | Up |
| RPL3L | 3.9366847 | 4.50E-18 | 4.17E-17 | Up |
| DPEP3 | 3.7041023 | 0.0001521 | 0.000307 | Up |
| RHBDL3 | 2.2751665 | 5.26E-06 | 1.31E-05 | Up |
| GREB1L | 2.8720759 | 2.40E-07 | 7.06E-07 | Up |
| SLC13A5 | 5.3946083 | 1.65E-12 | 8.85E-12 | Up |
| CARD14 | 3.4759388 | 8.69E-27 | 1.50E-25 | Up |
| ZNF750 | 2.9062202 | 2.05E-13 | 1.21E-12 | Up |
| CBLN2 | 3.7991243 | 6.97E-05 | 0.0001476 | Up |
| PMAIP1 | 2.6590916 | 5.88E-23 | 8.05E-22 | Up |
| P3H4 | 2.1691463 | 3.53E-26 | 5.88E-25 | Up |
| STAC2 | 3.5722988 | 1.62E-05 | 3.76E-05 | Up |
| PLPP2 | 2.5827241 | 1.26E-22 | 1.68E-21 | Up |
| IL19 | 5.0947118 | 1.83E-08 | 6.19E-08 | Up |
| CBLC | 4.1840269 | 1.50E-37 | 4.56E-36 | Up |
| SLC6A3 | 2.8376629 | 0.0004277 | 0.000808 | Up |
| IGLON5 | 3.7970612 | 5.76E-13 | 3.23E-12 | Up |
| SLC2A5 | 2.3729867 | 4.85E-16 | 3.70E-15 | Up |
| PADI3 | 10.031832 | 5.17E-20 | 5.61E-19 | Up |
| PADI1 | 7.6131906 | 3.32E-15 | 2.35E-14 | Up |
| ARHGEF19 | 2.4521279 | 4.16E-27 | 7.32E-26 | Up |
| DMRTA2 | 6.5377703 | 2.76E-16 | 2.16E-15 | Up |
| PLK4 | 2.550366 | 2.20E-38 | 7.00E-37 | Up |
| KIF2C | 4.4316884 | 4.42E-74 | 7.07E-72 | Up |
| PROK1 | 2.637971 | 0.0024868 | 0.004207 | Up |
| CELSR2 | 2.8541501 | 1.06E-21 | 1.33E-20 | Up |
| UCK2 | 2.1967574 | 4.57E-29 | 9.06E-28 | Up |
| MAEL | 3.8965593 | 8.96E-06 | 2.15E-05 | Up |
| ADCY10 | 4.7281022 | 7.37E-12 | 3.68E-11 | Up |
| NECTIN4 | 3.2413768 | 1.60E-23 | 2.29E-22 | Up |
| NUF2 | 4.7325794 | 3.94E-57 | 2.82E-55 | Up |
| FCRL5 | 2.2343131 | 5.00E-07 | 1.42E-06 | Up |
| CRABP2 | 4.130186 | 2.30E-16 | 1.81E-15 | Up |
| FAM163A | 2.7647413 | 5.22E-05 | 0.0001125 | Up |
| HORMAD1 | 4.3725323 | 8.73E-10 | 3.45E-09 | Up |
| SYT14 | 7.386177 | 4.91E-23 | 6.77E-22 | Up |
| KCNH1 | 3.9337448 | 2.96E-10 | 1.23E-09 | Up |
| DTL | 2.7645918 | 6.87E-34 | 1.74E-32 | Up |
| SUSD4 | 3.0319241 | 3.16E-15 | 2.24E-14 | Up |
| HHIPL2 | 3.5539229 | 1.79E-07 | 5.36E-07 | Up |
| FLG2 | 3.3027025 | 1.56E-05 | 3.62E-05 | Up |
| CRNN | 8.68696 | 1.39E-07 | 4.23E-07 | Up |
| S100A8 | 2.7079266 | 1.99E-05 | 4.55E-05 | Up |
| S100A7 | 9.5674241 | 7.23E-15 | 4.91E-14 | Up |
| EFNA3 | 4.0420237 | 1.50E-35 | 4.14E-34 | Up |
| PKLR | 2.4324846 | 2.50E-05 | 5.64E-05 | Up |
| ETNK2 | 2.4070711 | 5.62E-24 | 8.23E-23 | Up |
| PPFIA4 | 2.8329317 | 1.02E-15 | 7.60E-15 | Up |
| SYT2 | 2.4913199 | 5.78E-08 | 1.84E-07 | Up |
| ATP6V1C2 | 2.5489244 | 5.40E-12 | 2.74E-11 | Up |
| ABCG8 | 4.1027882 | 3.94E-07 | 1.13E-06 | Up |
| CHAC2 | 2.0915726 | 1.11E-14 | 7.41E-14 | Up |
| DQX1 | 5.5806374 | 1.18E-34 | 3.09E-33 | Up |
| C1QL2 | 2.3912579 | 0.0047644 | 0.0077396 | Up |
| NT5DC4 | 2.0932892 | 1.87E-09 | 7.09E-09 | Up |
| THSD7B | 2.0972474 | 0.0001672 | 0.0003354 | Up |
| CDCA7 | 3.6743029 | 1.32E-36 | 3.85E-35 | Up |
| DLX1 | 6.5557325 | 9.65E-17 | 7.92E-16 | Up |
| CCDC150 | 2.9910177 | 1.36E-21 | 1.68E-20 | Up |
| PTH2R | 3.8661509 | 2.12E-07 | 6.28E-07 | Up |
| ABCA12 | 6.2157259 | 2.63E-33 | 6.48E-32 | Up |
| ACKR3 | 2.1682178 | 4.51E-09 | 1.64E-08 | Up |
| TRPM8 | 5.1530962 | 2.51E-11 | 1.19E-10 | Up |
| HES6 | 3.0292967 | 8.62E-09 | 3.04E-08 | Up |
| FANCD2 | 2.1453217 | 7.04E-34 | 1.79E-32 | Up |
| LRTM1 | 3.621973 | 2.16E-05 | 4.92E-05 | Up |
| TAGLN3 | 3.1741227 | 8.28E-05 | 0.0001734 | Up |
| IGSF11 | 4.9384213 | 3.57E-22 | 4.59E-21 | Up |
| ALDH1L1 | 2.3824694 | 1.66E-05 | 3.84E-05 | Up |
| UCN2 | 5.7996575 | 1.83E-24 | 2.76E-23 | Up |
| STXBP5L | 6.9684591 | 1.88E-14 | 1.22E-13 | Up |
| TM4SF19 | 2.3218601 | 2.03E-07 | 6.03E-07 | Up |
| AHSG | 7.1331777 | 8.56E-08 | 2.67E-07 | Up |
| ECE2 | 3.5213533 | 8.77E-39 | 2.86E-37 | Up |
| VWA5B2 | 2.2967066 | 2.93E-06 | 7.48E-06 | Up |
| SLC10A6 | 2.161512 | 1.87E-05 | 4.29E-05 | Up |
| CCNA2 | 4.0648365 | 1.45E-61 | 1.30E-59 | Up |
| GLRA3 | 3.3738043 | 4.33E-09 | 1.58E-08 | Up |
| CDH18 | 5.5710309 | 7.81E-07 | 2.16E-06 | Up |
| SRD5A1 | 2.20833 | 4.60E-17 | 3.91E-16 | Up |
| SKP2 | 2.8287316 | 7.69E-31 | 1.70E-29 | Up |
| UGT3A1 | 5.668297 | 5.23E-05 | 0.0001126 | Up |
| SHISAL2B | 2.8455712 | 2.91E-06 | 7.44E-06 | Up |
| BHMT | 2.7501133 | 1.77E-05 | 4.08E-05 | Up |
| LIX1 | 3.4024751 | 0.0001722 | 0.0003448 | Up |
| MEGF10 | 4.8234035 | 2.60E-12 | 1.37E-11 | Up |
| CXCL14 | 4.2288572 | 1.10E-13 | 6.67E-13 | Up |
| LECT2 | 4.6788452 | 7.32E-06 | 1.78E-05 | Up |
| SLC25A48 | 6.091966 | 1.47E-16 | 1.18E-15 | Up |
| SPINK7 | 3.1585552 | 0.0001954 | 0.0003887 | Up |
| CPLX2 | 4.7453346 | 4.20E-05 | 9.17E-05 | Up |
| TENM2 | 4.933424 | 6.60E-13 | 3.68E-12 | Up |
| TRIM7 | 3.0278202 | 1.75E-11 | 8.41E-11 | Up |
| RASGEF1C | 2.28023 | 5.73E-05 | 0.0001227 | Up |
| MLIP | 3.5988643 | 4.71E-10 | 1.92E-09 | Up |
| LGSN | 2.0637607 | 0.0044753 | 0.0073042 | Up |
| SCUBE3 | 2.317973 | 7.55E-08 | 2.37E-07 | Up |
| CRIP3 | 2.3575618 | 0.0001048 | 0.0002158 | Up |
| TTBK1 | 3.1577339 | 1.38E-10 | 5.97E-10 | Up |
| TPBG | 2.2147474 | 5.22E-22 | 6.63E-21 | Up |
| GABRR1 | 6.661809 | 2.60E-19 | 2.68E-18 | Up |
| CLVS2 | 4.4851599 | 7.01E-05 | 0.0001483 | Up |
| MTFR2 | 2.4545359 | 1.02E-26 | 1.75E-25 | Up |
| PNLDC1 | 3.9958446 | 6.29E-07 | 1.76E-06 | Up |
| VWDE | 2.501662 | 4.07E-08 | 1.32E-07 | Up |
| CDCA5 | 4.1981327 | 4.02E-79 | 8.16E-77 | Up |
| IGFBP3 | 2.8027574 | 1.66E-12 | 8.88E-12 | Up |
| IGFBP1 | 2.7204104 | 0.0053838 | 0.0086741 | Up |
| PSPH | 2.2594858 | 7.93E-17 | 6.55E-16 | Up |
| ZAN | 5.4174521 | 1.20E-11 | 5.88E-11 | Up |
| STRA8 | 5.5722162 | 2.21E-07 | 6.55E-07 | Up |
| CNPY1 | 4.0893699 | 2.29E-05 | 5.18E-05 | Up |
| NCAPG2 | 2.2723152 | 6.77E-32 | 1.56E-30 | Up |
| HTR2C | 7.9350528 | 1.09E-22 | 1.46E-21 | Up |
| IGSF1 | 4.1381824 | 7.03E-14 | 4.35E-13 | Up |
| ARHGAP36 | 5.025058 | 2.14E-07 | 6.34E-07 | Up |
| MAGEA4 | 9.4379986 | 6.67E-14 | 4.14E-13 | Up |
| CHRNA6 | 4.2000891 | 4.56E-07 | 1.30E-06 | Up |
| STAR | 5.0160195 | 2.20E-13 | 1.29E-12 | Up |
| ST18 | 3.4002917 | 5.59E-07 | 1.58E-06 | Up |
| RGS20 | 3.8765477 | 2.85E-16 | 2.22E-15 | Up |
| GINS4 | 3.6033479 | 5.14E-32 | 1.19E-30 | Up |
| PMP2 | 5.8188762 | 3.33E-06 | 8.45E-06 | Up |
| FAM83A | 6.5461707 | 1.34E-20 | 1.51E-19 | Up |
| GSDMC | 4.358014 | 6.72E-21 | 7.81E-20 | Up |
| CDKN2A | 4.4248719 | 4.36E-13 | 2.47E-12 | Up |
| NTRK2 | 3.1633188 | 1.66E-07 | 4.98E-07 | Up |
| PTGES | 2.2718199 | 4.58E-09 | 1.67E-08 | Up |
| CACNA1B | 6.6154885 | 5.59E-20 | 6.06E-19 | Up |
| A1CF | 4.3347274 | 2.25E-05 | 5.09E-05 | Up |
| CDHR1 | 3.78893 | 3.12E-12 | 1.63E-11 | Up |
| HTR7 | 3.1812675 | 5.87E-10 | 2.36E-09 | Up |
| VAX1 | 8.0836552 | 1.08E-15 | 8.00E-15 | Up |
| MKI67 | 3.7923267 | 3.89E-42 | 1.46E-40 | Up |
| INA | 6.9302193 | 1.60E-14 | 1.05E-13 | Up |
| ADAM12 | 3.3363914 | 4.41E-21 | 5.22E-20 | Up |
| SLC5A12 | 4.8921606 | 7.79E-21 | 8.98E-20 | Up |
| TENM4 | 2.8865956 | 4.06E-19 | 4.12E-18 | Up |
| DRD2 | 2.8364955 | 4.79E-06 | 1.19E-05 | Up |
| HTR3B | 3.5394529 | 1.86E-05 | 4.26E-05 | Up |
| PLCH2 | 3.6892741 | 2.20E-16 | 1.74E-15 | Up |
| CHEK1 | 2.9370461 | 1.96E-52 | 1.15E-50 | Up |
| CDH22 | 3.4914249 | 0.0001105 | 0.000227 | Up |
| TBX6 | 2.0353993 | 5.30E-09 | 1.91E-08 | Up |
| FAM57B | 3.3977574 | 7.58E-10 | 3.02E-09 | Up |
| HMGA2 | 6.9940454 | 1.90E-22 | 2.49E-21 | Up |
| MMP3 | 6.2543733 | 2.19E-16 | 1.73E-15 | Up |
| CNTN5 | 2.3715459 | 8.02E-06 | 1.94E-05 | Up |
| CDH8 | 2.9146339 | 3.60E-11 | 1.67E-10 | Up |
| LYPD1 | 3.6678013 | 1.37E-12 | 7.39E-12 | Up |
| LYPD6B | 3.1624849 | 6.96E-14 | 4.31E-13 | Up |
| C11orf53 | 4.1715383 | 6.09E-06 | 1.50E-05 | Up |
| SLC7A11 | 4.4407623 | 2.43E-15 | 1.74E-14 | Up |
| GPR158 | 2.0526413 | 8.32E-06 | 2.01E-05 | Up |
| BTBD11 | 3.8936226 | 3.90E-23 | 5.45E-22 | Up |
| ALLC | 2.9471527 | 1.95E-05 | 4.46E-05 | Up |
| MSGN1 | 6.7357542 | 1.13E-09 | 4.39E-09 | Up |
| AKR1C2 | 5.542699 | 2.27E-15 | 1.63E-14 | Up |
| DPYSL4 | 2.2857351 | 3.24E-07 | 9.41E-07 | Up |
| CENPU | 3.2024071 | 1.45E-37 | 4.44E-36 | Up |
| MCHR2 | 2.8805874 | 0.0002261 | 0.0004456 | Up |
| TUBA3E | 2.90474 | 0.0002202 | 0.0004347 | Up |
| POU4F1 | 5.8505778 | 4.76E-14 | 2.98E-13 | Up |
| SPC25 | 3.3066401 | 5.92E-43 | 2.28E-41 | Up |
| PDK1 | 2.2027608 | 2.68E-26 | 4.49E-25 | Up |
| SPOCK1 | 3.22103 | 4.38E-15 | 3.04E-14 | Up |
| BOLL | 3.243033 | 6.90E-09 | 2.46E-08 | Up |
| DSPP | 3.0938081 | 8.02E-08 | 2.51E-07 | Up |
| DMP1 | 2.8441059 | 9.74E-10 | 3.83E-09 | Up |
| ANKRD22 | 2.4753749 | 3.52E-16 | 2.72E-15 | Up |
| GRM1 | 2.1094331 | 0.0002798 | 0.0005436 | Up |
| CNTNAP4 | 4.6282793 | 6.16E-09 | 2.21E-08 | Up |
| PLOD2 | 2.0981842 | 5.10E-15 | 3.51E-14 | Up |
| NRSN1 | 4.758916 | 6.70E-05 | 0.0001421 | Up |
| ZIC1 | 6.4933414 | 1.33E-08 | 4.59E-08 | Up |
| CENPH | 2.5627081 | 2.45E-36 | 7.05E-35 | Up |
| CLGN | 2.1460794 | 1.85E-06 | 4.88E-06 | Up |
| ADGRF4 | 4.8134155 | 7.44E-20 | 8.01E-19 | Up |
| FRMD1 | 2.5417907 | 0.0044936 | 0.0073307 | Up |
| PLEKHG4B | 2.3829185 | 1.58E-08 | 5.38E-08 | Up |
| TMPRSS11D | 9.0357449 | 1.06E-19 | 1.13E-18 | Up |
| HS3ST3A1 | 2.1952132 | 2.19E-10 | 9.26E-10 | Up |
| CABYR | 4.7342864 | 1.02E-19 | 1.09E-18 | Up |
| C16orf74 | 3.2737287 | 6.30E-19 | 6.28E-18 | Up |
| JPH3 | 3.7403255 | 9.20E-08 | 2.86E-07 | Up |
| CDH12 | 4.0946017 | 1.49E-05 | 3.47E-05 | Up |
| CERS3 | 8.509313 | 3.55E-31 | 7.97E-30 | Up |
| UCHL1 | 4.8687894 | 2.30E-17 | 2.00E-16 | Up |
| GPR26 | 2.1659284 | 0.0012991 | 0.0022836 | Up |
| C10orf90 | 4.4153044 | 5.68E-09 | 2.04E-08 | Up |
| SRSF12 | 2.5335475 | 1.97E-12 | 1.05E-11 | Up |
| CHODL | 4.0702604 | 9.82E-16 | 7.30E-15 | Up |
| TMPRSS15 | 4.2404411 | 2.78E-07 | 8.11E-07 | Up |
| SKA1 | 4.4416709 | 2.02E-57 | 1.48E-55 | Up |
| RAB6B | 2.4902878 | 8.66E-08 | 2.70E-07 | Up |
| EME1 | 3.6244106 | 2.46E-43 | 9.63E-42 | Up |
| EPHB1 | 2.6196671 | 2.42E-07 | 7.12E-07 | Up |
| CA10 | 2.4421696 | 0.0024475 | 0.0041458 | Up |
| CNTNAP5 | 5.5963702 | 1.84E-10 | 7.84E-10 | Up |
| PROM2 | 3.3948273 | 1.88E-16 | 1.50E-15 | Up |
| GPR78 | 2.0018695 | 1.04E-05 | 2.47E-05 | Up |
| PPM1J | 2.1827951 | 9.52E-17 | 7.83E-16 | Up |
| SLC16A1 | 2.3093217 | 1.36E-16 | 1.10E-15 | Up |
| MAGEC1 | 8.8950971 | 8.64E-08 | 2.69E-07 | Up |
| SLC24A2 | 5.0814555 | 2.80E-26 | 4.70E-25 | Up |
| RAET1L | 9.1807504 | 1.84E-30 | 3.96E-29 | Up |
| GRIP1 | 2.3858031 | 6.93E-12 | 3.47E-11 | Up |
| KIF5A | 3.3412098 | 1.43E-10 | 6.18E-10 | Up |
| MAGEA8 | 3.412459 | 2.29E-06 | 5.94E-06 | Up |
| ALX3 | 6.4128908 | 4.92E-09 | 1.78E-08 | Up |
| ART3 | 4.0080983 | 1.76E-05 | 4.05E-05 | Up |
| CXCL13 | 4.3844848 | 1.28E-09 | 4.95E-09 | Up |
| NAA11 | 6.4507353 | 9.62E-06 | 2.30E-05 | Up |
| CLDN17 | 7.01158 | 1.06E-08 | 3.68E-08 | Up |
| CLDN8 | 2.0585863 | 0.0032337 | 0.0053798 | Up |
| SORCS3 | 5.0948277 | 4.86E-07 | 1.38E-06 | Up |
| FUT6 | 3.1507712 | 1.62E-06 | 4.29E-06 | Up |
| FBXO43 | 3.3271416 | 9.62E-23 | 1.29E-21 | Up |
| LRFN2 | 2.962741 | 1.50E-05 | 3.50E-05 | Up |
| GLYATL2 | 5.7816709 | 3.20E-12 | 1.67E-11 | Up |
| ATAD2 | 2.3867537 | 5.42E-37 | 1.60E-35 | Up |
| FBXO32 | 2.2300268 | 1.80E-19 | 1.88E-18 | Up |
| ZIC3 | 5.9846918 | 3.80E-06 | 9.59E-06 | Up |
| LHFPL4 | 2.4391005 | 0.0033903 | 0.0056242 | Up |
| BUB1B | 4.4730654 | 1.12E-74 | 1.85E-72 | Up |
| TMEM171 | 3.9526939 | 1.05E-24 | 1.60E-23 | Up |
| LRP8 | 2.006932 | 6.54E-13 | 3.64E-12 | Up |
| CCNB2 | 4.2387071 | 3.49E-74 | 5.69E-72 | Up |
| DGKI | 2.5663317 | 1.25E-08 | 4.32E-08 | Up |
| GAREM2 | 2.634635 | 9.82E-25 | 1.50E-23 | Up |
| DPYSL5 | 6.9970019 | 9.24E-10 | 3.64E-09 | Up |
| GRHL3 | 6.5538846 | 5.01E-34 | 1.28E-32 | Up |
| GALNT14 | 4.3854153 | 1.03E-22 | 1.39E-21 | Up |
| XDH | 3.6058519 | 2.11E-15 | 1.52E-14 | Up |
| RHBDL2 | 2.2149595 | 2.02E-11 | 9.64E-11 | Up |
| HIST1H2BD | 2.2351973 | 3.64E-15 | 2.56E-14 | Up |
| CDC25C | 4.4533499 | 7.39E-53 | 4.47E-51 | Up |
| HIST1H4H | 2.1368097 | 1.35E-08 | 4.65E-08 | Up |
| CPA2 | 2.761751 | 5.81E-06 | 1.43E-05 | Up |
| POM121L2 | 2.2698588 | 0.0001386 | 0.0002813 | Up |
| DYNC1I1 | 2.9450257 | 3.15E-14 | 2.01E-13 | Up |
| PAGE5 | 4.0238889 | 0.0002649 | 0.0005166 | Up |
| HTR6 | 2.5523117 | 2.18E-06 | 5.67E-06 | Up |
| PLA2G2F | 7.6669955 | 6.21E-11 | 2.81E-10 | Up |
| SLC5A11 | 2.9203628 | 1.95E-06 | 5.12E-06 | Up |
| APOA2 | 7.9030597 | 1.79E-05 | 4.11E-05 | Up |
| SV2A | 2.1666748 | 1.65E-11 | 7.98E-11 | Up |
| LAD1 | 2.5619833 | 3.26E-23 | 4.59E-22 | Up |
| HOXB13 | 9.2569856 | 7.01E-17 | 5.82E-16 | Up |
| IGF2BP1 | 7.4785284 | 4.02E-17 | 3.44E-16 | Up |
| CBR1 | 2.0858993 | 6.68E-09 | 2.38E-08 | Up |
| CBR3 | 2.8069164 | 1.17E-10 | 5.15E-10 | Up |
| CHAF1B | 2.4511328 | 2.02E-34 | 5.28E-33 | Up |
| SIM2 | 2.8652542 | 7.39E-15 | 5.01E-14 | Up |
| PLA2G4D | 2.009053 | 2.61E-05 | 5.87E-05 | Up |
| CES5A | 3.7951757 | 1.27E-05 | 2.99E-05 | Up |
| CELF3 | 4.7922727 | 3.11E-05 | 6.91E-05 | Up |
| TCHH | 3.3253929 | 5.91E-07 | 1.66E-06 | Up |
| TGM7 | 2.0456121 | 0.0021457 | 0.0036659 | Up |
| SPRR2G | 8.3164234 | 2.05E-11 | 9.78E-11 | Up |
| PGLYRP3 | 9.1045176 | 3.47E-26 | 5.79E-25 | Up |
| ISL2 | 3.0022647 | 1.42E-16 | 1.15E-15 | Up |
| ANKLE1 | 2.1252031 | 2.93E-09 | 1.09E-08 | Up |
| CILP2 | 2.7500292 | 1.92E-12 | 1.02E-11 | Up |
| TFF2 | 6.107766 | 2.10E-06 | 5.48E-06 | Up |
| TFF1 | 4.154066 | 1.31E-06 | 3.52E-06 | Up |
| HSF2BP | 2.7019807 | 1.24E-21 | 1.54E-20 | Up |
| AIRE | 3.1745679 | 4.36E-06 | 1.09E-05 | Up |
| FTCD | 3.5889963 | 1.05E-08 | 3.67E-08 | Up |
| COX6B2 | 5.5144231 | 1.14E-16 | 9.34E-16 | Up |
| NLRP4 | 2.6653538 | 0.0007959 | 0.0014446 | Up |
| CHRNB2 | 3.7967543 | 4.20E-10 | 1.72E-09 | Up |
| LY6K | 4.8435955 | 5.24E-19 | 5.25E-18 | Up |
| TONSL | 2.8886135 | 3.80E-35 | 1.02E-33 | Up |
| RECQL4 | 3.9415463 | 1.76E-46 | 7.87E-45 | Up |
| COL26A1 | 2.583254 | 0.0001021 | 0.0002107 | Up |
| FOXH1 | 5.3214742 | 1.82E-12 | 9.72E-12 | Up |
| CELF5 | 2.7232596 | 1.87E-07 | 5.58E-07 | Up |
| YDJC | 2.1095383 | 2.63E-22 | 3.43E-21 | Up |
| FBXO27 | 3.3124871 | 1.47E-16 | 1.18E-15 | Up |
| BDH1 | 2.5421372 | 1.50E-20 | 1.69E-19 | Up |
| NPHS1 | 2.1770894 | 0.0027562 | 0.0046299 | Up |
| RACGAP1 | 2.4741009 | 1.02E-43 | 4.03E-42 | Up |
| KRT84 | 6.7990453 | 1.21E-09 | 4.71E-09 | Up |
| KRT82 | 3.3360225 | 1.48E-07 | 4.49E-07 | Up |
| SPC24 | 3.8135731 | 2.39E-49 | 1.21E-47 | Up |
| FGF11 | 4.4560624 | 1.08E-24 | 1.64E-23 | Up |
| SSTR5 | 4.4087437 | 5.37E-07 | 1.52E-06 | Up |
| MEIOB | 3.8578172 | 1.28E-05 | 3.02E-05 | Up |
| TEDC2 | 4.1358911 | 1.33E-56 | 9.35E-55 | Up |
| CCNF | 2.7248246 | 1.29E-47 | 6.03E-46 | Up |
| FGF19 | 8.3461048 | 1.69E-08 | 5.75E-08 | Up |
| ELAVL4 | 2.5254598 | 3.42E-05 | 7.58E-05 | Up |
| AK4 | 2.7336822 | 4.47E-27 | 7.86E-26 | Up |
| AKR7A3 | 3.1505638 | 4.02E-09 | 1.47E-08 | Up |
| LRRC38 | 4.2648152 | 3.86E-06 | 9.73E-06 | Up |
| LHX8 | 7.7038668 | 2.57E-08 | 8.53E-08 | Up |
| SLAMF9 | 3.3267262 | 1.37E-10 | 5.96E-10 | Up |
| VANGL2 | 2.4850375 | 2.84E-18 | 2.67E-17 | Up |
| C1orf74 | 2.5004554 | 2.63E-22 | 3.43E-21 | Up |
| LMX1A | 3.0821029 | 0.0017037 | 0.002948 | Up |
| AXDND1 | 2.5640655 | 1.35E-05 | 3.17E-05 | Up |
| TDRD5 | 4.6678485 | 9.70E-15 | 6.49E-14 | Up |
| KIF26B | 2.4050486 | 1.29E-18 | 1.26E-17 | Up |
| TRIM17 | 2.2625422 | 4.04E-08 | 1.31E-07 | Up |
| FAM84A | 2.6061327 | 3.32E-15 | 2.35E-14 | Up |
| FBXO41 | 2.0187123 | 8.43E-17 | 6.96E-16 | Up |
| VSNL1 | 3.2698105 | 1.16E-15 | 8.55E-15 | Up |
| EN1 | 7.5574266 | 6.87E-16 | 5.17E-15 | Up |
| LCE3D | 9.6637511 | 2.77E-12 | 1.46E-11 | Up |
| SMCP | 6.8509399 | 1.88E-08 | 6.36E-08 | Up |
| IVL | 7.3814575 | 9.97E-18 | 8.97E-17 | Up |
| SPRR3 | 10.365851 | 2.37E-19 | 2.46E-18 | Up |
| SPRR2D | 7.9600777 | 1.70E-16 | 1.36E-15 | Up |
| PGLYRP4 | 5.2720156 | 9.00E-16 | 6.70E-15 | Up |
| S100A9 | 2.7133196 | 2.53E-06 | 6.53E-06 | Up |
| NPPC | 5.8604694 | 4.32E-12 | 2.22E-11 | Up |
| DAPL1 | 6.967619 | 4.37E-20 | 4.77E-19 | Up |
| CLDN1 | 2.9609247 | 7.50E-11 | 3.37E-10 | Up |
| LENEP | 2.0674601 | 1.08E-06 | 2.93E-06 | Up |
| INAVA | 2.3153915 | 1.13E-19 | 1.20E-18 | Up |
| FAM19A4 | 2.2149951 | 0.0001251 | 0.0002554 | Up |
| C3orf30 | 2.6147036 | 0.0003277 | 0.0006303 | Up |
| PDCL2 | 5.5512846 | 9.98E-07 | 2.72E-06 | Up |
| TSACC | 2.7196048 | 2.69E-21 | 3.24E-20 | Up |
| TMEM79 | 3.0275679 | 2.70E-25 | 4.29E-24 | Up |
| CRYBA2 | 4.8562568 | 3.78E-08 | 1.23E-07 | Up |
| CIP2A | 3.0465216 | 1.04E-43 | 4.11E-42 | Up |
| RETNLB | 5.5701488 | 1.01E-17 | 9.10E-17 | Up |
| FCRL4 | 2.3623425 | 1.72E-05 | 3.96E-05 | Up |
| DPPA2 | 7.1092139 | 5.75E-08 | 1.83E-07 | Up |
| SGO2 | 2.1490659 | 5.11E-26 | 8.47E-25 | Up |
| AIM2 | 2.8445541 | 1.20E-10 | 5.25E-10 | Up |
| NKX6-1 | 3.2953871 | 1.67E-10 | 7.17E-10 | Up |
| SYNPR | 6.0942273 | 2.53E-06 | 6.53E-06 | Up |
| ALB | 3.1891372 | 0.0002918 | 0.0005653 | Up |
| C3orf67 | 2.7913891 | 4.95E-18 | 4.57E-17 | Up |
| FANCD2OS | 2.205215 | 2.13E-08 | 7.15E-08 | Up |
| PLSCR2 | 2.0471717 | 4.47E-06 | 1.12E-05 | Up |
| KIF15 | 3.2797428 | 3.33E-42 | 1.26E-40 | Up |
| RTP3 | 5.3266846 | 2.50E-06 | 6.44E-06 | Up |
| POLR2H | 2.0108809 | 5.20E-28 | 9.69E-27 | Up |
| CAMK2N2 | 3.9489151 | 1.55E-16 | 1.25E-15 | Up |
| RFC4 | 3.3275098 | 5.89E-43 | 2.28E-41 | Up |
| RPL39L | 3.6747968 | 5.96E-26 | 9.84E-25 | Up |
| PIGX | 2.0063522 | 4.29E-17 | 3.66E-16 | Up |
| MELTF | 3.7092624 | 5.42E-21 | 6.37E-20 | Up |
| S100P | 2.7435201 | 6.48E-06 | 1.59E-05 | Up |
| CLDN19 | 4.1120545 | 4.24E-05 | 9.25E-05 | Up |
| CDC25A | 3.5712886 | 5.73E-49 | 2.84E-47 | Up |
| HSPA4L | 2.0054828 | 1.95E-13 | 1.16E-12 | Up |
| CAMKV | 3.4288867 | 1.89E-07 | 5.64E-07 | Up |
| PITX2 | 8.1351356 | 5.25E-18 | 4.84E-17 | Up |
| MAD2L1 | 3.5212375 | 4.61E-47 | 2.10E-45 | Up |
| F2RL2 | 2.7959422 | 2.44E-06 | 6.30E-06 | Up |
| PRDM9 | 6.1333081 | 4.28E-08 | 1.38E-07 | Up |
| SPINK1 | 4.0523127 | 1.43E-06 | 3.80E-06 | Up |
| CAGE1 | 3.5292543 | 2.61E-15 | 1.86E-14 | Up |
| TERT | 6.6428622 | 7.96E-23 | 1.08E-21 | Up |
| GJB7 | 2.8060559 | 8.40E-10 | 3.33E-09 | Up |
| GRIK2 | 2.2445913 | 4.01E-05 | 8.77E-05 | Up |
| FABP7 | 8.4899159 | 1.70E-09 | 6.47E-09 | Up |
| TLX3 | 8.8337825 | 5.54E-14 | 3.47E-13 | Up |
| CALHM4 | 2.0434 | 0.0060029 | 0.0096107 | Up |
| TBXT | 4.4542594 | 1.45E-05 | 3.38E-05 | Up |
| SFXN1 | 2.0689825 | 7.14E-30 | 1.49E-28 | Up |
| IL22RA2 | 3.9175685 | 2.88E-14 | 1.85E-13 | Up |
| IL31RA | 2.9093638 | 1.25E-09 | 4.84E-09 | Up |
| TBX20 | 3.2665134 | 0.0001033 | 0.0002131 | Up |
| PTTG1 | 3.2754716 | 7.67E-51 | 4.15E-49 | Up |
| FBXL21 | 5.5136155 | 9.77E-07 | 2.67E-06 | Up |
| SLC29A4 | 2.7297936 | 1.09E-12 | 5.94E-12 | Up |
| STEAP1 | 2.1238592 | 1.02E-12 | 5.57E-12 | Up |
| SP8 | 6.0823367 | 2.37E-12 | 1.25E-11 | Up |
| SLC35G3 | 3.4013051 | 3.48E-05 | 7.70E-05 | Up |
| SUN3 | 3.2049048 | 1.14E-06 | 3.07E-06 | Up |
| HNF4G | 2.0540296 | 6.07E-07 | 1.70E-06 | Up |
| EN2 | 6.0752873 | 1.36E-15 | 9.97E-15 | Up |
| KCNV1 | 3.3124238 | 5.01E-05 | 0.0001082 | Up |
| DEFB1 | 2.1789807 | 0.0001374 | 0.0002789 | Up |
| TMEM184A | 2.4757316 | 1.76E-12 | 9.40E-12 | Up |
| GBX1 | 3.5622081 | 0.0011263 | 0.0019987 | Up |
| CTHRC1 | 3.510757 | 4.06E-28 | 7.61E-27 | Up |
| DIRAS2 | 4.6576628 | 1.04E-08 | 3.65E-08 | Up |
| ZMAT4 | 4.8638492 | 7.04E-07 | 1.96E-06 | Up |
| NKX6-3 | 5.0402667 | 7.90E-07 | 2.19E-06 | Up |
| RNF183 | 3.8203276 | 5.83E-09 | 2.09E-08 | Up |
| PCDH19 | 4.3944749 | 5.93E-13 | 3.32E-12 | Up |
| WNK2 | 3.974712 | 3.06E-18 | 2.87E-17 | Up |
| ZNF367 | 2.0807081 | 3.48E-17 | 3.00E-16 | Up |
| MELK | 4.7541037 | 1.32E-55 | 8.94E-54 | Up |
| SLC7A3 | 3.5263366 | 1.98E-05 | 4.53E-05 | Up |
| TSHR | 2.4481731 | 1.22E-09 | 4.72E-09 | Up |
| PHYHIPL | 2.6834147 | 7.16E-05 | 0.0001512 | Up |
| SLC16A9 | 2.2848288 | 1.31E-07 | 3.99E-07 | Up |
| PHOX2A | 2.5574983 | 0.0012105 | 0.0021385 | Up |
| GJB2 | 6.4267979 | 4.39E-24 | 6.47E-23 | Up |
| SKA3 | 4.166544 | 8.90E-61 | 7.73E-59 | Up |
| DDIAS | 2.2254605 | 2.98E-21 | 3.57E-20 | Up |
| RPL10L | 2.6287069 | 0.0004114 | 0.0007791 | Up |
| TMEM63C | 3.8671961 | 1.93E-16 | 1.53E-15 | Up |
| NGB | 7.9473984 | 1.71E-13 | 1.02E-12 | Up |
| CDX2 | 6.4221021 | 5.52E-08 | 1.76E-07 | Up |
| OTX2 | 8.9990498 | 1.33E-10 | 5.79E-10 | Up |
| OXGR1 | 2.0060767 | 1.35E-05 | 3.18E-05 | Up |
| SOHLH1 | 8.9229699 | 1.99E-12 | 1.06E-11 | Up |
| FRMD7 | 3.2927646 | 3.19E-06 | 8.13E-06 | Up |
| RET | 2.4360975 | 2.34E-05 | 5.30E-05 | Up |
| SLC39A2 | 4.7310517 | 2.37E-14 | 1.53E-13 | Up |
| RNASE7 | 6.4497508 | 4.10E-14 | 2.58E-13 | Up |
| PRAP1 | 6.552184 | 3.15E-09 | 1.17E-08 | Up |
| CYP2C19 | 2.1684108 | 0.0004659 | 0.0008752 | Up |
| C10orf82 | 3.3500759 | 1.28E-05 | 3.02E-05 | Up |
| E2F7 | 4.4535804 | 5.59E-43 | 2.17E-41 | Up |
| LARGE2 | 3.472605 | 2.42E-32 | 5.71E-31 | Up |
| SMCO2 | 2.8953085 | 2.19E-17 | 1.91E-16 | Up |
| SERPINA12 | 3.784709 | 2.01E-05 | 4.60E-05 | Up |
| SLC6A5 | 2.6672521 | 5.84E-05 | 0.0001248 | Up |
| NELL1 | 3.0703772 | 0.0002287 | 0.0004504 | Up |
| PASD1 | 6.6350338 | 4.33E-05 | 9.43E-05 | Up |
| SVOP | 3.4006233 | 3.98E-06 | 1.00E-05 | Up |
| GPT2 | 2.8990869 | 3.25E-34 | 8.39E-33 | Up |
| PPP1R14D | 3.5997274 | 2.85E-07 | 8.31E-07 | Up |
| LRTM2 | 4.3786861 | 2.63E-05 | 5.90E-05 | Up |
| SPIC | 4.542442 | 5.90E-05 | 0.0001261 | Up |
| NETO1 | 5.126497 | 4.13E-18 | 3.84E-17 | Up |
| MOGAT2 | 3.8438736 | 1.81E-06 | 4.77E-06 | Up |
| CYB5R2 | 2.101537 | 9.64E-13 | 5.27E-12 | Up |
| SERPINB7 | 4.8021792 | 9.77E-12 | 4.82E-11 | Up |
| LMO1 | 4.5758686 | 1.35E-12 | 7.28E-12 | Up |
| WDR72 | 5.0454671 | 1.61E-28 | 3.09E-27 | Up |
| CRABP1 | 5.4019165 | 2.63E-10 | 1.10E-09 | Up |
| CENPN | 2.2931666 | 9.61E-28 | 1.76E-26 | Up |
| MCM7 | 2.1569192 | 9.42E-34 | 2.37E-32 | Up |
| A2ML1 | 9.1251344 | 6.12E-24 | 8.93E-23 | Up |
| SLC38A8 | 2.8531642 | 0.000476 | 0.0008929 | Up |
| MC4R | 3.6192741 | 4.10E-08 | 1.33E-07 | Up |
| SERPINB12 | 7.6757312 | 3.63E-12 | 1.88E-11 | Up |
| MMP10 | 6.7405549 | 3.28E-14 | 2.09E-13 | Up |
| HTR3A | 4.673769 | 7.39E-10 | 2.95E-09 | Up |
| PCLAF | 3.8084264 | 1.61E-43 | 6.31E-42 | Up |
| GLYATL1 | 2.6411412 | 1.16E-05 | 2.75E-05 | Up |
| PLK1 | 4.2904782 | 1.28E-72 | 1.86E-70 | Up |
| TAC3 | 2.8202235 | 0.0004301 | 0.0008118 | Up |
| CHP2 | 3.7291981 | 6.40E-07 | 1.79E-06 | Up |
| C15orf48 | 2.2833793 | 2.27E-08 | 7.58E-08 | Up |
| SCG5 | 2.6417049 | 2.46E-07 | 7.23E-07 | Up |
| GREM1 | 4.185649 | 4.21E-20 | 4.60E-19 | Up |
| TGM6 | 6.4106341 | 1.20E-06 | 3.24E-06 | Up |
| NAT16 | 4.4005231 | 5.74E-06 | 1.42E-05 | Up |
| B4GALNT2 | 3.5813492 | 6.53E-07 | 1.82E-06 | Up |
| PRRX2 | 2.4305272 | 2.15E-12 | 1.14E-11 | Up |
| UGT1A6 | 5.8157165 | 2.71E-18 | 2.55E-17 | Up |
| OR51E2 | 4.6451869 | 1.62E-06 | 4.29E-06 | Up |
| LPO | 2.5907619 | 6.31E-08 | 2.00E-07 | Up |
| JSRP1 | 2.0356275 | 1.08E-07 | 3.33E-07 | Up |
| CDT1 | 3.8592747 | 1.57E-55 | 1.06E-53 | Up |
| CYP2S1 | 2.8456372 | 1.49E-11 | 7.22E-11 | Up |
| ANKRD33 | 2.3008627 | 4.59E-05 | 9.97E-05 | Up |
| TTYH1 | 3.7580201 | 3.73E-10 | 1.53E-09 | Up |
| TMEM145 | 3.0138975 | 1.13E-10 | 4.96E-10 | Up |
| NLRP7 | 3.9292392 | 1.57E-08 | 5.35E-08 | Up |
| ATCAY | 4.2826076 | 2.48E-05 | 5.58E-05 | Up |
| LY6D | 8.55876 | 2.61E-16 | 2.04E-15 | Up |
| CHAF1A | 2.2263606 | 1.27E-25 | 2.05E-24 | Up |
| KIFC2 | 2.1730836 | 4.56E-13 | 2.58E-12 | Up |
| TRPV3 | 2.8266117 | 1.76E-11 | 8.44E-11 | Up |
| C19orf48 | 2.2906823 | 5.64E-34 | 1.44E-32 | Up |
| KLK4 | 2.6428511 | 3.45E-06 | 8.75E-06 | Up |
| KLK2 | 2.8029278 | 0.000393 | 0.000746 | Up |
| KLK6 | 7.6017583 | 2.08E-13 | 1.23E-12 | Up |
| KLK13 | 3.530022 | 7.64E-07 | 2.12E-06 | Up |
| KRT80 | 2.1001583 | 8.54E-09 | 3.01E-08 | Up |
| KRT1 | 3.935637 | 7.86E-06 | 1.90E-05 | Up |
| ACER1 | 2.223167 | 0.0003509 | 0.0006716 | Up |
| RCOR2 | 3.8847331 | 2.15E-18 | 2.04E-17 | Up |
| NUDT8 | 2.1399057 | 7.55E-15 | 5.11E-14 | Up |
| TBX10 | 4.3002514 | 4.37E-08 | 1.41E-07 | Up |
| TK1 | 3.7550673 | 1.10E-54 | 7.13E-53 | Up |
| KRT24 | 7.097203 | 1.54E-07 | 4.64E-07 | Up |
| SOST | 6.4222225 | 3.03E-09 | 1.12E-08 | Up |
| RAB26 | 3.4305381 | 1.39E-16 | 1.12E-15 | Up |
| CASKIN1 | 4.5090179 | 8.75E-20 | 9.37E-19 | Up |
| PBK | 4.3937661 | 4.98E-50 | 2.64E-48 | Up |
| OR1F1 | 4.1754103 | 3.05E-07 | 8.88E-07 | Up |
| KCNJ4 | 4.3403687 | 2.83E-07 | 8.27E-07 | Up |
| FAM83B | 6.3906352 | 2.53E-47 | 1.16E-45 | Up |
| GNG4 | 6.044295 | 2.35E-19 | 2.44E-18 | Up |
| FOXI1 | 5.2527087 | 3.87E-05 | 8.49E-05 | Up |
| HIST1H1E | 2.8006062 | 1.10E-12 | 5.99E-12 | Up |
| PPDPFL | 3.8833452 | 0.0007992 | 0.0014503 | Up |
| MTNR1A | 2.2534232 | 3.87E-05 | 8.48E-05 | Up |
| HR | 3.888492 | 4.58E-15 | 3.18E-14 | Up |
| FEN1 | 2.3006061 | 1.29E-37 | 3.96E-36 | Up |
| MTCL1 | 2.158584 | 7.20E-13 | 3.99E-12 | Up |
| GBX2 | 4.2298899 | 6.68E-11 | 3.02E-10 | Up |
| SERINC2 | 2.1747072 | 5.98E-11 | 2.71E-10 | Up |
| COL3A1 | 2.0165129 | 2.55E-08 | 8.48E-08 | Up |
| GDNF | 4.659887 | 1.75E-14 | 1.15E-13 | Up |
| UGT3A2 | 3.4657656 | 9.17E-07 | 2.51E-06 | Up |
| LRP1B | 3.2941174 | 4.56E-07 | 1.30E-06 | Up |
| CA7 | 2.199952 | 0.0002546 | 0.0004982 | Up |
| FAM178B | 2.5600786 | 5.01E-06 | 1.25E-05 | Up |
| SHOX2 | 5.9505544 | 8.88E-29 | 1.73E-27 | Up |
| HTR1E | 4.1776848 | 0.0005731 | 0.0010635 | Up |
| FSTL5 | 5.0406925 | 6.59E-08 | 2.09E-07 | Up |
| NTSR2 | 3.9237305 | 2.26E-05 | 5.12E-05 | Up |
| VCX3A | 4.1339093 | 3.81E-08 | 1.24E-07 | Up |
| RAB3B | 5.1843819 | 4.33E-17 | 3.69E-16 | Up |
| GPRIN1 | 2.8920101 | 4.33E-22 | 5.53E-21 | Up |
| NR0B1 | 8.7601362 | 1.85E-09 | 7.02E-09 | Up |
| GP2 | 5.9730272 | 0.0005319 | 0.0009917 | Up |
| KCNK9 | 4.3802623 | 5.62E-12 | 2.85E-11 | Up |
| COL22A1 | 4.8306444 | 3.58E-12 | 1.86E-11 | Up |
| SPRR1B | 9.2431565 | 5.68E-22 | 7.21E-21 | Up |
| SPRR1A | 9.1252532 | 3.51E-19 | 3.58E-18 | Up |
| SLC38A11 | 4.7776644 | 9.79E-08 | 3.03E-07 | Up |
| CRCT1 | 8.480295 | 1.53E-11 | 7.39E-11 | Up |
| ZNF280A | 7.3094735 | 5.02E-17 | 4.25E-16 | Up |
| CT55 | 3.8434287 | 0.0002657 | 0.0005179 | Up |
| BNC1 | 2.4130781 | 2.65E-06 | 6.82E-06 | Up |
| CKAP2L | 4.1996099 | 2.37E-68 | 2.73E-66 | Up |
| PROKR1 | 2.0145109 | 0.0002832 | 0.0005497 | Up |
| BUB1 | 3.578174 | 1.36E-62 | 1.26E-60 | Up |
| CHRNA5 | 3.3293526 | 2.65E-27 | 4.70E-26 | Up |
| RAC3 | 2.998946 | 3.39E-22 | 4.38E-21 | Up |
| LINGO1 | 2.5375875 | 3.72E-09 | 1.37E-08 | Up |
| ONECUT1 | 4.2238335 | 1.48E-09 | 5.69E-09 | Up |
| WNT10B | 2.7092382 | 1.09E-08 | 3.79E-08 | Up |
| CALML6 | 2.8877792 | 1.28E-10 | 5.57E-10 | Up |
| PYDC1 | 6.3050314 | 3.00E-09 | 1.11E-08 | Up |
| ZPLD1 | 7.21166 | 1.75E-09 | 6.68E-09 | Up |
| NSG2 | 3.8258319 | 0.0002303 | 0.0004533 | Up |
| HOXD4 | 3.2804116 | 4.62E-07 | 1.32E-06 | Up |
| HOXD12 | 5.9777883 | 1.87E-17 | 1.64E-16 | Up |
| CNGB3 | 2.9021718 | 1.12E-12 | 6.06E-12 | Up |
| CDK1 | 3.4999234 | 6.54E-53 | 4.00E-51 | Up |
| EMX2 | 2.558957 | 0.0018464 | 0.0031828 | Up |
| CST1 | 7.9645097 | 1.11E-21 | 1.38E-20 | Up |
| KRT78 | 6.5275796 | 1.22E-18 | 1.19E-17 | Up |
| SDR9C7 | 6.346256 | 1.29E-14 | 8.56E-14 | Up |
| KRT86 | 2.6465692 | 5.26E-06 | 1.30E-05 | Up |
| KRT75 | 8.3513287 | 4.90E-17 | 4.15E-16 | Up |
| KRT6C | 8.8976428 | 1.77E-19 | 1.85E-18 | Up |
| KRT74 | 6.7810492 | 1.74E-24 | 2.63E-23 | Up |
| COX7B2 | 9.5647272 | 2.75E-07 | 8.04E-07 | Up |
| KRT83 | 5.9840569 | 5.26E-11 | 2.40E-10 | Up |
| CDH2 | 2.0127441 | 0.0001732 | 0.0003468 | Up |
| SIX2 | 4.2050053 | 3.19E-14 | 2.04E-13 | Up |
| GTSF1 | 3.1765849 | 8.51E-06 | 2.05E-05 | Up |
| HOXB9 | 7.6996882 | 5.09E-14 | 3.19E-13 | Up |
| GPR37 | 2.1880719 | 4.05E-06 | 1.02E-05 | Up |
| CDCA4 | 2.8460655 | 2.90E-44 | 1.17E-42 | Up |
| BFSP2 | 2.2123975 | 2.63E-06 | 6.77E-06 | Up |
| CEL | 5.1252796 | 2.50E-13 | 1.46E-12 | Up |
| TRH | 3.4788438 | 0.0015043 | 0.0026259 | Up |
| PLAC1 | 6.1285568 | 3.93E-17 | 3.37E-16 | Up |
| HS6ST2 | 3.1074139 | 4.57E-15 | 3.17E-14 | Up |
| C8orf74 | 2.9139245 | 0.000131 | 0.0002666 | Up |
| NRTN | 2.2512131 | 2.34E-10 | 9.84E-10 | Up |
| CLDN20 | 2.4440693 | 2.54E-06 | 6.55E-06 | Up |
| SHCBP1 | 2.8720829 | 1.59E-45 | 6.91E-44 | Up |
| NPTX1 | 2.1713646 | 0.0005055 | 0.0009454 | Up |
| ESCO2 | 3.7260024 | 7.42E-43 | 2.84E-41 | Up |
| KRT19 | 2.2902213 | 4.83E-13 | 2.73E-12 | Up |
| KRT15 | 6.1600441 | 1.70E-20 | 1.91E-19 | Up |
| KRT38 | 4.2541734 | 3.08E-06 | 7.86E-06 | Up |
| KRT13 | 8.1940584 | 3.51E-17 | 3.02E-16 | Up |
| KRT9 | 6.6516803 | 3.59E-18 | 3.36E-17 | Up |
| KRT20 | 5.5839365 | 6.82E-07 | 1.90E-06 | Up |
| CDK5R2 | 5.708955 | 7.50E-11 | 3.37E-10 | Up |
| DLK2 | 3.1336152 | 4.66E-18 | 4.31E-17 | Up |
| LPAR3 | 2.1506338 | 1.10E-09 | 4.30E-09 | Up |
| NEUROD2 | 3.552785 | 9.21E-08 | 2.86E-07 | Up |
| OTP | 8.3023606 | 2.13E-07 | 6.32E-07 | Up |
| ECEL1 | 4.6626644 | 1.01E-09 | 3.96E-09 | Up |
| FGB | 5.8726485 | 7.37E-05 | 0.0001555 | Up |
| DEFB4A | 2.9282196 | 0.0002815 | 0.0005466 | Up |
| PAH | 5.9370776 | 1.68E-07 | 5.06E-07 | Up |
| RRM2 | 4.2465402 | 1.18E-59 | 9.79E-58 | Up |
| CYP4F11 | 6.032987 | 6.76E-12 | 3.39E-11 | Up |
| SCG2 | 3.6637954 | 2.66E-06 | 6.85E-06 | Up |
| FOXB1 | 2.9008783 | 1.41E-05 | 3.30E-05 | Up |
| ZNF556 | 4.7726865 | 4.69E-12 | 2.40E-11 | Up |
| GAP43 | 3.5849566 | 6.31E-10 | 2.53E-09 | Up |
| LRRC15 | 2.6435481 | 9.96E-09 | 3.49E-08 | Up |
| MTBP | 2.380902 | 1.03E-32 | 2.45E-31 | Up |
| C5orf34 | 2.6669792 | 5.39E-29 | 1.06E-27 | Up |
| STARD5 | 2.092641 | 4.97E-16 | 3.79E-15 | Up |
| PRSS27 | 2.5652509 | 8.62E-10 | 3.41E-09 | Up |
| FUT9 | 5.2699663 | 9.58E-09 | 3.36E-08 | Up |
| AGXT | 3.3914979 | 0.0009139 | 0.0016436 | Up |
| ACOT12 | 3.234346 | 0.000102 | 0.0002106 | Up |
| NIPAL4 | 4.605651 | 7.58E-13 | 4.19E-12 | Up |
| MUCL1 | 5.6306004 | 8.13E-09 | 2.88E-08 | Up |
| FADS6 | 6.7987466 | 4.31E-18 | 4.00E-17 | Up |
| OVOL1 | 3.8860291 | 2.70E-21 | 3.25E-20 | Up |
| KRT2 | 4.1411132 | 1.47E-07 | 4.45E-07 | Up |
| LVRN | 4.4649529 | 2.00E-06 | 5.23E-06 | Up |
| MYEOV | 5.3323617 | 3.41E-15 | 2.40E-14 | Up |
| 1-Dec | 3.5480277 | 3.92E-09 | 1.44E-08 | Up |
| ADAMTS20 | 6.4860419 | 2.10E-19 | 2.19E-18 | Up |
| CKS1B | 2.5177161 | 9.21E-28 | 1.69E-26 | Up |
| MAB21L3 | 3.4784806 | 1.42E-10 | 6.16E-10 | Up |
| SYT12 | 4.295994 | 1.20E-13 | 7.24E-13 | Up |
| C11orf86 | 6.1456523 | 1.04E-05 | 2.47E-05 | Up |
| DMRT2 | 3.441105 | 4.23E-09 | 1.54E-08 | Up |
| CST9 | 4.130167 | 7.56E-07 | 2.10E-06 | Up |
| INSM1 | 6.0655895 | 3.67E-07 | 1.06E-06 | Up |
| PPP1R14B | 2.0208225 | 5.31E-28 | 9.87E-27 | Up |
| PC | 2.1056439 | 4.44E-13 | 2.52E-12 | Up |
| UGT2A1 | 4.0177543 | 0.0001844 | 0.0003678 | Up |
| GPRC6A | 2.6913436 | 9.50E-05 | 0.000197 | Up |
| MUC13 | 2.9192587 | 0.0005431 | 0.0010112 | Up |
| JUP | 2.3068505 | 1.30E-22 | 1.73E-21 | Up |
| HAP1 | 5.6117796 | 1.17E-20 | 1.33E-19 | Up |
| TDRD12 | 5.3511022 | 5.39E-09 | 1.94E-08 | Up |
| CBX2 | 3.451941 | 2.11E-21 | 2.57E-20 | Up |
| SPTBN2 | 2.9161015 | 9.86E-30 | 2.04E-28 | Up |
| XXYLT1 | 2.4163007 | 6.25E-27 | 1.09E-25 | Up |
| FBXO45 | 2.1585729 | 3.58E-28 | 6.74E-27 | Up |
| SPERT | 5.9545786 | 1.72E-27 | 3.08E-26 | Up |
| TENT5D | 3.0535114 | 1.43E-05 | 3.34E-05 | Up |
| NWD2 | 4.1312789 | 3.91E-08 | 1.27E-07 | Up |
| SNX31 | 5.2855949 | 3.13E-13 | 1.81E-12 | Up |
| EVX2 | 4.1852406 | 6.12E-09 | 2.19E-08 | Up |
| DIRC1 | 3.4503282 | 1.22E-11 | 5.98E-11 | Up |
| CHRNA9 | 3.0603084 | 5.75E-05 | 0.0001231 | Up |
| EXO1 | 4.4619422 | 3.48E-69 | 4.40E-67 | Up |
| GOLGA6L2 | 6.1035679 | 6.51E-11 | 2.94E-10 | Up |
| CNTNAP2 | 5.4891379 | 1.76E-14 | 1.15E-13 | Up |
| LINGO2 | 2.5153271 | 1.52E-05 | 3.53E-05 | Up |
| KLK15 | 5.0258549 | 1.17E-05 | 2.77E-05 | Up |
| IL20RB | 5.6560612 | 1.34E-18 | 1.30E-17 | Up |
| UGT8 | 2.8612689 | 7.75E-12 | 3.86E-11 | Up |
| BRSK2 | 2.1431405 | 5.72E-05 | 0.0001226 | Up |
| LEP | 3.9898034 | 8.68E-09 | 3.06E-08 | Up |
| ODAPH | 4.6250115 | 9.23E-08 | 2.86E-07 | Up |
| CNIH2 | 2.0224753 | 1.71E-09 | 6.53E-09 | Up |
| GPR149 | 5.2184082 | 1.60E-05 | 3.71E-05 | Up |
| ZIC4 | 6.2817161 | 4.60E-09 | 1.67E-08 | Up |
| CA5A | 2.995287 | 0.0003164 | 0.0006099 | Up |
| UBE2C | 5.1581869 | 1.37E-74 | 2.26E-72 | Up |
| DSG4 | 5.8384171 | 9.19E-11 | 4.07E-10 | Up |
| RTP1 | 3.8333784 | 3.79E-06 | 9.55E-06 | Up |
| WFDC5 | 4.7229146 | 2.75E-11 | 1.29E-10 | Up |
| CCNE2 | 2.5319136 | 3.61E-21 | 4.31E-20 | Up |
| ANKS4B | 4.6611621 | 7.21E-05 | 0.0001523 | Up |
| CHRNA7 | 2.1804332 | 4.33E-08 | 1.40E-07 | Up |
| NRIP3 | 2.2236888 | 8.08E-14 | 4.97E-13 | Up |
| PCSK1 | 4.442804 | 3.36E-09 | 1.24E-08 | Up |
| SUGCT | 2.5740451 | 8.74E-14 | 5.34E-13 | Up |
| RMI2 | 3.3943116 | 3.85E-36 | 1.10E-34 | Up |
| GPR156 | 2.2354593 | 1.01E-07 | 3.13E-07 | Up |
| C11orf44 | 2.1265768 | 0.0023594 | 0.0040052 | Up |
| SFN | 2.7129729 | 1.72E-13 | 1.03E-12 | Up |
| ETV4 | 3.425143 | 1.09E-18 | 1.07E-17 | Up |
| CALCB | 2.8176655 | 2.80E-06 | 7.18E-06 | Up |
| CREG2 | 4.0264637 | 1.76E-15 | 1.28E-14 | Up |
| TMEM270 | 2.5366764 | 2.45E-07 | 7.21E-07 | Up |
| HOXD8 | 2.0218343 | 5.03E-07 | 1.43E-06 | Up |
| CRYBG2 | 2.2457493 | 3.11E-09 | 1.15E-08 | Up |
| GPX2 | 7.4617572 | 1.89E-23 | 2.68E-22 | Up |
| FOXG1 | 6.250192 | 4.79E-07 | 1.36E-06 | Up |
| ENTHD1 | 2.2941577 | 8.49E-08 | 2.65E-07 | Up |
| CIDEA | 3.7978096 | 6.90E-06 | 1.69E-05 | Up |
| OR11H4 | 3.9625859 | 3.88E-05 | 8.50E-05 | Up |
| ATAD5 | 2.1728368 | 4.86E-25 | 7.55E-24 | Up |
| SLC35G1 | 2.2016802 | 1.21E-08 | 4.18E-08 | Up |
| B3GNT4 | 2.7178684 | 1.25E-17 | 1.12E-16 | Up |
| RIMS2 | 5.2672758 | 2.07E-18 | 1.97E-17 | Up |
| VPS37D | 2.1480474 | 7.02E-12 | 3.51E-11 | Up |
| DIRAS1 | 2.1835128 | 4.43E-06 | 1.11E-05 | Up |
| CNBD1 | 3.5201872 | 3.11E-06 | 7.92E-06 | Up |
| LMNB2 | 2.0948419 | 3.19E-25 | 5.05E-24 | Up |
| MAGEB6 | 6.8617116 | 1.75E-10 | 7.49E-10 | Up |
| CDK5R1 | 2.7839301 | 1.54E-22 | 2.03E-21 | Up |
| TCERG1L | 2.6083923 | 0.0015433 | 0.0026899 | Up |
| GRIN1 | 2.4532753 | 3.72E-08 | 1.21E-07 | Up |
| SOX11 | 5.3011189 | 1.88E-09 | 7.12E-09 | Up |
| TYMS | 2.6613261 | 2.01E-30 | 4.33E-29 | Up |
| FUT2 | 2.4756283 | 2.48E-13 | 1.45E-12 | Up |
| MUC20 | 2.5790107 | 6.05E-07 | 1.70E-06 | Up |
| ZDHHC22 | 3.7573701 | 0.0001736 | 0.0003476 | Up |
| TRIM72 | 4.0672547 | 7.94E-08 | 2.49E-07 | Up |
| UMODL1 | 2.0229777 | 0.0003167 | 0.0006103 | Up |
| RPRM | 2.7279695 | 1.65E-05 | 3.83E-05 | Up |
| HASPIN | 2.978653 | 8.38E-35 | 2.22E-33 | Up |
| C12orf54 | 4.3170645 | 6.07E-13 | 3.39E-12 | Up |
| SRRM3 | 3.6598821 | 4.26E-24 | 6.28E-23 | Up |
| KCNJ10 | 2.6752656 | 4.84E-09 | 1.75E-08 | Up |
| SPINK6 | 3.020219 | 0.000842 | 0.0015211 | Up |
| PARD6G | 2.1385669 | 3.74E-21 | 4.46E-20 | Up |
| TMEM151B | 3.6931609 | 3.60E-12 | 1.87E-11 | Up |
| SLITRK1 | 4.7814272 | 6.33E-06 | 1.55E-05 | Up |
| CALML3 | 10.276681 | 6.03E-32 | 1.39E-30 | Up |
| CALML5 | 8.1113997 | 5.51E-13 | 3.10E-12 | Up |
| CCDC185 | 4.2298951 | 5.26E-15 | 3.61E-14 | Up |
| NEUROG2 | 4.7310824 | 1.48E-07 | 4.49E-07 | Up |
| GLDC | 2.9630546 | 4.86E-07 | 1.38E-06 | Up |
| TUBAL3 | 2.0544062 | 3.07E-06 | 7.83E-06 | Up |
| DYNAP | 5.3108615 | 3.02E-07 | 8.78E-07 | Up |
| ERFE | 2.6568395 | 8.20E-11 | 3.66E-10 | Up |
| CPN2 | 2.0224831 | 0.000215 | 0.0004247 | Up |
| CPNE7 | 2.7572581 | 6.84E-11 | 3.08E-10 | Up |
| C5orf46 | 2.5824621 | 2.36E-09 | 8.85E-09 | Up |
| TMEM52 | 2.5154428 | 1.13E-10 | 4.95E-10 | Up |
| FOXE1 | 8.4135087 | 5.50E-25 | 8.49E-24 | Up |
| LGALS7B | 6.9264396 | 1.20E-15 | 8.85E-15 | Up |
| AURKB | 4.6808882 | 1.31E-68 | 1.55E-66 | Up |
| C14orf39 | 2.9963377 | 8.25E-06 | 1.99E-05 | Up |
| TRIML2 | 3.9281601 | 7.32E-05 | 0.0001545 | Up |
| FAM133A | 4.6756217 | 7.43E-08 | 2.34E-07 | Up |
| C12orf42 | 2.6353989 | 5.84E-10 | 2.35E-09 | Up |
| HES7 | 2.3938777 | 2.10E-06 | 5.49E-06 | Up |
| ALOXE3 | 5.0696588 | 5.00E-17 | 4.23E-16 | Up |
| MKRN3 | 5.2403822 | 9.91E-13 | 5.41E-12 | Up |
| ALOX12B | 3.5502158 | 1.53E-08 | 5.23E-08 | Up |
| HTR1D | 2.1882912 | 7.84E-05 | 0.0001648 | Up |
| ARL14 | 3.2419847 | 4.35E-07 | 1.24E-06 | Up |
| OR6C2 | 3.9961591 | 1.04E-05 | 2.47E-05 | Up |
| WDR97 | 2.1477824 | 3.49E-07 | 1.01E-06 | Up |
| APOBEC3B | 3.6014722 | 1.13E-24 | 1.72E-23 | Up |
| ATOH7 | 3.1860695 | 6.59E-10 | 2.64E-09 | Up |
| MRGPRX3 | 5.7166459 | 1.11E-10 | 4.90E-10 | Up |
| NKPD1 | 4.7202131 | 3.52E-22 | 4.54E-21 | Up |
| ABCA13 | 2.2843239 | 3.72E-07 | 1.07E-06 | Up |
| NLRP11 | 3.9477898 | 6.02E-07 | 1.69E-06 | Up |
| B3GNT3 | 3.4422725 | 2.88E-13 | 1.68E-12 | Up |
| NRXN1 | 2.9634683 | 8.71E-05 | 0.0001818 | Up |
| ZNF648 | 3.1324471 | 1.53E-06 | 4.08E-06 | Up |
| TH | 5.1584678 | 1.79E-06 | 4.71E-06 | Up |
| F2 | 6.2791604 | 1.53E-05 | 3.56E-05 | Up |
| SLC9A4 | 5.8424171 | 3.60E-12 | 1.87E-11 | Up |
| ADGRD2 | 5.140627 | 1.96E-15 | 1.42E-14 | Up |
| ALX1 | 4.9570774 | 2.24E-07 | 6.64E-07 | Up |
| MEIOC | 2.423137 | 9.19E-07 | 2.52E-06 | Up |
| CCDC129 | 2.2745163 | 0.0035756 | 0.0059148 | Up |
| TPRXL | 5.0786042 | 2.70E-23 | 3.80E-22 | Up |
| SLC47A2 | 3.1265566 | 1.19E-07 | 3.65E-07 | Up |
| S1PR5 | 2.3957981 | 1.05E-10 | 4.62E-10 | Up |
| OR51E1 | 3.1185195 | 5.59E-14 | 3.49E-13 | Up |
| HOXC9 | 3.6387954 | 3.27E-13 | 1.89E-12 | Up |
| HOXC10 | 7.1753339 | 3.74E-16 | 2.88E-15 | Up |
| MAP6D1 | 2.4815062 | 3.47E-15 | 2.44E-14 | Up |
| OR56B4 | 2.078192 | 2.81E-05 | 6.29E-05 | Up |
| C1orf105 | 3.1911761 | 5.77E-05 | 0.0001235 | Up |
| NQO1 | 2.9071617 | 1.11E-09 | 4.34E-09 | Up |
| PENK | 2.1383439 | 0.0051542 | 0.008328 | Up |
| HIST3H2A | 3.4708311 | 1.33E-17 | 1.19E-16 | Up |
| HEPHL1 | 5.3572051 | 7.96E-13 | 4.39E-12 | Up |
| OFCC1 | 7.2224381 | 2.76E-21 | 3.31E-20 | Up |
| DDN | 3.8443168 | 8.82E-17 | 7.27E-16 | Up |
| SAGE1 | 5.1947559 | 4.93E-05 | 0.0001066 | Up |
| SOX2 | 5.1371636 | 5.07E-19 | 5.08E-18 | Up |
| TMEM45A | 2.1577833 | 3.78E-08 | 1.23E-07 | Up |
| FANCB | 2.9160364 | 3.95E-36 | 1.13E-34 | Up |
| FDCSP | 6.6421725 | 2.31E-08 | 7.70E-08 | Up |
| ANKRD62 | 2.1276527 | 2.30E-05 | 5.20E-05 | Up |
| SIAH2 | 2.1033421 | 8.03E-24 | 1.16E-22 | Up |
| ADGRB1 | 3.690895 | 4.80E-07 | 1.37E-06 | Up |
| GINS3 | 2.0572688 | 4.43E-26 | 7.36E-25 | Up |
| USH1G | 7.4091297 | 7.13E-27 | 1.24E-25 | Up |
| FAM181B | 5.0142324 | 8.42E-19 | 8.30E-18 | Up |
| RGMA | 3.0571469 | 3.50E-14 | 2.22E-13 | Up |
| SHMT2 | 2.1255038 | 1.33E-33 | 3.33E-32 | Up |
| GABRG3 | 5.4136412 | 4.57E-09 | 1.66E-08 | Up |
| NLRP10 | 4.0162643 | 3.36E-06 | 8.52E-06 | Up |
| B4GALNT4 | 5.8277206 | 9.49E-41 | 3.36E-39 | Up |
| NXPH4 | 6.7648134 | 4.09E-41 | 1.47E-39 | Up |
| TEX19 | 5.1585347 | 2.36E-11 | 1.12E-10 | Up |
| KPNA2 | 2.5743947 | 6.77E-41 | 2.41E-39 | Up |
| LHFPL1 | 3.5132786 | 1.02E-13 | 6.22E-13 | Up |
| RNASE10 | 4.7870007 | 4.50E-10 | 1.83E-09 | Up |
| EPHB3 | 2.8222233 | 1.43E-20 | 1.62E-19 | Up |
| VCX | 5.0880272 | 2.32E-12 | 1.22E-11 | Up |
| SNORC | 2.2507877 | 6.26E-15 | 4.28E-14 | Up |
| HS3ST4 | 5.3574146 | 4.17E-06 | 1.05E-05 | Up |
| KCNB2 | 3.0050736 | 6.36E-05 | 0.0001353 | Up |
| PPP1R27 | 3.3769152 | 1.87E-12 | 9.95E-12 | Up |
| GALR2 | 2.687253 | 8.30E-08 | 2.60E-07 | Up |
| TSKU | 2.2247743 | 2.19E-19 | 2.28E-18 | Up |
| SLC35D3 | 5.227135 | 8.68E-07 | 2.39E-06 | Up |
| MAFA | 3.6376911 | 1.48E-07 | 4.49E-07 | Up |
| MAGEB17 | 3.2627309 | 3.74E-05 | 8.24E-05 | Up |
| TCHHL1 | 5.6457735 | 6.16E-10 | 2.48E-09 | Up |
| OTOP3 | 10.080224 | 2.00E-11 | 9.56E-11 | Up |
| SOX1 | 7.0586887 | 2.89E-06 | 7.39E-06 | Up |
| PYCR1 | 2.9921076 | 3.09E-27 | 5.47E-26 | Up |
| OTOP2 | 4.8484005 | 2.68E-10 | 1.12E-09 | Up |
| PCP4 | 3.921385 | 2.55E-06 | 6.57E-06 | Up |
| SLC25A10 | 2.5201542 | 1.19E-27 | 2.17E-26 | Up |
| NKX2-5 | 7.9217293 | 2.26E-14 | 1.46E-13 | Up |
| NEB | 2.1743029 | 1.29E-11 | 6.30E-11 | Up |
| CALHM3 | 3.3705491 | 0.0001382 | 0.0002805 | Up |
| RIPPLY3 | 2.0618872 | 2.97E-06 | 7.59E-06 | Up |
| GPR19 | 3.3829095 | 5.15E-25 | 7.98E-24 | Up |
| CALN1 | 2.8887516 | 0.0006807 | 0.0012494 | Up |
| GABRR3 | 2.3839481 | 0.0007242 | 0.0013241 | Up |
| GBP6 | 5.6370114 | 1.25E-17 | 1.12E-16 | Up |
| PMCH | 2.2107938 | 1.10E-06 | 2.99E-06 | Up |
| 11-Mar | 5.195197 | 2.74E-05 | 6.16E-05 | Up |
| PSG9 | 4.7152446 | 6.74E-06 | 1.65E-05 | Up |
| IFNL2 | 4.7457274 | 4.61E-08 | 1.49E-07 | Up |
| NPBWR1 | 5.9923769 | 4.05E-13 | 2.31E-12 | Up |
| ASCL2 | 2.3309454 | 5.46E-11 | 2.48E-10 | Up |
| ACP7 | 6.0556302 | 3.34E-14 | 2.13E-13 | Up |
| TRAIP | 2.4707386 | 5.35E-35 | 1.44E-33 | Up |
| CHEK2 | 2.2944818 | 1.39E-31 | 3.18E-30 | Up |
| FOXL2 | 7.982707 | 1.92E-17 | 1.69E-16 | Up |
| B3GALT5 | 2.375413 | 6.24E-05 | 0.0001329 | Up |
| SLC35F3 | 2.8976147 | 1.47E-08 | 5.03E-08 | Up |
| EMILIN3 | 3.9031113 | 1.73E-09 | 6.58E-09 | Up |
| PNMA3 | 2.5251469 | 3.29E-07 | 9.54E-07 | Up |
| IQGAP3 | 3.8560255 | 6.55E-45 | 2.71E-43 | Up |
| SCN5A | 2.4946098 | 6.24E-08 | 1.99E-07 | Up |
| LRRC55 | 2.1690666 | 0.0009728 | 0.0017423 | Up |
| KCNH8 | 2.7679139 | 9.71E-08 | 3.01E-07 | Up |
| NPW | 4.2266716 | 4.60E-13 | 2.60E-12 | Up |
| TBX1 | 2.1600854 | 1.04E-09 | 4.08E-09 | Up |
| CNTN2 | 5.8679066 | 5.12E-07 | 1.45E-06 | Up |
| SPRR4 | 9.4734859 | 8.37E-13 | 4.60E-12 | Up |
| C1QTNF12 | 4.0839752 | 4.97E-14 | 3.11E-13 | Up |
| HIST2H2AC | 2.022811 | 2.14E-11 | 1.02E-10 | Up |
| KCNK12 | 3.225702 | 3.93E-10 | 1.61E-09 | Up |
| HIST2H2AB | 2.1544598 | 2.17E-06 | 5.64E-06 | Up |
| S100A7A | 9.1171022 | 2.14E-13 | 1.26E-12 | Up |
| SRPK3 | 2.0025478 | 1.83E-07 | 5.47E-07 | Up |
| KRTAP19-1 | 8.6037937 | 1.63E-13 | 9.71E-13 | Up |
| HIST1H1B | 4.5439898 | 8.09E-18 | 7.35E-17 | Up |
| PKP3 | 2.4139185 | 4.22E-25 | 6.60E-24 | Up |
| MAP7D2 | 3.8373815 | 4.93E-12 | 2.52E-11 | Up |
| KCND2 | 2.2980257 | 3.50E-10 | 1.44E-09 | Up |
| KNTC1 | 2.3878218 | 2.43E-39 | 8.09E-38 | Up |
| BPIFC | 5.0233827 | 6.14E-08 | 1.95E-07 | Up |
| POU3F2 | 5.2383732 | 5.41E-09 | 1.95E-08 | Up |
| GAST | 7.6091836 | 3.01E-16 | 2.34E-15 | Up |
| CEND1 | 2.7003298 | 7.98E-08 | 2.50E-07 | Up |
| PIWIL3 | 2.8339092 | 0.0057763 | 0.0092636 | Up |
| CDCA2 | 4.1693448 | 1.48E-52 | 8.69E-51 | Up |
| RALYL | 5.7718115 | 1.18E-05 | 2.79E-05 | Up |
| CLDN6 | 4.1038872 | 1.58E-05 | 3.68E-05 | Up |
| SATL1 | 3.0208672 | 4.18E-05 | 9.14E-05 | Up |
| OR6C70 | 5.7812609 | 7.91E-08 | 2.48E-07 | Up |
| MUC6 | 2.2189775 | 0.0027279 | 0.0045868 | Up |
| IFNE | 3.0420624 | 2.30E-06 | 5.97E-06 | Up |
| RFX6 | 2.7458253 | 0.0060074 | 0.0096164 | Up |
| LRRC14B | 5.4638387 | 6.84E-09 | 2.44E-08 | Up |
| SEMA4B | 2.1996923 | 2.27E-15 | 1.63E-14 | Up |
| MROH2A | 4.8405761 | 3.66E-08 | 1.19E-07 | Up |
| SGCZ | 4.8915193 | 3.32E-07 | 9.62E-07 | Up |
| KRT76 | 7.5951792 | 4.96E-09 | 1.79E-08 | Up |
| MYADML2 | 2.6184081 | 6.49E-10 | 2.60E-09 | Up |
| HIST1H2BL | 2.3422556 | 1.33E-10 | 5.78E-10 | Up |
| MIXL1 | 2.7140031 | 1.56E-13 | 9.34E-13 | Up |
| MAGEA11 | 10.780977 | 6.17E-16 | 4.66E-15 | Up |
| C12orf56 | 6.9037452 | 2.81E-39 | 9.29E-38 | Up |
| HS6ST3 | 2.1352405 | 0.0024765 | 0.0041918 | Up |
| KRT6B | 9.103429 | 8.83E-26 | 1.44E-24 | Up |
| PARPBP | 3.0884654 | 6.41E-35 | 1.71E-33 | Up |
| FAM131C | 5.5166704 | 5.24E-36 | 1.49E-34 | Up |
| DLK1 | 7.4116421 | 4.43E-05 | 9.63E-05 | Up |
| AHNAK2 | 2.6662523 | 3.96E-10 | 1.62E-09 | Up |
| DBX2 | 3.3213051 | 0.0006227 | 0.0011493 | Up |
| PDIA2 | 3.6277805 | 1.32E-07 | 4.02E-07 | Up |
| NDUFA4L2 | 2.915195 | 4.84E-11 | 2.21E-10 | Up |
| POU3F1 | 4.1611949 | 2.54E-12 | 1.34E-11 | Up |
| PRAME | 8.7981299 | 3.14E-30 | 6.70E-29 | Up |
| C11orf87 | 2.4249628 | 0.0001995 | 0.0003962 | Up |
| KCNQ5 | 2.8649138 | 5.49E-13 | 3.09E-12 | Up |
| NAT8L | 3.1117372 | 1.28E-08 | 4.42E-08 | Up |
| TMPRSS11B | 7.9937027 | 9.40E-10 | 3.70E-09 | Up |
| NKAIN3 | 2.6243769 | 0.0013233 | 0.0023243 | Up |
| LCE3A | 7.3505195 | 7.36E-10 | 2.94E-09 | Up |
| LCE3E | 8.0351318 | 3.19E-10 | 1.32E-09 | Up |
| PLK5 | 2.9896612 | 7.93E-07 | 2.19E-06 | Up |
| LEMD1 | 3.4934285 | 5.18E-11 | 2.36E-10 | Up |
| ATP4B | 6.0830412 | 4.64E-05 | 0.0001007 | Up |
| HTR3E | 3.1391201 | 0.0002895 | 0.0005612 | Up |
| C15orf41 | 2.021966 | 1.85E-20 | 2.07E-19 | Up |
| KRT5 | 7.4716905 | 1.05E-29 | 2.18E-28 | Up |
| NBPF6 | 2.6878005 | 0.0004034 | 0.0007649 | Up |
| CYP4F2 | 4.1169044 | 3.45E-07 | 9.99E-07 | Up |
| KIF18B | 5.0120966 | 1.38E-60 | 1.18E-58 | Up |
| BPIFB4 | 4.6256238 | 8.54E-07 | 2.35E-06 | Up |
| SAPCD2 | 5.0060752 | 3.53E-42 | 1.33E-40 | Up |
| EDARADD | 3.3738291 | 3.81E-15 | 2.67E-14 | Up |
| LCE1E | 4.8963534 | 1.18E-08 | 4.09E-08 | Up |
| GABRA5 | 7.4672144 | 5.27E-10 | 2.13E-09 | Up |
| THBS2 | 2.202548 | 5.12E-11 | 2.34E-10 | Up |
| KRT10 | 2.3917422 | 5.87E-11 | 2.67E-10 | Up |
| KRT3 | 5.3486502 | 1.52E-14 | 1.00E-13 | Up |
| KLK12 | 5.5825708 | 1.66E-07 | 5.01E-07 | Up |
| CYP4F8 | 2.6674922 | 0.0003507 | 0.0006715 | Up |
| CYP4F3 | 5.1867047 | 1.64E-09 | 6.26E-09 | Up |
| HPDL | 4.4993076 | 9.77E-21 | 1.12E-19 | Up |
| CYP27C1 | 3.808071 | 2.22E-11 | 1.05E-10 | Up |
| OR10H1 | 3.0686378 | 1.83E-05 | 4.21E-05 | Up |
| MPPED1 | 6.4380298 | 1.03E-11 | 5.05E-11 | Up |
| FOXE3 | 4.5012644 | 4.66E-09 | 1.69E-08 | Up |
| VSIG10L | 2.1132597 | 3.81E-08 | 1.24E-07 | Up |
| KRT16 | 9.2486076 | 1.66E-27 | 2.99E-26 | Up |
| SELENOV | 5.0926597 | 3.12E-07 | 9.07E-07 | Up |
| KRT14 | 8.9491421 | 1.17E-16 | 9.52E-16 | Up |
| QRFPR | 3.6231438 | 0.0002202 | 0.0004347 | Up |
| ERCC6L | 4.0913376 | 3.09E-48 | 1.48E-46 | Up |
| TNFRSF18 | 3.7515799 | 5.77E-23 | 7.91E-22 | Up |
| C1QL4 | 5.1738813 | 1.06E-13 | 6.41E-13 | Up |
| SERPINA11 | 4.8051019 | 1.28E-08 | 4.42E-08 | Up |
| ESPN | 3.4187911 | 4.74E-21 | 5.59E-20 | Up |
| TMPRSS11A | 9.7143856 | 3.14E-19 | 3.22E-18 | Up |
| LYPD6 | 2.1999237 | 1.61E-08 | 5.47E-08 | Up |
| AKR1C1 | 4.4676488 | 9.01E-11 | 4.00E-10 | Up |
| FOXD3 | 6.1390451 | 1.48E-23 | 2.13E-22 | Up |
| SPATA21 | 3.2703808 | 9.66E-05 | 0.0002001 | Up |
| MAGED4B | 2.5536607 | 1.86E-07 | 5.55E-07 | Up |
| NPSR1 | 8.4829453 | 2.13E-15 | 1.53E-14 | Up |
| FAM9C | 4.1933458 | 0.0015795 | 0.002749 | Up |
| ATP13A5 | 3.4069532 | 5.51E-06 | 1.36E-05 | Up |
| AGMO | 2.4951852 | 7.35E-07 | 2.04E-06 | Up |
| COX8C | 5.1239009 | 6.11E-06 | 1.50E-05 | Up |
| PLEKHN1 | 2.9817504 | 3.86E-17 | 3.31E-16 | Up |
| PERM1 | 2.8227272 | 5.78E-16 | 4.38E-15 | Up |
| ERC2 | 2.2049627 | 2.52E-09 | 9.46E-09 | Up |
| ERAS | 2.4913143 | 0.0014196 | 0.0024846 | Up |
| AMTN | 8.6316725 | 1.90E-12 | 1.01E-11 | Up |
| CXorf67 | 4.0905803 | 0.0004164 | 0.000788 | Up |
| SLC18A3 | 8.2244159 | 2.66E-12 | 1.40E-11 | Up |
| FANCA | 2.4850678 | 1.63E-30 | 3.53E-29 | Up |
| LIN28B | 7.240794 | 1.97E-07 | 5.86E-07 | Up |
| FAM69C | 5.358486 | 8.09E-10 | 3.21E-09 | Up |
| DNAH17 | 2.0288142 | 3.69E-06 | 9.33E-06 | Up |
| EIF4EBP1 | 3.129385 | 5.55E-21 | 6.51E-20 | Up |
| ASCL4 | 6.8852651 | 1.16E-07 | 3.56E-07 | Up |
| SHISA7 | 3.024725 | 4.82E-09 | 1.75E-08 | Up |
| AC091057.1 | 2.2091589 | 5.46E-21 | 6.41E-20 | Up |
| CPSF4L | 2.0833341 | 2.12E-05 | 4.82E-05 | Up |
| C17orf99 | 3.8201677 | 1.20E-15 | 8.82E-15 | Up |
| CLCN1 | 2.2169347 | 1.54E-06 | 4.08E-06 | Up |
| WNT7B | 2.3761729 | 5.66E-11 | 2.57E-10 | Up |
| MESP2 | 3.225011 | 6.93E-10 | 2.77E-09 | Up |
| FAM25A | 6.9166982 | 2.09E-10 | 8.86E-10 | Up |
| TMEM215 | 2.751532 | 0.0030042 | 0.0050184 | Up |
| OTOG | 4.8043884 | 7.53E-11 | 3.38E-10 | Up |
| HEPACAM2 | 4.3238881 | 2.25E-05 | 5.10E-05 | Up |
| POTEE | 2.1734455 | 1.78E-05 | 4.09E-05 | Up |
| IL17REL | 3.7686716 | 5.64E-07 | 1.59E-06 | Up |
| OR7A5 | 5.7141384 | 3.07E-13 | 1.78E-12 | Up |
| RUFY4 | 2.0728613 | 5.68E-07 | 1.60E-06 | Up |
| IGFL1 | 7.7554126 | 3.96E-13 | 2.26E-12 | Up |
| SBK1 | 2.2597246 | 1.23E-08 | 4.24E-08 | Up |
| SLC38A3 | 3.119407 | 1.35E-06 | 3.61E-06 | Up |
| PRR19 | 2.5465424 | 7.39E-24 | 1.08E-22 | Up |
| ZP3 | 2.3487218 | 4.21E-15 | 2.93E-14 | Up |
| C10orf99 | 8.9500109 | 1.67E-20 | 1.88E-19 | Up |
| JAKMIP3 | 2.9647391 | 4.50E-08 | 1.45E-07 | Up |
| CLEC2A | 6.3142675 | 4.13E-05 | 9.03E-05 | Up |
| H2AFX | 2.1578034 | 3.68E-28 | 6.92E-27 | Up |
| LCTL | 2.0111323 | 9.83E-15 | 6.57E-14 | Up |
| NCCRP1 | 5.4592101 | 1.63E-16 | 1.30E-15 | Up |
| KRTDAP | 7.4155613 | 2.40E-11 | 1.13E-10 | Up |
| FBLL1 | 2.7477478 | 2.13E-05 | 4.85E-05 | Up |
| NKAIN2 | 5.3826702 | 3.94E-17 | 3.37E-16 | Up |
| PAQR9 | 2.0711347 | 0.0043201 | 0.007061 | Up |
| FAM72B | 3.3034922 | 9.52E-33 | 2.28E-31 | Up |
| IGFL3 | 7.723108 | 5.60E-12 | 2.84E-11 | Up |
| OR56A5 | 3.9161079 | 4.04E-06 | 1.02E-05 | Up |
| FZD9 | 3.1269118 | 2.00E-10 | 8.50E-10 | Up |
| SPRED3 | 2.3304008 | 1.29E-10 | 5.64E-10 | Up |
| HMX2 | 4.9159919 | 9.20E-09 | 3.23E-08 | Up |
| GLRA4 | 2.5602214 | 0.0027313 | 0.0045922 | Up |
| ENTPD8 | 3.2854373 | 6.23E-07 | 1.75E-06 | Up |
| GJB3 | 5.150123 | 1.72E-30 | 3.70E-29 | Up |
| SBSN | 7.6562902 | 6.30E-14 | 3.92E-13 | Up |
| RNF222 | 3.7178324 | 1.99E-09 | 7.52E-09 | Up |
| CGB5 | 5.2067928 | 1.34E-05 | 3.16E-05 | Up |
| FAM111B | 3.2493341 | 2.87E-25 | 4.55E-24 | Up |
| GAGE2A | 6.6897729 | 0.0001393 | 0.0002825 | Up |
| IL1RAPL2 | 4.6924145 | 1.23E-11 | 5.99E-11 | Up |
| ANKRD34B | 2.9937234 | 6.20E-06 | 1.52E-05 | Up |
| KRT77 | 9.1894742 | 2.03E-15 | 1.46E-14 | Up |
| GJB5 | 6.0021627 | 1.43E-33 | 3.57E-32 | Up |
| C6orf222 | 3.5516215 | 1.12E-09 | 4.38E-09 | Up |
| ALG1L | 4.4053226 | 2.77E-29 | 5.57E-28 | Up |
| SH2D5 | 4.9568643 | 2.19E-17 | 1.92E-16 | Up |
| GJB4 | 5.5094641 | 4.35E-20 | 4.75E-19 | Up |
| MYBPC1 | 7.0354208 | 3.02E-08 | 9.94E-08 | Up |
| SPOCK3 | 2.1582052 | 0.0004484 | 0.0008441 | Up |
| MYT1 | 2.2993665 | 9.28E-05 | 0.0001928 | Up |
| AKR1C3 | 3.349087 | 2.85E-08 | 9.43E-08 | Up |
| ATP2A1 | 2.1876981 | 4.93E-15 | 3.40E-14 | Up |
| CGB7 | 2.8099117 | 5.12E-12 | 2.61E-11 | Up |
| ADH7 | 6.1053103 | 6.16E-12 | 3.11E-11 | Up |
| ELAVL3 | 3.0668003 | 0.0006222 | 0.0011485 | Up |
| NUDT11 | 3.1590732 | 2.19E-13 | 1.28E-12 | Up |
| NBPF4 | 2.9332682 | 0.0001805 | 0.0003606 | Up |
| ARL9 | 2.6550913 | 7.75E-12 | 3.86E-11 | Up |
| SPTSSB | 5.3954183 | 1.92E-15 | 1.39E-14 | Up |
| FAM72A | 2.3728258 | 2.40E-21 | 2.89E-20 | Up |
| XRCC2 | 3.9129904 | 2.86E-48 | 1.37E-46 | Up |
| POTEF | 3.1435457 | 5.71E-08 | 1.82E-07 | Up |
| MMP1 | 6.4401601 | 1.87E-20 | 2.08E-19 | Up |
| UGT2B15 | 4.0780375 | 1.14E-05 | 2.70E-05 | Up |
| SLC30A10 | 3.6692002 | 1.46E-07 | 4.43E-07 | Up |
| LCE1B | 4.5579841 | 3.46E-08 | 1.13E-07 | Up |
| HIST1H2AI | 3.8182445 | 6.02E-18 | 5.52E-17 | Up |
| CLPSL2 | 4.4417735 | 5.36E-08 | 1.72E-07 | Up |
| S100A2 | 7.0293982 | 8.85E-26 | 1.44E-24 | Up |
| HIST1H2AG | 3.172276 | 9.16E-18 | 8.27E-17 | Up |
| SPRR2B | 6.9732433 | 2.05E-08 | 6.90E-08 | Up |
| CHRNG | 3.1303883 | 2.73E-07 | 7.97E-07 | Up |
| ADA | 2.288364 | 9.88E-16 | 7.33E-15 | Up |
| PATE2 | 2.1921635 | 0.0001339 | 0.0002722 | Up |
| HIST1H2AD | 3.4207618 | 2.23E-16 | 1.75E-15 | Up |
| SCN8A | 2.7700865 | 4.36E-16 | 3.34E-15 | Up |
| HIST1H4C | 2.1635442 | 7.20E-08 | 2.27E-07 | Up |
| KRT35 | 4.2115801 | 4.06E-05 | 8.89E-05 | Up |
| LCE1C | 7.4909643 | 3.19E-13 | 1.85E-12 | Up |
| SLC6A17 | 3.2967583 | 6.25E-07 | 1.75E-06 | Up |
| IFNL3 | 3.2868862 | 9.94E-06 | 2.37E-05 | Up |
| HIST1H3J | 4.7155501 | 8.42E-19 | 8.30E-18 | Up |
| MAGEA6 | 10.102758 | 7.27E-14 | 4.49E-13 | Up |
| PIWIL2 | 2.261663 | 8.04E-05 | 0.0001688 | Up |
| CYSRT1 | 2.0996342 | 1.97E-07 | 5.88E-07 | Up |
| HIST1H4J | 2.4038839 | 9.72E-10 | 3.82E-09 | Up |
| C6orf141 | 2.5757029 | 8.80E-08 | 2.74E-07 | Up |
| RAD54B | 2.4993686 | 1.52E-33 | 3.78E-32 | Up |
| BLM | 2.8513465 | 2.24E-41 | 8.18E-40 | Up |
| CYP2B6 | 4.5458477 | 0.0008526 | 0.0015391 | Up |
| HIST1H3D | 3.0777572 | 8.11E-17 | 6.70E-16 | Up |
| OGDHL | 3.850332 | 2.09E-12 | 1.11E-11 | Up |
| ZNF695 | 5.278811 | 8.38E-25 | 1.28E-23 | Up |
| COL4A6 | 2.9394106 | 9.00E-09 | 3.17E-08 | Up |
| DMBX1 | 6.0241954 | 5.93E-16 | 4.48E-15 | Up |
| MYH6 | 4.9480886 | 1.13E-10 | 4.98E-10 | Up |
| SERPINB2 | 4.1454161 | 2.64E-09 | 9.89E-09 | Up |
| SERPINB13 | 7.9479075 | 1.74E-20 | 1.95E-19 | Up |
| LHFPL5 | 4.7452087 | 2.16E-12 | 1.14E-11 | Up |
| HOXC6 | 2.9852179 | 3.97E-13 | 2.27E-12 | Up |
| PRB3 | 4.2560598 | 2.00E-14 | 1.30E-13 | Up |
| UGT2B17 | 3.1714267 | 2.04E-05 | 4.65E-05 | Up |
| ERO1A | 2.0837122 | 1.33E-14 | 8.78E-14 | Up |
| AADACL2 | 2.9880152 | 0.0001244 | 0.0002541 | Up |
| ELOVL2 | 2.4464786 | 2.59E-07 | 7.59E-07 | Up |
| KEL | 2.2875794 | 3.58E-05 | 7.89E-05 | Up |
| ZNF560 | 3.6185049 | 0.0002941 | 0.0005696 | Up |
| AVPR1B | 2.1991429 | 0.0044382 | 0.0072469 | Up |
| AKR1B10 | 9.7471825 | 8.00E-22 | 1.00E-20 | Up |
| TMPRSS11F | 8.6245979 | 1.57E-20 | 1.76E-19 | Up |
| MB | 2.2080479 | 2.86E-05 | 6.40E-05 | Up |
| CACNA1E | 3.3680451 | 1.65E-07 | 4.97E-07 | Up |
| DCAF12L2 | 4.3588935 | 4.65E-08 | 1.50E-07 | Up |
| NRARP | 2.3468435 | 6.87E-14 | 4.26E-13 | Up |
| KRTAP4-1 | 7.6369851 | 1.33E-27 | 2.40E-26 | Up |
| B3GNT6 | 3.2969976 | 0.000184 | 0.0003671 | Up |
| C2CD4A | 3.188076 | 2.09E-11 | 9.97E-11 | Up |
| WDHD1 | 2.5069879 | 4.12E-40 | 1.42E-38 | Up |
| AKR1C4 | 3.9708457 | 1.42E-06 | 3.79E-06 | Up |
| MAGEA1 | 10.406608 | 3.73E-13 | 2.14E-12 | Up |
| ABCA4 | 3.601979 | 1.88E-06 | 4.93E-06 | Up |
| ANKRD13B | 2.2599257 | 2.07E-13 | 1.22E-12 | Up |
| TEX45 | 2.0635157 | 1.34E-07 | 4.10E-07 | Up |
| SMOC1 | 2.2601818 | 0.0001776 | 0.0003553 | Up |
| PLXNB3 | 3.192378 | 1.75E-22 | 2.30E-21 | Up |
| EPS8L3 | 3.6307177 | 0.0001959 | 0.0003897 | Up |
| APCDD1L | 4.9075812 | 2.48E-15 | 1.77E-14 | Up |
| ALPK2 | 3.7665505 | 1.92E-12 | 1.02E-11 | Up |
| PAX9 | 3.6596239 | 3.52E-15 | 2.47E-14 | Up |
| ARHGAP11A | 3.0848677 | 3.32E-52 | 1.91E-50 | Up |
| C1orf68 | 6.3314229 | 6.54E-09 | 2.34E-08 | Up |
| PNMA5 | 7.1476079 | 8.09E-08 | 2.53E-07 | Up |
| PRC1 | 3.7468816 | 2.27E-61 | 2.03E-59 | Up |
| L1CAM | 2.4767954 | 1.21E-05 | 2.85E-05 | Up |
| POU3F3 | 5.1482919 | 6.44E-05 | 0.0001368 | Up |
| CSAG1 | 8.5787016 | 8.81E-11 | 3.91E-10 | Up |
| METTL11B | 6.3306726 | 2.81E-23 | 3.95E-22 | Up |
| CENPW | 2.9621246 | 8.25E-32 | 1.89E-30 | Up |
| LOR | 4.0319014 | 2.18E-06 | 5.67E-06 | Up |
| PRR9 | 3.3951441 | 0.0009834 | 0.0017601 | Up |
| SPRR2E | 9.2085953 | 7.90E-15 | 5.35E-14 | Up |
| KPRP | 8.5636321 | 1.58E-12 | 8.49E-12 | Up |
| PLPP4 | 4.6807199 | 3.51E-23 | 4.92E-22 | Up |
| HIST2H2BF | 2.6584508 | 6.86E-11 | 3.09E-10 | Up |
| PNLIPRP3 | 5.8858573 | 2.03E-05 | 4.63E-05 | Up |
| RIPPLY2 | 4.197031 | 7.40E-07 | 2.05E-06 | Up |
| C10orf62 | 2.2709361 | 6.56E-05 | 0.0001391 | Up |
| ZYG11A | 5.683315 | 5.16E-28 | 9.62E-27 | Up |
| LCN8 | 3.1565862 | 0.0037657 | 0.0062047 | Up |
| CT83 | 5.6507912 | 1.52E-05 | 3.53E-05 | Up |
| LIPK | 7.742573 | 6.12E-15 | 4.19E-14 | Up |
| LRIT2 | 3.1120506 | 1.55E-06 | 4.11E-06 | Up |
| FOXO6 | 2.4866582 | 4.53E-10 | 1.84E-09 | Up |
| CLPSL1 | 4.0473841 | 2.16E-05 | 4.90E-05 | Up |
| TXNDC8 | 2.1838911 | 0.0012844 | 0.0022589 | Up |
| COL5A2 | 2.0718582 | 1.06E-09 | 4.14E-09 | Up |
| TMEM235 | 2.525024 | 0.0020921 | 0.0035793 | Up |
| C6orf10 | 3.3944137 | 4.71E-06 | 1.17E-05 | Up |
| BTBD17 | 2.3059158 | 0.0037648 | 0.006204 | Up |
| LY6G6C | 5.2464378 | 2.64E-12 | 1.39E-11 | Up |
| PSORS1C2 | 3.0513267 | 1.16E-06 | 3.12E-06 | Up |
| CDSN | 3.5208268 | 4.80E-05 | 0.000104 | Up |
| C6orf15 | 5.8901799 | 2.04E-07 | 6.07E-07 | Up |
| KRTAP5-10 | 2.1442285 | 0.0010207 | 0.0018209 | Up |
| TRIM15 | 4.7907754 | 7.31E-09 | 2.60E-08 | Up |
| TRIM31 | 2.3707234 | 2.14E-05 | 4.86E-05 | Up |
| DISP3 | 2.9107586 | 4.18E-06 | 1.05E-05 | Up |
| C5orf60 | 2.3017692 | 7.13E-06 | 1.74E-05 | Up |
| SPDYC | 4.7521808 | 4.19E-06 | 1.05E-05 | Up |
| IGFL2 | 2.7199434 | 1.70E-06 | 4.49E-06 | Up |
| IGFL4 | 5.7140183 | 8.56E-18 | 7.75E-17 | Up |
| KRT40 | 3.5983021 | 0.000197 | 0.0003917 | Up |
| GRXCR2 | 2.9522842 | 1.77E-07 | 5.31E-07 | Up |
| CD177 | 3.4874599 | 4.64E-07 | 1.32E-06 | Up |
| PSG5 | 7.8969714 | 5.10E-10 | 2.07E-09 | Up |
| FBXO47 | 2.0549702 | 0.0001426 | 0.0002887 | Up |
| PRSS1 | 3.8577577 | 1.21E-06 | 3.25E-06 | Up |
| CLLU1OS | 3.4112483 | 4.85E-07 | 1.38E-06 | Up |
| LGALS7 | 7.9059856 | 2.61E-13 | 1.52E-12 | Up |
| TMEM88B | 3.2413725 | 9.58E-09 | 3.36E-08 | Up |
| ARID3C | 2.1755912 | 4.12E-07 | 1.18E-06 | Up |
| CCDC144NL | 3.3447492 | 8.58E-05 | 0.0001792 | Up |
| LGR4 | 2.1436969 | 1.96E-16 | 1.56E-15 | Up |
| TRBV20OR9-2 | 5.0443394 | 2.34E-09 | 8.79E-09 | Up |
| CTXN3 | 2.654031 | 1.78E-05 | 4.09E-05 | Up |
| SLCO6A1 | 5.4193897 | 3.27E-14 | 2.08E-13 | Up |
| KRT6A | 9.7524641 | 3.55E-38 | 1.12E-36 | Up |
| KRT81 | 4.1236774 | 1.74E-10 | 7.47E-10 | Up |
| TP53TG3D | 2.1670763 | 9.35E-05 | 0.0001941 | Up |
| CPT1B | 2.2956207 | 1.36E-12 | 7.34E-12 | Up |
| ARSH | 4.7068433 | 1.28E-15 | 9.43E-15 | Up |
| MANSC4 | 3.1558783 | 2.92E-05 | 6.53E-05 | Up |
| CYP2D7 | 2.2932297 | 2.88E-11 | 1.35E-10 | Up |
| GMNC | 3.8794447 | 0.0002211 | 0.0004362 | Up |
| KRTAP5-6 | 3.7198629 | 8.24E-08 | 2.58E-07 | Up |
| ONECUT3 | 5.504133 | 2.03E-10 | 8.60E-10 | Up |
| SERPINB4 | 7.2741688 | 1.25E-16 | 1.02E-15 | Up |
| SERPINB5 | 7.3780155 | 1.80E-37 | 5.47E-36 | Up |
| ELOA2 | 2.9341533 | 1.12E-07 | 3.46E-07 | Up |
| FOXL2NB | 7.7922335 | 5.86E-14 | 3.66E-13 | Up |
| TMEM200C | 2.5050807 | 3.93E-09 | 1.44E-08 | Up |
| PRSS50 | 4.1225946 | 3.85E-09 | 1.41E-08 | Up |
| DIO2 | 3.5044483 | 1.27E-13 | 7.66E-13 | Up |
| IGKJ5 | 2.4212512 | 3.87E-07 | 1.11E-06 | Up |
| IGKV5-2 | 2.4900846 | 2.35E-05 | 5.31E-05 | Up |
| IGKV6-21 | 2.2140262 | 0.0001196 | 0.0002447 | Up |
| IGKV3D-20 | 2.1556853 | 1.66E-05 | 3.85E-05 | Up |
| IGLV4-60 | 2.2201368 | 0.0004527 | 0.0008517 | Up |
| IGLV6-57 | 2.1524633 | 1.88E-05 | 4.30E-05 | Up |
| IGLV11-55 | 2.9004219 | 5.17E-05 | 0.0001113 | Up |
| IGLV5-52 | 2.4460717 | 1.43E-05 | 3.33E-05 | Up |
| IGLV1-47 | 2.2879559 | 3.59E-06 | 9.08E-06 | Up |
| IGLV5-45 | 2.1354828 | 0.0004386 | 0.0008271 | Up |
| IGLV1-44 | 2.077338 | 4.40E-06 | 1.10E-05 | Up |
| IGLV7-43 | 2.2742298 | 3.17E-05 | 7.06E-05 | Up |
| IGLV2-33 | 2.3939533 | 3.01E-05 | 6.72E-05 | Up |
| IGLV3-16 | 2.3569616 | 2.54E-05 | 5.73E-05 | Up |
| IGLV3-9 | 2.2523256 | 0.0001298 | 0.0002643 | Up |
| IGLV4-3 | 2.0411061 | 0.0012707 | 0.002237 | Up |
| IGLJ1 | 2.3232082 | 2.95E-05 | 6.58E-05 | Up |
| IGLJ2 | 2.1476501 | 1.48E-06 | 3.95E-06 | Up |
| IGLC7 | 2.1163765 | 0.0001484 | 0.0002998 | Up |
| IGHE | 2.8643355 | 4.53E-06 | 1.13E-05 | Up |
| IGHG4 | 3.6429094 | 5.36E-10 | 2.16E-09 | Up |
| IGHG2 | 2.640309 | 2.35E-08 | 7.86E-08 | Up |
| IGHG3 | 2.0390553 | 4.64E-06 | 1.16E-05 | Up |
| IGHD3-16 | 2.2208916 | 0.0002376 | 0.0004669 | Up |
| IGHD3-9 | 2.1788673 | 0.0011326 | 0.0020093 | Up |
| IGHD3-3 | 2.2103748 | 9.94E-05 | 0.0002055 | Up |
| IGHD2-2 | 2.1194371 | 0.0010862 | 0.0019306 | Up |
| IGHV1-18 | 2.1937344 | 7.20E-06 | 1.75E-05 | Up |
| IGHV3-21 | 2.0214776 | 1.70E-05 | 3.92E-05 | Up |
| IGHV1-24 | 2.0178967 | 4.27E-05 | 9.32E-05 | Up |
| IGHV3-33 | 2.3025345 | 2.30E-06 | 5.96E-06 | Up |
| IGHV3-38 | 2.2759038 | 6.25E-06 | 1.53E-05 | Up |
| IGHV1-45 | 3.137331 | 0.0001069 | 0.00022 | Up |
| IGHV3-49 | 2.0760195 | 2.21E-05 | 5.02E-05 | Up |
| IGHV5-51 | 2.1579793 | 3.21E-06 | 8.16E-06 | Up |
| IGHV3-73 | 2.649719 | 1.74E-07 | 5.23E-07 | Up |
| KRTAP29-1 | 3.4726898 | 1.11E-06 | 3.02E-06 | Up |
| KRTAP2-3 | 4.2824508 | 1.28E-05 | 3.02E-05 | Up |
| KRTAP3-1 | 4.5713813 | 1.77E-06 | 4.67E-06 | Up |
| KLK9 | 5.2611808 | 2.79E-06 | 7.16E-06 | Up |
| CGB8 | 4.9275812 | 1.17E-05 | 2.77E-05 | Up |
| TRIM59 | 2.5292592 | 1.87E-26 | 3.16E-25 | Up |
| MLLT11 | 2.7382298 | 9.72E-16 | 7.23E-15 | Up |
| MAGEA12 | 8.8526162 | 5.61E-09 | 2.02E-08 | Up |
| GPC2 | 3.5489829 | 2.17E-18 | 2.07E-17 | Up |
| LTB4R | 2.3699894 | 6.44E-11 | 2.91E-10 | Up |
| LTB4R2 | 2.149699 | 4.52E-11 | 2.08E-10 | Up |
| HBE1 | 2.6308183 | 0.0016831 | 0.0029155 | Up |
| SMCO1 | 2.3970616 | 6.45E-08 | 2.05E-07 | Up |
| ALG3 | 2.1763237 | 3.70E-26 | 6.16E-25 | Up |
| COLCA2 | 2.622493 | 1.62E-08 | 5.50E-08 | Up |
| FOXI3 | 7.9302526 | 5.10E-17 | 4.31E-16 | Up |
| EML6 | 2.0361095 | 1.80E-08 | 6.08E-08 | Up |
| CAPN14 | 4.3068256 | 1.08E-12 | 5.88E-12 | Up |
| FER1L6 | 2.9202292 | 7.94E-06 | 1.92E-05 | Up |
| RAD51AP2 | 4.1419519 | 2.05E-18 | 1.96E-17 | Up |
| UBE2QL1 | 3.5729766 | 1.47E-09 | 5.64E-09 | Up |
| KCNU1 | 4.4179794 | 2.94E-05 | 6.56E-05 | Up |
| HMX1 | 6.8283126 | 3.03E-08 | 9.99E-08 | Up |
| GCGR | 5.7050203 | 1.37E-11 | 6.65E-11 | Up |
| FAM72D | 3.2777245 | 4.04E-25 | 6.35E-24 | Up |
| TNFRSF25 | 2.2292327 | 1.60E-14 | 1.05E-13 | Up |
| RPTN | 8.9005017 | 9.55E-12 | 4.71E-11 | Up |
| SP9 | 7.7962737 | 4.02E-18 | 3.74E-17 | Up |
| PPP1R3G | 2.0350216 | 3.06E-10 | 1.27E-09 | Up |
| FANCG | 2.1184156 | 7.23E-21 | 8.38E-20 | Up |
| MAGEA3 | 10.121076 | 5.37E-14 | 3.36E-13 | Up |
| C10orf55 | 2.2809247 | 8.82E-11 | 3.92E-10 | Up |
| CKMT1A | 4.1304694 | 7.15E-22 | 9.01E-21 | Up |
| IGHV3-64 | 2.2932205 | 1.41E-05 | 3.29E-05 | Up |
| CERS1 | 2.0687159 | 0.0002028 | 0.0004022 | Up |
| IGKV3D-15 | 2.2006234 | 7.06E-06 | 1.72E-05 | Up |
| IGHV4-59 | 2.1451951 | 3.46E-06 | 8.78E-06 | Up |
| ZNF812P | 2.9819302 | 7.36E-07 | 2.04E-06 | Up |
| IGKV6D-21 | 2.2050098 | 0.0016098 | 0.0027969 | Up |
| ERVMER34-1 | 3.2558663 | 7.06E-15 | 4.80E-14 | Up |
| AKR1B15 | 8.5395545 | 1.70E-21 | 2.09E-20 | Up |
| IGKV3D-7 | 2.6278344 | 0.0001153 | 0.0002365 | Up |
| SPINK8 | 2.6123973 | 0.0001937 | 0.0003855 | Up |
| NKX1-2 | 8.9748383 | 6.05E-24 | 8.83E-23 | Up |
| PRAC2 | 6.9504753 | 3.89E-09 | 1.43E-08 | Up |
| ANKRD18B | 4.0858638 | 2.57E-16 | 2.01E-15 | Up |
| ANKRD63 | 3.7140437 | 4.27E-06 | 1.07E-05 | Up |
| IGHV4-31 | 2.5893745 | 5.93E-07 | 1.67E-06 | Up |
| DIRC3 | 2.941454 | 4.11E-09 | 1.50E-08 | Up |
| TMEM114 | 4.567624 | 1.56E-05 | 3.63E-05 | Up |
| PAGE2 | 9.6111746 | 1.68E-06 | 4.45E-06 | Up |
| MCIDAS | 2.1986524 | 1.82E-09 | 6.93E-09 | Up |
| SHISA8 | 3.1475692 | 1.12E-09 | 4.35E-09 | Up |
| CLEC2L | 6.1402736 | 8.33E-14 | 5.11E-13 | Up |
| TLX1NB | 5.721286 | 2.77E-07 | 8.09E-07 | Up |
| C4orf51 | 2.3866854 | 0.0001853 | 0.0003697 | Up |
| CKMT1B | 4.5642051 | 1.71E-24 | 2.58E-23 | Up |
| PRSS56 | 6.5113296 | 3.20E-06 | 8.14E-06 | Up |
| SHISA9 | 4.7240792 | 1.11E-06 | 2.99E-06 | Up |
| KIFC1 | 3.7271466 | 1.23E-57 | 9.15E-56 | Up |
| PAGE2B | 4.9729116 | 8.48E-05 | 0.0001774 | Up |
| IGKV2D-30 | 2.0218594 | 0.0040422 | 0.0066356 | Up |
| OR1J4 | 2.2991953 | 5.87E-06 | 1.45E-05 | Up |
| NME1 | 2.149657 | 5.46E-25 | 8.45E-24 | Up |
| IGKV1-6 | 2.4442904 | 1.78E-06 | 4.68E-06 | Up |
| IGKV1-37 | 2.6144025 | 2.61E-05 | 5.88E-05 | Up |
| IGKV1D-33 | 2.0637305 | 0.0016825 | 0.0029147 | Up |
| TEX35 | 2.3346401 | 2.05E-05 | 4.68E-05 | Up |
| UGT1A5 | 4.1158093 | 4.93E-08 | 1.58E-07 | Up |
| IGKV1-17 | 2.2939236 | 2.47E-06 | 6.37E-06 | Up |
| LCE1F | 7.4108247 | 6.49E-10 | 2.60E-09 | Up |
| IGKV1-16 | 2.0804703 | 3.32E-05 | 7.37E-05 | Up |
| MIF | 2.2122257 | 1.47E-20 | 1.66E-19 | Up |
| UGT1A9 | 5.202728 | 1.91E-07 | 5.71E-07 | Up |
| IGKV2-24 | 2.3427442 | 7.91E-06 | 1.91E-05 | Up |
| UGT1A1 | 4.4932128 | 1.07E-12 | 5.83E-12 | Up |
| TMEFF1 | 3.5579459 | 1.19E-09 | 4.63E-09 | Up |
| IGKV1-9 | 2.0189593 | 0.0001092 | 0.0002243 | Up |
| SPRR2A | 9.188346 | 1.08E-18 | 1.05E-17 | Up |
| IGKV1-33 | 2.4732231 | 0.0001549 | 0.0003123 | Up |
| UGT1A8 | 7.2040757 | 2.63E-07 | 7.72E-07 | Up |
| IGKV1-39 | 2.3775631 | 3.15E-05 | 7.01E-05 | Up |
| UGT1A10 | 7.9050577 | 7.81E-08 | 2.45E-07 | Up |
| IGKV2D-28 | 2.7997642 | 8.16E-05 | 0.0001709 | Up |
| CCDC169 | 3.0066823 | 1.09E-11 | 5.35E-11 | Up |
| STRC | 2.9832653 | 7.59E-10 | 3.02E-09 | Up |
| IGKV3-7 | 2.093099 | 1.58E-05 | 3.66E-05 | Up |
| UGT1A3 | 5.4627931 | 2.26E-14 | 1.46E-13 | Up |
| PSG4 | 8.2515161 | 7.90E-11 | 3.53E-10 | Up |
| EFNA4 | 2.085343 | 6.05E-29 | 1.19E-27 | Up |
| GSTA1 | 2.2775302 | 0.0006773 | 0.0012436 | Up |
| SPRR2F | 4.3943776 | 7.34E-07 | 2.04E-06 | Up |
| UGT1A7 | 10.153828 | 4.27E-15 | 2.97E-14 | Up |
| UGT1A4 | 4.4390403 | 2.68E-10 | 1.12E-09 | Up |
| ADH1C | 3.0341529 | 0.0002825 | 0.0005485 | Up |
| APELA | 2.5646888 | 4.30E-07 | 1.23E-06 | Up |
| PRR5-ARHGAP8 | 2.6118382 | 1.56E-14 | 1.02E-13 | Up |
| C4orf54 | 2.5552912 | 4.00E-05 | 8.75E-05 | Up |
| THEGL | 2.8385318 | 1.51E-08 | 5.17E-08 | Up |
| TMEM158 | 3.2616195 | 2.43E-17 | 2.12E-16 | Up |
| CCDC169-SOHLH2 | 2.0685052 | 0.0008578 | 0.0015477 | Up |
| IGKV2D-40 | 2.2274366 | 0.0001091 | 0.0002241 | Up |
| AL691442.1 | 2.6045084 | 8.65E-06 | 2.08E-05 | Up |
| FOXD1 | 4.4150898 | 4.69E-23 | 6.49E-22 | Up |
| IGKV1D-39 | 2.464072 | 0.0002497 | 0.0004892 | Up |
| HOXA10 | 5.3620824 | 7.57E-29 | 1.48E-27 | Up |
| ETV3L | 3.156271 | 8.73E-05 | 0.0001821 | Up |
| MEX3A | 4.05602 | 1.78E-23 | 2.53E-22 | Up |
| KRTAP5-9 | 2.385452 | 2.98E-05 | 6.64E-05 | Up |
| CCDC166 | 3.7535635 | 1.01E-05 | 2.42E-05 | Up |
| CARD17 | 3.4002802 | 1.35E-11 | 6.56E-11 | Up |
| OR5M11 | 3.7595908 | 3.86E-08 | 1.26E-07 | Up |
| CARD18 | 8.0990896 | 3.24E-13 | 1.87E-12 | Up |
| ASIC5 | 4.3351448 | 5.23E-09 | 1.89E-08 | Up |
| SALL3 | 7.2882393 | 1.24E-08 | 4.29E-08 | Up |
| KLRF2 | 3.0381343 | 0.0001214 | 0.0002482 | Up |
| CAPNS2 | 6.8943579 | 1.00E-20 | 1.15E-19 | Up |
| KHDC1L | 6.403639 | 2.65E-11 | 1.25E-10 | Up |
| TAS2R38 | 4.9954653 | 2.49E-12 | 1.31E-11 | Up |
| MGAM2 | 4.0368535 | 6.66E-06 | 1.63E-05 | Up |
| OR9A4 | 2.3236502 | 0.0008415 | 0.0015207 | Up |
| AL355102.2 | 2.6238727 | 6.87E-07 | 1.92E-06 | Up |
| OR11H7 | 2.678678 | 1.47E-09 | 5.67E-09 | Up |
| TUBB3 | 3.5803518 | 2.06E-16 | 1.63E-15 | Up |
| TMEM179 | 5.5996194 | 9.36E-09 | 3.28E-08 | Up |
| IGHV3OR15-7 | 2.2748152 | 2.10E-05 | 4.78E-05 | Up |
| LYPD8 | 5.0255973 | 1.18E-10 | 5.15E-10 | Up |
| TGFBR3L | 3.0315736 | 1.44E-11 | 6.97E-11 | Up |
| AC107871.1 | 2.8850646 | 1.54E-10 | 6.63E-10 | Up |
| HOXB7 | 2.5320115 | 6.28E-15 | 4.29E-14 | Up |
| FRRS1L | 3.6267713 | 2.21E-07 | 6.53E-07 | Up |
| KCNJ18 | 4.354007 | 1.21E-05 | 2.86E-05 | Up |
| AL035425.2 | 4.8473565 | 0.0001162 | 0.0002381 | Up |
| XKR7 | 2.2920967 | 0.0019762 | 0.003394 | Up |
| BOP1 | 2.2160699 | 6.69E-27 | 1.16E-25 | Up |
| TUBB8 | 2.0518312 | 1.59E-06 | 4.21E-06 | Up |
| TMEM249 | 3.4077084 | 1.68E-14 | 1.10E-13 | Up |
| TCF24 | 3.8983389 | 2.10E-11 | 1.00E-10 | Up |
| MIA | 3.7127915 | 5.68E-06 | 1.40E-05 | Up |
| GFY | 8.2023736 | 3.91E-10 | 1.60E-09 | Up |
| MMP12 | 6.377282 | 3.05E-31 | 6.89E-30 | Up |
| FAM72C | 3.4766579 | 1.46E-21 | 1.80E-20 | Up |
| RBP3 | 2.4872855 | 2.13E-05 | 4.85E-05 | Up |
| ZNF488 | 3.9826729 | 2.15E-20 | 2.39E-19 | Up |
| AC020922.1 | 5.0925395 | 1.43E-08 | 4.89E-08 | Up |
| EDDM13 | 2.829697 | 7.81E-10 | 3.10E-09 | Up |
| CCDC177 | 5.1288955 | 7.26E-11 | 3.26E-10 | Up |
| MAGEA9B | 9.2325843 | 3.09E-14 | 1.98E-13 | Up |
| GABRQ | 6.8113068 | 6.12E-25 | 9.42E-24 | Up |
| MAGEA2 | 4.8407811 | 3.19E-06 | 8.11E-06 | Up |
| CT45A1 | 4.4753906 | 0.0023792 | 0.0040359 | Up |
| ERVV-2 | 7.4014022 | 4.87E-06 | 1.21E-05 | Up |
| SPIB | 2.2814289 | 0.0002239 | 0.0004415 | Up |
| ERVV-1 | 4.3026783 | 3.45E-05 | 7.64E-05 | Up |
| CT45A10 | 3.8605209 | 0.0011729 | 0.0020759 | Up |
| AC011473.4 | 7.9191242 | 3.58E-10 | 1.47E-09 | Up |
| RNF225 | 3.1591801 | 7.79E-11 | 3.48E-10 | Up |
| MEI4 | 2.6389425 | 0.000644 | 0.0011855 | Up |
| AL121758.1 | 2.8602437 | 7.13E-09 | 2.53E-08 | Up |
| IGHV3OR16-12 | 2.028555 | 0.0002552 | 0.0004992 | Up |
| RPS10-NUDT3 | 2.3988553 | 2.57E-07 | 7.53E-07 | Up |
| RASL10B | 2.081858 | 4.99E-07 | 1.42E-06 | Up |
| SRXN1 | 2.8100262 | 9.22E-10 | 3.64E-09 | Up |
| AL603832.3 | 2.205882 | 7.97E-08 | 2.50E-07 | Up |
| IFNL4 | 4.2253068 | 1.90E-06 | 5.00E-06 | Up |
| AC115284.1 | 2.0951494 | 1.16E-06 | 3.12E-06 | Up |
| ZBTB8B | 2.3845161 | 3.54E-05 | 7.81E-05 | Up |
| TM4SF19-TCTEX1D2 | 2.0202691 | 1.83E-07 | 5.49E-07 | Up |
| HIST1H4K | 2.0421017 | 1.41E-08 | 4.83E-08 | Up |
| EPOP | 2.1507541 | 9.24E-14 | 5.64E-13 | Up |
| HIST1H2BM | 3.9849836 | 7.89E-11 | 3.52E-10 | Up |
| LHX1 | 5.7504101 | 3.61E-07 | 1.04E-06 | Up |
| HIST1H2BG | 3.7137007 | 1.76E-18 | 1.69E-17 | Up |
| IGKV2-40 | 2.4627235 | 0.0009391 | 0.0016853 | Up |
| HIST1H3G | 4.2951927 | 3.51E-17 | 3.02E-16 | Up |
| HIST1H3B | 4.4990776 | 1.69E-17 | 1.49E-16 | Up |
| ADRA2B | 2.7067312 | 6.03E-08 | 1.92E-07 | Up |
| HIST1H2BE | 3.065734 | 4.38E-11 | 2.01E-10 | Up |
| HIST1H2BO | 4.5226965 | 2.33E-21 | 2.82E-20 | Up |
| HIST1H2AH | 3.2049528 | 8.79E-12 | 4.35E-11 | Up |
| ZNF280B | 2.3851768 | 1.33E-08 | 4.60E-08 | Up |
| AC091980.2 | 3.6010942 | 2.96E-05 | 6.61E-05 | Up |
| HIST1H3I | 2.9383315 | 6.17E-06 | 1.52E-05 | Up |
| AL449403.2 | 3.1277978 | 0.0046877 | 0.0076247 | Up |
| HIST1H2BH | 5.6652978 | 5.20E-23 | 7.15E-22 | Up |
| HIST1H3A | 2.4935589 | 4.95E-07 | 1.40E-06 | Up |
| UHRF1 | 4.066902 | 1.65E-51 | 9.25E-50 | Up |
| HIST1H2AJ | 4.2822579 | 2.21E-13 | 1.29E-12 | Up |
| HIST1H2BB | 3.3196339 | 7.82E-09 | 2.77E-08 | Up |
| HIST1H2AL | 3.0678909 | 3.61E-10 | 1.49E-09 | Up |
| HIST1H4E | 3.0592428 | 7.91E-16 | 5.92E-15 | Up |
| HIST1H2AE | 3.8555478 | 2.99E-18 | 2.80E-17 | Up |
| HIST1H4D | 2.4262723 | 9.80E-11 | 4.33E-10 | Up |
| HIST1H2BF | 3.9361072 | 3.56E-16 | 2.74E-15 | Up |
| NEFL | 5.2987671 | 1.41E-09 | 5.42E-09 | Up |
| HIST1H3F | 5.0189841 | 1.07E-16 | 8.71E-16 | Up |
| RDM1 | 3.9727956 | 5.25E-28 | 9.77E-27 | Up |
| AC135068.2 | 2.0379407 | 3.93E-05 | 8.61E-05 | Up |
| HIST1H3C | 4.1908473 | 1.37E-12 | 7.38E-12 | Up |
| HIST1H2AB | 3.2938786 | 2.03E-10 | 8.61E-10 | Up |
| HIST1H2BI | 3.0866427 | 8.16E-09 | 2.89E-08 | Up |
| HIST1H2AM | 3.5959623 | 3.02E-16 | 2.35E-15 | Up |
| HIST1H4B | 2.5128602 | 6.48E-10 | 2.60E-09 | Up |
| HIST1H3H | 2.5668452 | 2.96E-12 | 1.55E-11 | Up |
| AL031602.1 | 4.1225821 | 5.67E-13 | 3.18E-12 | Up |
| AC244230.1 | 2.4097762 | 8.97E-06 | 2.15E-05 | Up |
| AL357140.3 | 2.739499 | 1.20E-06 | 3.24E-06 | Up |
| IGHV1-69D | 2.1712959 | 1.26E-05 | 2.98E-05 | Up |
| AC134981.1 | 2.140833 | 0.0010233 | 0.0018255 | Up |
| AC135068.8 | 2.4133679 | 0.0003769 | 0.0007181 | Up |

**Supplementary table 4: Differentially expressed lncRNAs in non-smoking LUSC**

| **lncRNA** | **logFC** | **p-value** | **FDR** | **regulate** |
| --- | --- | --- | --- | --- |
| C10orf95 | -2.285508 | 8.09E-42 | 5.59E-40 | Down |
| KIAA0087 | -2.356023 | 5.68E-13 | 4.56E-12 | Down |
| LINC01558 | -2.016542 | 1.40E-18 | 1.94E-17 | Down |
| MIR1-1HG-AS1 | -2.11739 | 1.22E-13 | 1.07E-12 | Down |
| FO393415.1 | -2.382995 | 7.71E-20 | 1.20E-18 | Down |
| LINC00982 | -2.768912 | 9.51E-33 | 3.72E-31 | Down |
| LINC00469 | -2.46772 | 1.24E-13 | 1.08E-12 | Down |
| AC011944.1 | -2.707382 | 1.62E-25 | 4.02E-24 | Down |
| AC105206.1 | -4.601863 | 2.36E-72 | 5.41E-70 | Down |
| C5orf64 | -2.823831 | 3.07E-41 | 2.02E-39 | Down |
| AC004832.1 | -3.227982 | 2.75E-22 | 5.30E-21 | Down |
| LINC00315 | -2.090405 | 1.49E-32 | 5.77E-31 | Down |
| UMODL1-AS1 | -3.085785 | 1.00E-18 | 1.41E-17 | Down |
| MIR22HG | -2.076903 | 3.03E-46 | 2.51E-44 | Down |
| AL354714.1 | -3.003245 | 4.94E-21 | 8.41E-20 | Down |
| MIR29B2CHG | -2.156564 | 4.00E-26 | 1.04E-24 | Down |
| ADAMTSL4-AS1 | -2.373008 | 9.52E-48 | 8.49E-46 | Down |
| ACOXL-AS1 | -2.627154 | 1.78E-54 | 2.23E-52 | Down |
| AC010624.1 | -2.177301 | 3.98E-16 | 4.44E-15 | Down |
| AC092384.1 | -2.435763 | 3.03E-34 | 1.32E-32 | Down |
| LINC00671 | -2.772975 | 8.90E-43 | 6.32E-41 | Down |
| AC116407.1 | -4.240138 | 9.08E-115 | 2.43E-111 | Down |
| AC004540.1 | -2.051925 | 2.58E-25 | 6.38E-24 | Down |
| LMO7DN-IT1 | -2.039697 | 1.79E-12 | 1.36E-11 | Down |
| LINC02471 | -3.950032 | 4.81E-33 | 1.93E-31 | Down |
| AL133264.2 | -2.149892 | 7.83E-17 | 9.38E-16 | Down |
| AL606469.1 | -5.483664 | 2.18E-98 | 1.75E-95 | Down |
| AL031599.1 | -2.89962 | 3.50E-32 | 1.32E-30 | Down |
| AL355388.1 | -4.174312 | 2.08E-59 | 3.15E-57 | Down |
| AL161785.1 | -2.365659 | 9.09E-29 | 2.83E-27 | Down |
| SMIM25 | -3.022923 | 7.33E-56 | 9.80E-54 | Down |
| AC007182.1 | -2.095992 | 1.28E-08 | 5.70E-08 | Down |
| PGM5-AS1 | -4.057106 | 1.77E-59 | 2.73E-57 | Down |
| LINC01645 | -3.711398 | 2.01E-43 | 1.48E-41 | Down |
| AL591178.1 | -3.497637 | 4.17E-22 | 7.90E-21 | Down |
| LHFPL3-AS2 | -4.125919 | 8.30E-51 | 9.00E-49 | Down |
| AC079630.1 | -3.968246 | 3.33E-69 | 6.86E-67 | Down |
| AC078942.1 | -3.100719 | 2.75E-26 | 7.29E-25 | Down |
| SFTA1P | -4.808354 | 1.48E-81 | 4.96E-79 | Down |
| LINC01671 | -2.259143 | 3.55E-17 | 4.42E-16 | Down |
| ATP13A4-AS1 | -3.184468 | 2.69E-16 | 3.07E-15 | Down |
| TTTY16 | -2.659894 | 1.02E-11 | 6.92E-11 | Down |
| CADM3-AS1 | -3.31068 | 4.94E-34 | 2.11E-32 | Down |
| PACRG-AS3 | -4.555018 | 1.93E-58 | 2.76E-56 | Down |
| AC004540.2 | -2.644691 | 6.74E-25 | 1.61E-23 | Down |
| LINC00694 | -3.791841 | 1.51E-72 | 3.56E-70 | Down |
| AL731557.1 | -3.040228 | 4.01E-34 | 1.74E-32 | Down |
| AL109741.1 | -3.315922 | 7.09E-72 | 1.58E-69 | Down |
| AC092802.1 | -2.071301 | 6.32E-25 | 1.52E-23 | Down |
| AL035530.1 | -2.415225 | 3.49E-18 | 4.75E-17 | Down |
| AL135960.1 | -3.215876 | 2.95E-46 | 2.47E-44 | Down |
| AL008733.1 | -3.669795 | 7.63E-30 | 2.54E-28 | Down |
| LINC01108 | -3.294285 | 4.56E-25 | 1.10E-23 | Down |
| BX248123.1 | -2.846082 | 4.43E-35 | 2.09E-33 | Down |
| LINC00840 | -2.698267 | 5.39E-24 | 1.20E-22 | Down |
| AL390036.1 | -2.851708 | 1.80E-35 | 8.67E-34 | Down |
| AC135178.1 | -2.73607 | 9.45E-30 | 3.13E-28 | Down |
| AL451069.1 | -2.40387 | 1.30E-13 | 1.13E-12 | Down |
| C14orf132 | -2.528073 | 4.25E-38 | 2.49E-36 | Down |
| MGAT3-AS1 | -2.170256 | 1.10E-10 | 6.48E-10 | Down |
| AC010998.1 | -3.974714 | 1.16E-44 | 9.10E-43 | Down |
| LINC01624 | -2.23197 | 1.76E-15 | 1.85E-14 | Down |
| AC018647.1 | -3.733244 | 1.03E-74 | 2.65E-72 | Down |
| TARID | -2.297337 | 8.81E-15 | 8.66E-14 | Down |
| AC003991.1 | -3.700111 | 6.60E-50 | 6.79E-48 | Down |
| AC008440.1 | -2.228044 | 2.84E-24 | 6.46E-23 | Down |
| AL590226.1 | -4.411403 | 2.10E-90 | 9.37E-88 | Down |
| LINC01013 | -2.417419 | 3.78E-19 | 5.53E-18 | Down |
| AL033519.3 | -3.93119 | 3.88E-50 | 4.05E-48 | Down |
| AL445307.1 | -4.381913 | 7.08E-95 | 4.37E-92 | Down |
| SRGAP3-AS2 | -3.530768 | 1.25E-17 | 1.63E-16 | Down |
| LINC01165 | -2.891715 | 3.50E-24 | 7.90E-23 | Down |
| MED4-AS1 | -2.394693 | 4.45E-55 | 5.76E-53 | Down |
| LINC02038 | -3.12552 | 3.85E-33 | 1.56E-31 | Down |
| AL662860.1 | -2.061123 | 3.04E-11 | 1.91E-10 | Down |
| AC243836.1 | -2.026221 | 1.36E-11 | 8.97E-11 | Down |
| AC105053.1 | -3.932215 | 4.63E-48 | 4.17E-46 | Down |
| LINC01150 | -2.07598 | 2.41E-19 | 3.59E-18 | Down |
| LINC01747 | -2.0042 | 1.80E-14 | 1.72E-13 | Down |
| AL035409.1 | -2.621415 | 1.12E-35 | 5.59E-34 | Down |
| AL158071.3 | -2.037262 | 1.49E-26 | 4.01E-25 | Down |
| AL391807.1 | -2.208756 | 1.01E-11 | 6.80E-11 | Down |
| HHATL-AS1 | -3.131721 | 3.16E-16 | 3.56E-15 | Down |
| AC013275.1 | -2.565209 | 8.13E-15 | 8.03E-14 | Down |
| AC096637.2 | -2.449521 | 9.19E-17 | 1.09E-15 | Down |
| AC002451.1 | -2.828647 | 2.64E-28 | 7.86E-27 | Down |
| AL445426.1 | -2.417802 | 1.14E-28 | 3.51E-27 | Down |
| AC013264.1 | -3.128646 | 1.14E-22 | 2.26E-21 | Down |
| AP003774.3 | -2.042031 | 7.89E-18 | 1.04E-16 | Down |
| AL592114.3 | -3.162531 | 9.05E-29 | 2.83E-27 | Down |
| AP002856.2 | -5.476299 | 5.53E-41 | 3.58E-39 | Down |
| AL451069.2 | -2.380196 | 1.55E-21 | 2.76E-20 | Down |
| AL353747.3 | -2.992266 | 3.83E-10 | 2.11E-09 | Down |
| AL035701.1 | -2.105237 | 3.53E-17 | 4.39E-16 | Down |
| PGM5P4-AS1 | -3.376463 | 3.45E-35 | 1.64E-33 | Down |
| AC011899.1 | -3.613719 | 4.82E-21 | 8.24E-20 | Down |
| LINC01105 | -3.955547 | 1.97E-30 | 6.74E-29 | Down |
| AC093390.1 | -2.528115 | 2.22E-09 | 1.11E-08 | Down |
| LINC01031 | -2.151479 | 2.09E-19 | 3.12E-18 | Down |
| AL354714.3 | -4.08705 | 2.42E-38 | 1.44E-36 | Down |
| KIF26B-AS1 | -2.589769 | 2.24E-17 | 2.85E-16 | Down |
| VIPR1-AS1 | -2.187212 | 3.94E-29 | 1.27E-27 | Down |
| AC016910.1 | -2.321009 | 3.87E-26 | 1.01E-24 | Down |
| ELN-AS1 | -3.08194 | 9.15E-32 | 3.39E-30 | Down |
| LINC01412 | -2.898775 | 1.48E-32 | 5.76E-31 | Down |
| AL355304.1 | -2.432098 | 5.11E-36 | 2.63E-34 | Down |
| AC092809.2 | -2.790976 | 2.33E-32 | 8.90E-31 | Down |
| LINC01166 | -2.984743 | 5.80E-15 | 5.81E-14 | Down |
| CYP1B1-AS1 | -2.014571 | 4.79E-29 | 1.53E-27 | Down |
| AC011899.2 | -3.043658 | 2.71E-54 | 3.34E-52 | Down |
| LINC00892 | -2.107394 | 1.01E-17 | 1.32E-16 | Down |
| LINC00702 | -3.137449 | 5.87E-53 | 6.73E-51 | Down |
| AC007743.1 | -2.137068 | 5.42E-17 | 6.64E-16 | Down |
| TLR8-AS1 | -2.799493 | 1.05E-18 | 1.49E-17 | Down |
| HCG21 | -2.011797 | 1.50E-19 | 2.26E-18 | Down |
| LINC01765 | -3.473121 | 1.33E-17 | 1.73E-16 | Down |
| LINC00656 | -3.026566 | 8.47E-28 | 2.47E-26 | Down |
| AC004947.1 | -4.318623 | 4.87E-63 | 8.50E-61 | Down |
| AL157895.1 | -2.372431 | 9.55E-26 | 2.43E-24 | Down |
| AC006042.4 | -2.158086 | 1.90E-26 | 5.07E-25 | Down |
| AC243772.3 | -2.382599 | 4.27E-23 | 8.83E-22 | Down |
| LANCL1-AS1 | -4.109214 | 4.93E-163 | 3.95E-159 | Down |
| AC123595.1 | -2.570368 | 1.67E-37 | 9.36E-36 | Down |
| AC007405.1 | -2.629909 | 9.07E-27 | 2.47E-25 | Down |
| MAGI2-AS3 | -2.258992 | 2.54E-36 | 1.32E-34 | Down |
| LINC01506 | -2.374538 | 8.03E-17 | 9.59E-16 | Down |
| AL445470.1 | -3.564545 | 1.30E-53 | 1.56E-51 | Down |
| LINC00163 | -3.714075 | 1.43E-35 | 6.99E-34 | Down |
| LINC01827 | -2.581004 | 7.17E-11 | 4.31E-10 | Down |
| AL365199.1 | -3.042016 | 9.60E-32 | 3.53E-30 | Down |
| AL133415.1 | -2.082855 | 5.68E-24 | 1.26E-22 | Down |
| SPAAR | -3.370978 | 7.07E-105 | 9.45E-102 | Down |
| AC006159.1 | -2.758244 | 1.23E-24 | 2.86E-23 | Down |
| AGAP1-IT1 | -2.006245 | 2.25E-20 | 3.70E-19 | Down |
| AC008268.1 | -6.314569 | 1.70E-48 | 1.60E-46 | Down |
| LINC00607 | -2.868188 | 2.68E-50 | 2.83E-48 | Down |
| LINC01936 | -3.365476 | 1.92E-48 | 1.80E-46 | Down |
| AL161618.1 | -3.276896 | 3.36E-13 | 2.80E-12 | Down |
| MYO16-AS1 | -3.183553 | 1.32E-23 | 2.82E-22 | Down |
| AP001189.1 | -4.068117 | 1.23E-101 | 1.14E-98 | Down |
| AC091806.1 | -2.769365 | 5.03E-29 | 1.60E-27 | Down |
| AC123023.1 | -2.824045 | 3.03E-11 | 1.91E-10 | Down |
| AC007278.2 | -2.333101 | 1.02E-13 | 9.00E-13 | Down |
| STARD13-AS | -3.056742 | 1.52E-38 | 9.18E-37 | Down |
| AL138900.2 | -3.552318 | 5.26E-20 | 8.32E-19 | Down |
| LINC01732 | -2.350421 | 7.26E-14 | 6.48E-13 | Down |
| AL445489.1 | -2.594877 | 1.53E-11 | 1.00E-10 | Down |
| AC096531.2 | -4.120918 | 4.20E-33 | 1.69E-31 | Down |
| AL157714.2 | -2.433835 | 5.17E-12 | 3.63E-11 | Down |
| AL356356.1 | -2.03514 | 1.66E-26 | 4.43E-25 | Down |
| LINC00211 | -2.860595 | 3.42E-25 | 8.40E-24 | Down |
| AC002066.1 | -2.590865 | 5.46E-27 | 1.52E-25 | Down |
| NFIA-AS2 | -2.105528 | 3.10E-14 | 2.90E-13 | Down |
| AC093110.1 | -3.729077 | 2.56E-158 | 1.03E-154 | Down |
| ZEB2-AS1 | -2.150029 | 2.40E-33 | 9.88E-32 | Down |
| LINC01352 | -2.955582 | 4.54E-47 | 4.00E-45 | Down |
| LINC01625 | -2.078586 | 1.14E-11 | 7.66E-11 | Down |
| AC116366.2 | -2.129547 | 2.76E-20 | 4.50E-19 | Down |
| AC092691.1 | -3.914344 | 2.06E-21 | 3.62E-20 | Down |
| LINC01168 | -2.616629 | 5.21E-12 | 3.65E-11 | Down |
| ADAMTS9-AS1 | -3.957513 | 3.64E-81 | 1.17E-78 | Down |
| ADAMTS9-AS2 | -3.649535 | 1.62E-93 | 9.26E-91 | Down |
| AC119424.1 | -4.442416 | 5.52E-52 | 6.15E-50 | Down |
| PARAL1 | -3.265169 | 1.00E-19 | 1.53E-18 | Down |
| AL512328.1 | -2.149003 | 6.91E-16 | 7.47E-15 | Down |
| LINC02016 | -6.516508 | 4.94E-93 | 2.48E-90 | Down |
| AC087521.1 | -4.352609 | 7.54E-106 | 1.21E-102 | Down |
| AC096564.1 | -2.763882 | 7.00E-46 | 5.73E-44 | Down |
| LINC01513 | -2.6355 | 4.83E-14 | 4.40E-13 | Down |
| LINC00968 | -4.422707 | 5.53E-96 | 3.70E-93 | Down |
| AC010255.1 | -2.200184 | 2.98E-13 | 2.49E-12 | Down |
| MEF2C-AS1 | -2.452618 | 2.24E-31 | 8.10E-30 | Down |
| AL645924.1 | -2.596807 | 5.21E-16 | 5.75E-15 | Down |
| LINC01197 | -2.771809 | 2.63E-49 | 2.58E-47 | Down |
| AC093772.1 | -3.340711 | 1.17E-30 | 4.05E-29 | Down |
| AC002070.1 | -2.046081 | 2.84E-34 | 1.24E-32 | Down |
| C8orf34-AS1 | -3.357092 | 1.12E-25 | 2.84E-24 | Down |
| HHIP-AS1 | -2.776398 | 3.82E-31 | 1.37E-29 | Down |
| AC114291.1 | -4.045529 | 1.09E-24 | 2.54E-23 | Down |
| AC105389.2 | -2.386673 | 9.57E-20 | 1.47E-18 | Down |
| LINC02265 | -2.554651 | 1.28E-21 | 2.31E-20 | Down |
| AC112206.2 | -3.616086 | 1.21E-89 | 4.86E-87 | Down |
| AC092834.1 | -2.805947 | 2.20E-17 | 2.81E-16 | Down |
| AC010255.2 | -2.362032 | 1.05E-14 | 1.02E-13 | Down |
| AC113349.1 | -3.149497 | 7.85E-13 | 6.21E-12 | Down |
| CARMN | -2.081696 | 1.12E-35 | 5.59E-34 | Down |
| AC105384.1 | -2.687251 | 3.65E-33 | 1.49E-31 | Down |
| LINC02147 | -2.696151 | 1.46E-20 | 2.42E-19 | Down |
| ARHGEF38-IT1 | -2.055097 | 8.93E-13 | 7.00E-12 | Down |
| AC007663.2 | -2.187985 | 1.92E-13 | 1.64E-12 | Down |
| AP000866.2 | -3.271763 | 3.17E-107 | 6.36E-104 | Down |
| LINC01612 | -2.94318 | 9.47E-16 | 1.02E-14 | Down |
| AC022101.1 | -2.043561 | 1.23E-11 | 8.21E-11 | Down |
| AC112722.1 | -3.194847 | 8.36E-71 | 1.81E-68 | Down |
| AC004066.2 | -2.113293 | 5.36E-12 | 3.74E-11 | Down |
| AC109361.2 | -2.209675 | 3.33E-19 | 4.91E-18 | Down |
| AC079467.1 | -4.552842 | 1.84E-44 | 1.43E-42 | Down |
| AC037459.2 | -2.249487 | 2.16E-82 | 7.53E-80 | Down |
| F11-AS1 | -2.070941 | 3.60E-08 | 1.51E-07 | Down |
| MIR3945HG | -3.938662 | 5.70E-64 | 1.06E-61 | Down |
| LINC01099 | -2.191652 | 3.23E-17 | 4.06E-16 | Down |
| AC091849.2 | -2.837337 | 4.33E-22 | 8.17E-21 | Down |
| AP003548.1 | -2.69176 | 2.05E-21 | 3.61E-20 | Down |
| AC104211.2 | -4.469359 | 2.66E-77 | 7.63E-75 | Down |
| AC046195.1 | -3.165654 | 5.70E-17 | 6.94E-16 | Down |
| AC022217.2 | -2.850778 | 9.00E-29 | 2.82E-27 | Down |
| LINC01942 | -2.316594 | 4.85E-14 | 4.42E-13 | Down |
| NKX2-1-AS1 | -2.713394 | 5.03E-15 | 5.11E-14 | Down |
| AC090796.1 | -3.87053 | 1.64E-32 | 6.31E-31 | Down |
| LINC01863 | -5.391704 | 3.81E-91 | 1.80E-88 | Down |
| RBPMS-AS1 | -3.050965 | 1.41E-59 | 2.21E-57 | Down |
| AC012349.1 | -2.172935 | 5.08E-09 | 2.40E-08 | Down |
| AP001972.1 | -3.521605 | 7.43E-60 | 1.19E-57 | Down |
| NAV2-AS2 | -3.179423 | 9.25E-36 | 4.73E-34 | Down |
| AP001189.3 | -3.408599 | 4.65E-65 | 8.89E-63 | Down |
| AC009806.1 | -2.771736 | 2.61E-44 | 2.01E-42 | Down |
| AC044810.2 | -2.111292 | 2.02E-16 | 2.33E-15 | Down |
| LINC02489 | -4.149621 | 2.46E-45 | 1.97E-43 | Down |
| AC090559.1 | -2.408746 | 1.43E-34 | 6.43E-33 | Down |
| KC877392.1 | -2.149576 | 1.42E-14 | 1.36E-13 | Down |
| AP001189.5 | -2.877317 | 6.77E-31 | 2.37E-29 | Down |
| TBX5-AS1 | -2.712509 | 8.86E-42 | 6.08E-40 | Down |
| AP002954.1 | -2.359325 | 2.71E-20 | 4.42E-19 | Down |
| AP003064.2 | -3.063964 | 7.09E-24 | 1.55E-22 | Down |
| AP001528.2 | -2.504177 | 1.16E-34 | 5.27E-33 | Down |
| AP001189.6 | -2.130547 | 2.11E-16 | 2.43E-15 | Down |
| AC010185.1 | -2.065128 | 5.39E-08 | 2.20E-07 | Down |
| RMST | -3.401624 | 6.39E-17 | 7.69E-16 | Down |
| AC007406.3 | -2.270747 | 2.52E-36 | 1.31E-34 | Down |
| AC084880.3 | -3.266984 | 6.70E-22 | 1.24E-20 | Down |
| AC026369.2 | -2.209296 | 1.70E-43 | 1.27E-41 | Down |
| AC026369.3 | -4.326101 | 3.17E-62 | 5.41E-60 | Down |
| AC007207.2 | -2.286426 | 5.12E-09 | 2.41E-08 | Down |
| AP000438.1 | -3.08223 | 1.60E-34 | 7.15E-33 | Down |
| AP002761.3 | -2.377585 | 1.05E-37 | 6.00E-36 | Down |
| C11orf97 | -3.254577 | 5.94E-13 | 4.75E-12 | Down |
| AC084398.2 | -3.513047 | 1.85E-43 | 1.38E-41 | Down |
| AC027288.1 | -3.553958 | 2.70E-27 | 7.65E-26 | Down |
| AL132857.1 | -3.886311 | 5.13E-22 | 9.62E-21 | Down |
| AC023509.2 | -2.741296 | 2.28E-41 | 1.52E-39 | Down |
| HLX-AS1 | -2.910533 | 5.01E-34 | 2.13E-32 | Down |
| LINC02356 | -2.076761 | 5.88E-22 | 1.09E-20 | Down |
| AC124312.2 | -2.005031 | 3.41E-17 | 4.27E-16 | Down |
| AC027288.3 | -4.039292 | 4.73E-93 | 2.48E-90 | Down |
| AC024257.1 | -3.735153 | 1.08E-50 | 1.16E-48 | Down |
| AC090001.1 | -2.702694 | 1.17E-17 | 1.53E-16 | Down |
| FAM181A-AS1 | -2.115216 | 1.41E-08 | 6.22E-08 | Down |
| LINC00930 | -2.992098 | 1.06E-22 | 2.11E-21 | Down |
| AC091544.2 | -2.521997 | 2.37E-16 | 2.71E-15 | Down |
| AL357093.1 | -2.039461 | 1.50E-09 | 7.59E-09 | Down |
| AL357093.2 | -2.533926 | 1.12E-12 | 8.68E-12 | Down |
| LINC02289 | -2.434608 | 1.95E-26 | 5.19E-25 | Down |
| AL162511.1 | -4.55558 | 1.54E-63 | 2.74E-61 | Down |
| AC106028.2 | -2.185302 | 1.69E-23 | 3.57E-22 | Down |
| AL049871.1 | -2.234558 | 1.17E-28 | 3.59E-27 | Down |
| LINC02285 | -2.00236 | 5.91E-23 | 1.21E-21 | Down |
| AC005479.1 | -2.056783 | 1.34E-27 | 3.80E-26 | Down |
| AC013457.1 | -4.827495 | 2.12E-33 | 8.76E-32 | Down |
| PTCSC3 | -2.96834 | 1.08E-25 | 2.74E-24 | Down |
| LINC00924 | -2.61388 | 2.62E-29 | 8.56E-28 | Down |
| AC015914.1 | -2.614298 | 1.93E-32 | 7.40E-31 | Down |
| AC012409.1 | -2.144286 | 2.86E-11 | 1.81E-10 | Down |
| CTXND1 | -4.384698 | 7.20E-97 | 5.25E-94 | Down |
| AC087286.4 | -2.043921 | 8.94E-19 | 1.27E-17 | Down |
| AC026992.1 | -2.942586 | 9.78E-35 | 4.49E-33 | Down |
| AL132801.1 | -2.161384 | 2.13E-11 | 1.38E-10 | Down |
| AC106738.2 | -2.250209 | 2.43E-10 | 1.37E-09 | Down |
| AL365361.1 | -2.206363 | 1.61E-19 | 2.42E-18 | Down |
| LINC02126 | -2.709311 | 8.30E-25 | 1.97E-23 | Down |
| AC130456.1 | -2.878532 | 3.06E-21 | 5.28E-20 | Down |
| AC025259.3 | -2.293048 | 1.68E-22 | 3.30E-21 | Down |
| AC130456.2 | -3.398098 | 6.61E-31 | 2.33E-29 | Down |
| LINC00261 | -3.812714 | 7.05E-27 | 1.94E-25 | Down |
| AC104938.1 | -2.034534 | 1.91E-38 | 1.14E-36 | Down |
| LINC01571 | -3.061705 | 1.65E-10 | 9.54E-10 | Down |
| AC020763.1 | -2.2569 | 1.66E-17 | 2.14E-16 | Down |
| AC120498.2 | -2.181642 | 2.16E-11 | 1.39E-10 | Down |
| AC027277.2 | -2.74741 | 7.56E-35 | 3.49E-33 | Down |
| AC091544.4 | -2.105286 | 1.92E-12 | 1.44E-11 | Down |
| AC068700.1 | -2.008354 | 2.61E-17 | 3.29E-16 | Down |
| AL133355.1 | -2.435034 | 9.06E-80 | 2.80E-77 | Down |
| LINC01290 | -2.408354 | 2.49E-46 | 2.12E-44 | Down |
| AC130456.3 | -2.582356 | 4.58E-22 | 8.62E-21 | Down |
| AC008669.1 | -2.036677 | 7.14E-38 | 4.09E-36 | Down |
| AC022164.1 | -2.50738 | 5.72E-24 | 1.26E-22 | Down |
| AC026471.3 | -2.169962 | 5.98E-18 | 8.01E-17 | Down |
| SERTM2 | -3.178467 | 7.77E-21 | 1.31E-19 | Down |
| AC093510.2 | -2.419317 | 8.05E-28 | 2.37E-26 | Down |
| LINC02555 | -4.281644 | 3.73E-53 | 4.33E-51 | Down |
| AC003985.2 | -2.874115 | 4.74E-10 | 2.58E-09 | Down |
| AL133297.2 | -2.066814 | 5.53E-24 | 1.23E-22 | Down |
| AL008628.1 | -2.172 | 8.62E-20 | 1.33E-18 | Down |
| AC093278.2 | -2.508179 | 5.09E-52 | 5.75E-50 | Down |
| AC096921.2 | -3.048565 | 8.28E-83 | 3.02E-80 | Down |
| AC083837.1 | -3.171781 | 1.46E-49 | 1.45E-47 | Down |
| AC026992.2 | -3.662578 | 4.76E-56 | 6.48E-54 | Down |
| TTC39A-AS1 | -2.40414 | 8.80E-25 | 2.07E-23 | Down |
| LINC00165 | -2.860448 | 7.99E-11 | 4.78E-10 | Down |
| LINC01996 | -5.737874 | 7.89E-83 | 3.02E-80 | Down |
| LINC02185 | -3.054774 | 7.31E-25 | 1.75E-23 | Down |
| AC087392.2 | -2.943285 | 4.86E-29 | 1.55E-27 | Down |
| AC005736.1 | -2.658868 | 1.27E-37 | 7.18E-36 | Down |
| AC129507.2 | -3.064419 | 5.67E-29 | 1.79E-27 | Down |
| AC108134.3 | -2.11583 | 1.07E-28 | 3.30E-27 | Down |
| AC129507.3 | -2.759248 | 6.57E-26 | 1.68E-24 | Down |
| AC127521.1 | -2.939446 | 9.86E-50 | 9.89E-48 | Down |
| AC027281.1 | -3.227611 | 1.27E-13 | 1.11E-12 | Down |
| AC115099.1 | -2.06449 | 2.11E-14 | 1.99E-13 | Down |
| HID1-AS1 | -4.265984 | 9.87E-102 | 1.13E-98 | Down |
| AC091588.1 | -2.420238 | 5.78E-20 | 9.10E-19 | Down |
| AC015908.3 | -2.040999 | 7.37E-25 | 1.75E-23 | Down |
| AC104984.1 | -3.364956 | 1.47E-38 | 8.93E-37 | Down |
| NPY4R2 | -2.008447 | 1.32E-09 | 6.73E-09 | Down |
| AP000894.2 | -2.045182 | 2.22E-09 | 1.11E-08 | Down |
| LINC01908 | -2.239184 | 8.11E-08 | 3.20E-07 | Down |
| AC090844.3 | -2.803739 | 2.19E-22 | 4.24E-21 | Down |
| GATA6-AS1 | -3.274968 | 6.38E-64 | 1.16E-61 | Down |
| AC104984.4 | -4.493352 | 7.19E-90 | 3.04E-87 | Down |
| AP001094.3 | -2.410183 | 1.36E-10 | 7.96E-10 | Down |
| AC091588.3 | -3.250866 | 4.95E-59 | 7.22E-57 | Down |
| AC025048.1 | -2.583837 | 9.33E-22 | 1.69E-20 | Down |
| SLC14A2-AS1 | -3.0221 | 6.19E-33 | 2.47E-31 | Down |
| PCAT19 | -3.087962 | 1.03E-70 | 2.18E-68 | Down |
| AC005884.1 | -3.763776 | 7.67E-67 | 1.54E-64 | Down |
| AC011444.2 | -3.253904 | 5.20E-31 | 1.85E-29 | Down |
| AC002398.2 | -3.542156 | 1.06E-43 | 7.99E-42 | Down |
| AC005180.1 | -3.005408 | 2.52E-46 | 2.12E-44 | Down |
| AC003070.2 | -2.018815 | 1.10E-13 | 9.64E-13 | Down |
| AC079210.1 | -2.54294 | 1.16E-31 | 4.21E-30 | Down |
| AC005180.2 | -2.691835 | 1.11E-41 | 7.51E-40 | Down |
| LINC01836 | -2.702814 | 9.55E-36 | 4.82E-34 | Down |
| MIR497HG | -2.107726 | 4.19E-49 | 4.00E-47 | Down |
| AC007998.3 | -2.60989 | 4.38E-44 | 3.35E-42 | Down |
| AC011511.5 | -4.572823 | 1.28E-101 | 1.14E-98 | Down |
| AC005856.1 | -2.999592 | 9.05E-75 | 2.42E-72 | Down |
| LDLRAD4-AS1 | -3.907192 | 2.73E-59 | 4.06E-57 | Down |
| AC093567.1 | -2.338219 | 3.09E-24 | 7.01E-23 | Down |
| AC010776.2 | -3.888581 | 7.90E-23 | 1.58E-21 | Down |
| AC243960.3 | -2.802859 | 2.91E-43 | 2.11E-41 | Down |
| FENDRR | -4.106527 | 8.12E-73 | 1.97E-70 | Down |
| AC010329.1 | -2.747795 | 1.90E-15 | 2.00E-14 | Down |
| AC135012.3 | -5.149949 | 1.66E-73 | 4.17E-71 | Down |
| AC245128.3 | -2.310241 | 4.00E-22 | 7.61E-21 | Down |
| AL354861.3 | -3.300437 | 4.43E-23 | 9.13E-22 | Down |
| AL009178.2 | -2.400275 | 1.01E-27 | 2.94E-26 | Down |
| LINC01082 | -4.41469 | 1.05E-75 | 2.89E-73 | Down |
| AC092071.1 | -4.325397 | 1.73E-42 | 1.21E-40 | Down |
| AC016831.5 | -2.009918 | 5.90E-13 | 4.72E-12 | Down |
| LINC02104 | -2.201649 | 2.31E-17 | 2.93E-16 | Down |
| AL138900.3 | -2.787565 | 2.33E-13 | 1.97E-12 | Down |
| AC091544.5 | -2.043173 | 1.30E-13 | 1.13E-12 | Down |
| AC005740.4 | -2.084427 | 1.68E-37 | 9.36E-36 | Down |
| AC012467.1 | -2.240474 | 8.57E-14 | 7.61E-13 | Down |
| LINC00551 | -2.586312 | 5.57E-21 | 9.44E-20 | Down |
| AC116312.1 | -2.206956 | 9.20E-14 | 8.15E-13 | Down |
| AL357054.4 | -2.299094 | 1.20E-27 | 3.44E-26 | Down |
| AC006273.1 | -3.088027 | 4.66E-58 | 6.56E-56 | Down |
| AL109659.2 | -2.178087 | 7.44E-22 | 1.37E-20 | Down |
| AC009974.1 | -2.20559 | 1.61E-21 | 2.87E-20 | Down |
| AC012511.1 | -2.285591 | 1.72E-24 | 3.96E-23 | Down |
| AC004982.1 | -2.297866 | 2.18E-34 | 9.68E-33 | Down |
| AC147067.2 | -3.022683 | 3.03E-48 | 2.80E-46 | Down |
| AC019193.2 | -2.582576 | 1.31E-34 | 5.95E-33 | Down |
| AC010976.2 | -3.631541 | 3.33E-53 | 3.93E-51 | Down |
| AC006238.1 | -2.446692 | 3.15E-33 | 1.29E-31 | Down |
| AL445423.1 | -3.023429 | 2.40E-46 | 2.07E-44 | Down |
| AC011899.3 | -2.723648 | 7.02E-34 | 2.95E-32 | Down |
| AC016717.2 | -2.117907 | 2.14E-09 | 1.07E-08 | Down |
| AC004921.1 | -2.056397 | 3.71E-25 | 9.09E-24 | Down |
| AC027449.1 | -2.02847 | 2.45E-15 | 2.55E-14 | Down |
| SFTPD-AS1 | -2.426604 | 9.39E-28 | 2.73E-26 | Down |
| AC104836.1 | -2.057262 | 3.34E-27 | 9.37E-26 | Down |
| AGBL1 | -3.411646 | 1.78E-28 | 5.34E-27 | Down |
| AC024337.2 | -2.163923 | 7.27E-24 | 1.58E-22 | Down |
| AC018755.4 | -2.206089 | 3.73E-20 | 6.03E-19 | Down |
| AC236972.3 | -5.666804 | 2.56E-77 | 7.61E-75 | Down |
| AL135999.3 | -4.338275 | 2.60E-38 | 1.53E-36 | Down |
| AC024909.2 | -2.423337 | 4.10E-34 | 1.76E-32 | Down |
| AC106028.4 | -2.646659 | 3.06E-42 | 2.14E-40 | Down |
| AL031665.1 | -2.034786 | 5.26E-23 | 1.08E-21 | Down |
| AC023449.2 | -2.354966 | 2.72E-27 | 7.69E-26 | Down |
| AL034397.3 | -2.301103 | 1.66E-21 | 2.94E-20 | Down |
| AC007671.1 | -2.563723 | 3.85E-31 | 1.37E-29 | Down |
| AC018529.1 | -2.774587 | 1.89E-37 | 1.05E-35 | Down |
| AC245884.11 | -2.343794 | 8.98E-23 | 1.79E-21 | Down |
| AL445493.3 | -3.840301 | 6.83E-50 | 6.94E-48 | Down |
| AC104237.2 | -3.029594 | 1.98E-30 | 6.74E-29 | Down |
| AL133320.1 | -3.031224 | 2.27E-11 | 1.45E-10 | Down |
| AL021578.1 | -2.132519 | 6.28E-25 | 1.51E-23 | Down |
| AC104237.3 | -2.991219 | 3.39E-32 | 1.28E-30 | Down |
| AC025271.4 | -3.552575 | 6.39E-43 | 4.58E-41 | Down |
| AC124319.3 | -2.078173 | 3.80E-25 | 9.26E-24 | Down |
| AC012409.3 | -2.314676 | 7.44E-22 | 1.37E-20 | Down |
| AC110048.2 | -2.035648 | 3.05E-22 | 5.85E-21 | Down |
| AC013553.3 | -2.556747 | 2.57E-39 | 1.61E-37 | Down |
| AC005277.2 | -2.361579 | 8.80E-22 | 1.60E-20 | Down |
| PGM5P3-AS1 | -3.642203 | 2.50E-36 | 1.31E-34 | Down |
| AC007906.2 | -2.146767 | 4.71E-15 | 4.79E-14 | Down |
| AC037198.2 | -2.901298 | 4.82E-35 | 2.26E-33 | Down |
| BANCR | -2.141741 | 5.26E-09 | 2.48E-08 | Down |
| LINC02033 | -2.133532 | 3.80E-28 | 1.13E-26 | Down |
| AC091891.2 | -2.786771 | 1.02E-15 | 1.09E-14 | Down |
| LINC00891 | -3.746666 | 5.57E-66 | 1.09E-63 | Down |
| LINC01587 | 2.4090046 | 8.67E-08 | 3.41E-07 | Up |
| IGF2-AS | 3.1073459 | 2.72E-05 | 7.17E-05 | Up |
| H19 | 3.4608256 | 1.60E-06 | 5.13E-06 | Up |
| LINC00470 | 4.0681725 | 1.09E-10 | 6.41E-10 | Up |
| LINC00525 | 2.0374842 | 3.80E-06 | 1.16E-05 | Up |
| PART1 | 4.4543045 | 3.81E-12 | 2.75E-11 | Up |
| C2orf48 | 2.7302805 | 5.24E-16 | 5.77E-15 | Up |
| LINC01116 | 2.863853 | 8.08E-12 | 5.52E-11 | Up |
| IGF2BP2-AS1 | 3.2190841 | 2.98E-11 | 1.88E-10 | Up |
| OR51B5 | 2.7537227 | 0.0004639 | 0.0009885 | Up |
| MIR31HG | 4.7977092 | 6.45E-11 | 3.91E-10 | Up |
| AP000769.1 | 2.4362225 | 7.62E-17 | 9.16E-16 | Up |
| CCDC197 | 2.1294756 | 0.000952 | 0.001929 | Up |
| LINC00303 | 3.2554802 | 0.0001732 | 0.0003962 | Up |
| TYMSOS | 3.0410568 | 1.74E-23 | 3.67E-22 | Up |
| AP000679.1 | 3.2690032 | 6.54E-16 | 7.09E-15 | Up |
| LINC01561 | 3.8620923 | 2.00E-12 | 1.51E-11 | Up |
| AC020907.1 | 6.2132545 | 1.82E-23 | 3.83E-22 | Up |
| C10orf91 | 2.7932953 | 3.48E-13 | 2.88E-12 | Up |
| LINC01559 | 5.2081786 | 1.24E-10 | 7.24E-10 | Up |
| OGFRP1 | 2.519114 | 1.06E-27 | 3.06E-26 | Up |
| LINC00518 | 5.186757 | 4.49E-09 | 2.14E-08 | Up |
| B3GALT5-AS1 | 3.5123508 | 1.66E-06 | 5.32E-06 | Up |
| LINC01405 | 4.7237991 | 5.02E-08 | 2.05E-07 | Up |
| TCL6 | 2.0526839 | 0.0007147 | 0.0014788 | Up |
| AC010969.1 | 2.3691757 | 0.0011645 | 0.002322 | Up |
| LINC00319 | 2.4321994 | 1.72E-05 | 4.72E-05 | Up |
| AC069277.1 | 5.3651692 | 2.97E-27 | 8.35E-26 | Up |
| LINC00615 | 7.3691369 | 6.32E-12 | 4.38E-11 | Up |
| LINC00173 | 2.3053155 | 1.97E-10 | 1.12E-09 | Up |
| NPSR1-AS1 | 6.9022922 | 1.48E-26 | 4.01E-25 | Up |
| AC068631.1 | 2.7682424 | 6.07E-11 | 3.70E-10 | Up |
| AC090673.1 | 2.3149303 | 1.42E-11 | 9.31E-11 | Up |
| AL157838.1 | 2.0667283 | 1.43E-21 | 2.56E-20 | Up |
| CYYR1-AS1 | 2.5288624 | 8.85E-07 | 2.97E-06 | Up |
| MUC2 | 5.4185847 | 1.64E-08 | 7.17E-08 | Up |
| IQANK1 | 2.9241755 | 2.45E-41 | 1.62E-39 | Up |
| LINC00970 | 2.7137975 | 5.93E-05 | 0.0001475 | Up |
| LINC00501 | 3.1879884 | 1.90E-06 | 6.04E-06 | Up |
| SLC12A5-AS1 | 2.002467 | 1.20E-07 | 4.62E-07 | Up |
| TDRG1 | 5.8966322 | 8.56E-07 | 2.88E-06 | Up |
| AL590644.1 | 5.1603013 | 4.14E-13 | 3.39E-12 | Up |
| PKP4-AS1 | 3.2282684 | 4.84E-14 | 4.41E-13 | Up |
| LINC01854 | 6.8760403 | 1.84E-07 | 6.88E-07 | Up |
| ST8SIA6-AS1 | 2.8837003 | 0.0001535 | 0.0003545 | Up |
| FAM83A-AS1 | 6.3497676 | 3.36E-17 | 4.21E-16 | Up |
| LINC01121 | 2.0233356 | 1.77E-08 | 7.71E-08 | Up |
| LINC01602 | 3.0561712 | 0.0002181 | 0.0004918 | Up |
| AC005863.1 | 5.2202153 | 6.85E-11 | 4.14E-10 | Up |
| LINC01460 | 3.1194257 | 8.84E-12 | 6.01E-11 | Up |
| CALML3-AS1 | 6.1301581 | 6.68E-37 | 3.64E-35 | Up |
| LINC01446 | 3.7538016 | 0.000374 | 0.0008093 | Up |
| LINC00898 | 7.1420069 | 4.33E-24 | 9.70E-23 | Up |
| ADARB2-AS1 | 2.2696857 | 9.03E-05 | 0.0002171 | Up |
| AC006305.1 | 5.3553149 | 2.14E-24 | 4.90E-23 | Up |
| DUXAP8 | 3.5058919 | 1.38E-20 | 2.30E-19 | Up |
| AC117402.1 | 4.2712778 | 2.22E-12 | 1.66E-11 | Up |
| EWSAT1 | 3.3912289 | 2.35E-08 | 1.01E-07 | Up |
| FIRRE | 3.8703541 | 1.66E-21 | 2.94E-20 | Up |
| AC019080.1 | 2.5288304 | 7.78E-11 | 4.66E-10 | Up |
| UCA1 | 4.0122539 | 1.43E-09 | 7.29E-09 | Up |
| LINC00887 | 4.0574867 | 6.42E-18 | 8.57E-17 | Up |
| AC087491.1 | 2.4149118 | 2.96E-06 | 9.18E-06 | Up |
| CDIPTOSP | 4.0199123 | 1.54E-17 | 1.99E-16 | Up |
| AC010168.1 | 4.9662043 | 1.19E-38 | 7.28E-37 | Up |
| AC131097.1 | 3.1140201 | 6.21E-08 | 2.51E-07 | Up |
| LINC01139 | 2.9715423 | 5.65E-06 | 1.66E-05 | Up |
| LINC01356 | 2.4765951 | 7.74E-08 | 3.08E-07 | Up |
| AC007249.1 | 2.8621077 | 2.45E-09 | 1.21E-08 | Up |
| AC073316.1 | 5.5224013 | 4.10E-10 | 2.25E-09 | Up |
| AC011298.1 | 4.2911284 | 1.84E-06 | 5.88E-06 | Up |
| LINC02076 | 3.0096752 | 8.08E-13 | 6.38E-12 | Up |
| AC246793.1 | 4.5560971 | 7.81E-15 | 7.74E-14 | Up |
| AC112721.2 | 2.1374523 | 3.24E-06 | 9.96E-06 | Up |
| LINC02068 | 2.2760613 | 5.31E-08 | 2.17E-07 | Up |
| AC009988.1 | 5.0133211 | 7.21E-11 | 4.33E-10 | Up |
| LINC01615 | 3.5631311 | 4.97E-12 | 3.50E-11 | Up |
| SAMSN1-AS1 | 2.2010341 | 9.70E-06 | 2.75E-05 | Up |
| LINC01208 | 5.0050442 | 3.46E-14 | 3.22E-13 | Up |
| LINP1 | 4.8470464 | 2.14E-11 | 1.38E-10 | Up |
| AC073365.1 | 7.1103363 | 1.52E-12 | 1.16E-11 | Up |
| AC004870.2 | 3.8654244 | 2.79E-05 | 7.36E-05 | Up |
| LINC01805 | 3.1979594 | 6.40E-06 | 1.87E-05 | Up |
| LINC01707 | 2.8278496 | 2.64E-05 | 6.98E-05 | Up |
| SMCR2 | 2.3724486 | 4.02E-10 | 2.21E-09 | Up |
| LINC02561 | 4.0702594 | 8.24E-12 | 5.62E-11 | Up |
| AC091729.1 | 2.9915313 | 1.69E-10 | 9.71E-10 | Up |
| POU6F2-AS1 | 3.672925 | 3.31E-09 | 1.61E-08 | Up |
| MIR548XHG | 7.6781814 | 1.69E-08 | 7.37E-08 | Up |
| AL390729.1 | 3.0566263 | 1.25E-08 | 5.56E-08 | Up |
| AC008278.1 | 3.4128975 | 7.62E-06 | 2.20E-05 | Up |
| LINC00466 | 4.2204037 | 1.65E-10 | 9.53E-10 | Up |
| AL391427.1 | 3.8047537 | 4.86E-12 | 3.43E-11 | Up |
| LINC01133 | 5.1087759 | 2.90E-16 | 3.28E-15 | Up |
| AL023754.1 | 7.4298249 | 4.18E-20 | 6.67E-19 | Up |
| AP000697.1 | 3.8636374 | 2.50E-10 | 1.41E-09 | Up |
| AL117329.1 | 7.5210559 | 1.57E-36 | 8.40E-35 | Up |
| AC131097.3 | 2.2641476 | 6.30E-08 | 2.54E-07 | Up |
| LINC01527 | 4.7622085 | 5.05E-10 | 2.72E-09 | Up |
| AL139246.1 | 2.8097978 | 1.09E-08 | 4.88E-08 | Up |
| LINC01280 | 4.7160451 | 2.36E-11 | 1.51E-10 | Up |
| AC114489.1 | 6.8904173 | 1.17E-26 | 3.18E-25 | Up |
| AP001476.1 | 2.2599463 | 0.000839 | 0.0017174 | Up |
| AL606970.1 | 3.8520863 | 4.09E-07 | 1.45E-06 | Up |
| AC092803.1 | 2.9233602 | 1.74E-05 | 4.75E-05 | Up |
| LINC01087 | 3.932407 | 1.56E-05 | 4.30E-05 | Up |
| LINC01117 | 2.4500864 | 1.55E-09 | 7.83E-09 | Up |
| LINC00885 | 2.741208 | 2.15E-13 | 1.83E-12 | Up |
| AL513164.1 | 2.3689734 | 0.0005834 | 0.0012225 | Up |
| AC024600.1 | 2.4258838 | 0.0021679 | 0.0041018 | Up |
| AC098936.1 | 2.8918713 | 5.08E-06 | 1.51E-05 | Up |
| AL391704.1 | 4.6573235 | 4.21E-09 | 2.02E-08 | Up |
| AL353768.1 | 2.2559897 | 4.67E-06 | 1.40E-05 | Up |
| AC012363.1 | 4.7016829 | 8.06E-06 | 2.32E-05 | Up |
| AL355482.1 | 2.1411286 | 0.0001239 | 0.0002913 | Up |
| AC114812.2 | 5.4085864 | 6.07E-18 | 8.11E-17 | Up |
| AC010789.1 | 4.9545148 | 2.61E-07 | 9.52E-07 | Up |
| AC093843.1 | 2.9130605 | 1.04E-05 | 2.94E-05 | Up |
| AL161908.1 | 3.550484 | 5.22E-07 | 1.83E-06 | Up |
| LINC00393 | 7.5199919 | 1.77E-13 | 1.51E-12 | Up |
| CDKN2A-DT | 3.5570156 | 4.29E-08 | 1.77E-07 | Up |
| AC019155.3 | 5.633845 | 6.11E-15 | 6.10E-14 | Up |
| LINC00184 | 2.4774604 | 1.34E-08 | 5.94E-08 | Up |
| LINC01752 | 4.3142357 | 8.03E-19 | 1.15E-17 | Up |
| U82695.1 | 2.1270867 | 5.63E-05 | 0.0001409 | Up |
| AL512363.1 | 6.9446804 | 7.84E-34 | 3.28E-32 | Up |
| AL355483.1 | 3.6938396 | 7.15E-13 | 5.70E-12 | Up |
| LINC00337 | 2.6207345 | 1.33E-13 | 1.15E-12 | Up |
| AL583808.1 | 5.522275 | 1.13E-24 | 2.62E-23 | Up |
| AC092484.1 | 6.5399855 | 2.57E-11 | 1.63E-10 | Up |
| LINC00237 | 3.1302482 | 0.000347 | 0.000756 | Up |
| SLC9A3-AS1 | 2.6061592 | 7.50E-15 | 7.45E-14 | Up |
| AC073957.1 | 3.2283108 | 1.73E-05 | 4.72E-05 | Up |
| AC012354.1 | 2.9880432 | 1.04E-05 | 2.94E-05 | Up |
| AL662890.1 | 2.1495395 | 2.26E-05 | 6.07E-05 | Up |
| OSTM1-AS1 | 4.8367167 | 2.77E-06 | 8.64E-06 | Up |
| AL590617.2 | 2.1828734 | 7.36E-19 | 1.06E-17 | Up |
| MIR137HG | 5.9610209 | 5.49E-09 | 2.58E-08 | Up |
| AP000527.1 | 6.18785 | 1.40E-09 | 7.14E-09 | Up |
| LINC00705 | 2.1931783 | 6.78E-05 | 0.0001669 | Up |
| AL354707.1 | 2.8373861 | 6.65E-15 | 6.62E-14 | Up |
| CYP4A22-AS1 | 2.8680877 | 3.78E-20 | 6.10E-19 | Up |
| LINC01703 | 2.9479743 | 2.71E-21 | 4.71E-20 | Up |
| LINC01393 | 2.1275717 | 1.00E-12 | 7.83E-12 | Up |
| LINC01980 | 8.4574525 | 3.72E-39 | 2.31E-37 | Up |
| AL163953.1 | 6.6769268 | 4.77E-10 | 2.59E-09 | Up |
| MIAT | 2.2591196 | 1.44E-07 | 5.50E-07 | Up |
| AL109610.1 | 4.0640464 | 3.88E-08 | 1.62E-07 | Up |
| AC007953.1 | 2.0521083 | 0.003063 | 0.0056088 | Up |
| AC073321.1 | 3.7755584 | 2.19E-11 | 1.41E-10 | Up |
| AC092669.1 | 4.3759235 | 8.83E-11 | 5.25E-10 | Up |
| LINC00626 | 7.1199367 | 6.42E-16 | 6.98E-15 | Up |
| AL162431.1 | 2.0427336 | 1.03E-06 | 3.43E-06 | Up |
| AC016582.1 | 2.3153039 | 4.33E-06 | 1.30E-05 | Up |
| LINC01456 | 6.8652868 | 3.34E-15 | 3.45E-14 | Up |
| SATB2-AS1 | 3.3416459 | 1.28E-18 | 1.79E-17 | Up |
| LINC00381 | 2.4342977 | 7.28E-06 | 2.11E-05 | Up |
| AL122058.1 | 3.3389818 | 6.38E-11 | 3.87E-10 | Up |
| AC099066.1 | 2.52396 | 5.67E-08 | 2.31E-07 | Up |
| HAGLROS | 4.5037324 | 1.32E-23 | 2.82E-22 | Up |
| LINC01876 | 2.3667377 | 6.85E-16 | 7.42E-15 | Up |
| PARD3-AS1 | 2.414674 | 1.15E-13 | 1.00E-12 | Up |
| LINC02542 | 2.5245055 | 2.01E-14 | 1.90E-13 | Up |
| LINC01748 | 5.3660615 | 1.37E-36 | 7.37E-35 | Up |
| LINC01918 | 2.686 | 1.23E-06 | 4.04E-06 | Up |
| UPK1A-AS1 | 2.1814856 | 0.0010464 | 0.0021055 | Up |
| CYP4F26P | 3.8143927 | 3.98E-15 | 4.08E-14 | Up |
| AL359979.1 | 2.2133299 | 0.0004112 | 0.0008829 | Up |
| LINC00974 | 3.6659641 | 3.05E-08 | 1.29E-07 | Up |
| AC019118.1 | 2.5520168 | 2.92E-08 | 1.24E-07 | Up |
| TEX41 | 2.4683287 | 9.39E-11 | 5.57E-10 | Up |
| FGF12-AS3 | 2.7898705 | 1.27E-06 | 4.13E-06 | Up |
| AC007966.1 | 2.8767486 | 2.37E-11 | 1.52E-10 | Up |
| NAALADL2-AS2 | 5.9434706 | 1.03E-14 | 1.00E-13 | Up |
| LINC00161 | 2.201949 | 9.28E-06 | 2.65E-05 | Up |
| AC092167.1 | 2.6428041 | 1.09E-06 | 3.60E-06 | Up |
| LINC02527 | 5.2940535 | 6.30E-14 | 5.65E-13 | Up |
| LINC00511 | 3.9343956 | 5.74E-41 | 3.69E-39 | Up |
| Z98257.1 | 2.5428963 | 0.0001965 | 0.000446 | Up |
| AC084149.1 | 3.5938622 | 5.54E-07 | 1.92E-06 | Up |
| AL512785.1 | 5.1547426 | 3.78E-08 | 1.58E-07 | Up |
| LINC01630 | 2.2718048 | 0.0006679 | 0.001387 | Up |
| AC110015.1 | 2.5766748 | 0.000413 | 0.0008861 | Up |
| DLG1-AS1 | 2.0105816 | 2.33E-09 | 1.16E-08 | Up |
| AC012501.1 | 5.7940723 | 7.21E-13 | 5.74E-12 | Up |
| AC099066.2 | 3.0206381 | 1.24E-11 | 8.26E-11 | Up |
| SLC2A1-AS1 | 3.3672416 | 3.48E-29 | 1.12E-27 | Up |
| AL122019.1 | 4.5294517 | 3.16E-08 | 1.34E-07 | Up |
| SOX21-AS1 | 4.3400839 | 6.41E-23 | 1.30E-21 | Up |
| STEAP2-AS1 | 2.9422048 | 5.36E-13 | 4.31E-12 | Up |
| LINC00355 | 7.3979982 | 1.34E-15 | 1.42E-14 | Up |
| AL033504.1 | 2.6056759 | 0.0016336 | 0.0031631 | Up |
| AL358394.1 | 3.5888995 | 4.73E-07 | 1.67E-06 | Up |
| AL391097.2 | 2.3586988 | 9.00E-07 | 3.01E-06 | Up |
| LINC01850 | 2.7172137 | 0.0001056 | 0.0002516 | Up |
| MRLN | 2.6394077 | 0.0011385 | 0.0022747 | Up |
| SELENOOLP | 3.93436 | 7.86E-06 | 2.26E-05 | Up |
| LINC01429 | 4.0594033 | 9.54E-12 | 6.46E-11 | Up |
| RAPGEF4-AS1 | 3.5584401 | 2.02E-14 | 1.91E-13 | Up |
| AC069257.1 | 2.0585347 | 2.07E-10 | 1.18E-09 | Up |
| AL160408.1 | 2.0936097 | 4.87E-06 | 1.45E-05 | Up |
| MELTF-AS1 | 3.0023493 | 5.07E-32 | 1.90E-30 | Up |
| LINC01494 | 2.687754 | 3.51E-06 | 1.07E-05 | Up |
| AC097713.1 | 6.0271869 | 2.57E-11 | 1.63E-10 | Up |
| AL355607.1 | 2.0698455 | 0.0001139 | 0.0002694 | Up |
| LINC01320 | 4.0201175 | 8.95E-05 | 0.0002153 | Up |
| LINC02048 | 2.4683134 | 1.67E-05 | 4.56E-05 | Up |
| AL021707.2 | 2.022315 | 4.04E-34 | 1.74E-32 | Up |
| PCAT6 | 2.7785304 | 5.57E-30 | 1.88E-28 | Up |
| TBX18-AS1 | 3.4692934 | 5.04E-08 | 2.06E-07 | Up |
| LINC00392 | 9.0585089 | 3.00E-10 | 1.68E-09 | Up |
| LINC01546 | 2.2867119 | 2.54E-06 | 7.93E-06 | Up |
| AC106875.1 | 5.8749995 | 2.20E-06 | 6.92E-06 | Up |
| AC022387.1 | 2.8487015 | 1.52E-05 | 4.19E-05 | Up |
| AL138962.1 | 2.3530935 | 4.17E-05 | 0.0001063 | Up |
| AC010731.2 | 4.7684276 | 2.93E-13 | 2.46E-12 | Up |
| AC005042.2 | 4.6549477 | 7.59E-09 | 3.49E-08 | Up |
| HOTAIR | 6.807011 | 4.48E-15 | 4.56E-14 | Up |
| AL023755.1 | 3.1311251 | 1.09E-16 | 1.28E-15 | Up |
| LINC00659 | 3.8618403 | 2.38E-08 | 1.02E-07 | Up |
| LINC02575 | 2.3560482 | 0.0006033 | 0.0012617 | Up |
| LINC01344 | 2.2168327 | 2.54E-05 | 6.74E-05 | Up |
| AC107419.1 | 5.4110896 | 1.44E-12 | 1.10E-11 | Up |
| AL365356.3 | 4.4778498 | 7.47E-10 | 3.93E-09 | Up |
| LINC02041 | 2.5246399 | 2.83E-11 | 1.79E-10 | Up |
| SATB1-AS1 | 2.2012877 | 2.07E-13 | 1.76E-12 | Up |
| AL356479.1 | 3.5793395 | 7.85E-08 | 3.11E-07 | Up |
| AC009955.1 | 3.2547797 | 2.53E-09 | 1.25E-08 | Up |
| AL445471.1 | 2.1819243 | 9.05E-13 | 7.09E-12 | Up |
| AC233280.1 | 3.124646 | 1.27E-15 | 1.36E-14 | Up |
| AC022201.1 | 5.4219306 | 3.81E-10 | 2.10E-09 | Up |
| LINC01981 | 5.3389658 | 5.77E-20 | 9.09E-19 | Up |
| AL596223.1 | 3.8034434 | 1.80E-05 | 4.90E-05 | Up |
| AL133353.1 | 2.8236005 | 1.67E-16 | 1.95E-15 | Up |
| AL353693.1 | 4.9677428 | 1.93E-14 | 1.83E-13 | Up |
| AL139246.4 | 2.9528434 | 1.32E-08 | 5.87E-08 | Up |
| AL596330.1 | 4.6424895 | 5.99E-08 | 2.43E-07 | Up |
| MIR5689HG | 2.9569084 | 6.17E-07 | 2.13E-06 | Up |
| LINC00858 | 4.825186 | 1.99E-17 | 2.55E-16 | Up |
| AL451062.1 | 3.8688083 | 1.11E-06 | 3.65E-06 | Up |
| AC053503.2 | 2.2308751 | 8.16E-09 | 3.73E-08 | Up |
| AL390115.1 | 2.0481441 | 0.0001135 | 0.0002686 | Up |
| AC011287.1 | 6.9842113 | 2.66E-22 | 5.13E-21 | Up |
| MYOSLID | 3.2657824 | 1.17E-15 | 1.25E-14 | Up |
| AL161636.1 | 4.3269041 | 5.20E-07 | 1.82E-06 | Up |
| AL109924.2 | 2.1964581 | 4.44E-05 | 0.0001128 | Up |
| EMX2OS | 2.6408324 | 0.0008983 | 0.0018285 | Up |
| STEAP3-AS1 | 2.1882995 | 3.98E-09 | 1.91E-08 | Up |
| CASC20 | 5.6518411 | 3.61E-14 | 3.34E-13 | Up |
| AL157373.2 | 2.03855 | 6.50E-07 | 2.23E-06 | Up |
| AC128709.1 | 2.2822856 | 2.25E-05 | 6.03E-05 | Up |
| TFAP2A-AS1 | 3.4860435 | 3.08E-45 | 2.45E-43 | Up |
| AL590666.2 | 3.1788955 | 1.57E-09 | 7.94E-09 | Up |
| AL357143.1 | 2.3594524 | 0.0001273 | 0.0002989 | Up |
| AC080129.1 | 3.8199545 | 6.13E-06 | 1.80E-05 | Up |
| MAGEA4-AS1 | 8.0162438 | 2.12E-14 | 2.00E-13 | Up |
| AC007128.1 | 6.44759 | 8.78E-54 | 1.07E-51 | Up |
| AC006357.1 | 4.5091589 | 2.06E-06 | 6.51E-06 | Up |
| TRPM2-AS | 4.731526 | 1.53E-21 | 2.75E-20 | Up |
| FGF12-AS2 | 2.2422561 | 5.99E-05 | 0.0001489 | Up |
| AP000688.1 | 2.3246841 | 7.45E-10 | 3.93E-09 | Up |
| NFE4 | 2.7552208 | 0.0024886 | 0.0046513 | Up |
| ELOVL2-AS1 | 2.4270309 | 0.0001988 | 0.000451 | Up |
| FEZF1-AS1 | 5.9697195 | 5.99E-23 | 1.22E-21 | Up |
| AL121580.1 | 4.5700938 | 3.69E-16 | 4.13E-15 | Up |
| DSCR9 | 2.4568544 | 6.51E-09 | 3.02E-08 | Up |
| AL118508.1 | 3.3893133 | 1.84E-07 | 6.88E-07 | Up |
| LINC01048 | 2.1538093 | 7.91E-05 | 0.0001919 | Up |
| AC114803.1 | 5.1362799 | 1.54E-16 | 1.79E-15 | Up |
| AL356234.2 | 5.0381273 | 1.99E-16 | 2.30E-15 | Up |
| AL645608.3 | 2.9415694 | 1.00E-08 | 4.52E-08 | Up |
| AL035252.2 | 3.1110175 | 1.02E-05 | 2.89E-05 | Up |
| FOXD3-AS1 | 6.9066613 | 2.66E-28 | 7.90E-27 | Up |
| AC068580.2 | 3.5347249 | 5.67E-09 | 2.66E-08 | Up |
| LINC01614 | 2.4646709 | 2.68E-08 | 1.14E-07 | Up |
| MAGEA8-AS1 | 2.2809865 | 3.14E-05 | 8.17E-05 | Up |
| AL079301.1 | 3.6774523 | 4.05E-06 | 1.23E-05 | Up |
| MIR205HG | 6.1380881 | 7.30E-46 | 5.92E-44 | Up |
| LINC02541 | 3.7534273 | 2.57E-23 | 5.38E-22 | Up |
| AL049649.1 | 5.2966996 | 2.14E-10 | 1.21E-09 | Up |
| AL355303.1 | 2.9141877 | 4.70E-09 | 2.23E-08 | Up |
| AC093833.1 | 2.2965938 | 4.23E-05 | 0.0001078 | Up |
| LINC02522 | 4.011898 | 1.66E-07 | 6.26E-07 | Up |
| AL078587.1 | 2.3870036 | 1.82E-10 | 1.04E-09 | Up |
| LINC01468 | 6.9071452 | 2.64E-15 | 2.74E-14 | Up |
| AC093702.1 | 4.1598711 | 1.80E-11 | 1.18E-10 | Up |
| LINC01098 | 3.0114649 | 3.19E-05 | 8.31E-05 | Up |
| AC097347.1 | 2.9804967 | 1.06E-16 | 1.25E-15 | Up |
| APCDD1L-DT | 3.9614903 | 5.62E-10 | 3.01E-09 | Up |
| AC106799.1 | 6.1882903 | 1.29E-22 | 2.55E-21 | Up |
| AC004888.1 | 2.9478802 | 5.94E-06 | 1.74E-05 | Up |
| MANCR | 2.1452979 | 1.25E-05 | 3.49E-05 | Up |
| TBL1XR1-AS1 | 2.5540085 | 4.95E-11 | 3.04E-10 | Up |
| AP000696.1 | 4.5579091 | 2.13E-11 | 1.38E-10 | Up |
| LINC01160 | 2.1217941 | 2.99E-05 | 7.84E-05 | Up |
| AL445673.1 | 2.2682707 | 1.05E-08 | 4.74E-08 | Up |
| WASIR2 | 2.4430908 | 1.98E-08 | 8.59E-08 | Up |
| LINC01305 | 6.3072743 | 4.40E-21 | 7.53E-20 | Up |
| AL359636.2 | 2.1807186 | 3.05E-07 | 1.10E-06 | Up |
| AC074389.2 | 4.9652236 | 1.42E-05 | 3.93E-05 | Up |
| AC141930.2 | 2.0929755 | 0.0003558 | 0.0007729 | Up |
| AL365356.4 | 4.4986698 | 1.33E-14 | 1.28E-13 | Up |
| LINC01249 | 8.2119694 | 1.55E-14 | 1.48E-13 | Up |
| CLEC12A-AS1 | 3.3336027 | 1.36E-08 | 5.99E-08 | Up |
| AC234772.2 | 3.5527471 | 1.66E-21 | 2.94E-20 | Up |
| AC009501.1 | 2.6376625 | 3.59E-14 | 3.33E-13 | Up |
| LUARIS | 4.7569467 | 2.40E-17 | 3.04E-16 | Up |
| FSIP2-AS1 | 3.1318946 | 1.01E-16 | 1.19E-15 | Up |
| LINC01698 | 6.7277553 | 1.20E-18 | 1.67E-17 | Up |
| AL033397.1 | 7.6768373 | 2.40E-12 | 1.78E-11 | Up |
| AL354993.1 | 3.1798141 | 1.53E-19 | 2.30E-18 | Up |
| AC112907.1 | 4.3076309 | 4.36E-09 | 2.08E-08 | Up |
| ABCA9-AS1 | 4.1576617 | 1.73E-13 | 1.48E-12 | Up |
| CHODL-AS1 | 3.7010501 | 2.43E-08 | 1.04E-07 | Up |
| DLX6-AS1 | 6.9266486 | 1.26E-27 | 3.61E-26 | Up |
| Z93403.1 | 4.1534551 | 2.61E-19 | 3.89E-18 | Up |
| LINC01611 | 6.411667 | 2.32E-12 | 1.73E-11 | Up |
| PCAT7 | 4.2320643 | 1.33E-25 | 3.34E-24 | Up |
| LINC01819 | 2.7999262 | 0.00078 | 0.0016019 | Up |
| AL139393.1 | 4.3776199 | 2.54E-09 | 1.25E-08 | Up |
| AL031848.1 | 2.9223815 | 1.19E-09 | 6.12E-09 | Up |
| AL359551.1 | 4.9773664 | 2.35E-12 | 1.75E-11 | Up |
| DARS-AS1 | 2.2662568 | 5.15E-26 | 1.33E-24 | Up |
| AC073316.2 | 2.0919833 | 2.19E-06 | 6.90E-06 | Up |
| AC019185.2 | 2.9550464 | 9.90E-06 | 2.80E-05 | Up |
| AC116666.1 | 4.7395889 | 7.82E-08 | 3.11E-07 | Up |
| AL078590.2 | 4.3182036 | 1.68E-10 | 9.68E-10 | Up |
| AL356361.2 | 2.9193617 | 1.91E-05 | 5.17E-05 | Up |
| AC005537.1 | 5.6559554 | 2.20E-40 | 1.40E-38 | Up |
| LINC01807 | 7.6002458 | 4.51E-20 | 7.18E-19 | Up |
| LINC01063 | 2.4820916 | 5.72E-16 | 6.24E-15 | Up |
| LINC01697 | 5.7188072 | 9.12E-12 | 6.18E-11 | Up |
| LINC00867 | 2.8146939 | 0.0002942 | 0.0006484 | Up |
| LINC01873 | 4.6372142 | 1.87E-20 | 3.10E-19 | Up |
| Z99943.1 | 2.2592628 | 2.40E-06 | 7.54E-06 | Up |
| AL596442.1 | 3.6814786 | 4.54E-05 | 0.0001151 | Up |
| AL353613.1 | 2.841281 | 6.00E-05 | 0.000149 | Up |
| LINC02043 | 4.6365047 | 6.53E-16 | 7.09E-15 | Up |
| AL139327.2 | 6.4510093 | 5.37E-15 | 5.42E-14 | Up |
| AL031674.1 | 3.4846207 | 6.56E-08 | 2.64E-07 | Up |
| AL138902.1 | 4.4636833 | 4.93E-24 | 1.10E-22 | Up |
| AC093627.1 | 4.0091589 | 1.55E-06 | 4.98E-06 | Up |
| AL354864.1 | 2.092985 | 1.81E-06 | 5.77E-06 | Up |
| LINC00112 | 2.8036601 | 0.0006475 | 0.0013481 | Up |
| AC006329.1 | 3.3702112 | 5.86E-17 | 7.12E-16 | Up |
| AL354824.2 | 3.5600225 | 1.12E-07 | 4.35E-07 | Up |
| LINC01450 | 2.0353703 | 0.000118 | 0.0002786 | Up |
| AC092920.1 | 3.0789521 | 6.34E-07 | 2.19E-06 | Up |
| AC073323.1 | 4.1792178 | 3.13E-06 | 9.65E-06 | Up |
| AC104088.1 | 4.5448735 | 4.05E-20 | 6.48E-19 | Up |
| LINC01549 | 5.3369346 | 9.31E-08 | 3.65E-07 | Up |
| AL122034.1 | 3.0126529 | 3.89E-05 | 9.99E-05 | Up |
| LINC00665 | 2.2068107 | 4.75E-09 | 2.25E-08 | Up |
| LINC01705 | 3.1424744 | 4.86E-10 | 2.63E-09 | Up |
| AC002511.1 | 2.0908963 | 0.0012621 | 0.0025 | Up |
| AC097717.1 | 2.2906036 | 8.79E-11 | 5.23E-10 | Up |
| AC004990.1 | 2.6918303 | 1.25E-06 | 4.08E-06 | Up |
| AL355483.3 | 2.1611081 | 4.76E-06 | 1.43E-05 | Up |
| AC003986.2 | 2.7966425 | 9.72E-10 | 5.06E-09 | Up |
| AF165147.1 | 3.7979258 | 5.29E-12 | 3.70E-11 | Up |
| AF212831.1 | 3.6342812 | 1.04E-07 | 4.05E-07 | Up |
| AL157400.2 | 4.2471816 | 3.87E-15 | 3.98E-14 | Up |
| LINC01399 | 2.9168428 | 1.03E-07 | 4.01E-07 | Up |
| BX322234.2 | 2.4117285 | 0.0006636 | 0.0013785 | Up |
| AC002511.2 | 2.0784566 | 0.0009572 | 0.001938 | Up |
| LINC01687 | 5.9424888 | 1.90E-07 | 7.09E-07 | Up |
| AC133785.1 | 7.5333832 | 4.05E-41 | 2.64E-39 | Up |
| AL512604.2 | 2.460092 | 1.36E-08 | 6.02E-08 | Up |
| XXYLT1-AS1 | 2.6379688 | 2.83E-09 | 1.38E-08 | Up |
| AL122008.3 | 2.4548911 | 7.68E-06 | 2.22E-05 | Up |
| AL391361.3 | 5.1393155 | 1.66E-07 | 6.26E-07 | Up |
| AL035401.1 | 2.2102762 | 0.003896 | 0.0070045 | Up |
| AC096537.1 | 2.5171089 | 1.31E-07 | 5.02E-07 | Up |
| AL445524.1 | 2.6063122 | 3.28E-13 | 2.73E-12 | Up |
| AC008163.1 | 3.2233506 | 0.0027787 | 0.0051505 | Up |
| AL021395.1 | 4.6829864 | 4.00E-06 | 1.21E-05 | Up |
| LINC01518 | 8.4567842 | 6.53E-14 | 5.85E-13 | Up |
| AC005162.2 | 3.1579618 | 3.09E-08 | 1.31E-07 | Up |
| LINC00460 | 2.6667476 | 3.01E-05 | 7.88E-05 | Up |
| AL592043.1 | 5.5748682 | 9.64E-12 | 6.52E-11 | Up |
| AL138789.1 | 4.7457802 | 6.15E-17 | 7.44E-16 | Up |
| LINC00462 | 5.2103919 | 2.12E-10 | 1.20E-09 | Up |
| AC019068.1 | 4.3838657 | 1.13E-07 | 4.35E-07 | Up |
| LINC02579 | 2.1372119 | 4.85E-06 | 1.45E-05 | Up |
| MYCNOS | 3.8529495 | 4.84E-07 | 1.70E-06 | Up |
| KCNQ5-IT1 | 3.7827557 | 1.21E-10 | 7.07E-10 | Up |
| POU6F2-AS2 | 6.9887631 | 1.21E-36 | 6.57E-35 | Up |
| YEATS2-AS1 | 2.1480617 | 2.19E-17 | 2.80E-16 | Up |
| Z83851.1 | 2.5997941 | 5.67E-30 | 1.91E-28 | Up |
| AL591501.1 | 4.5986822 | 3.26E-09 | 1.59E-08 | Up |
| AC073529.1 | 2.0134578 | 5.75E-18 | 7.72E-17 | Up |
| LINC02535 | 2.9905769 | 3.11E-07 | 1.12E-06 | Up |
| AC006012.1 | 2.0711607 | 3.04E-05 | 7.95E-05 | Up |
| KCNH1-IT1 | 5.1188982 | 1.60E-10 | 9.26E-10 | Up |
| AL138720.1 | 2.7866955 | 2.63E-11 | 1.67E-10 | Up |
| DEPDC1-AS1 | 3.4259599 | 1.76E-17 | 2.27E-16 | Up |
| LINC01731 | 3.4015745 | 3.34E-05 | 8.69E-05 | Up |
| AC009264.1 | 7.9034933 | 1.22E-29 | 4.01E-28 | Up |
| LINC01250 | 3.5120558 | 8.40E-07 | 2.83E-06 | Up |
| AC092811.1 | 3.0514355 | 9.54E-07 | 3.18E-06 | Up |
| LINC00701 | 4.0012775 | 2.50E-07 | 9.14E-07 | Up |
| CHL1-AS1 | 2.2636624 | 2.34E-05 | 6.25E-05 | Up |
| AC002076.1 | 3.8621142 | 9.63E-21 | 1.62E-19 | Up |
| AF015262.1 | 3.1503844 | 2.14E-05 | 5.75E-05 | Up |
| AC005064.1 | 2.7038944 | 8.33E-05 | 0.0002014 | Up |
| LINC01287 | 6.687824 | 3.53E-08 | 1.48E-07 | Up |
| IQCM | 6.3362475 | 8.24E-19 | 1.17E-17 | Up |
| AC003958.2 | 6.2271705 | 6.22E-17 | 7.51E-16 | Up |
| AL035258.1 | 7.8161221 | 5.08E-16 | 5.61E-15 | Up |
| LINC01524 | 4.929781 | 7.22E-16 | 7.80E-15 | Up |
| AL512622.1 | 2.5375101 | 1.76E-06 | 5.63E-06 | Up |
| DPP10-AS1 | 3.1023876 | 0.0001678 | 0.0003849 | Up |
| AC062015.1 | 5.7735174 | 7.60E-08 | 3.02E-07 | Up |
| BX324167.1 | 2.7526761 | 5.91E-08 | 2.40E-07 | Up |
| FAM237A | 5.3259017 | 2.40E-09 | 1.19E-08 | Up |
| AL645504.1 | 2.0151836 | 1.36E-06 | 4.39E-06 | Up |
| AC128709.2 | 2.1394457 | 8.20E-05 | 0.0001983 | Up |
| LINC00601 | 4.1629676 | 1.51E-07 | 5.76E-07 | Up |
| FAM83C-AS1 | 2.4343089 | 1.18E-13 | 1.03E-12 | Up |
| AF127577.3 | 7.4145038 | 1.73E-29 | 5.68E-28 | Up |
| AC145207.1 | 2.3846508 | 2.49E-05 | 6.61E-05 | Up |
| AC016723.1 | 5.9173997 | 2.62E-10 | 1.48E-09 | Up |
| AC093083.1 | 4.2101201 | 9.38E-13 | 7.33E-12 | Up |
| LINC01967 | 5.9318137 | 3.39E-22 | 6.48E-21 | Up |
| AC103923.1 | 2.2818125 | 1.69E-12 | 1.28E-11 | Up |
| BARX1-DT | 7.0337 | 1.08E-18 | 1.52E-17 | Up |
| LINC01647 | 3.9906572 | 5.00E-05 | 0.000126 | Up |
| AC116614.1 | 2.4773379 | 0.0003502 | 0.0007617 | Up |
| AC007389.3 | 4.1205761 | 7.07E-12 | 4.85E-11 | Up |
| AC010148.1 | 2.8369323 | 5.40E-19 | 7.80E-18 | Up |
| AC099796.1 | 3.5350811 | 2.10E-06 | 6.64E-06 | Up |
| LINC00941 | 3.7520496 | 5.08E-13 | 4.11E-12 | Up |
| LINC01564 | 4.1332012 | 1.30E-15 | 1.38E-14 | Up |
| AC007405.2 | 3.3466973 | 2.31E-07 | 8.50E-07 | Up |
| AL138760.1 | 4.5512714 | 4.28E-12 | 3.05E-11 | Up |
| AL354766.2 | 2.1612301 | 0.0004797 | 0.0010191 | Up |
| AC004920.1 | 3.7722764 | 1.88E-08 | 8.15E-08 | Up |
| AL034399.2 | 5.2421722 | 6.79E-22 | 1.25E-20 | Up |
| ELFN1-AS1 | 3.5930606 | 3.71E-08 | 1.55E-07 | Up |
| LINC01639 | 6.2022375 | 9.04E-09 | 4.10E-08 | Up |
| PTCSC2 | 4.7432758 | 3.34E-12 | 2.44E-11 | Up |
| KDM4A-AS1 | 2.863397 | 8.82E-22 | 1.60E-20 | Up |
| AC009262.1 | 5.5116963 | 4.06E-24 | 9.12E-23 | Up |
| LINC01361 | 2.9222638 | 1.30E-12 | 1.00E-11 | Up |
| AC019197.1 | 2.977119 | 1.07E-06 | 3.53E-06 | Up |
| SLC12A9-AS1 | 2.586479 | 2.68E-19 | 3.98E-18 | Up |
| AL354719.2 | 3.7618927 | 2.28E-10 | 1.29E-09 | Up |
| AL513123.1 | 7.1966427 | 3.59E-27 | 1.00E-25 | Up |
| AL355472.3 | 3.1186682 | 4.30E-11 | 2.66E-10 | Up |
| AL358115.1 | 2.3738834 | 2.17E-20 | 3.57E-19 | Up |
| AL359313.1 | 3.4514645 | 1.16E-05 | 3.27E-05 | Up |
| LINC00479 | 2.6188194 | 8.76E-06 | 2.51E-05 | Up |
| AL121970.1 | 2.3792463 | 9.35E-05 | 0.0002246 | Up |
| AL589986.2 | 4.8271646 | 2.49E-15 | 2.59E-14 | Up |
| FAM157A | 2.1682165 | 2.94E-11 | 1.86E-10 | Up |
| AC010894.3 | 3.8923347 | 2.93E-10 | 1.64E-09 | Up |
| AC067956.1 | 2.7430122 | 4.53E-09 | 2.16E-08 | Up |
| AC003092.1 | 4.0228965 | 3.06E-05 | 7.99E-05 | Up |
| LINC00896 | 2.1300032 | 3.46E-12 | 2.52E-11 | Up |
| SIX3-AS1 | 2.1350486 | 0.0043515 | 0.0077403 | Up |
| ATP13A5-AS1 | 2.5584588 | 0.000104 | 0.0002484 | Up |
| LINC01695 | 2.0546724 | 2.05E-05 | 5.53E-05 | Up |
| AC003986.3 | 2.574008 | 4.94E-05 | 0.0001246 | Up |
| AL033527.2 | 2.6637949 | 2.07E-07 | 7.65E-07 | Up |
| DLX2-DT | 5.149669 | 1.34E-14 | 1.29E-13 | Up |
| AC006450.3 | 4.3904936 | 2.58E-07 | 9.44E-07 | Up |
| OVAAL | 2.9865617 | 0.0004538 | 0.0009679 | Up |
| AL033384.1 | 4.906426 | 1.27E-20 | 2.13E-19 | Up |
| LINC01563 | 2.3337027 | 0.0026913 | 0.0050023 | Up |
| AC024560.1 | 3.5640074 | 2.39E-09 | 1.19E-08 | Up |
| AC005865.1 | 3.3084912 | 1.15E-16 | 1.35E-15 | Up |
| AL356270.1 | 2.6842431 | 1.61E-07 | 6.10E-07 | Up |
| AL162411.1 | 2.1462661 | 4.95E-05 | 0.0001247 | Up |
| AL139412.1 | 3.660435 | 5.82E-16 | 6.33E-15 | Up |
| AL136131.2 | 2.7672651 | 3.89E-05 | 9.99E-05 | Up |
| MLIP-IT1 | 4.364757 | 3.38E-16 | 3.79E-15 | Up |
| HAND2-AS1 | 2.4485965 | 0.0030862 | 0.005646 | Up |
| LINC01792 | 2.8156193 | 1.50E-05 | 4.14E-05 | Up |
| AL445072.1 | 4.6769591 | 7.55E-13 | 5.99E-12 | Up |
| LINC01968 | 3.0304933 | 5.32E-13 | 4.29E-12 | Up |
| BX276092.7 | 5.4361481 | 5.50E-08 | 2.24E-07 | Up |
| LINC01343 | 3.8839926 | 2.99E-05 | 7.84E-05 | Up |
| AL034397.2 | 2.7253929 | 6.85E-05 | 0.0001684 | Up |
| TUSC8 | 2.7005206 | 0.0027973 | 0.005179 | Up |
| HOXD-AS2 | 3.5840947 | 2.82E-14 | 2.64E-13 | Up |
| CECR7 | 2.2311472 | 4.31E-06 | 1.30E-05 | Up |
| AL359771.1 | 4.3560785 | 7.12E-07 | 2.42E-06 | Up |
| AC096536.2 | 2.2335104 | 4.01E-09 | 1.93E-08 | Up |
| UNC5B-AS1 | 2.0491666 | 9.76E-08 | 3.81E-07 | Up |
| DGCR5 | 3.1782157 | 3.75E-17 | 4.65E-16 | Up |
| TTLL11-IT1 | 2.3620015 | 2.11E-05 | 5.68E-05 | Up |
| Z97206.2 | 3.4038658 | 5.83E-07 | 2.02E-06 | Up |
| AL590666.3 | 3.4128643 | 4.75E-09 | 2.25E-08 | Up |
| AP000251.1 | 4.7299339 | 2.53E-30 | 8.59E-29 | Up |
| AL161937.2 | 2.0027486 | 5.59E-09 | 2.62E-08 | Up |
| AL139039.3 | 2.7672552 | 3.60E-11 | 2.25E-10 | Up |
| AL109615.3 | 2.2361269 | 2.19E-09 | 1.09E-08 | Up |
| AC011287.2 | 3.7056697 | 1.09E-08 | 4.90E-08 | Up |
| AL136309.3 | 3.9207513 | 8.03E-05 | 0.0001947 | Up |
| AF130359.1 | 3.0741167 | 2.51E-06 | 7.87E-06 | Up |
| LINC01143 | 2.8763178 | 8.96E-06 | 2.56E-05 | Up |
| AC010099.4 | 2.7749342 | 1.53E-06 | 4.91E-06 | Up |
| LINC01370 | 7.4891029 | 3.15E-06 | 9.70E-06 | Up |
| DDR1-DT | 2.1120759 | 1.58E-07 | 5.96E-07 | Up |
| AC092422.1 | 2.1042467 | 0.0004788 | 0.0010174 | Up |
| AL035425.1 | 4.8771842 | 4.34E-09 | 2.07E-08 | Up |
| DGUOK-AS1 | 2.0883606 | 4.79E-18 | 6.47E-17 | Up |
| KCNMB2-AS1 | 7.3453703 | 2.70E-61 | 4.51E-59 | Up |
| AL606970.4 | 4.2720287 | 1.22E-06 | 4.00E-06 | Up |
| SPATA3-AS1 | 2.3441398 | 1.69E-07 | 6.35E-07 | Up |
| AC245100.6 | 3.8233179 | 1.61E-10 | 9.34E-10 | Up |
| AL121904.1 | 3.5952861 | 0.0007389 | 0.0015241 | Up |
| AC068196.1 | 2.8485481 | 8.09E-13 | 6.38E-12 | Up |
| LINC00391 | 4.0756188 | 2.43E-10 | 1.37E-09 | Up |
| LINC00707 | 3.6252744 | 6.36E-09 | 2.96E-08 | Up |
| LINC01448 | 4.8134633 | 2.94E-07 | 1.07E-06 | Up |
| LINC02031 | 3.4752856 | 5.22E-07 | 1.83E-06 | Up |
| ATP6V1B1-AS1 | 3.0322767 | 1.54E-09 | 7.79E-09 | Up |
| AC091212.1 | 2.0220889 | 7.68E-06 | 2.22E-05 | Up |
| AC019211.1 | 3.6712601 | 9.99E-15 | 9.74E-14 | Up |
| AC069439.2 | 3.21783 | 2.37E-05 | 6.31E-05 | Up |
| AC069431.1 | 4.0580582 | 7.38E-10 | 3.90E-09 | Up |
| AC128689.1 | 5.7410015 | 1.80E-21 | 3.17E-20 | Up |
| SAMMSON | 2.614725 | 7.15E-07 | 2.43E-06 | Up |
| LINC00973 | 3.6285778 | 7.67E-05 | 0.0001867 | Up |
| TM4SF1-AS1 | 2.6952202 | 3.50E-10 | 1.94E-09 | Up |
| LINC02067 | 2.3993349 | 3.74E-05 | 9.62E-05 | Up |
| AC063952.1 | 4.3814953 | 4.22E-22 | 7.99E-21 | Up |
| LINC02042 | 3.6709026 | 2.35E-07 | 8.62E-07 | Up |
| LSAMP-AS1 | 4.8135458 | 8.64E-17 | 1.03E-15 | Up |
| HOXA11-AS | 5.5410881 | 5.53E-21 | 9.40E-20 | Up |
| LINC01994 | 4.4876023 | 1.12E-08 | 5.00E-08 | Up |
| LINC01192 | 3.8810597 | 1.39E-06 | 4.49E-06 | Up |
| AL160408.4 | 5.2115031 | 1.38E-12 | 1.05E-11 | Up |
| PAQR9-AS1 | 3.3219425 | 1.29E-06 | 4.21E-06 | Up |
| AC123768.1 | 2.7348289 | 3.81E-08 | 1.59E-07 | Up |
| AC112493.1 | 3.0754831 | 3.61E-05 | 9.32E-05 | Up |
| AC084864.1 | 3.9150896 | 1.91E-22 | 3.73E-21 | Up |
| AC093904.2 | 3.3907607 | 1.57E-10 | 9.10E-10 | Up |
| AL365356.5 | 5.774845 | 1.88E-18 | 2.61E-17 | Up |
| HOXB-AS4 | 5.117676 | 2.49E-10 | 1.41E-09 | Up |
| AC121764.1 | 4.3117985 | 0.0002153 | 0.0004863 | Up |
| LINC01206 | 9.4261566 | 5.66E-15 | 5.69E-14 | Up |
| AC079943.2 | 4.1360377 | 3.90E-08 | 1.62E-07 | Up |
| AC107464.1 | 2.0555119 | 0.0002687 | 0.0005957 | Up |
| LINC02005 | 2.6952617 | 0.0002161 | 0.0004879 | Up |
| SOX2-OT | 5.7676061 | 5.54E-15 | 5.58E-14 | Up |
| PLCH1-AS2 | 2.3955334 | 1.51E-05 | 4.14E-05 | Up |
| AC011005.4 | 3.5933805 | 6.79E-20 | 1.06E-18 | Up |
| AC068633.1 | 3.9705736 | 7.37E-06 | 2.14E-05 | Up |
| LINC01998 | 3.1520521 | 0.0012984 | 0.0025687 | Up |
| MNX1-AS1 | 4.617629 | 3.05E-12 | 2.23E-11 | Up |
| AC068985.1 | 4.0735052 | 5.15E-08 | 2.11E-07 | Up |
| LINC01214 | 3.978064 | 3.98E-08 | 1.65E-07 | Up |
| CACNA2D3-AS1 | 2.3023895 | 3.47E-05 | 8.99E-05 | Up |
| HOTTIP | 5.6743981 | 5.06E-16 | 5.59E-15 | Up |
| AC117386.2 | 3.786091 | 1.59E-06 | 5.12E-06 | Up |
| LINC01322 | 5.1198947 | 4.29E-14 | 3.94E-13 | Up |
| AC091153.3 | 2.318564 | 5.41E-14 | 4.90E-13 | Up |
| LINC01995 | 4.6269442 | 1.46E-07 | 5.55E-07 | Up |
| AC117394.2 | 2.6001513 | 2.24E-16 | 2.58E-15 | Up |
| AC093904.3 | 4.0833541 | 8.47E-08 | 3.33E-07 | Up |
| LINC00698 | 2.8307409 | 1.99E-06 | 6.31E-06 | Up |
| AC055758.2 | 5.3619639 | 4.68E-08 | 1.92E-07 | Up |
| AC093001.1 | 5.9684 | 1.45E-06 | 4.68E-06 | Up |
| LINC01213 | 3.8359724 | 7.84E-08 | 3.11E-07 | Up |
| LINC01391 | 5.9175293 | 2.99E-13 | 2.51E-12 | Up |
| WNT5A-AS1 | 2.166453 | 7.15E-09 | 3.31E-08 | Up |
| LINC02086 | 2.9647955 | 8.17E-09 | 3.73E-08 | Up |
| AC108676.1 | 4.9333922 | 1.04E-11 | 7.02E-11 | Up |
| AL109761.1 | 2.3546828 | 3.14E-08 | 1.33E-07 | Up |
| AC087667.1 | 4.1319503 | 9.06E-13 | 7.09E-12 | Up |
| ARNTL2-AS1 | 3.6541415 | 5.91E-15 | 5.91E-14 | Up |
| AC026250.1 | 2.7127643 | 9.91E-20 | 1.52E-18 | Up |
| LINC00461 | 4.53361 | 1.49E-10 | 8.67E-10 | Up |
| DDX11-AS1 | 2.8945202 | 2.45E-31 | 8.82E-30 | Up |
| AC022075.1 | 4.0307278 | 6.06E-25 | 1.46E-23 | Up |
| MIR4300HG | 3.7523006 | 3.01E-05 | 7.87E-05 | Up |
| AF233439.1 | 2.8839867 | 1.07E-07 | 4.17E-07 | Up |
| LINC01096 | 6.020219 | 6.30E-23 | 1.28E-21 | Up |
| CASC8 | 4.545345 | 8.54E-25 | 2.02E-23 | Up |
| AC138904.1 | 3.3496228 | 7.10E-11 | 4.28E-10 | Up |
| LINC02466 | 6.2516484 | 1.13E-20 | 1.89E-19 | Up |
| AC099487.1 | 5.0118939 | 3.60E-23 | 7.48E-22 | Up |
| AC005329.1 | 2.156981 | 3.84E-07 | 1.37E-06 | Up |
| AC008443.3 | 3.1658926 | 1.43E-07 | 5.44E-07 | Up |
| AC108174.1 | 5.4104431 | 7.42E-10 | 3.91E-09 | Up |
| LINC01194 | 7.9550136 | 1.07E-09 | 5.56E-09 | Up |
| LINC02505 | 5.1643021 | 1.15E-06 | 3.79E-06 | Up |
| LINC02438 | 5.6352235 | 1.22E-11 | 8.16E-11 | Up |
| AC114956.1 | 3.5431445 | 8.76E-20 | 1.35E-18 | Up |
| AC004053.1 | 2.3740048 | 2.99E-07 | 1.08E-06 | Up |
| LINC02014 | 2.6050716 | 1.88E-12 | 1.42E-11 | Up |
| AC010307.2 | 2.2921582 | 4.61E-08 | 1.90E-07 | Up |
| AC023794.1 | 2.0434707 | 8.44E-09 | 3.85E-08 | Up |
| AC010275.1 | 6.4856194 | 9.13E-32 | 3.39E-30 | Up |
| LINC02120 | 2.3508356 | 0.0001637 | 0.0003766 | Up |
| AC010280.1 | 7.3699093 | 1.58E-19 | 2.37E-18 | Up |
| LINC00504 | 2.8644579 | 7.32E-08 | 2.92E-07 | Up |
| AC011352.1 | 3.6377778 | 3.44E-10 | 1.91E-09 | Up |
| LINC02122 | 2.3568402 | 0.0031748 | 0.0057897 | Up |
| AC016642.1 | 3.6402339 | 6.50E-05 | 0.0001606 | Up |
| AC024230.1 | 5.1455753 | 9.91E-11 | 5.86E-10 | Up |
| LINC02437 | 4.7090864 | 7.79E-09 | 3.58E-08 | Up |
| AC022784.1 | 3.6341067 | 5.99E-09 | 2.80E-08 | Up |
| OTX2-AS1 | 3.5851463 | 2.78E-06 | 8.66E-06 | Up |
| AC114956.2 | 4.3168598 | 6.74E-23 | 1.36E-21 | Up |
| GDNF-AS1 | 3.7110277 | 1.26E-09 | 6.48E-09 | Up |
| AC126768.1 | 5.2444167 | 1.43E-13 | 1.24E-12 | Up |
| AC018781.1 | 3.3704528 | 3.72E-12 | 2.69E-11 | Up |
| LINC00992 | 2.8110716 | 9.65E-09 | 4.36E-08 | Up |
| LINC02428 | 6.8572557 | 1.92E-36 | 1.01E-34 | Up |
| AC106771.1 | 6.397344 | 1.29E-07 | 4.94E-07 | Up |
| AC097512.1 | 5.6063246 | 5.85E-07 | 2.03E-06 | Up |
| LINC02065 | 2.1580548 | 0.0010931 | 0.0021916 | Up |
| AC012640.1 | 2.9135069 | 2.36E-15 | 2.46E-14 | Up |
| AC106799.2 | 7.6686511 | 4.50E-29 | 1.44E-27 | Up |
| AL133372.2 | 4.1827042 | 9.16E-08 | 3.59E-07 | Up |
| AC126768.2 | 3.264382 | 2.79E-11 | 1.77E-10 | Up |
| AC093895.1 | 5.4431759 | 1.69E-36 | 8.98E-35 | Up |
| AC093599.1 | 3.9874121 | 6.17E-11 | 3.75E-10 | Up |
| LINC01033 | 3.0275499 | 4.62E-05 | 0.0001169 | Up |
| C5orf66-AS1 | 7.466482 | 7.36E-18 | 9.74E-17 | Up |
| AC027627.1 | 2.37862 | 7.30E-07 | 2.47E-06 | Up |
| AC034223.1 | 4.8628768 | 4.07E-10 | 2.23E-09 | Up |
| AC025183.1 | 7.7792789 | 4.25E-19 | 6.18E-18 | Up |
| TMEM132D-AS1 | 7.4322416 | 9.73E-08 | 3.81E-07 | Up |
| AC026785.3 | 7.3037416 | 2.62E-15 | 2.71E-14 | Up |
| LINC00939 | 2.3492604 | 0.0003612 | 0.000784 | Up |
| AC091868.2 | 3.5084474 | 3.54E-16 | 3.96E-15 | Up |
| AC025183.2 | 4.639624 | 3.83E-09 | 1.85E-08 | Up |
| AC036214.1 | 2.2856068 | 4.66E-09 | 2.21E-08 | Up |
| AC093274.1 | 2.5154427 | 3.79E-05 | 9.74E-05 | Up |
| CASC11 | 3.306807 | 7.22E-09 | 3.33E-08 | Up |
| AL033397.2 | 4.8732409 | 6.66E-24 | 1.46E-22 | Up |
| LINC02071 | 3.1672007 | 5.60E-07 | 1.94E-06 | Up |
| CASC9 | 7.6231844 | 4.08E-49 | 3.94E-47 | Up |
| IL20RB-AS1 | 4.4961917 | 2.77E-12 | 2.04E-11 | Up |
| AC116049.2 | 7.7643546 | 4.89E-38 | 2.84E-36 | Up |
| LINC02477 | 3.3274634 | 2.13E-06 | 6.72E-06 | Up |
| LINC01470 | 2.8800333 | 2.27E-05 | 6.09E-05 | Up |
| AC023886.1 | 3.7812138 | 3.68E-06 | 1.12E-05 | Up |
| LINC01258 | 2.7490941 | 6.18E-05 | 0.000153 | Up |
| LINC01234 | 6.8103014 | 5.19E-18 | 6.98E-17 | Up |
| LINC02465 | 3.549499 | 2.75E-10 | 1.54E-09 | Up |
| LINC00942 | 7.0665153 | 2.12E-12 | 1.59E-11 | Up |
| HOXC13-AS | 7.5659369 | 1.33E-24 | 3.08E-23 | Up |
| AC106772.1 | 3.2949685 | 4.33E-12 | 3.08E-11 | Up |
| AC106795.2 | 2.4269588 | 1.40E-14 | 1.34E-13 | Up |
| LINC02261 | 2.8071678 | 9.61E-05 | 0.0002305 | Up |
| AC126768.3 | 3.5187731 | 6.63E-08 | 2.67E-07 | Up |
| AC112178.1 | 6.1483538 | 3.60E-09 | 1.74E-08 | Up |
| AC004704.1 | 5.6012203 | 1.06E-07 | 4.10E-07 | Up |
| PVT1 | 2.3867617 | 2.57E-28 | 7.70E-27 | Up |
| AC024581.1 | 4.7452809 | 8.98E-25 | 2.11E-23 | Up |
| AC006487.1 | 2.5919886 | 1.76E-07 | 6.60E-07 | Up |
| LINC00536 | 5.0500847 | 8.59E-09 | 3.91E-08 | Up |
| LINC02223 | 4.1528505 | 4.99E-07 | 1.75E-06 | Up |
| SLC7A11-AS1 | 2.3160139 | 2.63E-05 | 6.94E-05 | Up |
| AC096719.1 | 3.077145 | 1.50E-06 | 4.84E-06 | Up |
| AC106895.1 | 4.5554241 | 3.06E-08 | 1.30E-07 | Up |
| AC114316.2 | 3.4110485 | 2.94E-09 | 1.44E-08 | Up |
| LINC02377 | 7.1746706 | 1.14E-07 | 4.39E-07 | Up |
| AC122694.1 | 3.8731911 | 1.86E-07 | 6.94E-07 | Up |
| LINC02232 | 4.5583895 | 8.27E-07 | 2.78E-06 | Up |
| HOXC-AS2 | 5.5434339 | 1.52E-22 | 2.99E-21 | Up |
| AC099509.2 | 2.9465621 | 1.19E-06 | 3.91E-06 | Up |
| AC034206.1 | 4.8939901 | 1.25E-15 | 1.33E-14 | Up |
| AC068647.2 | 4.4942835 | 7.30E-11 | 4.38E-10 | Up |
| AC109439.2 | 3.985157 | 2.01E-05 | 5.44E-05 | Up |
| PURPL | 2.3998995 | 0.0032756 | 0.0059627 | Up |
| AC114296.1 | 3.7997628 | 1.48E-11 | 9.71E-11 | Up |
| AP001790.1 | 3.8327727 | 2.42E-09 | 1.20E-08 | Up |
| LINC02502 | 2.5935228 | 1.62E-06 | 5.20E-06 | Up |
| AC106798.1 | 4.8635801 | 7.82E-18 | 1.03E-16 | Up |
| CLSTN2-AS1 | 2.4474078 | 4.15E-06 | 1.26E-05 | Up |
| HOXC-AS1 | 4.122975 | 3.11E-16 | 3.50E-15 | Up |
| AP002784.1 | 4.8812638 | 4.66E-16 | 5.17E-15 | Up |
| AC079160.1 | 7.7242792 | 4.48E-56 | 6.20E-54 | Up |
| AC109454.3 | 5.8540765 | 4.04E-12 | 2.89E-11 | Up |
| LINC01511 | 4.4666795 | 4.55E-06 | 1.37E-05 | Up |
| LINC00491 | 7.0798949 | 9.53E-36 | 4.82E-34 | Up |
| AC009123.1 | 4.5613985 | 1.34E-25 | 3.36E-24 | Up |
| AC010343.3 | 5.104677 | 4.36E-11 | 2.70E-10 | Up |
| LINC02269 | 3.2330119 | 1.75E-05 | 4.77E-05 | Up |
| AC096759.2 | 5.6177103 | 1.64E-14 | 1.56E-13 | Up |
| AC005865.2 | 2.3996464 | 1.21E-07 | 4.66E-07 | Up |
| LINC02111 | 3.5909032 | 2.65E-06 | 8.27E-06 | Up |
| AC108865.1 | 4.6887332 | 8.45E-06 | 2.43E-05 | Up |
| EPHA5-AS1 | 2.4060918 | 0.0046172 | 0.0081766 | Up |
| AC010595.1 | 7.7233291 | 1.10E-31 | 4.02E-30 | Up |
| LINC02208 | 4.0225315 | 2.77E-08 | 1.18E-07 | Up |
| AL035458.2 | 2.3890423 | 3.19E-13 | 2.66E-12 | Up |
| AC105460.1 | 7.7459314 | 1.92E-08 | 8.33E-08 | Up |
| LINC01181 | 2.1061165 | 7.82E-05 | 0.0001901 | Up |
| LINC00492 | 3.2033169 | 5.61E-10 | 3.01E-09 | Up |
| LINC02382 | 4.1104274 | 1.43E-05 | 3.96E-05 | Up |
| AC020551.1 | 6.2023329 | 5.01E-09 | 2.37E-08 | Up |
| ZFPM2-AS1 | 5.2837805 | 7.47E-18 | 9.88E-17 | Up |
| LINC02163 | 7.1924825 | 6.51E-39 | 4.02E-37 | Up |
| AC104126.1 | 2.54001 | 0.0047146 | 0.0083418 | Up |
| LINC02506 | 3.3345526 | 0.0004715 | 0.0010038 | Up |
| AC090502.1 | 6.1091209 | 4.17E-09 | 2.00E-08 | Up |
| AC113346.1 | 4.2200393 | 4.49E-14 | 4.12E-13 | Up |
| HOXC-AS3 | 6.3511146 | 1.72E-13 | 1.47E-12 | Up |
| AC025244.1 | 5.8390567 | 1.30E-15 | 1.38E-14 | Up |
| AC034223.2 | 5.3407443 | 3.40E-11 | 2.13E-10 | Up |
| AC011352.3 | 4.4767774 | 4.27E-13 | 3.49E-12 | Up |
| LINC02475 | 3.3952208 | 2.19E-06 | 6.90E-06 | Up |
| AC012625.1 | 3.2650039 | 2.09E-12 | 1.57E-11 | Up |
| LINC02315 | 4.0990551 | 3.43E-12 | 2.49E-11 | Up |
| AC122710.2 | 2.6230886 | 7.99E-08 | 3.16E-07 | Up |
| LINC00958 | 5.659528 | 1.48E-25 | 3.71E-24 | Up |
| LINC00605 | 2.0019205 | 0.0007643 | 0.0015712 | Up |
| AC099520.1 | 4.7162132 | 7.85E-10 | 4.12E-09 | Up |
| AC105460.2 | 6.3569801 | 1.17E-11 | 7.80E-11 | Up |
| LINC01385 | 6.0921147 | 8.83E-16 | 9.51E-15 | Up |
| LINC02241 | 4.0143652 | 8.08E-05 | 0.0001957 | Up |
| AC105339.2 | 2.7746688 | 7.77E-33 | 3.07E-31 | Up |
| AC120042.2 | 3.2373961 | 9.10E-10 | 4.75E-09 | Up |
| AC008632.1 | 2.122843 | 0.0003397 | 0.0007413 | Up |
| AC100801.1 | 5.3337545 | 5.32E-06 | 1.58E-05 | Up |
| LINC01605 | 4.6681547 | 7.83E-20 | 1.21E-18 | Up |
| AC009630.2 | 3.0359355 | 3.89E-12 | 2.80E-11 | Up |
| AC138356.3 | 3.6193464 | 1.46E-05 | 4.02E-05 | Up |
| HOXA10-AS | 5.2985182 | 3.66E-19 | 5.38E-18 | Up |
| AC090192.2 | 2.6535606 | 0.0001213 | 0.0002857 | Up |
| AC068228.1 | 5.0387576 | 2.00E-12 | 1.50E-11 | Up |
| AC078906.1 | 2.2347109 | 2.21E-07 | 8.15E-07 | Up |
| AC111149.2 | 3.3538598 | 0.0003542 | 0.0007699 | Up |
| AP003469.1 | 3.0188734 | 7.42E-10 | 3.91E-09 | Up |
| AC084024.3 | 2.4820798 | 2.05E-06 | 6.48E-06 | Up |
| AC008708.1 | 2.2804017 | 0.0041504 | 0.0074156 | Up |
| AC069120.1 | 4.5899337 | 1.42E-08 | 6.25E-08 | Up |
| AC090809.1 | 6.4820891 | 1.24E-07 | 4.78E-07 | Up |
| AC131902.1 | 3.3674017 | 4.98E-05 | 0.0001255 | Up |
| AC021785.1 | 3.6960876 | 7.33E-08 | 2.92E-07 | Up |
| LINC00534 | 2.208209 | 0.0005099 | 0.0010786 | Up |
| AP003469.2 | 2.9463428 | 1.49E-19 | 2.26E-18 | Up |
| AC083973.1 | 4.3292825 | 3.06E-26 | 8.07E-25 | Up |
| AC124067.2 | 3.3054808 | 1.33E-11 | 8.81E-11 | Up |
| LINC02159 | 3.553056 | 9.74E-10 | 5.06E-09 | Up |
| AC022568.1 | 3.1335384 | 0.0009666 | 0.0019561 | Up |
| AC012213.1 | 5.2899959 | 5.75E-19 | 8.29E-18 | Up |
| AF121898.1 | 4.2786551 | 1.85E-07 | 6.89E-07 | Up |
| AC004080.2 | 4.6066877 | 5.85E-14 | 5.28E-13 | Up |
| AC120193.1 | 2.0843919 | 0.0011254 | 0.0022519 | Up |
| AC090568.2 | 3.151035 | 4.85E-10 | 2.63E-09 | Up |
| AC022639.1 | 7.4589568 | 8.84E-22 | 1.60E-20 | Up |
| AC018953.1 | 4.1282511 | 3.52E-08 | 1.48E-07 | Up |
| CERNA3 | 2.0692786 | 7.48E-07 | 2.53E-06 | Up |
| AF279873.3 | 6.7346395 | 5.48E-07 | 1.91E-06 | Up |
| LINC01592 | 3.6973322 | 3.12E-10 | 1.74E-09 | Up |
| AC008464.1 | 4.7131836 | 1.74E-07 | 6.52E-07 | Up |
| ZFHX4-AS1 | 5.9198543 | 2.95E-10 | 1.65E-09 | Up |
| AP000424.1 | 2.8123271 | 1.25E-06 | 4.09E-06 | Up |
| AC011632.1 | 7.8763253 | 4.72E-28 | 1.39E-26 | Up |
| AC022274.1 | 2.2657273 | 7.90E-07 | 2.67E-06 | Up |
| AC083841.2 | 4.718499 | 1.39E-09 | 7.07E-09 | Up |
| AP003469.3 | 2.5936363 | 7.20E-11 | 4.33E-10 | Up |
| AC108002.1 | 2.890142 | 2.24E-17 | 2.85E-16 | Up |
| AC091182.2 | 5.1507765 | 2.15E-18 | 2.96E-17 | Up |
| AC091946.1 | 3.627163 | 2.97E-05 | 7.80E-05 | Up |
| AC008663.1 | 5.892163 | 2.37E-10 | 1.34E-09 | Up |
| AC018616.1 | 4.4681036 | 2.88E-14 | 2.70E-13 | Up |
| FER1L6-AS2 | 6.2367998 | 1.39E-07 | 5.32E-07 | Up |
| LINC01419 | 8.2958949 | 3.04E-08 | 1.29E-07 | Up |
| AC103409.1 | 4.1540473 | 6.14E-05 | 0.0001523 | Up |
| AC011369.1 | 2.8636926 | 1.78E-06 | 5.68E-06 | Up |
| AC105118.1 | 4.8790446 | 5.76E-15 | 5.77E-14 | Up |
| AC007991.3 | 2.1250852 | 0.0042256 | 0.0075364 | Up |
| AC016573.1 | 3.2639048 | 1.96E-10 | 1.12E-09 | Up |
| LINC00051 | 4.8075784 | 1.54E-07 | 5.84E-07 | Up |
| AC092818.1 | 2.2861096 | 0.0001048 | 0.0002501 | Up |
| AC034154.1 | 4.0895229 | 7.32E-08 | 2.92E-07 | Up |
| AC115837.2 | 3.0607993 | 1.35E-06 | 4.37E-06 | Up |
| AP001208.2 | 3.8576438 | 4.88E-10 | 2.64E-09 | Up |
| AC025524.2 | 2.0377124 | 0.0002989 | 0.0006578 | Up |
| CASC19 | 6.6398151 | 4.09E-17 | 5.05E-16 | Up |
| AC100782.1 | 3.7278857 | 2.20E-12 | 1.64E-11 | Up |
| PKIA-AS1 | 2.1102987 | 3.53E-07 | 1.26E-06 | Up |
| AC037486.1 | 4.5522497 | 1.04E-10 | 6.14E-10 | Up |
| AC083967.1 | 3.0246797 | 0.0009731 | 0.0019672 | Up |
| MAFA-AS1 | 5.1018163 | 4.55E-15 | 4.63E-14 | Up |
| MIR2052HG | 5.1567894 | 4.40E-27 | 1.22E-25 | Up |
| LINC02584 | 3.1248421 | 5.65E-05 | 0.0001413 | Up |
| AC022762.1 | 2.4739373 | 1.43E-06 | 4.63E-06 | Up |
| AL136088.1 | 3.7136799 | 1.31E-05 | 3.64E-05 | Up |
| AC044839.2 | 4.6088602 | 2.33E-07 | 8.55E-07 | Up |
| AC105219.2 | 2.6268749 | 4.27E-09 | 2.05E-08 | Up |
| AC080023.1 | 2.5929973 | 5.49E-16 | 6.04E-15 | Up |
| BBOX1-AS1 | 6.7581138 | 3.59E-51 | 3.95E-49 | Up |
| AP001783.1 | 3.7366421 | 2.78E-07 | 1.01E-06 | Up |
| AC027031.2 | 2.4039201 | 9.58E-15 | 9.37E-14 | Up |
| AP003119.1 | 4.5772661 | 1.99E-09 | 9.98E-09 | Up |
| AC018716.1 | 4.1904885 | 1.62E-06 | 5.20E-06 | Up |
| LINC02551 | 2.6333983 | 1.39E-06 | 4.49E-06 | Up |
| AP003390.1 | 4.7436787 | 6.42E-33 | 2.55E-31 | Up |
| AP001360.1 | 5.3274231 | 2.79E-07 | 1.02E-06 | Up |
| AP001547.1 | 4.0268971 | 3.38E-06 | 1.03E-05 | Up |
| AP003063.1 | 4.0092605 | 9.40E-10 | 4.90E-09 | Up |
| AC105219.4 | 2.476306 | 4.40E-21 | 7.53E-20 | Up |
| AC124276.1 | 2.7421407 | 4.19E-08 | 1.73E-07 | Up |
| AF131216.3 | 5.3790172 | 3.97E-13 | 3.26E-12 | Up |
| AC093496.1 | 4.0123753 | 4.07E-05 | 0.000104 | Up |
| AC067930.5 | 2.7727064 | 4.94E-11 | 3.04E-10 | Up |
| GRM5-AS1 | 4.0873444 | 8.70E-08 | 3.42E-07 | Up |
| AP003119.2 | 3.2549805 | 3.35E-25 | 8.26E-24 | Up |
| OVOL1-AS1 | 2.25336 | 7.94E-08 | 3.14E-07 | Up |
| AP000880.1 | 2.3831572 | 1.20E-09 | 6.20E-09 | Up |
| AP001360.2 | 7.8599246 | 5.36E-12 | 3.74E-11 | Up |
| AP003390.2 | 2.502306 | 2.61E-07 | 9.54E-07 | Up |
| AC018716.2 | 5.0053521 | 9.36E-09 | 4.24E-08 | Up |
| SMIM35 | 2.7961739 | 1.81E-11 | 1.18E-10 | Up |
| RRM1-AS1 | 2.3799325 | 6.36E-09 | 2.96E-08 | Up |
| AC108136.1 | 4.0900639 | 2.06E-13 | 1.75E-12 | Up |
| SMILR | 3.5678328 | 3.33E-12 | 2.43E-11 | Up |
| AC120036.4 | 2.0194504 | 1.56E-08 | 6.85E-08 | Up |
| C8orf49 | 2.7844098 | 0.0002882 | 0.0006361 | Up |
| AP003063.2 | 3.0561138 | 1.54E-05 | 4.24E-05 | Up |
| AL137804.1 | 4.309404 | 4.57E-12 | 3.23E-11 | Up |
| MIR9-3HG | 3.4551291 | 1.33E-15 | 1.41E-14 | Up |
| AP000820.1 | 2.0068632 | 0.0005193 | 0.001097 | Up |
| AC008114.1 | 2.1598391 | 9.99E-07 | 3.32E-06 | Up |
| FAM222A-AS1 | 2.4919663 | 1.81E-08 | 7.86E-08 | Up |
| SNHG1 | 2.0712811 | 7.58E-30 | 2.54E-28 | Up |
| AC022509.1 | 4.4394035 | 2.31E-08 | 9.92E-08 | Up |
| AC069503.1 | 2.2526527 | 1.99E-08 | 8.62E-08 | Up |
| AC020611.2 | 2.3219103 | 3.64E-05 | 9.40E-05 | Up |
| AC026310.2 | 2.388652 | 4.28E-06 | 1.29E-05 | Up |
| AC006064.3 | 2.1609987 | 3.34E-12 | 2.44E-11 | Up |
| AP000439.3 | 3.5628791 | 0.0001048 | 0.0002501 | Up |
| AC007848.1 | 3.7572773 | 4.41E-16 | 4.90E-15 | Up |
| AC125616.1 | 4.6088832 | 3.56E-10 | 1.97E-09 | Up |
| AC090673.2 | 3.5652739 | 9.43E-11 | 5.59E-10 | Up |
| LINC02443 | 3.5573019 | 7.39E-14 | 6.59E-13 | Up |
| LINC01152 | 2.0571219 | 0.0001495 | 0.0003461 | Up |
| AC006206.1 | 3.2421523 | 5.39E-09 | 2.54E-08 | Up |
| AC022075.2 | 3.6840575 | 3.97E-13 | 3.26E-12 | Up |
| AP003721.1 | 3.4685445 | 7.01E-18 | 9.33E-17 | Up |
| AC007848.2 | 4.3776583 | 1.57E-12 | 1.20E-11 | Up |
| LINC02387 | 2.8961488 | 1.64E-07 | 6.19E-07 | Up |
| LINC02454 | 3.0300357 | 6.76E-07 | 2.32E-06 | Up |
| AC087258.1 | 6.0937952 | 1.40E-09 | 7.14E-09 | Up |
| AC006206.2 | 4.2877738 | 1.98E-25 | 4.91E-24 | Up |
| AP003559.1 | 2.7962778 | 2.59E-12 | 1.92E-11 | Up |
| AC148477.2 | 3.7150302 | 5.02E-06 | 1.49E-05 | Up |
| LINC02393 | 6.2100307 | 6.65E-08 | 2.67E-07 | Up |
| AC005906.2 | 3.7354904 | 7.91E-05 | 0.000192 | Up |
| AC087318.1 | 2.2039563 | 4.86E-06 | 1.45E-05 | Up |
| AP000721.2 | 2.115516 | 0.0001165 | 0.0002753 | Up |
| AP000851.1 | 4.0255465 | 2.10E-06 | 6.64E-06 | Up |
| AC084819.1 | 4.6222686 | 1.04E-07 | 4.05E-07 | Up |
| AP001453.2 | 3.767867 | 2.67E-21 | 4.65E-20 | Up |
| AC131009.2 | 4.1022208 | 3.03E-12 | 2.22E-11 | Up |
| AC135782.1 | 2.5311977 | 7.38E-05 | 0.0001802 | Up |
| AC084816.1 | 5.6192873 | 8.86E-12 | 6.02E-11 | Up |
| AC008115.2 | 3.4231233 | 8.86E-09 | 4.03E-08 | Up |
| AC008011.2 | 3.2492391 | 6.13E-11 | 3.73E-10 | Up |
| TMPO-AS1 | 2.3814088 | 2.38E-37 | 1.31E-35 | Up |
| LINC02293 | 3.8143574 | 2.78E-05 | 7.32E-05 | Up |
| AC090709.1 | 3.4529151 | 1.60E-14 | 1.53E-13 | Up |
| AC131157.1 | 6.7400757 | 2.80E-12 | 2.06E-11 | Up |
| AC023511.1 | 4.9004193 | 1.51E-08 | 6.65E-08 | Up |
| AC123905.1 | 3.2562062 | 7.84E-09 | 3.60E-08 | Up |
| KIRREL3-AS1 | 4.1390863 | 9.66E-06 | 2.75E-05 | Up |
| AC126177.4 | 2.3713235 | 0.0002108 | 0.0004767 | Up |
| AC068305.2 | 2.7446747 | 2.67E-09 | 1.31E-08 | Up |
| AC025575.1 | 3.3508294 | 4.59E-10 | 2.50E-09 | Up |
| AC055736.1 | 4.9563938 | 3.79E-09 | 1.83E-08 | Up |
| AC011601.1 | 3.851673 | 8.89E-09 | 4.04E-08 | Up |
| LINC01475 | 3.4968432 | 4.77E-05 | 0.0001207 | Up |
| AC078778.1 | 2.5969021 | 6.64E-23 | 1.35E-21 | Up |
| G2E3-AS1 | 8.6142621 | 9.17E-33 | 3.61E-31 | Up |
| AC089983.1 | 3.532518 | 3.63E-07 | 1.30E-06 | Up |
| AC078860.1 | 3.7729004 | 1.64E-09 | 8.27E-09 | Up |
| LINC02588 | 4.9311732 | 1.25E-08 | 5.57E-08 | Up |
| AL139023.1 | 7.281728 | 2.72E-18 | 3.73E-17 | Up |
| LINC02404 | 7.7922196 | 7.17E-08 | 2.86E-07 | Up |
| LINC02156 | 3.4199073 | 4.51E-20 | 7.18E-19 | Up |
| AC008083.1 | 3.3425169 | 6.66E-08 | 2.67E-07 | Up |
| LINC02457 | 6.3320213 | 3.72E-14 | 3.44E-13 | Up |
| AC025575.2 | 6.4635062 | 4.00E-24 | 9.02E-23 | Up |
| AC078923.1 | 2.8572603 | 3.30E-07 | 1.19E-06 | Up |
| AC078820.1 | 2.3620059 | 1.36E-05 | 3.79E-05 | Up |
| AC005841.1 | 2.1362226 | 8.56E-09 | 3.90E-08 | Up |
| AC089987.2 | 3.2521362 | 1.12E-08 | 5.03E-08 | Up |
| LINC02444 | 4.8646568 | 2.33E-08 | 9.99E-08 | Up |
| AC002351.1 | 6.0772971 | 1.97E-09 | 9.87E-09 | Up |
| LINC00592 | 3.1311746 | 4.48E-15 | 4.56E-14 | Up |
| AC090503.2 | 4.1095018 | 1.39E-07 | 5.32E-07 | Up |
| AL589182.1 | 6.0252662 | 2.20E-09 | 1.10E-08 | Up |
| AC068831.1 | 2.1366105 | 8.18E-28 | 2.40E-26 | Up |
| AL355922.2 | 2.0401092 | 2.46E-16 | 2.81E-15 | Up |
| LINC02313 | 2.835252 | 5.94E-07 | 2.06E-06 | Up |
| LINC00640 | 3.7226681 | 7.69E-19 | 1.10E-17 | Up |
| AL049830.3 | 2.4709209 | 2.56E-08 | 1.10E-07 | Up |
| AL079307.1 | 3.9757632 | 3.85E-10 | 2.11E-09 | Up |
| LINC00645 | 4.7026545 | 1.10E-14 | 1.06E-13 | Up |
| AL136298.1 | 2.8376357 | 2.00E-05 | 5.41E-05 | Up |
| AL391152.1 | 4.1910001 | 6.46E-29 | 2.03E-27 | Up |
| LINC01629 | 4.1082932 | 1.97E-08 | 8.56E-08 | Up |
| AC026495.1 | 2.2881488 | 3.91E-05 | 0.0001003 | Up |
| AL136018.1 | 5.1277083 | 5.82E-11 | 3.55E-10 | Up |
| AL079303.1 | 3.531509 | 1.12E-11 | 7.53E-11 | Up |
| AL049874.3 | 3.5281363 | 1.69E-06 | 5.43E-06 | Up |
| LINC01269 | 2.0294457 | 1.06E-05 | 2.98E-05 | Up |
| LINC00871 | 5.4388758 | 7.66E-12 | 5.25E-11 | Up |
| AL358334.2 | 4.5028216 | 9.81E-19 | 1.39E-17 | Up |
| LINC02301 | 4.3293934 | 5.88E-06 | 1.73E-05 | Up |
| AC122685.1 | 6.6647695 | 6.89E-12 | 4.74E-11 | Up |
| LINC00520 | 2.9837309 | 3.45E-07 | 1.24E-06 | Up |
| AL133153.2 | 2.2008316 | 5.83E-09 | 2.72E-08 | Up |
| LINC02310 | 4.7309657 | 1.70E-10 | 9.76E-10 | Up |
| AC131532.1 | 7.2505371 | 6.34E-31 | 2.24E-29 | Up |
| AL133370.1 | 6.2539244 | 6.32E-08 | 2.55E-07 | Up |
| LINC02321 | 2.7445643 | 1.11E-12 | 8.61E-12 | Up |
| AL049775.2 | 3.462592 | 1.26E-06 | 4.13E-06 | Up |
| LINC01956 | 5.4536079 | 1.46E-11 | 9.57E-11 | Up |
| AL132712.2 | 2.1113856 | 1.78E-16 | 2.06E-15 | Up |
| AL133467.2 | 4.2522301 | 1.38E-06 | 4.47E-06 | Up |
| AL358332.1 | 3.2820747 | 2.06E-14 | 1.95E-13 | Up |
| SALRNA1 | 2.4726272 | 2.56E-12 | 1.89E-11 | Up |
| LINC00519 | 5.8340455 | 3.74E-26 | 9.76E-25 | Up |
| AC244502.3 | 2.7135852 | 3.87E-06 | 1.18E-05 | Up |
| LINC00648 | 3.4379647 | 6.43E-06 | 1.88E-05 | Up |
| AL161757.5 | 3.5139089 | 1.31E-05 | 3.64E-05 | Up |
| AC004816.1 | 2.3364711 | 2.70E-16 | 3.07E-15 | Up |
| AC023906.2 | 7.1231498 | 1.89E-25 | 4.70E-24 | Up |
| LINC00928 | 4.901979 | 1.02E-14 | 9.95E-14 | Up |
| LINC02323 | 3.3225432 | 4.17E-14 | 3.84E-13 | Up |
| MIR4713HG | 5.2702976 | 1.27E-35 | 6.24E-34 | Up |
| AC022405.1 | 2.0964274 | 5.90E-06 | 1.73E-05 | Up |
| AC109462.1 | 3.5703223 | 2.72E-09 | 1.34E-08 | Up |
| AC020891.2 | 6.6561187 | 6.02E-34 | 2.54E-32 | Up |
| AC015660.1 | 3.1195772 | 7.66E-10 | 4.03E-09 | Up |
| AC013652.1 | 3.7069611 | 1.80E-35 | 8.67E-34 | Up |
| AC087473.1 | 3.9462507 | 4.85E-09 | 2.30E-08 | Up |
| AC118658.1 | 2.2555314 | 6.42E-10 | 3.42E-09 | Up |
| LINC01833 | 6.8729642 | 1.34E-12 | 1.03E-11 | Up |
| AC025219.1 | 2.3766403 | 2.92E-12 | 2.14E-11 | Up |
| AC013652.2 | 2.2181785 | 6.08E-07 | 2.10E-06 | Up |
| LINC02253 | 6.1929589 | 7.71E-16 | 8.31E-15 | Up |
| AC027243.1 | 3.0478819 | 2.23E-10 | 1.27E-09 | Up |
| LINC01583 | 2.9556689 | 1.05E-07 | 4.07E-07 | Up |
| AC067863.1 | 3.9588654 | 5.00E-13 | 4.05E-12 | Up |
| LINC01491 | 5.0676766 | 4.68E-07 | 1.65E-06 | Up |
| AC023034.1 | 3.3588076 | 1.30E-19 | 1.98E-18 | Up |
| AC015712.4 | 2.1710618 | 0.0011604 | 0.0023162 | Up |
| AC023034.2 | 3.5967861 | 2.23E-15 | 2.33E-14 | Up |
| AC010478.1 | 2.3635229 | 0.0005569 | 0.00117 | Up |
| LINC02254 | 3.5074628 | 4.07E-08 | 1.69E-07 | Up |
| AC087612.1 | 6.3931244 | 1.00E-07 | 3.91E-07 | Up |
| IDH2-DT | 2.4579053 | 0.0007767 | 0.0015956 | Up |
| AC104041.1 | 4.468076 | 8.46E-24 | 1.83E-22 | Up |
| AC020891.3 | 4.0426738 | 3.51E-17 | 4.39E-16 | Up |
| AL354993.2 | 3.8911405 | 2.68E-32 | 1.02E-30 | Up |
| AC103740.2 | 3.7042897 | 8.16E-11 | 4.88E-10 | Up |
| AC100839.2 | 4.0651347 | 4.47E-06 | 1.35E-05 | Up |
| AC007907.1 | 3.3299683 | 5.99E-08 | 2.43E-07 | Up |
| LINC02109 | 6.7341615 | 4.44E-16 | 4.94E-15 | Up |
| AC012640.2 | 2.1671565 | 1.23E-16 | 1.45E-15 | Up |
| AL355596.1 | 2.9570171 | 1.00E-07 | 3.92E-07 | Up |
| AL163952.1 | 4.7227411 | 4.22E-11 | 2.61E-10 | Up |
| AL121578.3 | 4.5472884 | 5.58E-07 | 1.94E-06 | Up |
| AC017100.1 | 2.2572432 | 1.28E-14 | 1.23E-13 | Up |
| LINC01992 | 6.8164093 | 1.09E-10 | 6.40E-10 | Up |
| AC112176.1 | 4.9984954 | 3.92E-09 | 1.89E-08 | Up |
| AC012174.1 | 3.1663818 | 5.78E-09 | 2.70E-08 | Up |
| LINC00556 | 3.2122407 | 8.42E-07 | 2.83E-06 | Up |
| AL032819.1 | 2.3753069 | 5.51E-09 | 2.59E-08 | Up |
| AC126696.1 | 2.1159127 | 0.0001672 | 0.0003838 | Up |
| LINC02137 | 3.8635671 | 4.31E-11 | 2.66E-10 | Up |
| AP000842.3 | 2.7104815 | 4.14E-07 | 1.47E-06 | Up |
| AC000032.1 | 3.9859295 | 2.35E-05 | 6.26E-05 | Up |
| AC124944.3 | 3.0776596 | 6.56E-27 | 1.81E-25 | Up |
| LINC02562 | 3.6348356 | 1.61E-23 | 3.42E-22 | Up |
| AC093515.1 | 5.0496472 | 5.44E-07 | 1.89E-06 | Up |
| AC083801.2 | 7.0441271 | 4.93E-20 | 7.82E-19 | Up |
| AL353746.1 | 2.8127828 | 2.42E-05 | 6.43E-05 | Up |
| ATP2A1-AS1 | 2.2501241 | 5.12E-15 | 5.19E-14 | Up |
| AL355607.2 | 3.8726628 | 3.16E-09 | 1.54E-08 | Up |
| C15orf59-AS1 | 3.5293085 | 3.50E-07 | 1.26E-06 | Up |
| AC104794.3 | 5.896404 | 3.65E-15 | 3.76E-14 | Up |
| AC131902.2 | 2.2275972 | 0.0023791 | 0.0044675 | Up |
| AC009148.1 | 2.2751635 | 2.36E-18 | 3.25E-17 | Up |
| AC126696.3 | 2.3978003 | 4.07E-08 | 1.69E-07 | Up |
| AC011374.1 | 5.3641815 | 5.40E-22 | 1.01E-20 | Up |
| AL359851.1 | 3.5358536 | 3.07E-08 | 1.30E-07 | Up |
| AC009097.2 | 3.3840525 | 5.37E-19 | 7.78E-18 | Up |
| AC012531.1 | 4.1340702 | 1.63E-15 | 1.72E-14 | Up |
| AC092125.1 | 2.8711667 | 1.02E-06 | 3.40E-06 | Up |
| AC020658.3 | 2.6060313 | 1.52E-10 | 8.85E-10 | Up |
| AC138305.1 | 5.8342355 | 1.03E-19 | 1.57E-18 | Up |
| AC004158.1 | 3.2156935 | 1.59E-06 | 5.10E-06 | Up |
| AL445531.1 | 5.7260843 | 1.09E-24 | 2.54E-23 | Up |
| AC120498.4 | 4.6396105 | 1.34E-09 | 6.87E-09 | Up |
| AC093520.1 | 2.8842331 | 3.71E-12 | 2.69E-11 | Up |
| AC106799.3 | 6.2180016 | 4.48E-19 | 6.51E-18 | Up |
| AC061975.1 | 2.4969761 | 2.85E-05 | 7.49E-05 | Up |
| LINC01964 | 4.9398487 | 3.99E-07 | 1.42E-06 | Up |
| AC099506.1 | 2.3635485 | 2.27E-05 | 6.08E-05 | Up |
| AP005233.2 | 6.2033778 | 4.32E-11 | 2.67E-10 | Up |
| LINC02170 | 2.2450775 | 7.65E-05 | 0.0001862 | Up |
| AC015818.2 | 4.79713 | 9.10E-14 | 8.07E-13 | Up |
| LINC01254 | 3.2566428 | 3.10E-05 | 8.09E-05 | Up |
| AC009139.1 | 3.4466204 | 1.40E-06 | 4.51E-06 | Up |
| LINC01416 | 3.3791325 | 3.04E-11 | 1.91E-10 | Up |
| FOXC2-AS1 | 2.4187597 | 0.0003996 | 0.000861 | Up |
| AC023824.3 | 6.224941 | 1.11E-07 | 4.30E-07 | Up |
| LINC00557 | 4.3242517 | 1.05E-08 | 4.72E-08 | Up |
| LINC01633 | 6.7638326 | 5.00E-35 | 2.33E-33 | Up |
| AC126407.1 | 3.4715558 | 1.53E-09 | 7.73E-09 | Up |
| LINC01572 | 3.2708237 | 8.76E-55 | 1.12E-52 | Up |
| LINC02544 | 2.8296241 | 1.07E-10 | 6.29E-10 | Up |
| LINC01228 | 3.2277455 | 0.0006924 | 0.0014349 | Up |
| LINC02178 | 6.9665413 | 8.31E-11 | 4.96E-10 | Up |
| AC093904.4 | 3.4353624 | 4.59E-11 | 2.83E-10 | Up |
| AL049555.1 | 6.7079676 | 4.09E-61 | 6.70E-59 | Up |
| LINC02473 | 4.9297923 | 3.44E-13 | 2.85E-12 | Up |
| LINC02167 | 9.1082828 | 1.81E-07 | 6.75E-07 | Up |
| AC009065.5 | 2.1181453 | 5.75E-09 | 2.69E-08 | Up |
| LINC01989 | 2.700087 | 0.0014683 | 0.0028736 | Up |
| AC112484.3 | 2.55835 | 2.61E-13 | 2.20E-12 | Up |
| LINC02188 | 2.4853893 | 2.35E-05 | 6.27E-05 | Up |
| AL031058.1 | 3.1768243 | 1.14E-30 | 3.99E-29 | Up |
| AC092142.1 | 2.7325191 | 4.46E-13 | 3.63E-12 | Up |
| LINC02128 | 3.5753485 | 1.91E-06 | 6.09E-06 | Up |
| AC008870.3 | 2.0734846 | 5.05E-09 | 2.38E-08 | Up |
| AC112236.1 | 5.1174746 | 1.37E-14 | 1.32E-13 | Up |
| AC106779.1 | 2.2788549 | 4.65E-11 | 2.86E-10 | Up |
| AC009081.1 | 5.3964414 | 3.62E-10 | 2.00E-09 | Up |
| AC009061.1 | 2.5302177 | 7.84E-07 | 2.65E-06 | Up |
| AC134312.5 | 2.2580861 | 5.73E-09 | 2.69E-08 | Up |
| AC010491.1 | 2.3046198 | 1.22E-17 | 1.59E-16 | Up |
| VPS9D1-AS1 | 3.6343789 | 6.15E-32 | 2.30E-30 | Up |
| AC010735.1 | 2.2309912 | 4.14E-05 | 0.0001057 | Up |
| AL591222.1 | 3.2654196 | 1.55E-07 | 5.89E-07 | Up |
| AL035425.3 | 5.5188806 | 6.07E-08 | 2.46E-07 | Up |
| AC021087.4 | 3.0052407 | 5.78E-09 | 2.70E-08 | Up |
| AC013391.3 | 4.590821 | 1.06E-15 | 1.13E-14 | Up |
| AC040174.1 | 6.2238517 | 1.63E-21 | 2.90E-20 | Up |
| DLGAP1-AS5 | 2.2551661 | 0.0026081 | 0.0048579 | Up |
| AP003119.3 | 2.802715 | 4.14E-19 | 6.03E-18 | Up |
| SSTR5-AS1 | 3.4002346 | 8.59E-06 | 2.46E-05 | Up |
| AL136537.2 | 5.0870761 | 8.45E-05 | 0.0002041 | Up |
| AC027228.2 | 3.2128524 | 7.18E-27 | 1.97E-25 | Up |
| AC244090.3 | 2.0018074 | 1.21E-06 | 3.96E-06 | Up |
| LINC02582 | 5.5752392 | 6.69E-06 | 1.95E-05 | Up |
| AC007342.4 | 2.0050361 | 8.82E-07 | 2.96E-06 | Up |
| LINC02141 | 5.5423878 | 1.42E-09 | 7.23E-09 | Up |
| AC090826.1 | 2.5407659 | 4.48E-05 | 0.0001136 | Up |
| AC090282.1 | 4.9248572 | 4.48E-15 | 4.56E-14 | Up |
| SMIM36 | 3.607916 | 2.42E-09 | 1.20E-08 | Up |
| AC005670.1 | 2.1849181 | 1.07E-08 | 4.81E-08 | Up |
| AC092115.3 | 2.8864862 | 8.77E-13 | 6.88E-12 | Up |
| AC116025.2 | 2.3573767 | 1.73E-07 | 6.50E-07 | Up |
| AL157931.1 | 4.217531 | 6.77E-12 | 4.67E-11 | Up |
| AC004584.1 | 2.1880118 | 5.21E-15 | 5.27E-14 | Up |
| AC004034.1 | 2.5944375 | 2.39E-11 | 1.53E-10 | Up |
| AJ003147.1 | 3.2433886 | 1.71E-05 | 4.68E-05 | Up |
| LINC01979 | 2.6150264 | 2.39E-07 | 8.75E-07 | Up |
| AJ003147.2 | 3.406463 | 8.02E-06 | 2.31E-05 | Up |
| AC005722.2 | 3.9597818 | 3.53E-12 | 2.57E-11 | Up |
| AC009121.1 | 3.2195836 | 1.62E-23 | 3.43E-22 | Up |
| AC100791.2 | 4.1076919 | 8.15E-13 | 6.41E-12 | Up |
| LINC01977 | 4.1941514 | 1.30E-23 | 2.78E-22 | Up |
| AC005695.1 | 2.0982952 | 4.23E-08 | 1.75E-07 | Up |
| LINC01896 | 6.4943659 | 1.52E-07 | 5.76E-07 | Up |
| TMEM238L | 2.7026259 | 0.0010439 | 0.0021014 | Up |
| AC100872.1 | 2.4265062 | 0.0003663 | 0.000794 | Up |
| AP005328.1 | 4.5219843 | 1.70E-13 | 1.46E-12 | Up |
| AC090125.1 | 2.4752182 | 4.95E-06 | 1.48E-05 | Up |
| AC007639.1 | 2.9678392 | 2.43E-16 | 2.78E-15 | Up |
| AC079062.1 | 7.8896587 | 8.20E-11 | 4.90E-10 | Up |
| AP001178.1 | 2.0864354 | 3.24E-07 | 1.17E-06 | Up |
| AP005230.1 | 6.390668 | 1.27E-35 | 6.24E-34 | Up |
| AC090371.2 | 3.283465 | 2.00E-07 | 7.41E-07 | Up |
| LINC01543 | 3.4957688 | 2.26E-05 | 6.07E-05 | Up |
| AC080037.1 | 4.3164386 | 4.46E-18 | 6.04E-17 | Up |
| AC104564.2 | 2.5843104 | 1.63E-09 | 8.20E-09 | Up |
| AC124254.1 | 2.5742565 | 0.0001165 | 0.0002752 | Up |
| LINC02003 | 3.6822614 | 2.46E-12 | 1.83E-11 | Up |
| AC005291.1 | 3.6843776 | 1.86E-08 | 8.11E-08 | Up |
| AC090403.1 | 2.8568463 | 3.41E-05 | 8.84E-05 | Up |
| AC084346.2 | 2.0803379 | 2.52E-09 | 1.24E-08 | Up |
| AC025211.1 | 2.8418493 | 8.34E-12 | 5.69E-11 | Up |
| AC091170.1 | 4.616364 | 1.10E-06 | 3.62E-06 | Up |
| AC104564.4 | 3.0232243 | 9.98E-07 | 3.32E-06 | Up |
| AC145207.8 | 2.4972203 | 2.73E-08 | 1.16E-07 | Up |
| AC005722.3 | 4.7655765 | 1.78E-08 | 7.73E-08 | Up |
| AC068025.1 | 2.488001 | 3.01E-10 | 1.68E-09 | Up |
| AC004147.4 | 2.1943707 | 0.0004472 | 0.0009548 | Up |
| AC099850.3 | 2.9776418 | 5.25E-38 | 3.03E-36 | Up |
| AC012447.1 | 2.1418548 | 6.13E-10 | 3.27E-09 | Up |
| AC023301.1 | 3.3105271 | 6.96E-11 | 4.20E-10 | Up |
| LINC00668 | 7.3410488 | 3.46E-26 | 9.10E-25 | Up |
| AC090912.1 | 2.4049346 | 9.95E-14 | 8.79E-13 | Up |
| LINC01387 | 2.0556078 | 0.0044569 | 0.0079102 | Up |
| GACAT2 | 4.4432799 | 5.10E-14 | 4.63E-13 | Up |
| ESRG | 6.9679776 | 3.75E-08 | 1.57E-07 | Up |
| AC016888.1 | 2.705142 | 2.94E-17 | 3.70E-16 | Up |
| AP001011.1 | 2.5709714 | 0.0003098 | 0.0006808 | Up |
| AP005203.1 | 2.8297867 | 1.39E-05 | 3.85E-05 | Up |
| LINC01910 | 2.8477706 | 2.06E-06 | 6.53E-06 | Up |
| LIVAR | 2.3718957 | 3.19E-06 | 9.81E-06 | Up |
| AP002478.1 | 3.7128683 | 1.63E-10 | 9.44E-10 | Up |
| MAGEA10-MAGEA5 | 5.3297239 | 4.25E-14 | 3.91E-13 | Up |
| AP001025.1 | 3.9910675 | 4.00E-07 | 1.42E-06 | Up |
| AC008109.1 | 4.8805242 | 7.08E-08 | 2.83E-07 | Up |
| DSG1-AS1 | 7.4845679 | 7.06E-26 | 1.80E-24 | Up |
| AC061975.6 | 6.8964422 | 1.59E-09 | 8.01E-09 | Up |
| AC090912.2 | 2.142874 | 7.52E-09 | 3.46E-08 | Up |
| AC005993.1 | 4.9995843 | 1.79E-20 | 2.97E-19 | Up |
| AC023421.1 | 2.4094933 | 0.0022872 | 0.0043142 | Up |
| TCF4-AS1 | 3.2124427 | 1.52E-07 | 5.76E-07 | Up |
| AC010980.2 | 2.8600873 | 4.15E-13 | 3.39E-12 | Up |
| LINC01905 | 4.1872519 | 6.99E-15 | 6.95E-14 | Up |
| AC005256.1 | 5.1552691 | 8.39E-11 | 5.00E-10 | Up |
| LINC02081 | 2.3375461 | 2.52E-08 | 1.08E-07 | Up |
| LINC01842 | 4.0070296 | 3.73E-11 | 2.33E-10 | Up |
| MIR2117HG | 5.6250063 | 6.56E-20 | 1.03E-18 | Up |
| AC022031.1 | 5.7824461 | 1.99E-20 | 3.28E-19 | Up |
| LINC01775 | 2.2655881 | 1.87E-11 | 1.22E-10 | Up |
| AC005381.1 | 3.0099888 | 0.0021054 | 0.0039909 | Up |
| AP005264.3 | 2.0502654 | 6.86E-05 | 0.0001685 | Up |
| AC011498.3 | 3.1248667 | 7.87E-15 | 7.79E-14 | Up |
| AC020928.2 | 2.6877433 | 1.01E-06 | 3.37E-06 | Up |
| AC022031.2 | 7.2815801 | 1.77E-39 | 1.12E-37 | Up |
| AC068473.3 | 3.1233236 | 1.38E-08 | 6.07E-08 | Up |
| AC008649.1 | 4.7266291 | 2.69E-12 | 1.99E-11 | Up |
| AC021504.1 | 5.2586705 | 1.58E-23 | 3.36E-22 | Up |
| AC009271.1 | 5.0373958 | 7.07E-21 | 1.20E-19 | Up |
| AC005330.1 | 3.1841437 | 1.60E-10 | 9.27E-10 | Up |
| AC016205.1 | 4.9064127 | 1.38E-30 | 4.76E-29 | Up |
| AC068473.4 | 3.2035526 | 2.03E-07 | 7.54E-07 | Up |
| LINC01901 | 5.6376524 | 3.75E-15 | 3.86E-14 | Up |
| AC020934.1 | 3.4024117 | 1.08E-06 | 3.55E-06 | Up |
| AC021683.2 | 2.2406808 | 0.0003966 | 0.0008551 | Up |
| AC010327.3 | 2.1016629 | 0.0002908 | 0.0006415 | Up |
| AC021683.3 | 5.189876 | 3.27E-09 | 1.59E-08 | Up |
| AC002116.2 | 2.0604833 | 1.41E-16 | 1.65E-15 | Up |
| AC024592.2 | 2.2425781 | 1.05E-06 | 3.49E-06 | Up |
| RUNDC3A-AS1 | 2.9728003 | 2.43E-16 | 2.79E-15 | Up |
| AC004221.1 | 3.2579618 | 3.90E-13 | 3.21E-12 | Up |
| AC011483.1 | 2.8834104 | 0.0001009 | 0.0002413 | Up |
| AC008750.3 | 2.6100298 | 5.50E-07 | 1.91E-06 | Up |
| AC009955.2 | 4.2189074 | 5.33E-13 | 4.29E-12 | Up |
| AC007785.1 | 3.5057466 | 9.37E-09 | 4.24E-08 | Up |
| AC010328.1 | 2.1549796 | 0.0004127 | 0.0008858 | Up |
| AC010605.1 | 2.5895529 | 2.43E-09 | 1.20E-08 | Up |
| FMR1-AS1 | 2.5385764 | 8.40E-11 | 5.00E-10 | Up |
| AC008687.2 | 2.4615871 | 6.67E-10 | 3.54E-09 | Up |
| AC008392.1 | 2.0552088 | 0.0017052 | 0.0032906 | Up |
| LINC02560 | 2.1458369 | 1.27E-10 | 7.45E-10 | Up |
| AL132655.1 | 2.5919889 | 3.69E-06 | 1.12E-05 | Up |
| AC006262.1 | 3.9401771 | 4.75E-17 | 5.85E-16 | Up |
| IGFL2-AS1 | 4.3708938 | 6.42E-10 | 3.42E-09 | Up |
| AL121761.2 | 2.6208462 | 6.26E-08 | 2.53E-07 | Up |
| AP003680.1 | 2.8149802 | 7.12E-18 | 9.46E-17 | Up |
| AL132655.2 | 2.2644496 | 1.09E-06 | 3.61E-06 | Up |
| LINC01711 | 3.944019 | 1.83E-16 | 2.13E-15 | Up |
| AL356740.3 | 2.8829706 | 1.79E-10 | 1.03E-09 | Up |
| LINC01224 | 3.4663156 | 9.30E-07 | 3.10E-06 | Up |
| AC005197.1 | 2.5429351 | 6.61E-07 | 2.27E-06 | Up |
| AC006262.2 | 3.2303805 | 1.82E-06 | 5.81E-06 | Up |
| AC011453.1 | 2.7453388 | 0.0016676 | 0.0032251 | Up |
| AL136172.1 | 2.4122113 | 1.38E-11 | 9.14E-11 | Up |
| AC078802.1 | 2.2127252 | 1.69E-12 | 1.29E-11 | Up |
| AC004817.4 | 2.9556263 | 1.47E-05 | 4.06E-05 | Up |
| AC091057.4 | 2.5352726 | 7.85E-21 | 1.32E-19 | Up |
| AC040174.2 | 4.5992571 | 4.75E-14 | 4.34E-13 | Up |
| AC036176.3 | 5.5749027 | 7.05E-22 | 1.30E-20 | Up |
| KC877982.1 | 7.3684349 | 4.51E-12 | 3.20E-11 | Up |
| AL513318.2 | 4.6833973 | 1.01E-19 | 1.55E-18 | Up |
| AC110769.2 | 2.0360204 | 2.27E-15 | 2.37E-14 | Up |
| AC027243.2 | 3.6609585 | 5.26E-13 | 4.24E-12 | Up |
| AL133467.4 | 4.110209 | 1.26E-06 | 4.11E-06 | Up |
| AC125494.3 | 2.1801965 | 7.61E-05 | 0.0001852 | Up |
| AC004080.4 | 2.7888073 | 1.27E-07 | 4.90E-07 | Up |
| AL162413.1 | 3.5923923 | 4.67E-07 | 1.65E-06 | Up |
| AC106900.2 | 5.685244 | 1.56E-41 | 1.05E-39 | Up |
| AC013731.1 | 2.5044176 | 3.12E-22 | 5.97E-21 | Up |
| LINC00221 | 7.0355111 | 1.44E-05 | 3.98E-05 | Up |
| AC108451.2 | 2.2220868 | 4.08E-07 | 1.45E-06 | Up |
| AC010719.1 | 2.4970852 | 5.45E-17 | 6.65E-16 | Up |
| AL033381.2 | 2.2242066 | 0.0003786 | 0.0008181 | Up |
| AC015849.5 | 3.5768889 | 4.38E-17 | 5.40E-16 | Up |
| AP000526.1 | 4.2159161 | 4.52E-16 | 5.01E-15 | Up |
| AC016737.1 | 2.4016017 | 4.57E-20 | 7.27E-19 | Up |
| AC090578.1 | 3.7614888 | 2.03E-10 | 1.15E-09 | Up |
| AL683887.1 | 6.0816803 | 5.33E-17 | 6.54E-16 | Up |
| AL359504.2 | 2.2566712 | 8.37E-19 | 1.19E-17 | Up |
| AL161729.4 | 2.1101989 | 1.21E-10 | 7.11E-10 | Up |
| AC010501.1 | 2.3716059 | 3.06E-08 | 1.30E-07 | Up |
| AC011337.1 | 2.254956 | 5.57E-16 | 6.12E-15 | Up |
| SMIM32 | 2.7451401 | 2.00E-05 | 5.41E-05 | Up |
| AC019080.3 | 2.4972812 | 2.69E-08 | 1.15E-07 | Up |
| AC012213.4 | 5.7772971 | 2.01E-19 | 3.01E-18 | Up |
| AL589740.1 | 4.2811738 | 4.82E-08 | 1.98E-07 | Up |
| AL136162.1 | 2.5425451 | 5.15E-22 | 9.63E-21 | Up |
| AC016877.3 | 2.7024715 | 4.82E-10 | 2.61E-09 | Up |
| U47924.1 | 2.1886085 | 5.13E-11 | 3.14E-10 | Up |
| AC019080.4 | 5.1318467 | 5.39E-12 | 3.76E-11 | Up |
| AL445647.1 | 6.0117911 | 2.26E-07 | 8.32E-07 | Up |
| AL365181.2 | 4.3593785 | 7.39E-10 | 3.90E-09 | Up |
| AC122710.3 | 3.7950583 | 1.01E-12 | 7.90E-12 | Up |
| AP006545.2 | 2.9596498 | 1.93E-13 | 1.64E-12 | Up |
| AL390719.2 | 2.2388985 | 5.05E-13 | 4.08E-12 | Up |
| AF106564.1 | 3.6711904 | 2.40E-05 | 6.39E-05 | Up |
| AC097358.2 | 3.6976384 | 5.26E-22 | 9.81E-21 | Up |
| AL451050.2 | 2.15827 | 1.50E-13 | 1.29E-12 | Up |
| AL356277.3 | 3.4853745 | 9.43E-06 | 2.69E-05 | Up |
| AL133255.1 | 2.1778457 | 2.08E-06 | 6.56E-06 | Up |
| AC007128.2 | 6.9496869 | 2.28E-46 | 1.99E-44 | Up |
| AC116609.3 | 2.5480118 | 0.001951 | 0.0037229 | Up |
| AL160408.5 | 4.1519209 | 1.20E-10 | 7.03E-10 | Up |
| AL365181.3 | 3.7942573 | 1.99E-10 | 1.13E-09 | Up |
| AL645608.7 | 2.4266748 | 4.12E-07 | 1.46E-06 | Up |
| AC113194.1 | 3.6227926 | 7.93E-09 | 3.64E-08 | Up |
| AP005328.2 | 2.9638791 | 4.44E-07 | 1.57E-06 | Up |
| AC017048.3 | 2.7000997 | 6.83E-07 | 2.34E-06 | Up |
| AFAP1-AS1 | 4.5328559 | 3.87E-09 | 1.87E-08 | Up |
| AC073352.1 | 2.1898859 | 5.98E-12 | 4.15E-11 | Up |
| AP005137.2 | 3.3612151 | 3.06E-06 | 9.45E-06 | Up |
| AC131235.3 | 2.0219502 | 8.45E-13 | 6.64E-12 | Up |
| AC007881.3 | 2.5905524 | 1.50E-13 | 1.29E-12 | Up |
| AC103702.2 | 5.4095296 | 3.80E-14 | 3.51E-13 | Up |
| AC097532.2 | 2.0115643 | 4.61E-07 | 1.63E-06 | Up |
| AP000864.1 | 2.2657258 | 3.39E-10 | 1.88E-09 | Up |
| AL008721.1 | 4.0225646 | 1.08E-09 | 5.60E-09 | Up |
| AC015712.6 | 2.3573625 | 1.06E-08 | 4.78E-08 | Up |
| AP000525.1 | 3.0333394 | 8.25E-15 | 8.15E-14 | Up |
| AC113189.4 | 3.115602 | 1.81E-12 | 1.37E-11 | Up |
| AL022324.3 | 3.1932496 | 7.02E-09 | 3.25E-08 | Up |
| AL356299.2 | 2.8110635 | 8.58E-22 | 1.57E-20 | Up |
| AP000553.2 | 2.4697905 | 3.82E-12 | 2.75E-11 | Up |
| MYHAS | 2.4701208 | 4.71E-09 | 2.23E-08 | Up |
| LINC02012 | 2.8161615 | 1.73E-12 | 1.31E-11 | Up |
| DGCR9 | 2.1218168 | 2.88E-10 | 1.61E-09 | Up |
| AC017083.1 | 2.7375843 | 1.90E-22 | 3.71E-21 | Up |
| AC093865.1 | 3.4283555 | 5.38E-10 | 2.90E-09 | Up |
| AP005229.2 | 4.5737328 | 1.25E-06 | 4.08E-06 | Up |
| AL133215.2 | 3.0476017 | 3.46E-35 | 1.64E-33 | Up |
| AC006946.2 | 2.2370921 | 1.08E-05 | 3.04E-05 | Up |
| AC090912.3 | 3.5353937 | 8.48E-10 | 4.44E-09 | Up |
| AC116565.1 | 4.2171769 | 9.85E-06 | 2.79E-05 | Up |
| Z82243.1 | 2.0787877 | 2.41E-14 | 2.27E-13 | Up |
| AC010997.5 | 2.0940859 | 3.86E-08 | 1.61E-07 | Up |
| AC009275.1 | 2.1739785 | 3.43E-08 | 1.45E-07 | Up |
| RBAKDN | 2.5661828 | 4.83E-06 | 1.45E-05 | Up |
| Z83851.2 | 2.5585637 | 7.83E-23 | 1.57E-21 | Up |
| AL355472.4 | 3.0200794 | 1.08E-11 | 7.26E-11 | Up |
| AC005291.2 | 2.0735392 | 0.0003375 | 0.0007372 | Up |
| AC083880.1 | 2.0213514 | 9.63E-15 | 9.41E-14 | Up |
| AP000904.1 | 2.02945 | 0.0023255 | 0.0043801 | Up |
| AL645608.9 | 2.328701 | 6.48E-06 | 1.89E-05 | Up |
| LINC02348 | 2.22957 | 6.91E-06 | 2.01E-05 | Up |
| AC020658.4 | 3.1127887 | 1.91E-12 | 1.44E-11 | Up |
| AL109954.2 | 2.7032082 | 4.98E-06 | 1.48E-05 | Up |
| FRMD6-AS1 | 2.3082415 | 2.55E-20 | 4.17E-19 | Up |
| AC022079.1 | 2.7157176 | 1.36E-08 | 6.01E-08 | Up |
| AL049794.1 | 2.0403141 | 9.26E-09 | 4.20E-08 | Up |
| AC022929.2 | 2.3757143 | 5.16E-06 | 1.53E-05 | Up |
| AC126177.8 | 2.341781 | 0.0002711 | 0.0006007 | Up |
| AL121772.1 | 2.2412267 | 1.24E-09 | 6.39E-09 | Up |
| AC078909.2 | 2.0328996 | 3.44E-07 | 1.23E-06 | Up |
| AL355338.1 | 2.3366944 | 1.19E-25 | 3.01E-24 | Up |
| AC025166.1 | 3.8522732 | 5.85E-19 | 8.41E-18 | Up |
| AL023803.2 | 2.8459678 | 9.50E-21 | 1.60E-19 | Up |
| LINC01297 | 6.0572813 | 4.46E-08 | 1.84E-07 | Up |
| AC132807.2 | 5.7769253 | 8.02E-11 | 4.80E-10 | Up |
| AL135936.1 | 2.8878362 | 8.91E-06 | 2.55E-05 | Up |
| AC011700.1 | 3.6907906 | 7.37E-08 | 2.94E-07 | Up |
| AL031710.2 | 2.0196178 | 5.62E-12 | 3.91E-11 | Up |
| AL161431.1 | 5.1140471 | 5.54E-12 | 3.86E-11 | Up |
| AC018695.6 | 2.78025 | 1.21E-12 | 9.36E-12 | Up |
| AL121832.3 | 2.0752143 | 1.45E-19 | 2.19E-18 | Up |
| AL049539.1 | 3.1594685 | 7.93E-17 | 9.48E-16 | Up |
| AL121827.2 | 2.444971 | 4.20E-06 | 1.27E-05 | Up |
| AL513548.3 | 2.3683458 | 0.0021487 | 0.0040673 | Up |
| AL139385.1 | 2.915056 | 9.68E-20 | 1.49E-18 | Up |
| AC125603.4 | 3.2250285 | 9.93E-07 | 3.30E-06 | Up |
| AC110285.6 | 2.4242389 | 2.45E-10 | 1.38E-09 | Up |
| AC026336.3 | 6.2434253 | 3.80E-11 | 2.36E-10 | Up |
| AC009118.2 | 3.2339923 | 7.13E-18 | 9.46E-17 | Up |
| AL136221.1 | 2.5251436 | 6.63E-12 | 4.58E-11 | Up |
| AL136962.1 | 2.001896 | 0.0006819 | 0.0014145 | Up |
| AC137834.2 | 2.6257447 | 7.63E-13 | 6.05E-12 | Up |
| AC105137.2 | 2.0615872 | 3.38E-20 | 5.47E-19 | Up |
| AC008556.1 | 2.1736726 | 8.77E-11 | 5.22E-10 | Up |
| AL122125.1 | 3.0427024 | 4.01E-23 | 8.32E-22 | Up |
| AL589743.5 | 3.1228418 | 8.58E-09 | 3.91E-08 | Up |
| AC090116.1 | 3.3716503 | 6.62E-05 | 0.0001633 | Up |
| AC083809.1 | 2.4101405 | 3.72E-05 | 9.58E-05 | Up |
| LHX1-DT | 7.5014432 | 9.66E-10 | 5.03E-09 | Up |
| AL160412.1 | 3.2252634 | 3.53E-06 | 1.08E-05 | Up |
| AL109976.1 | 2.3034861 | 2.19E-09 | 1.09E-08 | Up |
| AP001065.2 | 3.9579446 | 5.32E-05 | 0.0001338 | Up |
| AC010271.2 | 2.0453633 | 4.55E-06 | 1.37E-05 | Up |
| AC007996.1 | 2.4566484 | 2.45E-17 | 3.09E-16 | Up |
| AL023803.3 | 2.9671931 | 2.31E-13 | 1.96E-12 | Up |
| AC008406.3 | 5.4667386 | 2.39E-17 | 3.03E-16 | Up |
| AP003900.1 | 6.0771819 | 3.24E-08 | 1.37E-07 | Up |
| AC126175.1 | 3.0177122 | 0.0053953 | 0.0094523 | Up |
| AC107308.1 | 5.5629121 | 1.71E-15 | 1.80E-14 | Up |
| AL133325.3 | 6.5155252 | 8.60E-14 | 7.63E-13 | Up |
| AL161669.3 | 2.2787633 | 1.12E-06 | 3.70E-06 | Up |
| AC036108.4 | 2.8353082 | 4.14E-14 | 3.82E-13 | Up |
| AL118505.1 | 3.337275 | 1.61E-24 | 3.71E-23 | Up |
| LINC01971 | 3.4265548 | 7.03E-12 | 4.83E-11 | Up |
| AL359513.1 | 2.6604833 | 2.51E-20 | 4.13E-19 | Up |
| AC068831.6 | 4.7560368 | 3.29E-29 | 1.07E-27 | Up |
| AL161645.1 | 2.7687805 | 1.02E-06 | 3.38E-06 | Up |
| AC023310.4 | 5.2869194 | 1.28E-09 | 6.54E-09 | Up |
| AL162574.2 | 3.2577326 | 1.31E-06 | 4.26E-06 | Up |
| AL590226.2 | 4.1473633 | 8.38E-20 | 1.30E-18 | Up |
| AC007383.4 | 3.5593106 | 0.0003227 | 0.000707 | Up |
| CR392039.3 | 3.8538229 | 2.53E-05 | 6.70E-05 | Up |
| FAM230C | 6.834398 | 1.94E-07 | 7.22E-07 | Up |
| LINC01666 | 2.72872 | 0.0001075 | 0.0002553 | Up |
| AL358613.2 | 2.4594369 | 3.18E-05 | 8.28E-05 | Up |
| FP325330.3 | 6.4757508 | 3.55E-26 | 9.31E-25 | Up |
| AC022784.8 | 2.564079 | 6.60E-09 | 3.06E-08 | Up |
| CU634019.6 | 2.505805 | 2.41E-09 | 1.19E-08 | Up |
| LINC01667 | 5.0509013 | 6.57E-05 | 0.000162 | Up |
| CU639417.4 | 2.4787288 | 1.97E-07 | 7.30E-07 | Up |
| AL021877.2 | 5.9706531 | 1.96E-12 | 1.48E-11 | Up |
| KCNIP4-IT1 | 3.5530442 | 4.37E-07 | 1.55E-06 | Up |
| SH3PXD2A-AS1 | 3.637425 | 3.27E-14 | 3.04E-13 | Up |
| PCAT5 | 3.1068465 | 2.97E-05 | 7.80E-05 | Up |
| LINC01202 | 5.6479099 | 1.86E-11 | 1.21E-10 | Up |
| ELDR | 4.4257171 | 2.42E-09 | 1.20E-08 | Up |
| FOXCUT | 2.1796928 | 2.74E-05 | 7.23E-05 | Up |
| LINC00628 | 3.1553905 | 5.97E-05 | 0.0001485 | Up |
| AP003500.1 | 2.358518 | 9.46E-05 | 0.000227 | Up |
| LINC01127 | 3.9000761 | 4.53E-08 | 1.87E-07 | Up |
| LINC01338 | 2.3230146 | 6.12E-06 | 1.79E-05 | Up |
| HELLPAR | 2.6160548 | 7.90E-23 | 1.58E-21 | Up |
| AP003500.2 | 2.6114861 | 9.30E-05 | 0.0002235 | Up |
| SNHG4 | 2.7542133 | 1.25E-27 | 3.59E-26 | Up |
| BLACAT1 | 2.7737248 | 3.89E-12 | 2.80E-11 | Up |
| SAMD12-AS1 | 2.2587187 | 9.08E-08 | 3.56E-07 | Up |
| AP000851.2 | 3.3919039 | 1.02E-05 | 2.90E-05 | Up |
| AL009179.1 | 2.1050617 | 1.89E-13 | 1.61E-12 | Up |

**Supplementary table 5: Differentially expressed miRNAs in non-smoking LUSC**

| **miRNA** | **logFC** | **p-value** | **FDR** | **regulate** |
| --- | --- | --- | --- | --- |
| hsa-mir-1-1 | -2.702098 | 1.13E-28 | 2.17E-27 | Down |
| hsa-mir-1-2 | -2.71192 | 8.62E-31 | 2.07E-29 | Down |
| hsa-mir-101-1 | -2.269193 | 4.24E-62 | 3.63E-60 | Down |
| hsa-mir-101-2 | -2.274889 | 1.10E-62 | 1.10E-60 | Down |
| hsa-mir-1258 | -2.121515 | 1.89E-14 | 1.46E-13 | Down |
| hsa-mir-133a-1 | -3.016554 | 9.92E-40 | 4.95E-38 | Down |
| hsa-mir-133a-2 | -2.882692 | 9.14E-35 | 2.74E-33 | Down |
| hsa-mir-133b | -3.22451 | 9.42E-36 | 3.13E-34 | Down |
| hsa-mir-135a-2 | -2.211132 | 8.90E-09 | 3.98E-08 | Down |
| hsa-mir-139 | -2.646699 | 1.51E-49 | 1.01E-47 | Down |
| hsa-mir-144 | -3.865402 | 2.00E-79 | 1.20E-76 | Down |
| hsa-mir-218-1 | -2.081401 | 3.02E-40 | 1.65E-38 | Down |
| hsa-mir-218-2 | -2.05782 | 1.05E-37 | 4.48E-36 | Down |
| hsa-mir-3065 | -2.074562 | 1.49E-25 | 2.48E-24 | Down |
| hsa-mir-30a | -2.991353 | 1.59E-67 | 1.90E-65 | Down |
| hsa-mir-30d | -2.454671 | 9.06E-69 | 1.36E-66 | Down |
| hsa-mir-326 | -2.725019 | 2.24E-36 | 7.90E-35 | Down |
| hsa-mir-338 | -2.475306 | 1.98E-35 | 6.25E-34 | Down |
| hsa-mir-34c | -2.004021 | 4.00E-11 | 2.24E-10 | Down |
| hsa-mir-451a | -3.652675 | 5.26E-61 | 3.94E-59 | Down |
| hsa-mir-4709 | -2.07182 | 3.93E-22 | 5.11E-21 | Down |
| hsa-mir-4732 | -3.584951 | 1.54E-46 | 9.21E-45 | Down |
| hsa-mir-4777 | -2.152028 | 1.77E-23 | 2.47E-22 | Down |
| hsa-mir-486-1 | -3.922146 | 2.01E-71 | 6.02E-69 | Down |
| hsa-mir-486-2 | -3.895439 | 1.20E-70 | 2.40E-68 | Down |
| hsa-mir-490 | -3.608778 | 3.71E-29 | 7.66E-28 | Down |
| hsa-mir-105-1 | 5.5698079 | 1.17E-09 | 5.78E-09 | Up |
| hsa-mir-105-2 | 5.5572755 | 5.33E-10 | 2.68E-09 | Up |
| hsa-mir-1248 | 3.2469652 | 4.40E-13 | 2.83E-12 | Up |
| hsa-mir-1269a | 5.6025373 | 1.60E-12 | 9.68E-12 | Up |
| hsa-mir-1269b | 8.6509919 | 3.83E-10 | 2.01E-09 | Up |
| hsa-mir-1276 | 2.131983 | 1.30E-06 | 4.22E-06 | Up |
| hsa-mir-129-1 | 3.0496762 | 6.63E-07 | 2.25E-06 | Up |
| hsa-mir-129-2 | 3.0478219 | 5.20E-07 | 1.80E-06 | Up |
| hsa-mir-1293 | 2.0474829 | 0.0002017 | 0.0004832 | Up |
| hsa-mir-130b | 2.1479146 | 5.75E-28 | 1.04E-26 | Up |
| hsa-mir-137 | 2.0560952 | 0.0028685 | 0.0053695 | Up |
| hsa-mir-147b | 2.1016296 | 7.44E-07 | 2.48E-06 | Up |
| hsa-mir-149 | 2.8214504 | 3.25E-20 | 3.60E-19 | Up |
| hsa-mir-183 | 2.4492123 | 1.32E-22 | 1.76E-21 | Up |
| hsa-mir-1910 | 4.1631605 | 2.89E-16 | 2.62E-15 | Up |
| hsa-mir-1911 | 2.5525622 | 0.0006026 | 0.0013368 | Up |
| hsa-mir-196a-1 | 5.8124441 | 8.03E-18 | 8.15E-17 | Up |
| hsa-mir-196a-2 | 5.7040844 | 5.09E-18 | 5.25E-17 | Up |
| hsa-mir-196b | 4.9726811 | 3.68E-37 | 1.47E-35 | Up |
| hsa-mir-205 | 4.9065345 | 1.91E-33 | 4.98E-32 | Up |
| hsa-mir-210 | 3.9440626 | 2.03E-39 | 9.37E-38 | Up |
| hsa-mir-224 | 2.4768494 | 4.22E-13 | 2.75E-12 | Up |
| hsa-mir-301b | 3.110294 | 1.07E-19 | 1.17E-18 | Up |
| hsa-mir-31 | 5.2931249 | 1.56E-16 | 1.46E-15 | Up |
| hsa-mir-3161 | 2.59129 | 0.001661 | 0.0033363 | Up |
| hsa-mir-323a | 2.5956544 | 4.96E-08 | 1.98E-07 | Up |
| hsa-mir-323b | 3.1678275 | 2.21E-12 | 1.33E-11 | Up |
| hsa-mir-3618 | 2.0771364 | 1.43E-05 | 3.87E-05 | Up |
| hsa-mir-3662 | 3.857502 | 4.47E-14 | 3.26E-13 | Up |
| hsa-mir-4449 | 3.5320419 | 1.57E-10 | 8.46E-10 | Up |
| hsa-mir-4640 | 2.4158394 | 6.60E-09 | 3.06E-08 | Up |
| hsa-mir-4652 | 6.0380093 | 6.26E-32 | 1.56E-30 | Up |
| hsa-mir-466 | 2.3757994 | 0.0002691 | 0.0006346 | Up |
| hsa-mir-4664 | 2.5874001 | 1.21E-13 | 8.43E-13 | Up |
| hsa-mir-4665 | 2.2194398 | 8.25E-06 | 2.36E-05 | Up |
| hsa-mir-4713 | 3.5663138 | 4.16E-10 | 2.17E-09 | Up |
| hsa-mir-4745 | 2.8137507 | 3.87E-08 | 1.58E-07 | Up |
| hsa-mir-4766 | 2.5368992 | 9.06E-12 | 5.32E-11 | Up |
| hsa-mir-4778 | 4.6285523 | 1.07E-11 | 6.22E-11 | Up |
| hsa-mir-4788 | 2.4208738 | 0.0015061 | 0.0030581 | Up |
| hsa-mir-483 | 2.7830415 | 8.65E-05 | 0.0002159 | Up |
| hsa-mir-499a | 2.4324948 | 0.002212 | 0.0042741 | Up |
| hsa-mir-503 | 2.0206557 | 3.05E-13 | 2.03E-12 | Up |
| hsa-mir-5092 | 2.7044108 | 1.53E-09 | 7.50E-09 | Up |
| hsa-mir-516a-1 | 3.2580345 | 0.0030983 | 0.005728 | Up |
| hsa-mir-516a-2 | 3.2808157 | 0.0034332 | 0.0062317 | Up |
| hsa-mir-519a-1 | 3.1745854 | 0.0009477 | 0.0020273 | Up |
| hsa-mir-548f-1 | 3.9861765 | 5.01E-08 | 1.99E-07 | Up |
| hsa-mir-5579 | 3.4536325 | 8.84E-13 | 5.52E-12 | Up |
| hsa-mir-577 | 4.3768165 | 2.96E-15 | 2.59E-14 | Up |
| hsa-mir-615 | 3.8009073 | 5.22E-15 | 4.30E-14 | Up |
| hsa-mir-616 | 2.0533855 | 1.04E-14 | 8.44E-14 | Up |
| hsa-mir-6499 | 6.098368 | 8.57E-26 | 1.47E-24 | Up |
| hsa-mir-6510 | 4.5717133 | 1.84E-13 | 1.26E-12 | Up |
| hsa-mir-6512 | 3.3497258 | 1.84E-08 | 7.84E-08 | Up |
| hsa-mir-6728 | 2.0785126 | 1.70E-06 | 5.41E-06 | Up |
| hsa-mir-675 | 3.0610428 | 7.07E-07 | 2.36E-06 | Up |
| hsa-mir-7-3 | 2.0679856 | 7.00E-07 | 2.36E-06 | Up |
| hsa-mir-708 | 2.7289785 | 3.19E-25 | 5.17E-24 | Up |
| hsa-mir-7112 | 2.1873487 | 1.92E-07 | 7.06E-07 | Up |
| hsa-mir-760 | 2.2047161 | 4.47E-10 | 2.29E-09 | Up |
| hsa-mir-7641-1 | 2.7330138 | 6.80E-06 | 1.98E-05 | Up |
| hsa-mir-767 | 5.1731284 | 3.14E-09 | 1.49E-08 | Up |
| hsa-mir-7974 | 3.58652 | 1.66E-11 | 9.55E-11 | Up |
| hsa-mir-873 | 2.9049948 | 2.32E-06 | 7.15E-06 | Up |
| hsa-mir-877 | 2.1476848 | 2.35E-13 | 1.58E-12 | Up |
| hsa-mir-891a | 5.630049 | 2.20E-08 | 9.29E-08 | Up |
| hsa-mir-9-1 | 3.487047 | 4.24E-15 | 3.58E-14 | Up |
| hsa-mir-9-2 | 3.4838564 | 5.24E-15 | 4.30E-14 | Up |
| hsa-mir-9-3 | 3.5018179 | 3.95E-15 | 3.38E-14 | Up |
| hsa-mir-944 | 5.9147094 | 1.15E-34 | 3.28E-33 | Up |
| hsa-mir-96 | 2.5472996 | 7.61E-21 | 8.61E-20 | Up |

**Supplementary table 6: Differentially expressed mRNAs in non-smoking LUSC**

| **mRNA** | **logFC** | **p-value** | **FDR** | **regulate** |
| --- | --- | --- | --- | --- |
| FGR | -2.497208 | 1.10E-50 | 3.87E-49 | Down |
| ENPP4 | -2.105507 | 4.50E-29 | 6.17E-28 | Down |
| CFTR | -2.628052 | 1.86E-19 | 1.37E-18 | Down |
| HSPB6 | -3.604646 | 7.59E-69 | 5.90E-67 | Down |
| PDK4 | -4.193899 | 1.25E-79 | 1.47E-77 | Down |
| ZMYND10 | -3.2114 | 4.56E-29 | 6.25E-28 | Down |
| SLC4A1 | -2.091392 | 1.53E-06 | 3.54E-06 | Down |
| ALDH3B1 | -3.176546 | 1.74E-52 | 6.49E-51 | Down |
| ARHGAP44 | -3.017268 | 1.36E-66 | 9.29E-65 | Down |
| CEACAM21 | -2.264565 | 1.49E-31 | 2.36E-30 | Down |
| DNAH9 | -3.031211 | 9.69E-22 | 8.54E-21 | Down |
| CACNA2D2 | -4.786006 | 2.82E-114 | 1.21E-111 | Down |
| SELE | -3.35793 | 4.05E-30 | 5.94E-29 | Down |
| PGLYRP1 | -2.743828 | 1.23E-32 | 2.02E-31 | Down |
| RHOBTB2 | -2.827725 | 1.44E-58 | 6.86E-57 | Down |
| IYD | -2.258437 | 4.06E-12 | 1.63E-11 | Down |
| HHATL | -2.468049 | 3.03E-11 | 1.13E-10 | Down |
| SEMA3G | -3.314379 | 2.13E-110 | 8.17E-108 | Down |
| SLC6A13 | -2.441512 | 4.64E-26 | 5.31E-25 | Down |
| BTK | -2.079982 | 1.08E-29 | 1.55E-28 | Down |
| ANOS1 | -2.34416 | 4.76E-28 | 6.16E-27 | Down |
| TYROBP | -2.008313 | 1.76E-27 | 2.20E-26 | Down |
| SEMA3B | -2.675118 | 1.84E-30 | 2.75E-29 | Down |
| ALOX5 | -2.707784 | 7.74E-48 | 2.44E-46 | Down |
| GPRC5A | -3.131799 | 7.77E-46 | 2.27E-44 | Down |
| SLC11A1 | -2.470146 | 1.64E-44 | 4.55E-43 | Down |
| ATP1A2 | -3.988275 | 5.15E-56 | 2.23E-54 | Down |
| VSIG2 | -3.672039 | 1.92E-49 | 6.49E-48 | Down |
| MARCO | -3.850503 | 3.37E-49 | 1.13E-47 | Down |
| HGF | -2.301196 | 3.30E-23 | 3.17E-22 | Down |
| C8B | -4.590886 | 2.22E-31 | 3.49E-30 | Down |
| FHL1 | -3.833535 | 8.45E-100 | 2.18E-97 | Down |
| HSD17B6 | -4.114111 | 2.31E-170 | 2.08E-166 | Down |
| MUSK | -2.751885 | 4.05E-38 | 8.67E-37 | Down |
| ARHGAP31 | -2.765412 | 7.27E-78 | 7.85E-76 | Down |
| GAB2 | -2.173107 | 9.87E-39 | 2.16E-37 | Down |
| MYOC | -3.103441 | 4.33E-12 | 1.74E-11 | Down |
| DAPK2 | -2.439917 | 3.33E-45 | 9.62E-44 | Down |
| OTC | -4.508309 | 3.85E-64 | 2.40E-62 | Down |
| FLT4 | -2.438942 | 3.24E-62 | 1.82E-60 | Down |
| MSR1 | -2.767133 | 6.85E-39 | 1.51E-37 | Down |
| C6 | -3.628101 | 5.23E-26 | 5.95E-25 | Down |
| ADRB1 | -3.781627 | 1.64E-63 | 9.86E-62 | Down |
| PREX2 | -3.246215 | 1.72E-79 | 1.99E-77 | Down |
| ARHGAP6 | -3.210421 | 1.76E-72 | 1.60E-70 | Down |
| ROS1 | -2.793987 | 7.72E-22 | 6.86E-21 | Down |
| LMO3 | -3.32767 | 4.38E-33 | 7.38E-32 | Down |
| CELF2 | -2.547132 | 1.18E-52 | 4.46E-51 | Down |
| LAMC3 | -2.073883 | 1.58E-24 | 1.64E-23 | Down |
| KCNQ1 | -2.101886 | 3.51E-30 | 5.18E-29 | Down |
| CCDC85A | -3.487076 | 2.73E-55 | 1.15E-53 | Down |
| RASGRF1 | -3.729579 | 2.29E-46 | 6.90E-45 | Down |
| LIMCH1 | -3.380372 | 3.06E-71 | 2.61E-69 | Down |
| WISP2 | -3.506539 | 2.10E-63 | 1.25E-61 | Down |
| CHI3L2 | -3.335869 | 3.44E-42 | 8.63E-41 | Down |
| CALCRL | -2.527118 | 7.46E-56 | 3.21E-54 | Down |
| SLC9A3R2 | -2.37775 | 4.63E-76 | 4.77E-74 | Down |
| GLP2R | -2.580652 | 5.35E-11 | 1.95E-10 | Down |
| TIE1 | -2.934583 | 1.39E-94 | 2.98E-92 | Down |
| SPI1 | -2.144089 | 7.28E-37 | 1.46E-35 | Down |
| CLDN18 | -6.105105 | 9.33E-87 | 1.46E-84 | Down |
| KLF6 | -2.324104 | 1.53E-62 | 8.72E-61 | Down |
| ATP11A | -2.17071 | 1.32E-46 | 4.02E-45 | Down |
| RASGRP2 | -2.136847 | 1.78E-27 | 2.23E-26 | Down |
| PYGM | -2.01813 | 2.43E-34 | 4.29E-33 | Down |
| ADGRF5 | -3.842551 | 4.15E-104 | 1.27E-101 | Down |
| TGFBR3 | -2.01089 | 2.08E-25 | 2.27E-24 | Down |
| PLA2G10 | -2.145386 | 1.86E-23 | 1.81E-22 | Down |
| FGF10 | -2.522678 | 1.64E-12 | 6.83E-12 | Down |
| PTPN21 | -2.664835 | 3.06E-96 | 6.98E-94 | Down |
| RPS6KA2 | -2.657102 | 2.11E-85 | 3.06E-83 | Down |
| CYBRD1 | -2.326821 | 1.17E-54 | 4.83E-53 | Down |
| LIMS2 | -3.051054 | 4.81E-89 | 8.25E-87 | Down |
| TRHDE | -3.488042 | 1.67E-20 | 1.33E-19 | Down |
| FERMT2 | -2.009128 | 1.90E-43 | 5.07E-42 | Down |
| CD5L | -4.647103 | 3.98E-50 | 1.36E-48 | Down |
| FRY | -2.458461 | 1.92E-46 | 5.79E-45 | Down |
| MGLL | -2.402432 | 3.97E-42 | 9.95E-41 | Down |
| NTN4 | -2.309704 | 1.27E-39 | 2.87E-38 | Down |
| SCARF1 | -2.304392 | 2.89E-61 | 1.54E-59 | Down |
| SPAG6 | -2.954294 | 2.02E-17 | 1.27E-16 | Down |
| ACTN2 | -3.167086 | 3.23E-35 | 5.96E-34 | Down |
| ITGA8 | -3.478128 | 6.82E-73 | 6.41E-71 | Down |
| LAMP3 | -2.841324 | 6.65E-48 | 2.11E-46 | Down |
| EDN1 | -2.147773 | 4.54E-25 | 4.86E-24 | Down |
| ITM2A | -2.270009 | 5.81E-31 | 8.94E-30 | Down |
| RUNX1T1 | -2.070769 | 6.50E-24 | 6.53E-23 | Down |
| TNS1 | -3.102534 | 1.08E-103 | 3.20E-101 | Down |
| SCTR | -2.865015 | 1.43E-23 | 1.40E-22 | Down |
| SLC4A4 | -2.935496 | 2.04E-31 | 3.21E-30 | Down |
| PIH1D3 | -3.169076 | 1.79E-15 | 9.65E-15 | Down |
| CPB2 | -5.486298 | 3.26E-79 | 3.68E-77 | Down |
| CXCL2 | -4.2974 | 8.29E-71 | 6.86E-69 | Down |
| COL4A4 | -2.739466 | 2.37E-29 | 3.32E-28 | Down |
| MEF2C | -2.026858 | 5.47E-38 | 1.16E-36 | Down |
| CACNA1S | -2.45479 | 6.74E-10 | 2.20E-09 | Down |
| LRP2 | -3.170357 | 1.49E-30 | 2.24E-29 | Down |
| PGR | -2.834114 | 7.53E-45 | 2.12E-43 | Down |
| EFR3B | -2.436517 | 1.53E-27 | 1.92E-26 | Down |
| FCN1 | -3.26524 | 9.14E-59 | 4.40E-57 | Down |
| ABCB1 | -2.573673 | 4.82E-43 | 1.26E-41 | Down |
| PPP1R15A | -2.074318 | 2.03E-47 | 6.35E-46 | Down |
| CETP | -2.207351 | 1.00E-35 | 1.89E-34 | Down |
| CASS4 | -2.978201 | 1.56E-63 | 9.42E-62 | Down |
| REM1 | -2.104215 | 1.31E-22 | 1.21E-21 | Down |
| SIGLEC1 | -2.20035 | 3.42E-24 | 3.49E-23 | Down |
| F11 | -4.795998 | 1.32E-53 | 5.21E-52 | Down |
| CFAP61 | -2.663877 | 6.06E-26 | 6.87E-25 | Down |
| TBX5 | -2.249511 | 2.57E-37 | 5.29E-36 | Down |
| ICAM1 | -2.687809 | 8.17E-36 | 1.55E-34 | Down |
| NLRC4 | -2.630258 | 1.13E-73 | 1.08E-71 | Down |
| IL5RA | -2.908325 | 4.08E-28 | 5.30E-27 | Down |
| ABCC6 | -2.425839 | 2.05E-45 | 5.94E-44 | Down |
| APOH | -4.533021 | 2.25E-32 | 3.67E-31 | Down |
| CMA1 | -2.51178 | 9.68E-19 | 6.77E-18 | Down |
| JPH4 | -2.323131 | 3.56E-18 | 2.38E-17 | Down |
| TEKT2 | -2.793177 | 1.11E-22 | 1.03E-21 | Down |
| DPYSL2 | -2.824252 | 7.12E-87 | 1.13E-84 | Down |
| FMO2 | -3.268513 | 3.54E-45 | 1.02E-43 | Down |
| SH2D3C | -2.915118 | 4.15E-117 | 2.08E-114 | Down |
| SORBS1 | -2.729843 | 1.33E-62 | 7.63E-61 | Down |
| CRTAC1 | -3.655744 | 6.60E-40 | 1.51E-38 | Down |
| PGC | -5.176914 | 1.28E-48 | 4.13E-47 | Down |
| IGFALS | -2.710399 | 1.29E-24 | 1.34E-23 | Down |
| GADD45B | -2.998662 | 2.78E-88 | 4.69E-86 | Down |
| SERPIND1 | -2.128238 | 1.21E-07 | 3.15E-07 | Down |
| SUSD2 | -4.605957 | 2.52E-95 | 5.68E-93 | Down |
| SEC14L3 | -4.04809 | 4.22E-35 | 7.73E-34 | Down |
| MFNG | -2.222067 | 9.26E-48 | 2.92E-46 | Down |
| GGTLC2 | -4.045551 | 3.42E-49 | 1.15E-47 | Down |
| SLC5A4 | -2.339744 | 2.31E-19 | 1.70E-18 | Down |
| TIMP3 | -2.213035 | 4.37E-20 | 3.40E-19 | Down |
| CTSG | -2.318497 | 6.91E-16 | 3.84E-15 | Down |
| GALNT16 | -2.104365 | 6.71E-15 | 3.44E-14 | Down |
| SLC8A3 | -2.881686 | 4.89E-31 | 7.56E-30 | Down |
| TUBB1 | -3.275005 | 2.56E-108 | 8.39E-106 | Down |
| SPEF1 | -2.421279 | 1.25E-20 | 1.01E-19 | Down |
| RASSF2 | -2.185932 | 2.32E-33 | 3.99E-32 | Down |
| ANGPT4 | -4.190814 | 6.37E-83 | 8.21E-81 | Down |
| SIRPB1 | -3.141691 | 3.15E-49 | 1.06E-47 | Down |
| CCM2L | -3.021819 | 1.42E-93 | 2.92E-91 | Down |
| PPP1R16B | -2.154049 | 4.87E-27 | 5.95E-26 | Down |
| EPPIN | -2.670557 | 1.89E-10 | 6.55E-10 | Down |
| TLR8 | -2.223755 | 9.06E-21 | 7.40E-20 | Down |
| CHRDL1 | -3.65517 | 1.76E-46 | 5.33E-45 | Down |
| RS1 | -5.269418 | 6.56E-115 | 2.96E-112 | Down |
| GATA1 | -2.274113 | 1.92E-31 | 3.03E-30 | Down |
| CD40LG | -2.046561 | 6.18E-17 | 3.73E-16 | Down |
| SGCG | -2.54661 | 6.85E-16 | 3.81E-15 | Down |
| RGCC | -3.503063 | 3.15E-142 | 4.73E-139 | Down |
| PLLP | -2.070642 | 4.85E-40 | 1.12E-38 | Down |
| WFDC1 | -2.519669 | 9.64E-42 | 2.38E-40 | Down |
| FOXF1 | -2.883539 | 1.09E-69 | 8.76E-68 | Down |
| ZP2 | -2.500714 | 1.75E-15 | 9.44E-15 | Down |
| CRYM | -2.61749 | 2.05E-18 | 1.39E-17 | Down |
| TMC5 | -2.305605 | 1.65E-18 | 1.13E-17 | Down |
| AQP9 | -2.15338 | 4.04E-21 | 3.40E-20 | Down |
| CORO2B | -2.378771 | 3.54E-28 | 4.61E-27 | Down |
| RASL12 | -2.512501 | 6.60E-65 | 4.32E-63 | Down |
| CTSH | -2.300249 | 2.93E-39 | 6.56E-38 | Down |
| FAM189A1 | -2.514863 | 6.99E-18 | 4.56E-17 | Down |
| RP1 | -2.301328 | 9.32E-09 | 2.72E-08 | Down |
| RETN | -4.369109 | 2.33E-62 | 1.32E-60 | Down |
| CLEC4M | -4.908325 | 6.21E-68 | 4.53E-66 | Down |
| NOVA2 | -3.050338 | 3.70E-91 | 6.61E-89 | Down |
| LILRA1 | -2.241188 | 8.19E-36 | 1.56E-34 | Down |
| PRX | -3.914503 | 1.92E-145 | 3.47E-142 | Down |
| DENND3 | -2.409022 | 5.85E-56 | 2.52E-54 | Down |
| CEACAM4 | -2.166825 | 1.91E-24 | 1.98E-23 | Down |
| CD33 | -2.104358 | 3.98E-30 | 5.85E-29 | Down |
| SIGLEC6 | -2.591293 | 2.71E-33 | 4.62E-32 | Down |
| SIGLEC5 | -2.299998 | 4.06E-25 | 4.36E-24 | Down |
| HAS1 | -2.348891 | 1.01E-13 | 4.65E-13 | Down |
| CAPS | -2.022139 | 5.86E-19 | 4.17E-18 | Down |
| RASIP1 | -2.276914 | 2.86E-30 | 4.23E-29 | Down |
| PDE4C | -2.277015 | 2.93E-21 | 2.49E-20 | Down |
| MAG | -2.960178 | 5.71E-17 | 3.45E-16 | Down |
| CAV1 | -3.043408 | 7.07E-57 | 3.14E-55 | Down |
| TSPAN12 | -2.603248 | 5.91E-44 | 1.60E-42 | Down |
| CPED1 | -2.581645 | 6.98E-34 | 1.22E-32 | Down |
| COBL | -2.737942 | 7.13E-28 | 9.13E-27 | Down |
| CCL24 | -2.881717 | 3.18E-24 | 3.25E-23 | Down |
| PPP1R17 | -2.594492 | 2.42E-16 | 1.39E-15 | Down |
| MEOX2 | -2.166826 | 4.27E-20 | 3.32E-19 | Down |
| SLC1A1 | -3.532161 | 1.40E-85 | 2.07E-83 | Down |
| PRUNE2 | -2.049174 | 8.01E-22 | 7.11E-21 | Down |
| C5 | -2.139684 | 5.48E-29 | 7.46E-28 | Down |
| OGN | -3.631454 | 7.41E-43 | 1.93E-41 | Down |
| ENG | -2.379795 | 3.55E-71 | 3.02E-69 | Down |
| AK1 | -2.397572 | 2.08E-84 | 2.88E-82 | Down |
| DOCK8 | -2.090352 | 1.45E-32 | 2.39E-31 | Down |
| PIP5K1B | -2.655035 | 7.54E-34 | 1.31E-32 | Down |
| SH3GL2 | -2.749662 | 7.59E-15 | 3.88E-14 | Down |
| PTGDS | -2.576776 | 3.13E-26 | 3.62E-25 | Down |
| SPOCK2 | -3.53038 | 2.11E-70 | 1.72E-68 | Down |
| CSF3 | -4.743289 | 1.15E-52 | 4.33E-51 | Down |
| ASPA | -3.731782 | 1.71E-81 | 2.13E-79 | Down |
| 4-Sep | -2.105443 | 4.37E-41 | 1.06E-39 | Down |
| SLC6A4 | -6.078579 | 2.52E-101 | 6.89E-99 | Down |
| ICAM2 | -2.569565 | 6.79E-52 | 2.49E-50 | Down |
| CCL2 | -2.668257 | 4.04E-35 | 7.39E-34 | Down |
| SGCA | -3.153408 | 6.52E-41 | 1.56E-39 | Down |
| MYH1 | -2.403732 | 7.43E-12 | 2.92E-11 | Down |
| VTN | -2.278944 | 6.80E-19 | 4.81E-18 | Down |
| WFS1 | -2.231073 | 8.33E-88 | 1.37E-85 | Down |
| SOD3 | -2.735573 | 1.73E-64 | 1.09E-62 | Down |
| SNX25 | -2.144989 | 7.65E-86 | 1.14E-83 | Down |
| LRP2BP | -2.628715 | 9.81E-87 | 1.53E-84 | Down |
| ZBTB16 | -4.461675 | 1.79E-60 | 9.28E-59 | Down |
| FOLR1 | -4.075606 | 8.23E-42 | 2.04E-40 | Down |
| FOLR3 | -2.308885 | 1.79E-10 | 6.23E-10 | Down |
| APOA5 | -2.251203 | 3.70E-10 | 1.24E-09 | Down |
| C11orf21 | -2.21963 | 1.21E-23 | 1.19E-22 | Down |
| PTPN5 | -3.238948 | 9.11E-34 | 1.58E-32 | Down |
| VWF | -3.41833 | 5.47E-113 | 2.29E-110 | Down |
| CD69 | -2.450715 | 4.43E-30 | 6.50E-29 | Down |
| SELPLG | -2.19526 | 1.49E-37 | 3.09E-36 | Down |
| DAO | -2.39812 | 2.54E-24 | 2.62E-23 | Down |
| ACSS3 | -2.334436 | 8.07E-26 | 9.06E-25 | Down |
| TNS2 | -2.567325 | 2.31E-109 | 8.48E-107 | Down |
| ALDH2 | -2.086633 | 8.01E-42 | 1.98E-40 | Down |
| ART4 | -3.782554 | 3.03E-56 | 1.31E-54 | Down |
| MGP | -2.37375 | 1.74E-34 | 3.10E-33 | Down |
| ADGRD1 | -4.037572 | 2.34E-92 | 4.44E-90 | Down |
| RSPH4A | -2.710858 | 1.84E-24 | 1.90E-23 | Down |
| NEDD9 | -2.266802 | 2.32E-44 | 6.36E-43 | Down |
| FAM184A | -2.632188 | 4.59E-35 | 8.36E-34 | Down |
| RIPOR2 | -2.040249 | 1.78E-25 | 1.96E-24 | Down |
| SASH1 | -2.096269 | 1.17E-60 | 6.14E-59 | Down |
| PHACTR1 | -2.92244 | 1.80E-72 | 1.63E-70 | Down |
| CD83 | -2.23193 | 6.74E-49 | 2.21E-47 | Down |
| BMP5 | -2.686553 | 1.35E-17 | 8.60E-17 | Down |
| FHL5 | -4.122883 | 3.94E-109 | 1.34E-106 | Down |
| KHDRBS2 | -3.969016 | 7.21E-44 | 1.94E-42 | Down |
| C6orf118 | -3.185656 | 4.69E-15 | 2.43E-14 | Down |
| CLIC5 | -5.316934 | 2.74E-133 | 2.60E-130 | Down |
| LY86 | -2.204385 | 4.13E-30 | 6.07E-29 | Down |
| C7 | -3.530598 | 6.09E-39 | 1.35E-37 | Down |
| NME5 | -2.670685 | 4.65E-23 | 4.44E-22 | Down |
| BTNL8 | -2.996781 | 2.43E-47 | 7.55E-46 | Down |
| PCDH12 | -3.012472 | 6.53E-107 | 2.10E-104 | Down |
| LIFR | -2.247514 | 3.17E-24 | 3.24E-23 | Down |
| RBP2 | -3.833156 | 1.28E-53 | 5.03E-52 | Down |
| LRRC31 | -2.458675 | 2.29E-11 | 8.64E-11 | Down |
| HYAL1 | -2.984193 | 3.09E-67 | 2.15E-65 | Down |
| EFCC1 | -3.726817 | 1.17E-97 | 2.81E-95 | Down |
| VIPR1 | -3.453535 | 9.41E-85 | 1.33E-82 | Down |
| TNNC1 | -4.931908 | 5.36E-86 | 8.06E-84 | Down |
| SPTBN1 | -2.035253 | 3.41E-93 | 6.76E-91 | Down |
| ACADL | -3.373616 | 2.80E-46 | 8.41E-45 | Down |
| DNAH6 | -2.04731 | 8.12E-12 | 3.18E-11 | Down |
| ST3GAL5 | -2.073043 | 2.35E-36 | 4.56E-35 | Down |
| IL1RL1 | -3.480504 | 1.31E-24 | 1.36E-23 | Down |
| MLPH | -2.774816 | 2.28E-28 | 3.00E-27 | Down |
| EPAS1 | -3.335544 | 5.29E-119 | 2.73E-116 | Down |
| TNR | -3.638394 | 1.32E-31 | 2.10E-30 | Down |
| ANGPTL1 | -3.388036 | 2.54E-56 | 1.11E-54 | Down |
| LEPR | -2.699893 | 1.36E-57 | 6.18E-56 | Down |
| PRG4 | -4.287296 | 3.33E-55 | 1.40E-53 | Down |
| NR5A2 | -2.497196 | 4.05E-59 | 1.97E-57 | Down |
| KMO | -2.035665 | 4.74E-26 | 5.41E-25 | Down |
| ST6GALNAC5 | -2.340784 | 1.91E-43 | 5.09E-42 | Down |
| KIF17 | -2.139477 | 4.24E-34 | 7.44E-33 | Down |
| MROH9 | -3.117032 | 7.89E-26 | 8.86E-25 | Down |
| SLC5A9 | -4.139638 | 7.98E-76 | 8.18E-74 | Down |
| CASC1 | -2.218592 | 1.74E-18 | 1.19E-17 | Down |
| FILIP1 | -2.803484 | 6.77E-45 | 1.91E-43 | Down |
| ADGB | -3.269697 | 1.64E-15 | 8.83E-15 | Down |
| TCF21 | -4.629885 | 6.20E-109 | 2.07E-106 | Down |
| CASQ2 | -3.569562 | 1.27E-48 | 4.10E-47 | Down |
| DNAH7 | -2.469609 | 1.12E-24 | 1.17E-23 | Down |
| KLF9 | -2.282328 | 3.01E-53 | 1.16E-51 | Down |
| C2orf40 | -3.44887 | 4.16E-27 | 5.12E-26 | Down |
| SLC46A2 | -4.200333 | 1.98E-67 | 1.41E-65 | Down |
| NR4A3 | -3.426126 | 1.68E-50 | 5.83E-49 | Down |
| CSF3R | -2.593786 | 3.67E-34 | 6.45E-33 | Down |
| FLVCR2 | -2.199078 | 5.39E-40 | 1.24E-38 | Down |
| CNRIP1 | -2.016497 | 2.34E-40 | 5.47E-39 | Down |
| CFAP58 | -2.238041 | 2.18E-21 | 1.87E-20 | Down |
| SFRP5 | -2.40016 | 1.71E-09 | 5.39E-09 | Down |
| DUSP1 | -2.870002 | 2.58E-53 | 9.97E-52 | Down |
| TEK | -4.039959 | 7.58E-148 | 1.71E-144 | Down |
| CCDC170 | -2.744029 | 5.42E-28 | 6.98E-27 | Down |
| MYCT1 | -3.153897 | 3.20E-109 | 1.13E-106 | Down |
| KCNJ5 | -2.043823 | 1.17E-16 | 6.91E-16 | Down |
| IQSEC3 | -2.771278 | 1.54E-30 | 2.31E-29 | Down |
| EGR1 | -2.41983 | 2.16E-37 | 4.47E-36 | Down |
| CHRNA2 | -2.852798 | 1.26E-14 | 6.32E-14 | Down |
| ADRA1A | -3.341341 | 3.99E-16 | 2.26E-15 | Down |
| PDLIM2 | -2.222268 | 4.43E-77 | 4.67E-75 | Down |
| TBX2 | -2.059757 | 1.56E-30 | 2.35E-29 | Down |
| TBX4 | -3.16584 | 3.61E-47 | 1.12E-45 | Down |
| CAT | -2.006552 | 9.86E-63 | 5.70E-61 | Down |
| FABP3 | -2.374867 | 1.20E-40 | 2.85E-39 | Down |
| CCRL2 | -2.46913 | 8.55E-43 | 2.22E-41 | Down |
| LDB3 | -2.394984 | 8.38E-42 | 2.07E-40 | Down |
| RAMP3 | -3.634 | 5.65E-124 | 3.51E-121 | Down |
| GLIPR2 | -2.445658 | 3.20E-68 | 2.40E-66 | Down |
| DNAI1 | -2.773382 | 4.06E-15 | 2.11E-14 | Down |
| SFTPA1 | -4.869215 | 9.51E-43 | 2.47E-41 | Down |
| NECAB1 | -2.675807 | 1.57E-30 | 2.35E-29 | Down |
| ADGRE5 | -2.463727 | 1.06E-64 | 6.80E-63 | Down |
| ITIH5 | -2.89064 | 8.14E-31 | 1.24E-29 | Down |
| MMP19 | -2.811279 | 9.98E-51 | 3.53E-49 | Down |
| NR4A1 | -3.293276 | 5.20E-63 | 3.05E-61 | Down |
| PDE1B | -2.066476 | 5.24E-40 | 1.21E-38 | Down |
| NFE2 | -2.181241 | 8.16E-17 | 4.88E-16 | Down |
| C4BPA | -3.587377 | 1.00E-25 | 1.12E-24 | Down |
| C4BPB | -2.084697 | 5.71E-10 | 1.88E-09 | Down |
| DAW1 | -2.988423 | 2.36E-20 | 1.87E-19 | Down |
| MOGAT1 | -3.197725 | 3.32E-29 | 4.60E-28 | Down |
| FAM124B | -2.433759 | 1.74E-42 | 4.48E-41 | Down |
| C20orf85 | -3.306884 | 6.34E-14 | 2.98E-13 | Down |
| ATP8A1 | -2.500741 | 2.26E-37 | 4.65E-36 | Down |
| CEACAM8 | -4.96493 | 1.06E-82 | 1.35E-80 | Down |
| CRISP2 | -2.21058 | 6.34E-08 | 1.70E-07 | Down |
| SLC17A3 | -2.289799 | 5.40E-11 | 1.97E-10 | Down |
| TREM1 | -2.670142 | 1.42E-29 | 2.03E-28 | Down |
| KCNK17 | -2.222744 | 2.48E-20 | 1.96E-19 | Down |
| MYRF | -3.380824 | 3.66E-69 | 2.87E-67 | Down |
| SCGB2A1 | -2.012583 | 1.47E-07 | 3.80E-07 | Down |
| SLC10A2 | -3.989242 | 1.07E-26 | 1.28E-25 | Down |
| TEKT3 | -2.534511 | 1.09E-31 | 1.74E-30 | Down |
| MYH2 | -2.80699 | 2.09E-12 | 8.60E-12 | Down |
| KIR2DL1 | -2.574866 | 2.24E-16 | 1.30E-15 | Down |
| FOSB | -4.652689 | 4.15E-77 | 4.40E-75 | Down |
| FOXA2 | -3.387695 | 1.71E-29 | 2.42E-28 | Down |
| CD93 | -3.107465 | 3.11E-93 | 6.23E-91 | Down |
| BMP2 | -2.473021 | 1.11E-29 | 1.59E-28 | Down |
| FLRT3 | -2.745515 | 3.68E-26 | 4.23E-25 | Down |
| LRRN4 | -4.355298 | 4.20E-64 | 2.60E-62 | Down |
| SIRPD | -2.871414 | 1.81E-33 | 3.12E-32 | Down |
| S1PR4 | -2.483261 | 1.46E-45 | 4.24E-44 | Down |
| MMP24 | -3.039486 | 7.12E-62 | 3.93E-60 | Down |
| F10 | -2.906935 | 2.27E-75 | 2.31E-73 | Down |
| CFP | -2.845386 | 3.48E-57 | 1.56E-55 | Down |
| RHOJ | -2.702339 | 1.45E-86 | 2.21E-84 | Down |
| PZP | -2.702202 | 2.32E-20 | 1.84E-19 | Down |
| OMG | -2.804009 | 7.26E-27 | 8.77E-26 | Down |
| AVPR2 | -2.708056 | 6.88E-30 | 1.00E-28 | Down |
| RGS13 | -2.426344 | 7.20E-24 | 7.19E-23 | Down |
| MASP1 | -2.897788 | 9.53E-36 | 1.81E-34 | Down |
| ATP13A4 | -2.749786 | 5.71E-24 | 5.74E-23 | Down |
| PTPRB | -3.512183 | 3.94E-141 | 5.46E-138 | Down |
| KLF2 | -3.307391 | 3.49E-95 | 7.78E-93 | Down |
| F2RL3 | -2.807167 | 1.84E-58 | 8.75E-57 | Down |
| GNG11 | -3.012589 | 5.28E-83 | 6.90E-81 | Down |
| STEAP4 | -3.17675 | 1.12E-33 | 1.94E-32 | Down |
| ZFP36 | -2.869409 | 3.68E-68 | 2.72E-66 | Down |
| KDR | -2.677819 | 2.06E-69 | 1.63E-67 | Down |
| MGAT3 | -2.101092 | 7.65E-15 | 3.90E-14 | Down |
| DOCK4 | -2.217455 | 6.62E-54 | 2.63E-52 | Down |
| CDHR3 | -2.710567 | 3.94E-35 | 7.23E-34 | Down |
| CGNL1 | -3.154211 | 3.88E-74 | 3.82E-72 | Down |
| DLL4 | -2.345572 | 4.30E-67 | 2.98E-65 | Down |
| ALDH1A2 | -2.905374 | 2.28E-36 | 4.44E-35 | Down |
| ACKR4 | -2.768957 | 1.67E-57 | 7.54E-56 | Down |
| ATP1B2 | -2.17858 | 1.06E-30 | 1.61E-29 | Down |
| ADCY4 | -2.474481 | 7.21E-65 | 4.70E-63 | Down |
| RNASE1 | -2.809765 | 8.60E-51 | 3.05E-49 | Down |
| CDO1 | -2.93505 | 9.59E-31 | 1.46E-29 | Down |
| ARHGEF6 | -2.117755 | 8.67E-41 | 2.06E-39 | Down |
| ART1 | -3.24479 | 1.15E-37 | 2.39E-36 | Down |
| PLPPR3 | -2.329857 | 3.23E-13 | 1.43E-12 | Down |
| KCNA5 | -3.635951 | 1.59E-51 | 5.80E-50 | Down |
| STARD8 | -2.986257 | 1.21E-91 | 2.19E-89 | Down |
| DPP6 | -2.010737 | 3.50E-07 | 8.66E-07 | Down |
| USHBP1 | -3.1254 | 2.79E-116 | 1.36E-113 | Down |
| CACNG6 | -2.717618 | 2.93E-12 | 1.19E-11 | Down |
| IQCN | -2.956321 | 8.49E-46 | 2.48E-44 | Down |
| HRC | -2.092458 | 1.20E-19 | 8.98E-19 | Down |
| GATA5 | -2.061231 | 5.66E-11 | 2.06E-10 | Down |
| GMFG | -2.091484 | 2.69E-37 | 5.52E-36 | Down |
| RGN | -3.053152 | 6.42E-45 | 1.82E-43 | Down |
| AKAP12 | -2.400671 | 3.28E-27 | 4.05E-26 | Down |
| SYNE1 | -3.085279 | 6.34E-83 | 8.21E-81 | Down |
| TTLL9 | -2.20729 | 4.14E-17 | 2.54E-16 | Down |
| COX4I2 | -2.845469 | 8.53E-63 | 4.95E-61 | Down |
| HIGD1B | -3.679943 | 2.03E-133 | 2.04E-130 | Down |
| ADGRE3 | -3.174488 | 1.13E-46 | 3.45E-45 | Down |
| SH3BP5 | -2.220858 | 3.01E-43 | 7.99E-42 | Down |
| GALNT15 | -2.035955 | 1.93E-16 | 1.13E-15 | Down |
| NAPSA | -3.954274 | 1.18E-35 | 2.22E-34 | Down |
| AOC3 | -3.731067 | 1.24E-88 | 2.11E-86 | Down |
| RAMP2 | -3.166821 | 5.92E-138 | 7.11E-135 | Down |
| TMEM204 | -2.306001 | 2.17E-59 | 1.07E-57 | Down |
| FMO5 | -3.399501 | 1.17E-70 | 9.65E-69 | Down |
| RAI2 | -2.691229 | 3.88E-66 | 2.62E-64 | Down |
| NR0B2 | -3.940253 | 1.37E-29 | 1.96E-28 | Down |
| CNGA4 | -2.908768 | 5.71E-24 | 5.74E-23 | Down |
| RGS22 | -2.91174 | 4.54E-28 | 5.88E-27 | Down |
| HSPA12B | -3.002914 | 1.41E-86 | 2.17E-84 | Down |
| ALOX5AP | -2.853154 | 1.20E-54 | 4.91E-53 | Down |
| STOML3 | -2.587 | 3.75E-13 | 1.65E-12 | Down |
| KL | -3.501604 | 1.22E-64 | 7.79E-63 | Down |
| STARD13 | -2.429649 | 1.02E-55 | 4.34E-54 | Down |
| PRAM1 | -2.826626 | 6.59E-58 | 3.07E-56 | Down |
| PDZD2 | -2.131469 | 2.37E-16 | 1.37E-15 | Down |
| GIMAP6 | -2.731656 | 1.08E-59 | 5.47E-58 | Down |
| GIMAP4 | -2.060038 | 2.03E-32 | 3.33E-31 | Down |
| LRRIQ1 | -2.10127 | 5.26E-14 | 2.49E-13 | Down |
| SFTPD | -4.139495 | 2.37E-42 | 6.02E-41 | Down |
| DYDC2 | -2.535994 | 1.52E-17 | 9.64E-17 | Down |
| CA1 | -2.876155 | 8.72E-37 | 1.74E-35 | Down |
| LYVE1 | -4.019586 | 2.35E-109 | 8.48E-107 | Down |
| DUSP26 | -2.204174 | 6.93E-17 | 4.17E-16 | Down |
| PEBP4 | -4.472564 | 4.03E-45 | 1.15E-43 | Down |
| CNTN6 | -4.889221 | 6.81E-58 | 3.16E-56 | Down |
| CHIA | -4.579022 | 4.22E-38 | 9.01E-37 | Down |
| CD101 | -2.326067 | 6.08E-58 | 2.87E-56 | Down |
| CABLES1 | -2.414224 | 1.19E-47 | 3.74E-46 | Down |
| ADAMTS8 | -5.087364 | 1.17E-101 | 3.24E-99 | Down |
| KLB | -3.746664 | 2.38E-67 | 1.68E-65 | Down |
| FAM189A2 | -3.967644 | 1.36E-71 | 1.20E-69 | Down |
| CD36 | -3.08056 | 2.33E-47 | 7.23E-46 | Down |
| ADGRB3 | -2.047821 | 1.56E-10 | 5.44E-10 | Down |
| STX11 | -2.781207 | 6.81E-64 | 4.16E-62 | Down |
| DYSF | -2.109355 | 2.42E-35 | 4.50E-34 | Down |
| CAPN9 | -3.533153 | 8.38E-49 | 2.73E-47 | Down |
| SLC19A3 | -3.452476 | 4.64E-63 | 2.74E-61 | Down |
| CYP27A1 | -2.370449 | 1.82E-34 | 3.23E-33 | Down |
| STAB2 | -2.055043 | 3.27E-20 | 2.56E-19 | Down |
| USP44 | -2.203161 | 2.18E-26 | 2.56E-25 | Down |
| DRAM1 | -2.557216 | 4.44E-71 | 3.76E-69 | Down |
| LMO7 | -2.791591 | 3.33E-99 | 8.46E-97 | Down |
| SCEL | -2.690902 | 3.81E-22 | 3.45E-21 | Down |
| EDNRB | -4.315106 | 5.72E-144 | 9.38E-141 | Down |
| IL6 | -2.97517 | 1.08E-26 | 1.29E-25 | Down |
| NKX2-1 | -3.004552 | 7.81E-20 | 5.98E-19 | Down |
| TM6SF1 | -2.164543 | 2.97E-35 | 5.49E-34 | Down |
| SCN7A | -4.342942 | 1.66E-71 | 1.46E-69 | Down |
| GYPC | -2.32529 | 3.46E-44 | 9.44E-43 | Down |
| TLR4 | -2.164388 | 2.11E-26 | 2.48E-25 | Down |
| WDR38 | -3.112944 | 1.10E-19 | 8.31E-19 | Down |
| HEMGN | -2.535905 | 1.84E-13 | 8.31E-13 | Down |
| ENPP2 | -2.456886 | 4.50E-37 | 9.10E-36 | Down |
| IL33 | -2.066005 | 5.82E-16 | 3.26E-15 | Down |
| TTC29 | -3.088549 | 1.07E-14 | 5.36E-14 | Down |
| ARRB1 | -3.204566 | 9.99E-99 | 2.47E-96 | Down |
| SLCO2B1 | -2.325357 | 2.38E-29 | 3.33E-28 | Down |
| LRRC32 | -3.038958 | 1.20E-91 | 2.19E-89 | Down |
| TRPC6 | -2.230943 | 1.50E-26 | 1.78E-25 | Down |
| SMAD6 | -2.67784 | 6.93E-58 | 3.21E-56 | Down |
| DUOX1 | -2.09909 | 1.53E-32 | 2.51E-31 | Down |
| GCOM1 | -3.297344 | 1.19E-46 | 3.63E-45 | Down |
| GIPC2 | -2.278778 | 2.35E-17 | 1.47E-16 | Down |
| ARHGAP29 | -2.337989 | 9.76E-35 | 1.76E-33 | Down |
| DNASE2B | -3.09098 | 3.52E-24 | 3.59E-23 | Down |
| RBP4 | -2.989874 | 7.05E-23 | 6.64E-22 | Down |
| PLA2G12B | -3.860606 | 1.30E-27 | 1.64E-26 | Down |
| OIT3 | -2.40052 | 6.54E-30 | 9.54E-29 | Down |
| AOX1 | -2.766829 | 4.17E-44 | 1.13E-42 | Down |
| HECW2 | -2.339978 | 1.18E-74 | 1.17E-72 | Down |
| SECISBP2L | -2.300908 | 1.86E-129 | 1.52E-126 | Down |
| RASGEF1B | -2.01385 | 7.88E-38 | 1.66E-36 | Down |
| MMRN1 | -3.407687 | 1.85E-54 | 7.57E-53 | Down |
| TRPC3 | -2.246442 | 3.04E-29 | 4.23E-28 | Down |
| CDKL2 | -2.691381 | 2.38E-39 | 5.35E-38 | Down |
| ANXA3 | -2.218766 | 1.22E-27 | 1.54E-26 | Down |
| SLC39A8 | -3.877925 | 5.38E-132 | 4.85E-129 | Down |
| DHH | -2.331335 | 7.73E-30 | 1.12E-28 | Down |
| ACVRL1 | -3.212889 | 1.90E-146 | 3.80E-143 | Down |
| SSTR1 | -3.720309 | 4.78E-37 | 9.65E-36 | Down |
| SLC24A4 | -2.636787 | 2.59E-26 | 3.02E-25 | Down |
| FBLN5 | -3.050855 | 4.58E-76 | 4.75E-74 | Down |
| CYP1A1 | -4.089482 | 7.54E-36 | 1.44E-34 | Down |
| CCDC33 | -2.932878 | 3.76E-15 | 1.96E-14 | Down |
| CYP1A2 | -3.755364 | 3.70E-17 | 2.28E-16 | Down |
| NTRK3 | -2.041687 | 2.79E-09 | 8.57E-09 | Down |
| MYLK3 | -2.271271 | 6.42E-19 | 4.55E-18 | Down |
| CMTM2 | -2.453991 | 3.85E-27 | 4.74E-26 | Down |
| MYOCD | -3.694643 | 3.12E-46 | 9.31E-45 | Down |
| UNC45B | -3.453082 | 3.02E-51 | 1.09E-49 | Down |
| LRRC46 | -2.742381 | 1.19E-31 | 1.90E-30 | Down |
| ABCA8 | -4.01559 | 2.52E-68 | 1.91E-66 | Down |
| GATA6 | -2.698515 | 1.08E-58 | 5.17E-57 | Down |
| ZMYND15 | -2.009533 | 1.33E-38 | 2.90E-37 | Down |
| MAPK4 | -2.118425 | 8.98E-10 | 2.90E-09 | Down |
| NLRP12 | -2.997674 | 3.99E-36 | 7.71E-35 | Down |
| CFAP74 | -2.458719 | 6.45E-17 | 3.88E-16 | Down |
| PRDM16 | -2.540434 | 3.65E-26 | 4.20E-25 | Down |
| FCN3 | -5.350323 | 3.44E-129 | 2.59E-126 | Down |
| CYR61 | -2.408783 | 6.41E-38 | 1.35E-36 | Down |
| CYP4B1 | -4.718102 | 2.64E-54 | 1.07E-52 | Down |
| C1orf162 | -2.555253 | 3.07E-54 | 1.24E-52 | Down |
| ITGA10 | -2.375548 | 6.67E-41 | 1.60E-39 | Down |
| GPA33 | -4.861395 | 1.08E-93 | 2.24E-91 | Down |
| RXRG | -3.696125 | 1.62E-54 | 6.63E-53 | Down |
| RGS5 | -2.222393 | 3.99E-42 | 9.98E-41 | Down |
| RORC | -2.433985 | 1.62E-17 | 1.03E-16 | Down |
| CGN | -2.026182 | 7.67E-29 | 1.04E-27 | Down |
| ADAMTSL4 | -2.416283 | 5.07E-33 | 8.50E-32 | Down |
| SELENBP1 | -3.742822 | 4.74E-74 | 4.65E-72 | Down |
| LEFTY2 | -3.420624 | 8.90E-35 | 1.61E-33 | Down |
| REN | -2.315551 | 1.41E-21 | 1.23E-20 | Down |
| AFF3 | -3.579589 | 1.63E-56 | 7.13E-55 | Down |
| GPR17 | -3.176987 | 2.56E-38 | 5.54E-37 | Down |
| SCN1A | -3.232141 | 9.75E-27 | 1.17E-25 | Down |
| ZNF385B | -3.768889 | 3.03E-47 | 9.36E-46 | Down |
| RBMS3 | -2.102074 | 7.22E-43 | 1.89E-41 | Down |
| CSRNP1 | -3.74971 | 3.68E-168 | 2.21E-164 | Down |
| ITGA9 | -2.247907 | 5.73E-32 | 9.24E-31 | Down |
| STAC | -2.904637 | 3.82E-32 | 6.20E-31 | Down |
| PLA1A | -2.922971 | 3.04E-46 | 9.09E-45 | Down |
| AGTR1 | -3.368642 | 4.54E-49 | 1.51E-47 | Down |
| SLIT2 | -2.501398 | 1.48E-36 | 2.91E-35 | Down |
| ANK2 | -2.034545 | 1.16E-23 | 1.14E-22 | Down |
| ROPN1L | -2.700999 | 6.97E-21 | 5.73E-20 | Down |
| OTULINL | -2.817078 | 8.84E-79 | 9.78E-77 | Down |
| C1QTNF2 | -2.426828 | 2.04E-44 | 5.64E-43 | Down |
| DCDC2 | -3.314398 | 1.37E-44 | 3.82E-43 | Down |
| DAAM2 | -2.56805 | 1.23E-49 | 4.17E-48 | Down |
| HMGCLL1 | -2.384152 | 1.95E-13 | 8.79E-13 | Down |
| TCTE1 | -2.993605 | 1.82E-21 | 1.57E-20 | Down |
| VIP | -2.216302 | 1.11E-20 | 9.02E-20 | Down |
| SLC22A3 | -2.369288 | 5.33E-18 | 3.51E-17 | Down |
| DENND2A | -2.764836 | 1.15E-66 | 7.91E-65 | Down |
| CXorf36 | -2.761033 | 3.47E-85 | 4.97E-83 | Down |
| AWAT2 | -2.933995 | 5.13E-15 | 2.65E-14 | Down |
| NXF3 | -2.37065 | 7.64E-13 | 3.27E-12 | Down |
| DOK2 | -2.431572 | 3.59E-46 | 1.07E-44 | Down |
| RSPO2 | -3.251555 | 1.19E-19 | 8.96E-19 | Down |
| SHC3 | -2.624327 | 1.24E-44 | 3.45E-43 | Down |
| SNX30 | -2.015572 | 3.11E-77 | 3.32E-75 | Down |
| TMEM236 | -2.504111 | 8.71E-38 | 1.83E-36 | Down |
| ST8SIA6 | -2.677673 | 2.81E-26 | 3.26E-25 | Down |
| FAM13C | -2.409281 | 6.89E-42 | 1.71E-40 | Down |
| ANKRD1 | -5.489427 | 5.83E-75 | 5.88E-73 | Down |
| HABP2 | -2.223918 | 7.14E-13 | 3.06E-12 | Down |
| CYP17A1 | -2.47639 | 3.90E-29 | 5.38E-28 | Down |
| SCGB1A1 | -4.323024 | 5.92E-27 | 7.19E-26 | Down |
| CCDC81 | -2.103457 | 1.75E-35 | 3.28E-34 | Down |
| GRIK4 | -2.902822 | 2.18E-36 | 4.26E-35 | Down |
| GGTLC1 | -5.645546 | 3.45E-64 | 2.16E-62 | Down |
| TMC2 | -2.254537 | 1.06E-18 | 7.37E-18 | Down |
| MS4A3 | -2.743776 | 3.03E-17 | 1.88E-16 | Down |
| MS4A2 | -2.731433 | 2.87E-35 | 5.32E-34 | Down |
| ESAM | -3.145785 | 9.28E-134 | 9.85E-131 | Down |
| SCN2B | -2.507558 | 5.03E-20 | 3.90E-19 | Down |
| JPH2 | -2.060755 | 1.75E-20 | 1.39E-19 | Down |
| KLRF1 | -2.038939 | 1.75E-17 | 1.10E-16 | Down |
| CLEC1A | -2.109026 | 5.38E-21 | 4.48E-20 | Down |
| FXYD4 | -2.08858 | 1.07E-07 | 2.78E-07 | Down |
| PCDH15 | -2.179132 | 5.24E-09 | 1.57E-08 | Down |
| GPM6A | -5.12015 | 1.71E-67 | 1.22E-65 | Down |
| SPATA4 | -2.669992 | 1.32E-16 | 7.80E-16 | Down |
| C2orf50 | -2.02364 | 4.12E-14 | 1.97E-13 | Down |
| ENKUR | -2.361685 | 1.81E-19 | 1.34E-18 | Down |
| NR3C2 | -2.532478 | 1.02E-28 | 1.37E-27 | Down |
| FLI1 | -2.376552 | 4.47E-53 | 1.71E-51 | Down |
| WWC2 | -2.985606 | 5.77E-115 | 2.67E-112 | Down |
| TMEM132D | -2.510354 | 7.89E-09 | 2.32E-08 | Down |
| TMEM163 | -2.388087 | 1.04E-27 | 1.32E-26 | Down |
| HSPB8 | -2.174211 | 1.02E-23 | 1.01E-22 | Down |
| GUCY1A2 | -2.761941 | 3.56E-58 | 1.69E-56 | Down |
| PLEKHH2 | -2.029626 | 3.23E-25 | 3.49E-24 | Down |
| SPARCL1 | -2.096089 | 5.92E-25 | 6.31E-24 | Down |
| CAPSL | -2.880946 | 5.58E-16 | 3.13E-15 | Down |
| TCTEX1D1 | -2.612677 | 6.05E-33 | 1.01E-31 | Down |
| WDR78 | -2.19952 | 1.58E-25 | 1.74E-24 | Down |
| SLC16A12 | -2.254795 | 2.68E-13 | 1.19E-12 | Down |
| LMNTD1 | -2.283841 | 9.09E-10 | 2.94E-09 | Down |
| BANK1 | -2.098273 | 1.32E-27 | 1.66E-26 | Down |
| ACOXL | -3.595166 | 1.30E-84 | 1.81E-82 | Down |
| NR4A2 | -2.462886 | 1.12E-36 | 2.21E-35 | Down |
| FAM81B | -2.964486 | 5.13E-19 | 3.66E-18 | Down |
| LPCAT1 | -2.27476 | 3.95E-37 | 8.01E-36 | Down |
| C16orf89 | -4.127294 | 4.74E-47 | 1.46E-45 | Down |
| FAM92B | -2.884124 | 6.94E-21 | 5.71E-20 | Down |
| PID1 | -2.534702 | 6.87E-38 | 1.45E-36 | Down |
| LGI4 | -2.065257 | 1.44E-36 | 2.84E-35 | Down |
| DDAH1 | -2.099854 | 3.90E-37 | 7.93E-36 | Down |
| ANKRD29 | -2.005332 | 1.65E-19 | 1.22E-18 | Down |
| DNAAF1 | -2.602995 | 2.39E-20 | 1.89E-19 | Down |
| ROBO4 | -3.73097 | 1.88E-178 | 3.39E-174 | Down |
| NRGN | -3.037081 | 4.94E-50 | 1.69E-48 | Down |
| ABI3BP | -3.137228 | 6.02E-46 | 1.77E-44 | Down |
| ANGPT1 | -2.781382 | 9.87E-39 | 2.16E-37 | Down |
| ABCA9 | -2.452925 | 6.03E-49 | 1.99E-47 | Down |
| ABCA6 | -2.416598 | 1.29E-41 | 3.16E-40 | Down |
| DISP1 | -2.170785 | 2.18E-59 | 1.08E-57 | Down |
| FAM167A | -2.416112 | 1.42E-20 | 1.14E-19 | Down |
| PGM5 | -3.726461 | 2.21E-79 | 2.52E-77 | Down |
| CCDC173 | -2.806617 | 3.20E-37 | 6.54E-36 | Down |
| SORBS2 | -2.553693 | 3.17E-32 | 5.15E-31 | Down |
| PDE1C | -3.182993 | 2.34E-63 | 1.39E-61 | Down |
| JAM2 | -3.171971 | 3.79E-112 | 1.55E-109 | Down |
| ADAMTS1 | -2.071555 | 1.10E-29 | 1.58E-28 | Down |
| FGD5 | -2.828335 | 3.53E-73 | 3.36E-71 | Down |
| PIEZO2 | -2.071364 | 1.07E-20 | 8.66E-20 | Down |
| PTPRN2 | -2.345974 | 2.39E-34 | 4.22E-33 | Down |
| SLC7A7 | -2.018736 | 8.43E-28 | 1.07E-26 | Down |
| GRIA1 | -4.545067 | 3.93E-52 | 1.45E-50 | Down |
| XAGE2 | -3.148894 | 6.65E-16 | 3.71E-15 | Down |
| VSIG4 | -3.056284 | 8.23E-43 | 2.14E-41 | Down |
| ALS2CR12 | -2.093445 | 2.21E-24 | 2.27E-23 | Down |
| GNA14 | -2.805605 | 1.33E-44 | 3.71E-43 | Down |
| WIF1 | -2.058477 | 8.60E-06 | 1.84E-05 | Down |
| UGT2B4 | -2.922452 | 2.61E-14 | 1.27E-13 | Down |
| CFAP161 | -2.334238 | 6.51E-24 | 6.53E-23 | Down |
| ADAMTSL3 | -2.517629 | 8.39E-23 | 7.84E-22 | Down |
| FGF18 | -2.490759 | 4.24E-24 | 4.30E-23 | Down |
| B3GNT7 | -2.237963 | 1.83E-25 | 2.00E-24 | Down |
| C1orf158 | -3.409504 | 6.63E-16 | 3.70E-15 | Down |
| CACNA1D | -2.87245 | 2.55E-38 | 5.51E-37 | Down |
| HYDIN | -2.865378 | 2.85E-24 | 2.92E-23 | Down |
| AFAP1L1 | -2.03078 | 1.29E-40 | 3.05E-39 | Down |
| ERG | -2.499591 | 5.90E-72 | 5.24E-70 | Down |
| SLC34A2 | -3.794576 | 1.55E-35 | 2.91E-34 | Down |
| DRC1 | -2.309552 | 7.03E-12 | 2.77E-11 | Down |
| CIB4 | -2.556089 | 1.99E-18 | 1.36E-17 | Down |
| TRIM63 | -2.110807 | 4.99E-09 | 1.49E-08 | Down |
| ESYT3 | -2.882392 | 1.65E-42 | 4.26E-41 | Down |
| COLEC12 | -2.748574 | 3.01E-44 | 8.25E-43 | Down |
| SHROOM4 | -2.847615 | 1.37E-63 | 8.30E-62 | Down |
| CATIP | -2.756181 | 1.88E-30 | 2.81E-29 | Down |
| KCNB1 | -2.579415 | 9.51E-21 | 7.74E-20 | Down |
| AHCYL2 | -2.544217 | 1.67E-83 | 2.27E-81 | Down |
| ALAS2 | -4.564271 | 1.55E-102 | 4.44E-100 | Down |
| ITLN2 | -4.259957 | 1.22E-48 | 3.95E-47 | Down |
| ADAMTS4 | -2.043257 | 4.23E-22 | 3.81E-21 | Down |
| SCUBE1 | -3.644882 | 4.21E-36 | 8.13E-35 | Down |
| PADI4 | -3.419438 | 1.23E-47 | 3.84E-46 | Down |
| STARD9 | -2.355527 | 1.31E-42 | 3.37E-41 | Down |
| CCDC17 | -2.28472 | 6.01E-19 | 4.27E-18 | Down |
| DRC7 | -2.94398 | 8.31E-19 | 5.85E-18 | Down |
| ACE | -2.624782 | 7.05E-73 | 6.59E-71 | Down |
| LRRC36 | -4.269343 | 1.12E-96 | 2.65E-94 | Down |
| TPPP3 | -2.919719 | 3.85E-40 | 8.95E-39 | Down |
| AGRP | -4.445195 | 1.46E-86 | 2.21E-84 | Down |
| CPAMD8 | -3.299773 | 5.99E-55 | 2.49E-53 | Down |
| RSPH1 | -2.877472 | 5.03E-23 | 4.79E-22 | Down |
| TMEM190 | -3.063075 | 7.83E-21 | 6.42E-20 | Down |
| JAML | -2.567665 | 4.14E-46 | 1.23E-44 | Down |
| PTH1R | -2.934434 | 1.27E-67 | 9.15E-66 | Down |
| MYL3 | -2.63266 | 4.50E-41 | 1.09E-39 | Down |
| LRRC71 | -3.000552 | 4.40E-22 | 3.96E-21 | Down |
| AZGP1 | -2.314685 | 7.26E-11 | 2.61E-10 | Down |
| FGFR4 | -3.182725 | 7.07E-71 | 5.88E-69 | Down |
| CYP3A7 | -2.292418 | 1.63E-29 | 2.31E-28 | Down |
| HK3 | -2.106295 | 9.81E-24 | 9.73E-23 | Down |
| SCGB3A1 | -3.519742 | 1.65E-24 | 1.71E-23 | Down |
| COX7A1 | -2.032711 | 3.57E-26 | 4.12E-25 | Down |
| SIGLEC11 | -3.082582 | 4.04E-37 | 8.18E-36 | Down |
| CD300LG | -6.290681 | 1.02E-104 | 3.16E-102 | Down |
| GRASP | -2.46996 | 5.45E-40 | 1.25E-38 | Down |
| SCIMP | -2.269149 | 2.73E-28 | 3.58E-27 | Down |
| BCL6B | -2.743959 | 3.02E-85 | 4.36E-83 | Down |
| TNFSF13 | -2.17198 | 1.07E-62 | 6.14E-61 | Down |
| PPP1R32 | -2.072647 | 2.33E-28 | 3.07E-27 | Down |
| ITIH3 | -2.755827 | 1.77E-46 | 5.35E-45 | Down |
| TAL1 | -3.016941 | 3.92E-51 | 1.41E-49 | Down |
| SYNC | -2.067959 | 5.19E-39 | 1.15E-37 | Down |
| UBXN10 | -2.512547 | 1.22E-20 | 9.81E-20 | Down |
| ALPL | -2.785112 | 1.65E-36 | 3.24E-35 | Down |
| C1orf87 | -3.399343 | 1.43E-15 | 7.75E-15 | Down |
| NEXN | -2.041945 | 4.10E-33 | 6.93E-32 | Down |
| ADGRL4 | -2.248307 | 1.67E-53 | 6.49E-52 | Down |
| NTNG1 | -2.718603 | 8.78E-16 | 4.84E-15 | Down |
| WDR63 | -2.657015 | 7.11E-26 | 8.01E-25 | Down |
| KCNT2 | -2.942814 | 4.07E-43 | 1.07E-41 | Down |
| NLRP3 | -2.303997 | 4.92E-32 | 7.95E-31 | Down |
| TRIM58 | -2.294123 | 5.34E-09 | 1.60E-08 | Down |
| FCGR3B | -2.229071 | 1.47E-11 | 5.63E-11 | Down |
| LRRC52 | -2.737121 | 5.49E-14 | 2.59E-13 | Down |
| ATF3 | -2.168847 | 4.93E-28 | 6.37E-27 | Down |
| PIGR | -3.617021 | 1.18E-35 | 2.22E-34 | Down |
| TEKT4 | -2.447686 | 1.47E-15 | 7.92E-15 | Down |
| SPATA18 | -2.111089 | 3.68E-23 | 3.53E-22 | Down |
| NOSTRIN | -3.312948 | 1.03E-124 | 6.61E-122 | Down |
| CFAP221 | -3.119342 | 2.87E-31 | 4.49E-30 | Down |
| HPGDS | -2.613129 | 2.24E-47 | 6.99E-46 | Down |
| C1QTNF7 | -4.019889 | 1.68E-78 | 1.84E-76 | Down |
| TDRD10 | -3.064318 | 1.15E-42 | 2.98E-41 | Down |
| C1orf189 | -2.792432 | 5.65E-18 | 3.72E-17 | Down |
| LMOD3 | -2.907185 | 3.20E-61 | 1.71E-59 | Down |
| PROK2 | -2.536919 | 9.12E-18 | 5.91E-17 | Down |
| LMOD1 | -2.862604 | 3.00E-53 | 1.15E-51 | Down |
| CXCR1 | -3.35389 | 1.58E-30 | 2.37E-29 | Down |
| NEK10 | -2.304769 | 4.16E-20 | 3.24E-19 | Down |
| CCDC141 | -4.038926 | 4.12E-66 | 2.77E-64 | Down |
| IHH | -4.445707 | 3.47E-54 | 1.39E-52 | Down |
| TGFBR2 | -2.803913 | 2.86E-90 | 4.95E-88 | Down |
| FCRL1 | -2.262712 | 2.95E-12 | 1.20E-11 | Down |
| MNDA | -2.245558 | 2.80E-29 | 3.91E-28 | Down |
| EFHB | -2.53073 | 2.89E-26 | 3.34E-25 | Down |
| PTX3 | -2.162308 | 4.52E-14 | 2.16E-13 | Down |
| DNASE1L3 | -2.571326 | 7.93E-26 | 8.90E-25 | Down |
| CXCL3 | -2.957503 | 2.98E-35 | 5.51E-34 | Down |
| PPBP | -3.107042 | 7.56E-15 | 3.86E-14 | Down |
| PF4 | -3.427434 | 1.50E-32 | 2.47E-31 | Down |
| CPA3 | -2.295798 | 4.63E-23 | 4.42E-22 | Down |
| TM4SF18 | -2.358185 | 5.18E-41 | 1.25E-39 | Down |
| CLEC3B | -4.836098 | 1.85E-154 | 8.32E-151 | Down |
| SLC6A20 | -3.038421 | 3.85E-30 | 5.66E-29 | Down |
| LRRC2 | -2.647159 | 2.32E-30 | 3.45E-29 | Down |
| DNALI1 | -2.69055 | 2.51E-30 | 3.72E-29 | Down |
| KLF15 | -2.37055 | 2.21E-29 | 3.11E-28 | Down |
| CFAP100 | -2.47546 | 7.05E-15 | 3.61E-14 | Down |
| SGMS2 | -2.941656 | 8.78E-85 | 1.25E-82 | Down |
| EMCN | -3.726123 | 6.50E-104 | 1.95E-101 | Down |
| CAMP | -2.666434 | 8.24E-20 | 6.28E-19 | Down |
| HPGD | -2.56141 | 6.67E-22 | 5.96E-21 | Down |
| NPY1R | -2.056384 | 5.87E-11 | 2.13E-10 | Down |
| NPY5R | -2.10972 | 2.68E-07 | 6.71E-07 | Down |
| HHIP | -3.838531 | 1.83E-33 | 3.14E-32 | Down |
| ZNF474 | -2.080117 | 2.15E-16 | 1.24E-15 | Down |
| RANBP3L | -2.10283 | 1.07E-14 | 5.38E-14 | Down |
| SCGB3A2 | -2.77108 | 1.76E-14 | 8.70E-14 | Down |
| CITED2 | -2.622648 | 2.97E-66 | 2.02E-64 | Down |
| PI16 | -3.582429 | 3.00E-31 | 4.70E-30 | Down |
| BMPER | -2.06536 | 4.28E-13 | 1.87E-12 | Down |
| KIF6 | -2.032325 | 1.45E-15 | 7.86E-15 | Down |
| SOX17 | -3.584731 | 4.25E-116 | 2.02E-113 | Down |
| DLC1 | -4.050241 | 7.01E-131 | 6.02E-128 | Down |
| C7orf57 | -2.178427 | 3.40E-14 | 1.64E-13 | Down |
| GPR146 | -2.610259 | 4.22E-83 | 5.56E-81 | Down |
[truncated: 125,288 more chars]
